# Machine learning-based identification of leptin-associated biomarkers and prognostic prediction models in sepsis

**DOI:** 10.3389/fcimb.2025.1630446

**Published:** 2025-09-29

**Authors:** Xiaoshu Liu, Junmei Song, Yi Liao, Liqing Yang, Caiyu Jiang, Qiunan Zuo

**Affiliations:** ^1^ Department of Respiratory and Critical Care Medicine, Sichuan Provincial People’s Hospital, School of Medicine, University of Electronic Science and Technology of China, Chengdu, China; ^2^ Ultrasound in Cardiac Electrophysiology and Biomechanics Key Laboratory of Sichuan Province, Sichuan Clinical Research Center for Cardiovascular Disease, Sichuan Provincial People’s Hospital, University of Electronic Science and Technology of China, Chengdu, China; ^3^ Department of Cardiovascular Ultrasound & Noninvasive Cardiology, Sichuan Provincial People’s Hospital, University of Electronic Science and Technology of China, Chengdu, China; ^4^ Department of Geriatric Respiratory, Sichuan Provincial People’s Hospital, School of Medicine, University of Electronic Science and Technology of China, Chengdu, China

**Keywords:** leptin, sepsis, machine learning, prognosis, diagnosis

## Abstract

**Background:**

Leptin has been implicated in the prognosis of sepsis, yet its mechanistic role remains unclear. This study aimed to develop leptin-associated diagnostic and prognostic models for sepsis and identify potential biomarkers using machine learning approaches.

**Methods:**

Non-negative matrix factorization (NMF) was used to identify leptin-related molecular subtypes of sepsis. Weighted gene co-expression network analysis (WGCNA) determined relevant gene modules and hub genes. Differentially expressed genes (DEGs) between sepsis patients and controls were intersected with WGCNA results to refine key genes. Based on these analyses, a prognostic classification model predicting 28-day mortality was developed using the Least Absolute Shrinkage and Selection Operator and Random Forest algorithms, while a time-to-event prognostic model was constructed with Random Survival Forest and Gradient Boosting Machine. Single-cell RNA sequencing was performed to assess expression patterns of core genes across immune cell types. Expression validation was conducted using qPCR and Western blotting.

**Results:**

Three leptin-associated sepsis subtypes with distinct prognoses were identified. The pink and salmon modules from WGCNA were significantly associated with sepsis. Seventy core genes were selected from the DEGs and WGCNA intersection. The prognostic classification model and the time-to-event prognostic model demonstrated strong predictive performance in both the training and external validation cohorts. TFRC and PILRA were consistently highlighted through machine learning, single-cell data, and experimental validation as potential biomarkers.

**Conclusion:**

We established leptin-related prognostic models for sepsis using integrated machine learning. TFRC and PILRA may serve as promising biomarkers, offering insights into sepsis heterogeneity and clinical management.

## Introduction

Sepsis is a systemic inflammatory response syndrome triggered by infection, which can lead to organ dysfunction and even death in severe cases (Jacobi, 2022). According to recent studies, the global age-standardized incidence rate of sepsis is 677.5 cases per 100,000 population. The highest incidence rates are observed in sub-Saharan Africa, Oceania, and South Asia. In high-income countries, the case fatality rate of sepsis ranges from 15% to 25%, whereas in low- and middle-income countries, it often exceeds 40%, with the mortality rate of septic shock reaching as high as 50% (La Via et al., 2024). In recent years, the acute-phase survival rate of sepsis has improved globally; however, mortality remains significant. A multicenter prospective study involving 44 intensive care units (ICUs) across China reported an ICU mortality rate of 27.2% and an in-hospital mortality rate of 33.0% among sepsis patients. For those with septic shock, the ICU mortality rate increased to 39.0%, and the in-hospital mortality rate rose to 44.4% (Wang et al., 2020). Additionally, another study from China found that the 30-day mortality rate for sepsis patients was 29.5%, while for patients with septic shock, it reached 37.3% (Liu et al., 2022).

Early recognition of sepsis remains a major clinical challenge owing to its heterogeneous manifestations and rapid progression. Traditional scoring systems, such as the Sequential Organ Failure Assessment (SOFA) (Qiu et al., 2023) and Acute Physiology and Chronic Health Evaluation II (APACHE II) (Yuan et al., 2024), are commonly used to assess disease severity and predict outcomes in sepsis patients. However, these tools have limitations in predictive accuracy and may not fully capture the complex pathophysiological processes involved in sepsis. For instance, Zhang et al. developed a 28-day mortality prediction model using MIMIC-IV data, identifying variables such as ICU stay, hemoglobin, albumin, activated partial thromboplastin time, and total bilirubin, achieving an area under the receiver operating characteristic curve (AUC) of 0.904 (Zhang et al., 2024). Similarly, Xie et al. established a model based on IL-6, lactate, and procalcitonin, with AUCs of 0.849 and 0.828 in the training and validation cohorts, respectively (Xie et al., 2023). These results highlight the need for more precise and individualized prognostic tools in sepsis care.

Machine learning (ML) techniques, particularly when applied to high-dimensional sequencing data, provide more accurate and detailed methods for prognostic prediction and have been applied to a variety of diseases (Liao et al., 2025; Alshwayyat et al., 2025; López Gordo et al., 2025). For instance, a study has demonstrated that ML models, such as Random Survival Forest (RSF), can provide superior prognostic performance for elderly sepsis patients compared to traditional methods, with C-index values of 0.731, outperforming SOFA, Simplified Acute Physiology Score II (SAPS II) and Acute Physiology Score III (APS III) scoring systems (Zhang et al., 2022). Leptin, a hormone predominantly secreted by adipocytes, is integral to regulating energy balance, metabolism, and immune responses (Obradovic et al., 2021). Beyond its role in energy homeostasis, leptin acts as a cytokine, influencing inflammation and immune cell activity (Abella et al., 2017). Elevated leptin levels have been linked to adverse outcomes in sepsis (Jacobsson et al., 2017), while low leptin levels may indicate immune deficiency (Birlutiu and Boicean, 2021). Given its significant role in sepsis, leptin is considered a potential biomarker for prognosis. Therefore, in this study, we aim to utilize ML techniques to develop mortality prediction and prognostic models for sepsis, incorporating leptin and other biomarkers to improve predictive accuracy.

## Methods

### Data processing

Transcriptomic data from human blood samples were obtained from the Gene Expression Omnibus (GEO) and ArrayExpress databases. Datasets containing samples from minors were excluded. Additionally, datasets that did not report mortality outcomes, lacked complete expression data, or included duplicated entries were also excluded. The expression data were obtained from public repositories and normalized according to the platform specifications using the limma package (Ritchie et al., 2015). Probes without corresponding gene symbols were removed. In cases where multiple probe sets mapped to the same gene symbol, the average expression value was used. Batch effects were corrected using the ComBat algorithm from the sva R package. Each dataset was treated as a batch, and expression matrices were merged on the intersection of shared genes after collapsing duplicate probes with limma. ComBat was applied in parametric empirical Bayes mode with mean–variance adjustment and batch indicators only, without additional covariates. The corrected expression matrix was used for downstream analyses (Leek et al., 2012; Zhang et al., 2020a). Only genes common across all included datasets were retained for model development and validation.

### Non-negative matrix factorization

In this study, NMF was employed for unsupervised clustering based on Leptin-related genes from the GeneCard database (Stelzer et al., 2016), with the aim of identifying molecular subtypes of sepsis. Gene expression data for key genes were extracted from the GeneCard database, and the data were transposed to meet the input format required for NMF analysis. NMF was performed using the NMF package, with ranks ranging from 2 to 10 to explore different potential clusters of sepsis patients. The Brunet method was applied for matrix factorization.

### Immune infiltration analysis

Single Sample Gene Set Enrichment Analysis (ssGSEA) (Subramanian et al., 2005) was used to assess immune cell infiltration in blood samples from septic patients. This method estimates the enrichment of immune cell types in individual samples based on predefined gene sets corresponding to various immune cell types. The analysis was performed using the GSVA package (Hänzelmann et al., 2013), which calculates the enrichment score for each immune cell type in each sample. The enrichment score represents the relative abundance of each immune cell type within the respective sample.

### Weighted gene co-expression network analysis

Using the WGCNA package (Langfelder and Horvath, 2008), potential Leptin-related gene modules associated with sepsis were identified. The data were preprocessed by filtering out genes with low variance, retaining only those with variance greater than the median. Missing values in the dataset were checked, and genes or samples with excessive missing data were removed to ensure the integrity of the analysis. Hierarchical clustering was performed on the samples to detect potential outliers, with samples outside a predefined cutoff being excluded. Gene modules were identified using hierarchical clustering based on the Topological Overlap Matrix (TOM). The optimal soft-thresholding power was determined, with a scale-free topology criterion set at 0.9. The TOM was then calculated, followed by hierarchical clustering of genes to construct a dendrogram, which was used for module detection. Modules were identified using the dynamic tree cutting method, with a minimum module size of 50 genes. The identified modules were correlated with clinical traits, such as survival data, using Pearson’s correlation. Significant modules were identified based on the correlation between gene module membership (MM) and gene trait significance (GS). Finally, the relationship between gene expression and clinical traits was assessed by visualizing MM and GS values through scatter plots. Genes most closely related to significant modules were retained for further analysis, including the identification of potential biomarkers associated with sepsis and mortality.

### Functional enrichment analysis

To explore the biological functions and pathways associated with the identified gene modules and genes, Gene Ontology (GO) (The Gene Ontology Consortium, 2019) and Kyoto Encyclopedia of Genes and Genomes (KEGG) (Kanehisa and Goto, 2000) functional enrichment analyses were conducted. GO analysis classifies genes into three main categories: biological process, molecular function, and cellular component. KEGG pathway enrichment analysis was then performed to identify the biological pathways involved.

### Differentially expressed genes

DEGs were identified by applying a fold change (FC) cutoff of 0.8 and a p-value threshold of 0.05. The limma package (Ritchie et al., 2015) was used to perform the differential expression analysis, with p-values adjusted for multiple testing using the Benjamini-Hochberg method.

### Prognostic classification models for 28-day mortality prediction

In this study, multiple machine learning algorithms were employed to construct prognostic classification models for predicting 28-day mortality in sepsis. These included combinations such as Lasso Regression + Random Forest (RF), Generalized Linear Model Boosting (GLMB) + RF, Lasso Regression + Gradient Boosting Machine (GBM), RF, GBM, and combinations of Stepwise Logistic Regression (SLR) with RF, among others. These models were trained on internal datasets and validated externally. Variable selection was performed using methods such as Lasso Regression and Elastic Net (EN). The performance of the models was evaluated using several metrics, including Receiver Operating Characteristic (ROC) curves, confusion matrix (CM), and decision curve analyses, to assess predictive accuracy, classification performance, and clinical utility. ROC curves assessed model discrimination, the CM evaluated classification capability, and DCA measured the potential clinical benefit for predicting 28-day mortality and informing decision-making.

### Time-to-event prognostic models for sepsis

In this study, various machine learning algorithms were employed to construct prognostic models for sepsis, including combinations of Random Survival Forest (RSF), Lasso Regression, Stepwise Cox Regression (StepCox), Gradient Boosting Machine (GBM), CoxBoost, Elastic Net (Enet), Ridge Regression, and others. The models were trained and evaluated using external validation datasets. To assess model performance, the concordance index (c-index) was calculated to evaluate the discriminatory power of the models in predicting patient outcomes. Additionally, time-dependent receiver operating characteristic (ROC) curves were employed to assess the predictive accuracy of the models over time, particularly for 28-day mortality prediction. The area under the curve (AUC) of the time-dependent ROC was computed to measure the ability of the models to classify patients correctly at various time points, providing a more comprehensive evaluation of their performance.

### Single-cell RNA sequencing data processing

Single-cell RNA sequencing data for this study were retrieved from the GEO database under accession number GSE167363 (Qiu et al., 2021), encompassing transcriptomic profiles of peripheral blood mononuclear cells (PBMCs) derived from healthy individuals and patients with gram-negative sepsis (both survivors and non-survivors). Raw count matrices generated via the 10X Genomics platform were imported and processed using the Seurat (Butler et al., 2018; Slovin et al., 2021) framework in R. To retain high-confidence single-cell profiles, we applied stringent quality control filters: cells with fewer than 1,000 total RNA molecules, fewer than 200 or more than 10,000 detected genes, over 20% mitochondrial gene expression, or over 20% ribosomal gene content were excluded. This filtering strategy helped eliminate potential artifacts such as dead cells, doublets, or empty droplets. The resulting curated dataset was stored for subsequent integrative and functional analyses. Following rigorous quality assessment, gene expression values were normalized using the LogNormalize approach in the Seurat package, wherein each gene count was scaled relative to the total expression per cell and log-transformed after multiplication by a scale factor of 10,000. To capture transcriptional heterogeneity, highly variable genes (HVGs) were selected using the variance-stabilizing transformation (vst) method, retaining the top 2,000 genes. These features were subsequently standardized to zero mean and unit variance prior to dimensionality reduction. Principal component analysis (PCA) was carried out on the HVGs, and inter-sample variation was mitigated using Harmony (Korsunsky et al., 2019), leveraging sample identity as the integration variable. The corrected principal components were then used to generate a t-distributed stochastic neighbor embedding (t-SNE) projection for visualization. Clustering was performed using a graph-based shared nearest neighbor (SNN) approach, with resolution tuning informed by hierarchical visualization through clustree. Final clustering was executed at a resolution of 1.2. Cell type annotation combined automated classification via SingleR (Zhao et al., 2020)—using reference transcriptomes of human immune cells—with manual refinement based on canonical marker gene expression patterns.

### Sample collection and ethics statement

A total of nine peripheral blood samples were obtained from Sichuan Provincial People’s Hospital, comprising three healthy controls, three sepsis patients who survived beyond 28 days, and three sepsis patients who died within 28 days of hospital admission. All samples were collected at the time of admission, prior to the initiation of any therapeutic intervention. Peripheral blood mononuclear cells (PBMCs) were isolated from freshly drawn blood using Ficoll-Paque density gradient centrifugation. The study protocol was approved by the Medical Ethics Committee of Sichuan Provincial People’s Hospital (Approval No. 2023-581), and written informed consent was obtained from all participants or their legal guardians.

### Real-time quantitative PCR

Total RNA was extracted from PBMCs using Trizol reagent (Invitrogen, Carlsbad, CA, USA). cDNA synthesis was performed using the ReverTra Ace qPCR RT Kit (TOYOBO, Osaka, Japan) according to the manufacturer’s instructions. For real-time quantitative PCR detecting system (RQ-PCR) analysis, TransStart Tip Green qPCR SuperMix (TransGen Biotech, Beijing, China) was used. Gene expression was normalized to GAPDH, and relative expression was calculated using the ΔΔCt method. The primers are listed in **Supplementary Table 1**.

### Western blot

PBMCs were lysed in RIPA buffer (Beyotime Biotechnology, Shanghai, China) containing 1 mM PMSF. Protein concentrations were measured using a BCA assay kit (Beyotime Biotechnology, Shanghai, China). Equal protein aliquots (30–50 μg) were separated by 8–12% SDS-PAGE and transferred to PVDF membranes (Servicebio, Wuhan, China). The membranes were blocked with 5% non-fat milk in TBST for 1 hour at room temperature, followed by overnight incubation at 4 °C with primary antibodies: PILRA (1:1,000), TFRC (1:1,000), and GAPDH (1:3,000) for loading control. After washing with TBST three times, membranes were incubated with HRP-conjugated goat anti-rabbit or anti-mouse secondary antibodies (1:3,000; Servicebio, Wuhan, China) for 1 hour at room temperature. Protein bands were visualized using ECL reagent (Servicebio) and analyzed with ImageJ software. GAPDH was used as a loading control.

### Statistical analysis

The analysis in this study was performed using R software (version 4.3.1). Spearman’s rank correlation coefficient was used to evaluate correlations between variables. To assess survival differences among groups, Kaplan-Meier survival curves were generated and compared using the log-rank test. The optimal threshold for survival analysis was determined using the surv_cutpoint function, which identifies the cutoff point that maximizes the survival differences. For statistical comparisons, an independent t-test or Mann–Whitney U test was applied for two-group analyses, depending on data distribution, while one-way ANOVA or Kruskal–Wallis test was employed for comparisons among multiple groups. A P-value of less than 0.05 (two-sided) was considered statistically significant.

## Results

### NMF based clustering reveals immune infiltration and clinical characteristics in septic patients

**Figure 1:**
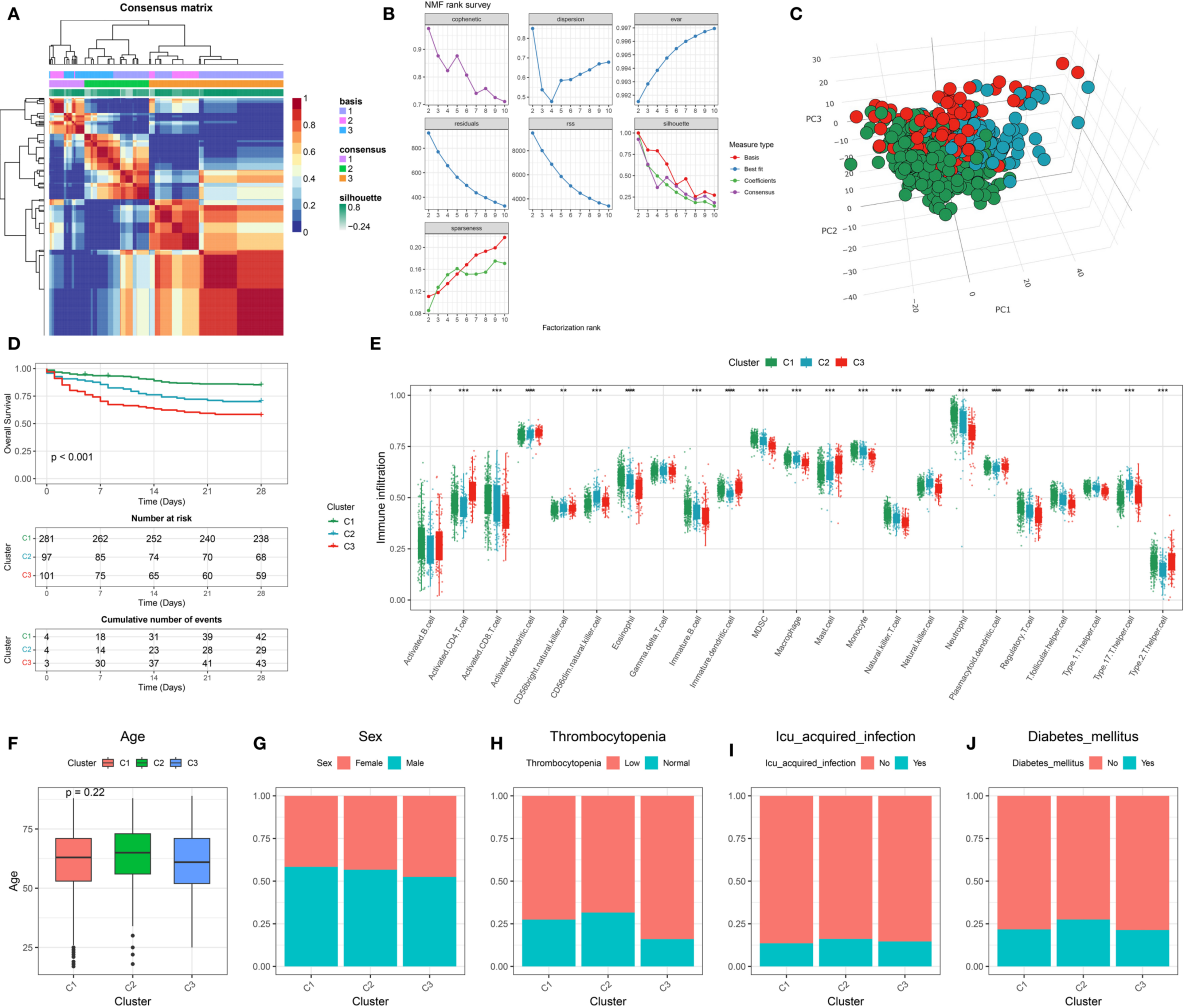
Identification and characterization of leptin-related molecular subtypes in sepsis. **(A)** Consensus clustering heatmap based on leptin-related gene expression profiles, identifying three stable molecular subtypes (C1, C2, C3). **(B)** Determination of optimal cluster number using non-negative matrix factorization (NMF) with multiple evaluation metrics, including cophenetic, dispersion, and silhouette scores. **(C)** Three-dimensional principal component analysis (3D PCA) showing clear separation among the three identified clusters. **(D)** Kaplan–Meier survival curves indicating significant differences in 28-day survival among the three clusters. **(E)** Boxplot showing expression levels of leptin-related genes across the three clusters; differences suggest subtype-specific molecular features. **(F)** Comparison of age distribution across clusters, with no significant difference observed. **(G–J)** Distribution of clinical features across clusters, including **(G)** sex, **(H)** thrombocytopenia status, **(I)** ICU-acquired infection, and **(J)** presence of diabetes mellitus, indicating potential clinical relevance of molecular subtypes.

The basic information of the datasets used in this study is provided in **Supplementary Table 2**. The code used in this study is provided in Supplementary Data Sheet S1. Genes associated with Leptin were retrieved from the GeneCards database (**Supplementary Table 3**). Using these genes, NMF was performed on sequencing data from GSE65682. As shown in **Figure 1A**, the consensus matrix divided the samples into three distinct clusters. To determine the optimal number of clusters, an NMF rank survey was conducted (**Figure 1B**). Various evaluation metrics, including cophenetic, dispersion, evar, residuals, RSS (Residual Sum of Squares), silhouette, and sparseness, were assessed across factorization ranks from 2 to 10. The NMF rank survey revealed that three clusters provided the best fit according to multiple metrics, indicating the clearest separation between the clusters. PCA further supported the distinct separation of the three clusters (**Figure 1C**). Prognostic analysis showed significant differences among the clusters, with Cluster 1 exhibiting the best prognosis, while Cluster 3 had the worst outcome (**Figure 1D**). Furthermore, immune infiltration differences between the clusters were compared. With the exception of Gamma.delta. T.cells, significant statistical differences in immune cell infiltration were observed between the three clusters (**Figure 1E**). In the analysis of clinical characteristics in relation to the clusters, several clinical variables were evaluated for their distribution across the clusters (**Figures 1F-J**). Age did not differ significantly between clusters, with similar age distributions observed across the three groups. The gender distribution was balanced across the clusters, with no significant differences in the male-to-female ratio within each cluster. For thrombocytopenia, there were no notable differences across the three groups, with both low and normal platelet levels present in all clusters. The proportion of patients with ICU-acquired infections was similar across the clusters, with no significant variation. Additionally, the prevalence of diabetes mellitus was evenly distributed across all clusters, showing no significant differences.

### WGCNA

**Figure 2:**
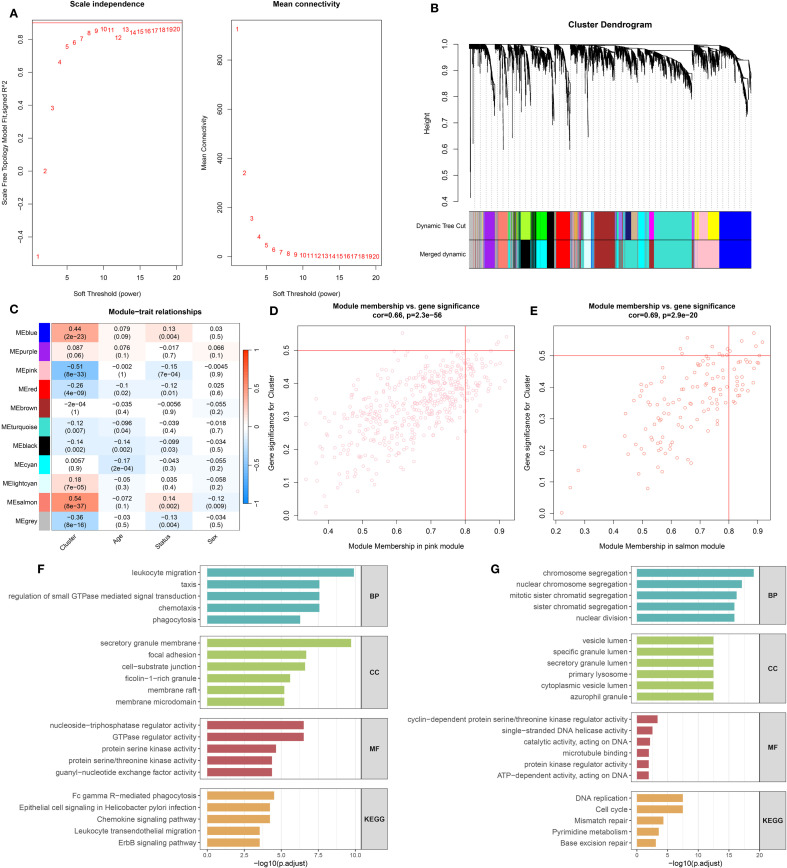
Co-expression network construction and enrichment analysis of leptin-related genes. **(A)** Determination of the soft-thresholding power (β) to achieve scale-free network topology, based on analyses of scale independence and mean connectivity. **(B)** Gene clustering dendrogram with corresponding module color assignments generated through WGCNA. **(C)** Correlation heatmap displaying associations between gene modules and clinical traits. **(D)** Relationship between module membership and gene significance in the pink module. **(E)** Relationship between module membership and gene significance in the salmon module. **(F)** Gene Ontology (GO) and KEGG pathway enrichment analysis results for genes in the pink module. **(G)** GO and KEGG enrichment analysis for genes in the salmon module.

Prior to performing WGCNA, the expression matrix derived from the GSE65682 dataset was confirmed to be of high quality, with no apparent outliers or missing values. A soft-thresholding power of β = 9 was selected, as it was the minimal value at which the scale-free topology model began to stabilize, as illustrated in **Figure 2A**. This threshold was subsequently used to generate the TOM and conduct preliminary module detection. Modules displaying similar gene expression profiles were further combined based on their eigengenes, yielding seven distinct gene modules (**Figure 2B**). Among them, the pink and salmon modules demonstrated particularly strong correlations, as shown in **Figure 2C**. Further analysis revealed significant associations between MM and GS within these modules (**Figures 2D, E**).

### Functional enrichment analysis of pink and salmon modules

Functional enrichment analysis revealed distinct biological roles for genes in the pink and salmon modules (**Figures 2F, G**). Genes in the pink module were mainly involved in immune-related processes, including leukocyte migration, chemotaxis, and phagocytosis. They were enriched in components such as secretory granule membranes and focal adhesions, and showed functions related to GTPase regulation and kinase activity. KEGG pathways indicated involvement in phagocytosis, chemokine signaling, and immune cell migration. In contrast, genes in the salmon module were associated with cell cycle–related processes like chromosome segregation and nuclear division. These genes were enriched in vesicle lumen and lysosome components, with functions including DNA helicase activity and microtubule binding. KEGG enrichment highlighted pathways such as DNA replication, cell cycle, and mismatch repair, suggesting roles in cell proliferation and genomic stability.

### Identification and functional analysis of hub genes

**Figure 3:**
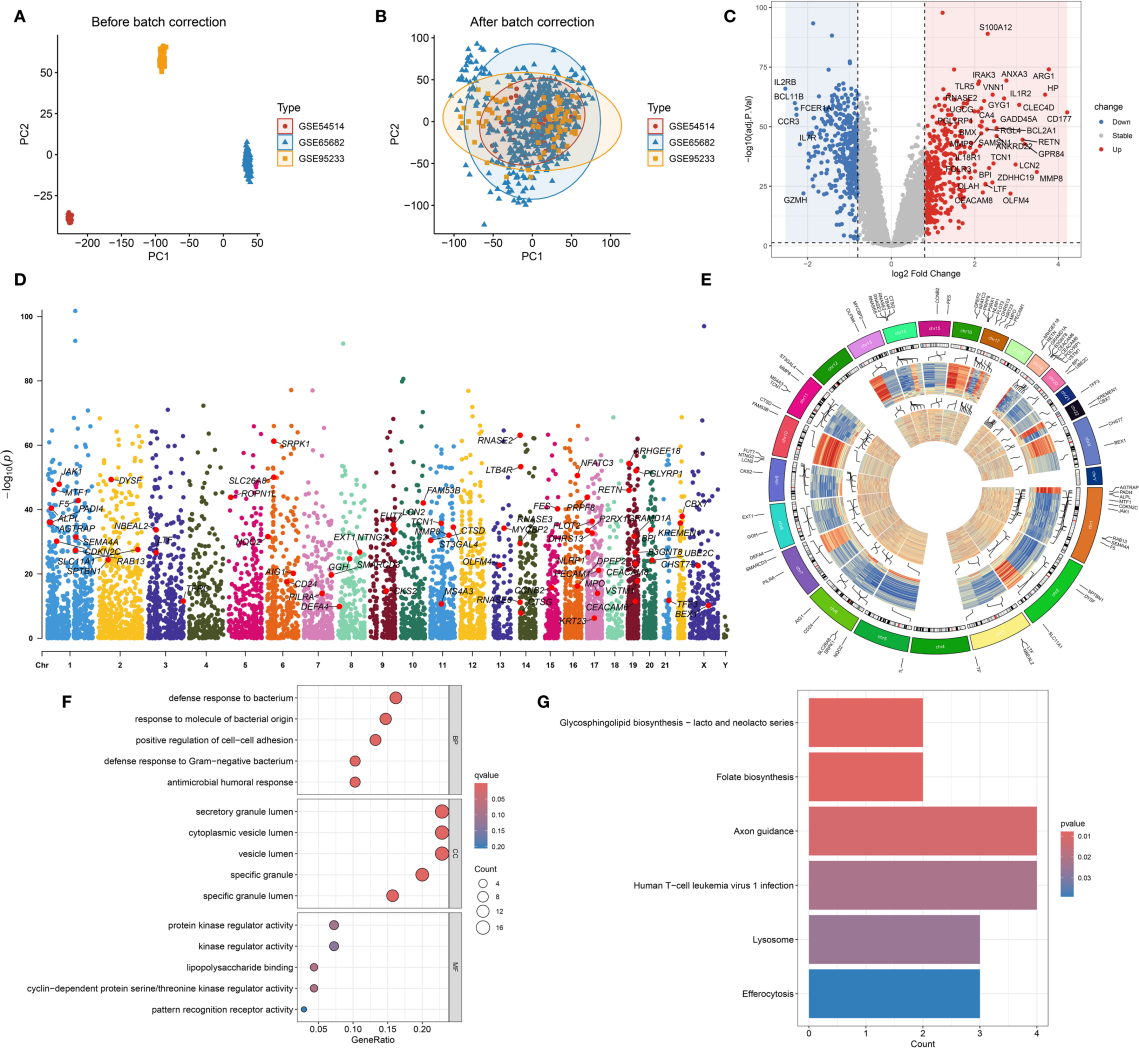
Batch effect correction, differential expression, and functional annotation of hub genes. **(A)** Principal component analysis (PCA) plot illustrating gene expression distribution prior to batch effect correction. **(B)** PCA plot after batch effect adjustment, showing improved sample clustering. **(C)** Volcano plot displaying differentially expressed genes (DEGs) between sepsis and healthy control groups. **(D)** Genomic chromosomal distribution of the 70 identified hub genes. **(E)** Manhattan plot representing the positional distribution and significance of the 70 hub genes. **(F)** Gene Ontology (GO) enrichment analysis of the 70 hub genes. **(G)** KEGG pathway enrichment analysis of the 70 hub genes.

To reduce technical variation among datasets, the ComBat algorithm was employed for batch effect correction. As shown in **Figure 3A**, prior to adjustment, samples from GSE54514, GSE65682, and GSE95233 clustered separately in the PCA plot, indicating substantial batch effects. After correction (**Figure 3B**), the datasets were well integrated, with samples from different cohorts intermixed, confirming effective removal of batch effects. Following integration, a total of 745 DEGs were identified based on predefined criteria (**Figure 3C**). These DEGs were intersected with gene sets from the pink and salmon modules derived from WGCNA, resulting in 70 overlapping hub genes (**Supplementary Table 4**). Chromosomal mapping showed that these hub genes were distributed across most chromosomes, excluding chromosomes 4, 12, 18, and the Y chromosome (**Figures 3D, E**). Functional enrichment analysis revealed that these hub genes are primarily involved in immune-related biological processes such as bacterial defense, cell–cell adhesion, and response to Gram-negative bacteria (**Figure 3F**). GO analysis also highlighted their localization to secretory granule lumens and involvement in kinase regulation and lipopolysaccharide binding. KEGG pathway analysis (**Figure 3G**) showed enrichment in pathways including axon guidance, lysosome, efferocytosis, and folate biosynthesis, suggesting potential roles in immune modulation and cellular clearance mechanisms in sepsis.

### Construction and validation of a machine learning–based prognostic classification model for 28-day mortality in sepsis

**Figure 4:**
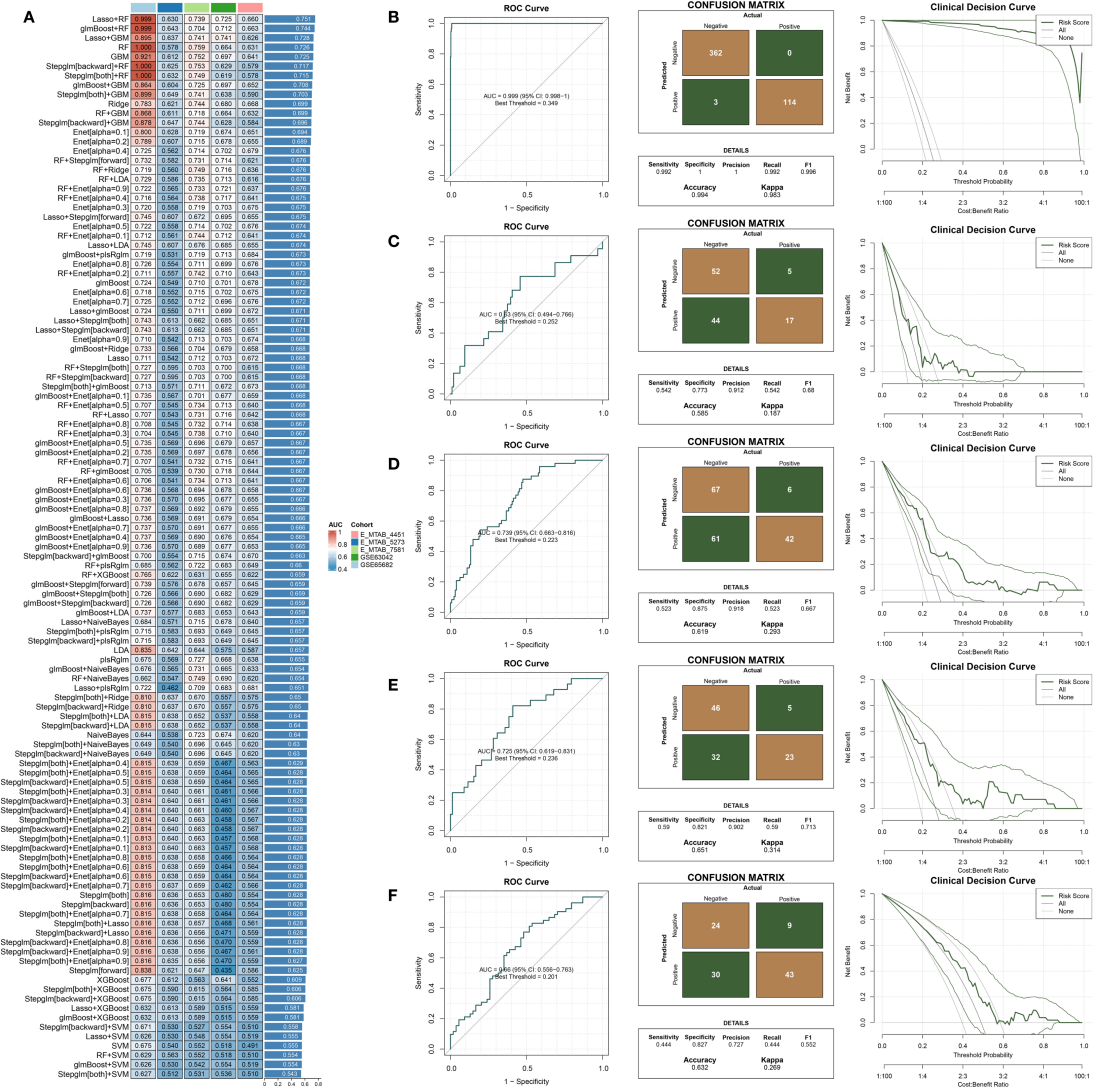
Construction and external validation of a diagnostic model for predicting 28-day mortality in sepsis. **(A)** AUC values of diagnostic models developed using different machine learning algorithms. **(B–F)** Combined presentation of ROC curves, confusion matrices, and decision curve analysis (DCA) for external validation across five independent datasets: **(B)** GSE65682, **(C)** E-MTAB-5273, **(D)** E-MTAB-7581, **(E)** GSE63042, and **(F)** E-MTAB-4451.

A variety of machine learning algorithms were utilized to construct a prognostic classification model aimed at predicting 28-day mortality in sepsis. The GSE65682 dataset was designated as the training cohort, while E-MTAB-4451, E-MTAB-5273, E-MTAB-7581, and GSE63042 served as independent validation cohorts. In total, 113 models—including both individual and ensemble algorithm strategies—were assessed, and their respective AUC values are presented in **Figure 4A**. Among these, the Lasso combined with Random Forest (RF) approach achieved the highest mean AUC across datasets and was therefore selected to construct the final predictive model (**Supplementary Table 5**) and compute the corresponding risk scores. The performance of the established risk score model was then evaluated in both the training and external validation sets using receiver operating characteristic (ROC) curves, confusion matrices, and clinical decision curve analysis (DCA) (**Figures 4B–F**). In the training set (GSE65682, **Figure 4B**), the model exhibited strong discriminatory capacity with an AUC of 0.94, along with high sensitivity, specificity, and net clinical benefit. In external datasets including E-MTAB-5273 (**Figure 4C**), E-MTAB-7581 (**Figure 4D**), GSE63042 (**Figure 4E**), and E-MTAB-4451 (**Figure 4F**), the model retained stable and reliable performance, with AUC values ranging from 0.74 to 0.83. The confusion matrices demonstrated balanced classification accuracy, while the DCA curves suggested superior clinical utility of the model compared to conventional reference strategies across a wide range of threshold probabilities. Besides, ROC curve analysis in the GSE65682 cohort demonstrated that the proposed model outperformed traditional clinical variables in predictive performance (**Supplementary Figure 1A**).

### Development and evaluation of a time-to-event prognostic model for sepsis

**Figure 5:**
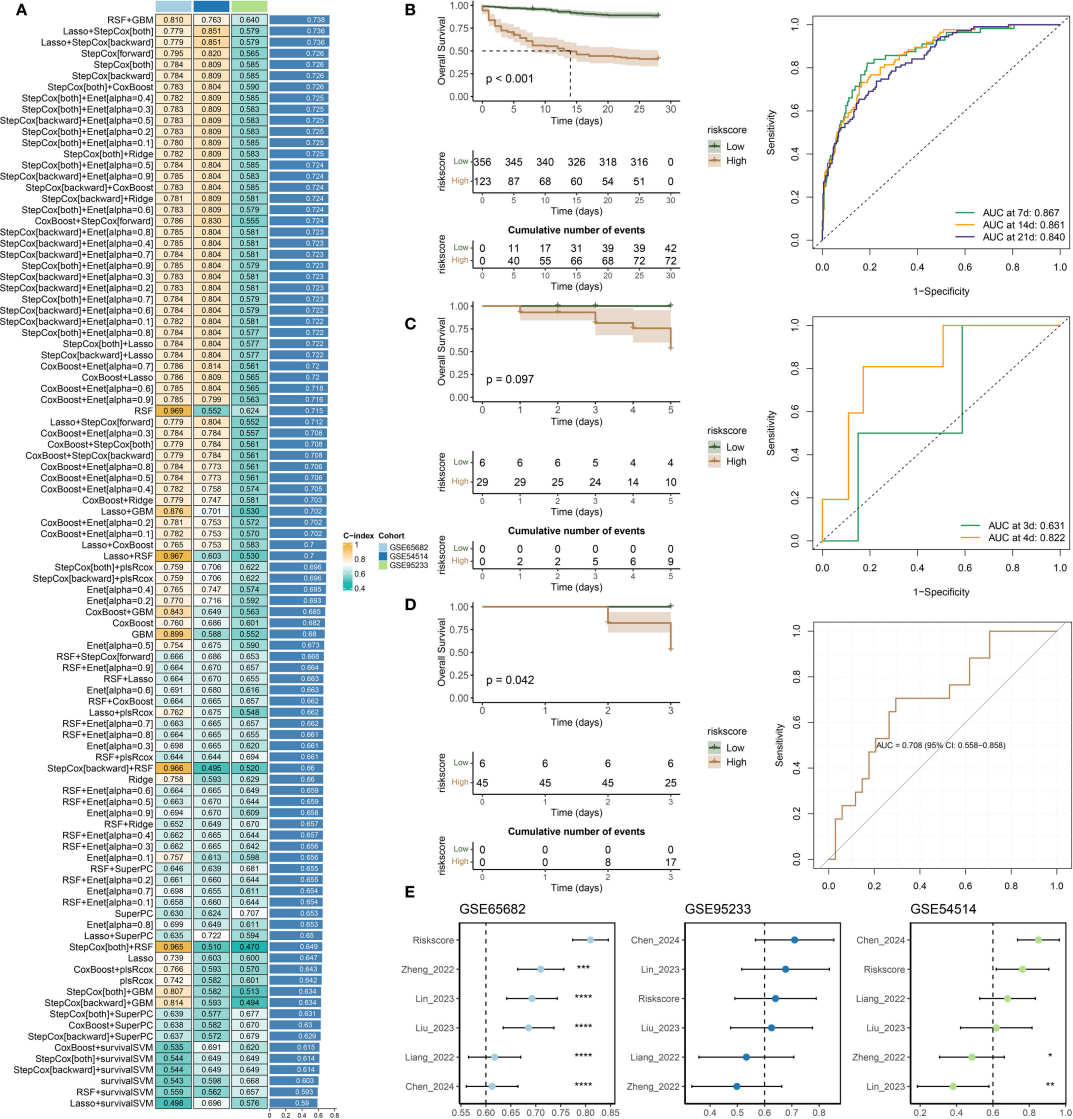
Development and validation of a prognostic model for sepsis. **(A)** Concordance index (C-index) values of prognostic models constructed using various machine learning algorithms. **(B–D)** Kaplan–Meier survival analysis and time-dependent ROC curves evaluating model performance in the following datasets: **(B)** GSE65682, **(C)** GSE54514, and **(D)** GSE95233. **(E)** Comparative analysis of the proposed model and previously published prognostic signatures, based on C-index values across all three datasets.

Multiple machine learning algorithms were applied to develop a prognostic model for sepsis patients. The GSE65682 dataset was used as the training cohort, while GSE54514 and GSE95233 served as external validation sets. A total of 98 models, constructed using either single algorithms or algorithmic combinations, were evaluated based on their concordance index (C-index) across datasets (**Figure 4A**). Among them, the RSF + GBM combination achieved the highest average C-index and was selected to build the final prognostic model (**Supplementary Table 5**) and compute individual risk scores (**Figure 5A**). As shown in **Figures 5B, C**, patients classified into the high-risk group had significantly worse survival outcomes compared to those in the low-risk group, with the exception of the GSE54514 dataset (**Figure 5D**). Furthermore, time-dependent ROC curves demonstrated that the risk score maintained strong prognostic accuracy across multiple time points. Finally, the model’s predictive performance was benchmarked against previously published prognostic models (Liang et al., 2022; Zheng et al., 2022; Chen et al., 2023; Lin et al., 2023; Liu et al., 2023; Chen et al., 2024) (**Figure 5E**). In the GSE65682 dataset, the proposed model outperformed all others, and in the remaining two cohorts, it consistently ranked among the top-performing models. Besides, C-index comparisons at 7, 14, and 28 days consistently demonstrated that the prognostic model achieved superior discriminative ability compared with conventional clinical variables (**Supplementary Figures 1B**). Moreover, both univariate and multivariate Cox regression analyses confirmed that the model-derived risk score was an independent prognostic factor for sepsis outcomes (**Supplementary Figures 1C, D**). Among them, five features—TFRC, PILRA, DEFA4, KRT23, and BEX1—are shared between the diagnostic and prognostic models.

### Single-cell transcriptomic profiling reveals cellular heterogeneity and gene expression signatures in sepsis

**Figure 6:**
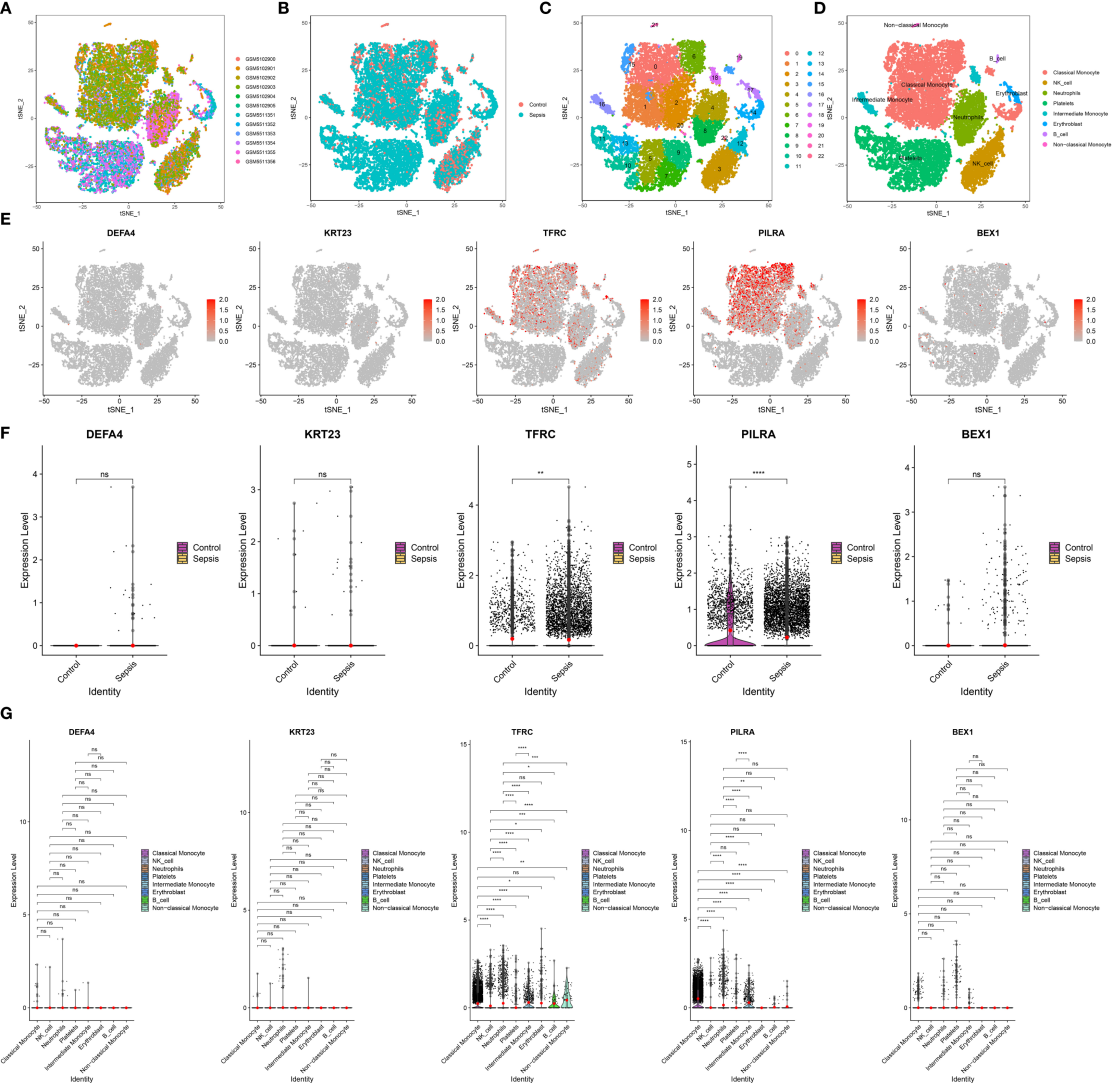
Single-cell transcriptomic analysis of key gene expression in sepsis. **(A)** t-SNE visualization of all 12 samples, demonstrating overall cell distribution. **(B)** t-SNE plots comparing cell distributions between control and sepsis groups. **(C)** Identification of 23 distinct cell clusters based on transcriptomic profiles. **(D)** Annotation of eight major cell types across all samples. **(E)** Feature plots showing the expression patterns of TFRC, PILRA, DEFA4, KRT23, and BEX1 within the eight annotated cell types. **(F)** Group-wise comparison of the five hub genes between sepsis and control samples. **(G)** Differential expression of TFRC, PILRA, DEFA4, KRT23, and BEX1 across the eight immune cell types.

After rigorous quality filtering to remove doublets, apoptotic cells, and empty droplets, high-confidence single-cell transcriptomic data were obtained (**Supplementary Figure 2A**). To correct sample-derived variability, the Harmony algorithm was applied, resulting in improved integration of cells across conditions (**Figure 6A**), with separate visualizations for the sepsis and control groups provided in **Figure 6B**. Clustering based on Harmony-corrected principal components (PC) (with PC 13 selected; **Supplementary Figures 2B, C**) identified 23 transcriptionally distinct clusters (**Figure 6C**), which were annotated using the SingleR package and grouped into six major immune cell types: Monocyte, NK_cell, Neutrophils, Platelets, Erythroblast, and B_cell (**Supplementary Figure 2D**). Within the monocyte lineage, three subsets were identified (**Supplementary Figure 2E**): classical, intermediate, and non-classical (Wong et al., 2011; Thomas et al., 2017; Li et al., 2024). In total, eight immune cell populations were delineated, including Monocytes (subdivided into classical, intermediate, and non-classical subsets), NK cells, Neutrophils, Platelets, Erythroblasts, and B cells (**Figure 6D**). Among the five candidate genes (TFRC, PILRA, DEFA4, KRT23, and BEX1), TFRC and PILRA displayed significantly higher expression in monocyte subsets compared with other immune cell types, whereas DEFA4, KRT23, and BEX1 showed minimal or negligible expression across all populations. Feature plots in **Figure 6E** confirmed their cell-type-specific expression patterns, and both TFRC and PILRA were differentially expressed between the sepsis and control groups (**Figure 6F**). Notably, in the sepsis group, TFRC and PILRA also exhibited significant variation in expression across the eight immune cell types, whereas no such differences were observed for the remaining genes (**Figure 6G**).

### Expression profiles of TFRC and PILRA in healthy controls and sepsis patients

**Figure 7:**
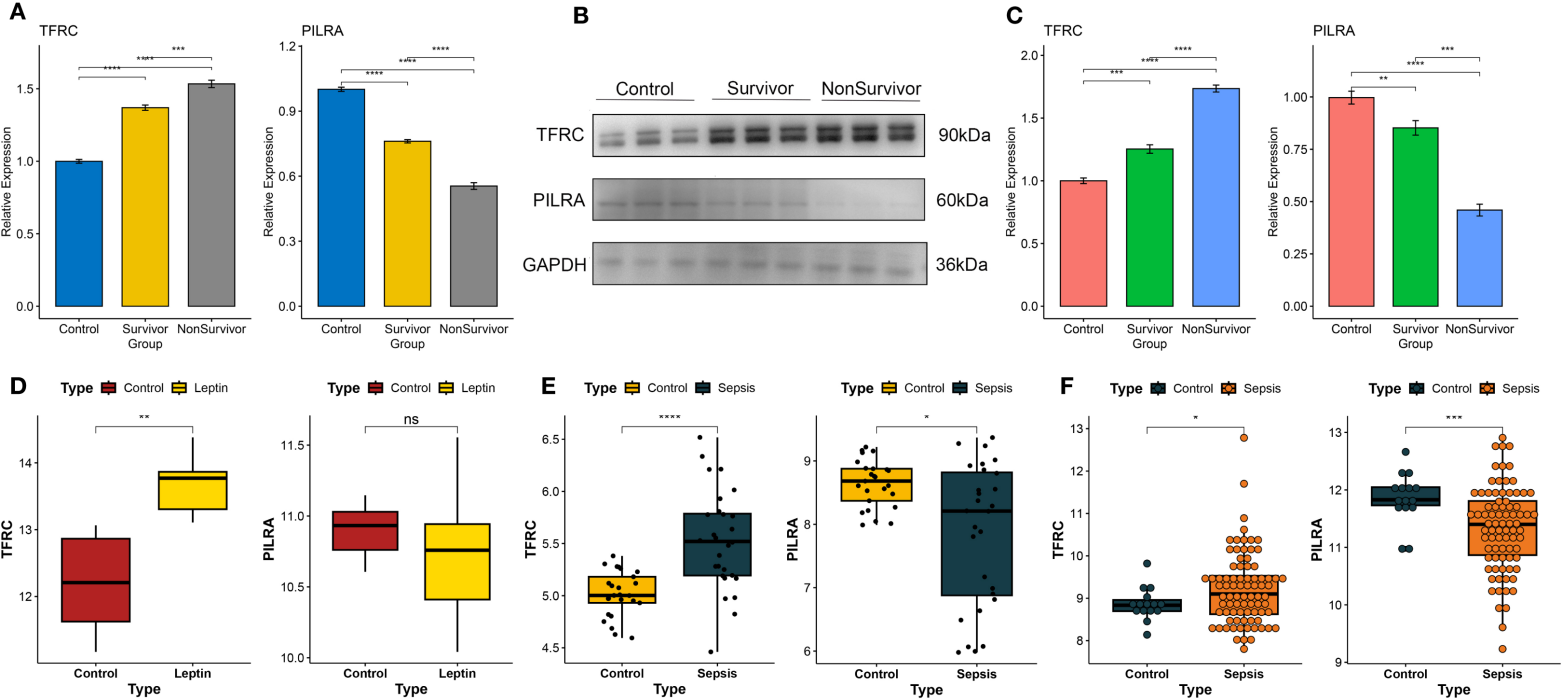
Experimental validation of TFRC and PILRA expression in human PBMCs and independent datasets. **(A)** Quantitative PCR (qPCR) analysis of TFRC and PILRA expression in peripheral blood mononuclear cells (PBMCs) from healthy controls, sepsis survivors, and non-survivors. **(B, C)** Western blot (WB) analysis of TFRC and PILRA protein expression in PBMCs across the same three groups. **(D)** Validation of TFRC and PILRA expression changes in T cells following leptin antibody treatment using the GSE154401 dataset. **(E)** Validation of TFRC and PILRA expression between sepsis patients and controls in the GSE57065 cohort. **(F)** Validation of TFRC and PILRA expression between sepsis patients and controls in the GSE131761 cohort. P < 0.05 was considered statistically significant and is indicated by *.

Validation in PBMCs from healthy controls, sepsis survivors, and non-survivors confirmed that TFRC expression progressively increased, whereas PILRA decreased across these groups (qPCR, **Figure 7A**; WB, **Figures 7B, C**). Analysis of the GSE154401 dataset showed that leptin antibody treatment of T cells significantly elevated TFRC, while PILRA exhibited a non-significant reduction (**Figure 7D**). Consistently, in GSE57065 and GSE131761, TFRC was upregulated and PILRA was downregulated in sepsis compared with controls (**Figures 7E, F**).

## Discussion

In this study, leptin-associated genes were identified through the integration of multiple computational approaches, including WGCNA, differential expression analysis, and NMF. Based on these candidate genes, two predictive models were developed: a prognostic model to estimate patient survival outcomes, and a diagnostic model specifically designed to predict 28-day mortality in sepsis. Both models demonstrated favorable predictive performance across internal and external validation cohorts. Among the genes incorporated into model development, TFRC and PILRA were consistently shared by both models and were selected for further investigation. Single-cell RNA sequencing data revealed their distinct expression profiles within specific immune cell populations. In addition, validation using patient-derived peripheral blood samples confirmed that TFRC and PILRA were significantly differentially expressed between septic individuals and healthy controls. These results support their potential utility as molecular indicators for disease classification and risk assessment in sepsis.

In recent years, numerous machine learning–based diagnostic models for sepsis have been proposed, many achieving high AUC values in training cohorts but showing limited generalizability in external datasets. For instance, Zhang et al. combined WGCNA and multiple classification algorithms to develop a diagnostic model based on 22 core genes (Zhang et al., 2025). While the model achieved an AUC of 0.999 in the training dataset, its performance dropped to 0.763 in the external validation cohort, reflecting potential overfitting and sensitivity to sample heterogeneity. Moor et al. developed a deep learning model trained on electronic health records from five countries, achieving an average AUC of 0.84 across multiple institutions and enabling sepsis detection approximately 3.7 hours earlier than clinical diagnosis (Moor et al., 2023). However, the model relied heavily on structured EHR data and lacked mechanistic biological insights. By contrast, our LASSO + RF-based diagnostic model, constructed using 14 transcriptomic features, achieved an AUC of 0.999 in the training dataset and maintained reasonable performance in four independent validation cohorts (AUCs ranging from 0.630 to 0.739). Unlike models relying solely on clinical variables or lacking external validation, our model is grounded in molecular expression profiles, offering both biological interpretability and cross-platform robustness, which enhances its potential for early, accurate identification of sepsis in diverse patient populations.

In addition to the representative prognostic models already compared in this study, several other recent models deserve attention. (Sweeney et al., 2018). developed three transcriptome-based mortality risk scores using 12 datasets, achieving AUCs between 0.77 and 0.89 in five external validation cohorts. Their strength lies in extensive cross-dataset validation, though the models employed relatively simple linear approaches and lacked nonlinear interaction modeling. (Chenoweth et al., 2024). identified eight prognostic genes through meta-analysis across international cohorts. Their logistic regression–based model yielded an AUC of 0.812, outperforming qSOFA in predicting 28-day mortality, yet still limited in modeling complex gene-gene interactions. In comparison, our prognostic model integrates 12 genes and leverages a RSF + GBM ensemble learning strategy to better capture nonlinear relationships. This combination yielded high predictive performance in both training and external datasets. Overall, compared to existing models, ours features broader pathway integration and more powerful algorithmic modeling, offering enhanced risk stratification capabilities in the context of sepsis’s multifactorial pathophysiology.

TFRC (transferrin receptor, CD71) is a transmembrane glycoprotein that mediates cellular iron uptake and is widely expressed in proliferating immune cells (Jabara et al., 2016). By binding to transferrin, TFRC facilitates the internalization of iron ions, thereby supporting lymphocyte proliferation and immune function (Jabara et al., 2016). Mutations in the TFRC gene can lead to combined immunodeficiency with T and B cell defects and hypogammaglobulinemia, underscoring its essential role in immune competence (Jabara et al., 2016). In contrast, PILRA (paired immunoglobulin-like type 2 receptor alpha) is an inhibitory receptor expressed on myeloid cells. It contains an intracellular ITIM domain, which recruits phosphatases such as SHP-1 to deliver negative signals (Mousseau et al., 2000). Through this mechanism, PILRA downregulates the activation of monocyte/macrophage and NK cell populations, contributing to immune homeostasis and limiting excessive inflammation (Mousseau et al., 2000). In animal models, mice deficient in PILRA show enhanced production of pro-inflammatory cytokines such as IL-1β and IL-6 following inflammatory stimulation, resulting in exacerbated tissue injury. These findings suggest a negative regulatory role for PILRA in immune responses (Sun et al., 2014).

The immunopathology of sepsis is characterized by a disruption of immune homeostasis. Emerging evidence suggests that altered expression of TFRC and PILRA may be involved in this dysregulation. Proteomic analyses have shown that TFRC levels are significantly elevated in the peripheral blood of sepsis patients compared to healthy controls, and particularly higher among nonsurvivors (Li et al., 2022). These findings indicate a strong association between elevated TFRC and poor prognosis, highlighting its diagnostic and prognostic value in sepsis (Li et al., 2022). Moreover, mechanistic studies have revealed that TFRC may influence disease progression by regulating ferroptosis, a form of iron-dependent cell death, which contributes to tissue damage during sepsis (Wang and Bian, 2025). In comparison, research on PILRA in the context of sepsis is limited. However, transcriptomic clustering analyses have identified PILRA as a marker distinguishing immune subtypes of sepsis, suggesting that its expression may modulate the magnitude and trajectory of inflammatory responses (Zhang et al., 2020b). Taken together, TFRC and PILRA appear to mediate distinct but complementary immunometabolic processes—iron regulation and innate immune modulation—that may jointly contribute to sepsis pathogenesis.

Our single-cell RNA sequencing data, together with independently collected samples from sepsis patients and healthy controls, revealed that TFRC is upregulated, while PILRA is downregulated in PBMCs. This pattern is consistent with previous findings and supports their potential utility as sepsis biomarkers. Elevated TFRC likely reflects heightened immune cell proliferation and may correlate with disease severity (Li et al., 2022), whereas reduced PILRA suggests attenuation of inhibitory signaling and a possible hyperinflammatory state. Therapeutically, both molecules present as promising targets for immune modulation. Strategies aimed at limiting TFRC-mediated iron uptake—such as iron chelation or ferroptosis inhibition—may mitigate inflammation and tissue injury in sepsis (Wang and Bian, 2025). Meanwhile, modulation of PILRA signaling, via agonists or antagonists, could serve dual purposes: dampening early hyperinflammation or reversing late-phase immunosuppression. In conclusion, TFRC and PILRA, supported by both transcriptomic data and existing literature, show promise as immunobiological markers and therapeutic targets in sepsis and warrant further investigation.

Despite the promising results, several limitations should be acknowledged. First, all models were derived from publicly available transcriptomic datasets, without inclusion of an independent, prospectively collected cohort, which may limit generalizability to broader clinical populations. Although external validation was performed across multiple datasets, most lacked detailed clinical annotations, restricting the integration of routine variables such as SOFA scores or lactate that could enhance bedside applicability. Second, experimental validation was limited to PBMC samples from a small cohort, and although consistent patterns were observed across two independent datasets (GSE57065 and GSE131761), mechanistic experiments such as functional perturbations (e.g., gene knockdown or leptin stimulation assays) were not performed. Third, while scRNA-seq analysis enabled immune cell annotation and highlighted the transcriptional heterogeneity of monocytes, the resolution remains limited and does not provide spatial context. Finally, the diagnostic and prognostic models demonstrated some variability in predictive performance across datasets, likely reflecting batch effects, demographic differences, or technical inconsistencies. Future work should focus on prospective multi-center validation, integration of multi-omics and harmonized clinical data, and experimental studies to elucidate causal mechanisms and strengthen the translational potential of these findings.

## Conclusion

Machine learning approaches identified leptin-associated molecular subtypes and facilitated the development of prognostic models for sepsis. TFRC and PILRA were highlighted as potential biomarkers, supported by multi-level validation. These findings underscore the potential of leptin-related pathways as important correlates of immune dysregulation in sepsis, although further mechanistic studies are warranted to confirm causality.

## Data Availability

The datasets presented in this study can be found in online repositories. The names of the repository/repositories and accession number(s) can be found in the article/**Supplementary Material**.
