# Supplementary material for: Machine learning-based identification of leptin-associated biomarkers and prognostic prediction models in sepsis
Source: Front Cell Infect Microbiol. 2025 Sep 29;15:1630446. doi: 10.3389/fcimb.2025.1630446 (PMC12515905; doi:10.3389/fcimb.2025.1630446)
Supplement: Supplementary file 1 [file DataSheet1.pdf]

NMF:

```
# Load necessary packages
library(NMF)

# Set working directory
setwd("path_to_your_project_directory")    # Please modify this to your actual directory path

# Read data
rt <- read.table("data_file.txt", header = TRUE, sep = "\t", check.names = FALSE, row.names = 1)

# NMF analysis
# Using gene expression data for analysis
data <- t(rt)

# Run NMF analysis, specifying rank (number of clusters)
res <- nmf(data, rank = 2:10, method = "brunet", nrun = 10, seed = 123456)

# Plot cophenetic plot and save it as PDF
pdf(file = "cophenetic.pdf", width = 8, height = 7, onefile = FALSE)
plot(res)
dev.off()

# Output heatmap for all clusters
pdf(file = "heatmap.all.pdf", width = 15, height = 15, onefile = FALSE)
consensusmap(res,
             annRow = NA,
             annCol = NA,
             main = "Consensus matrix",
             info = FALSE)
dev.off()

# Output clustering results
clusterNum <- 3    # Select the number of clusters based on the chosen criteria
res <- nmf(data, rank = clusterNum, method = "brunet", nrun = 10, seed = 123456)
Cluster <- predict(res)
Cluster <- as.data.frame(Cluster)
Cluster$Cluster <- paste0("C", Cluster$Cluster)
clusterOut <- rbind(ID = colnames(Cluster), Cluster)
write.table(clusterOut, file = "cluster.txt", sep = "\t", quote = FALSE, col.names = FALSE)

# Output heatmap for specific clusters
pdf(file = "heatmap.pdf", width = 6, height = 6, onefile = FALSE)
consensusmap(res,
             annRow = NA,
```

```
                annCol = NA,
                main = "Consensus matrix",
                info = FALSE)
dev.off()
```

WGCNA:

```
setwd('your_working_directory_here')    # Set your working directory
options(stringsAsFactors = FALSE)

# Read the expression matrix file
expression_data = read.csv('your_expression_data_file.txt', sep = '\t', row.names = 1)
dim(expression_data)

# Filter genes with variance greater than the median
gene_variance = apply(expression_data, 1, var)
filtered_data = expression_data[which(gene_variance > quantile(gene_variance, probs = 0.5)), ]
dim(filtered_data)

# Save the filtered data
write.table(filtered_data, file = "filtered_gene_input.txt", sep = '\t', quote = FALSE, row.names =
TRUE)

library("WGCNA")    # Load WGCNA package

# Set the working directory
data = read.table("filtered_gene_input.txt", sep = "\t", header = TRUE, check.names = FALSE,
row.names = 1)

# Remove normal samples (if applicable)
# Uncomment the following lines if needed
# group = sapply(strsplit(colnames(data), "\\-"), "[", 4)
# group = sapply(strsplit(group, ""), "[", 1)
# group = gsub("2", "1", group)
# data = data[, group == 0]
datExpr0 = t(data)

### Check for missing values
gsg = goodSamplesGenes(datExpr0, verbose = 3)
if (!gsg$allOK) {
  # Print removed genes and samples (optional)
  if (sum(!gsg$goodGenes) > 0)
    print(paste("Removing genes:", paste(names(datExpr0)[!gsg$goodGenes], collapse = ", ")))
  if (sum(!gsg$goodSamples) > 0)
    print(paste("Removing samples:", paste(rownames(datExpr0)[!gsg$goodSamples], collapse =
```

```
", ")))

    # Remove offending genes and samples
    datExpr0 = datExpr0[gsg$goodSamples, gsg$goodGenes]
}

### Sample clustering
sampleTree = hclust(dist(datExpr0), method = "average")
pdf(file = "sample_cluster.pdf", width = 12, height = 9)
par(cex = 0.6)
par(mar = c(0, 4, 2, 0))
plot(sampleTree, main = "Sample clustering to detect outliers", sub = "", xlab = "", cex.lab = 1.5,
cex.axis = 1.5, cex.main = 2)
abline(h = 90, col = "red")    # Cutting line
dev.off()

### Remove samples below the cutting line
clust = cutreeStatic(sampleTree, cutHeight = 90, minSize = 10)
table(clust)
keepSamples = (clust == 1)
datExpr0 = datExpr0[keepSamples, ]

### Read mRNAsi data
traitData = read.table("Trait.txt", row.names = 1, header = TRUE, comment.char = "",
check.names = FALSE)
fpkmSamples = rownames(datExpr0)
traitSamples = rownames(traitData)
sameSample = intersect(fpkmSamples, traitSamples)
datExpr0 = datExpr0[sameSample, ]
datTraits = traitData[sameSample, ]

### Sample clustering
sampleTree2 = hclust(dist(datExpr0), method = "average")
traitColors = numbers2colors(datTraits, signed = FALSE)
pdf(file = "sample_heatmap.pdf", width = 20, height = 8)
plotDendroAndColors(sampleTree2, traitColors, groupLabels = names(datTraits), main = "Sample
dendrogram and trait heatmap")
dev.off()

### Power value scatter plot
enableWGCNAThreads()      # Enable multi-threading
powers = c(1:20)              # Power range 1 to 20
sft = pickSoftThreshold(datExpr0, powerVector = powers, verbose = 5)
pdf(file = "scale_independence.pdf", width = 10, height = 8)
```

```
par(mfrow = c(1, 2))
cex1 = 0.9
# Fit index vs. power scatter plot
plot(sft$fitIndices[, 1], -sign(sft$fitIndices[, 3]) * sft$fitIndices[, 2], xlab = "Soft Threshold (power)", ylab = "Scale Free Topology Model Fit, signed R^2", type = "n", main = paste("Scale independence"))
text(sft$fitIndices[, 1], -sign(sft$fitIndices[, 3]) * sft$fitIndices[, 2], labels = powers, cex = cex1, col = "red")
abline(h = 0.9, col = "red")
# Mean connectivity vs. power scatter plot
plot(sft$fitIndices[, 1], sft$fitIndices[, 5], xlab = "Soft Threshold (power)", ylab = "Mean Connectivity", type = "n", main = paste("Mean connectivity"))
text(sft$fitIndices[, 1], sft$fitIndices[, 5], labels = powers, cex = cex1, col = "red")
dev.off()

### Adjacency matrix transformation
softPower = sft$powerEstimate    # Optimal power value
adjacency = adjacency(datExpr0, power = softPower)

### TOM matrix
TOM = TOMsimilarity(adjacency)
dissTOM = 1 - TOM

### Gene clustering
geneTree = hclust(as.dist(dissTOM), method = "average")
pdf(file = "gene_clustering.pdf", width = 12, height = 9)
plot(geneTree, xlab = "", sub = "", main = "Gene clustering on TOM-based dissimilarity", labels = FALSE, hang = 0.04)
dev.off()

### Dynamic module identification
minModuleSize = 50    # Minimum module gene size
dynamicMods = cutreeDynamic(dendro = geneTree, distM = dissTOM, deepSplit = 2, pamRespectsDendro = FALSE, minClusterSize = minModuleSize)
table(dynamicMods)
dynamicColors = labels2colors(dynamicMods)
table(dynamicColors)
pdf(file = "dynamic_tree.pdf", width = 8, height = 6)
plotDendroAndColors(geneTree, dynamicColors, "Dynamic Tree Cut", dendroLabels = FALSE, hang = 0.03, addGuide = TRUE, guideHang = 0.05, main = "Gene dendrogram and module colors")
dev.off()

### Similar module clustering
```

```
MEList = moduleEigengenes(datExpr0, colors = dynamicColors)
MEs = MEList$eigengenes
MEDiss = 1 - cor(MEs)
METree = hclust(as.dist(MEDiss), method = "average")
pdf(file = "clustering_module.pdf", width = 7, height = 5)
plot(METree, main = "Clustering of module eigengenes", xlab = "", sub = "")
MEDissThres = 0.3    # Dissimilarity threshold for merging
abline(h = MEDissThres, col = "red")
dev.off()

### Merge similar modules
merge = mergeCloseModules(datExpr0, dynamicColors, cutHeight = MEDissThres, verbose = 3)
mergedColors = merge$colors
mergedMEs = merge$newMEs
pdf(file = "merged_dynamic.pdf", width = 8, height = 6)
plotDendroAndColors(geneTree, cbind(dynamicColors, mergedColors), c("Dynamic Tree Cut", "Merged dynamic"), dendroLabels = FALSE, hang = 0.03, addGuide = TRUE, guideHang = 0.05)
dev.off()
moduleColors = mergedColors
table(moduleColors)
colorOrder = c("grey", standardColors(50))
moduleLabels = match(moduleColors, colorOrder) - 1
MEs = mergedMEs

### Module-trait relationships heatmap
nGenes = ncol(datExpr0)
nSamples = nrow(datExpr0)
moduleTraitCor = cor(MEs, datTraits, use = "p")
moduleTraitPvalue = corPvalueStudent(moduleTraitCor, nSamples)
pdf(file = "module_trait.pdf", width = 8, height = 7)
textMatrix = paste(signif(moduleTraitCor, 2), "\n(", signif(moduleTraitPvalue, 1), ")", sep = "")
dim(textMatrix) = dim(moduleTraitCor)
par(mar = c(5, 10, 3, 3))
labeledHeatmap(Matrix = moduleTraitCor, xLabels = names(datTraits), yLabels = names(MEs), ySymbols = names(MEs), colorLabels = FALSE, colors = blueWhiteRed(50), textMatrix = textMatrix, setStdMargins = FALSE, cex.text = 1, zlim = c(-1, 1), main = paste("Module-trait relationships"))
dev.off()

### Calculate MM and GS values
modNames = substring(names(MEs), 3)
geneModuleMembership = as.data.frame(cor(datExpr0, MEs, use = "p"))
MMPvalue = as.data.frame(corPvalueStudent(as.matrix(geneModuleMembership), nSamples))
names(geneModuleMembership) = paste("MM", modNames, sep = "")
```

```
names(MMPvalue) = paste("p.MM", modNames, sep = "")
traitNames = names(datTraits)
geneTraitSignificance = as.data.frame(cor(datExpr0, datTraits, use = "p"))
GSPvalue = as.data.frame(corPvalueStudent(as.matrix(geneTraitSignificance), nSamples))
names(geneTraitSignificance) = paste("GS.", traitNames, sep = "")
names(GSPvalue) = paste("p.GS.", traitNames, sep = "")

### Output GS_MM data
probes = colnames(datExpr0)
geneInfo0 = data.frame(probes = probes, moduleColor = moduleColors)
for (Tra in 1:ncol(geneTraitSignificance)) {
  oldNames = names(geneInfo0)
  geneInfo0 = data.frame(geneInfo0, geneTraitSignificance[, Tra], GSPvalue[, Tra])
  names(geneInfo0) = c(oldNames, names(geneTraitSignificance)[Tra], names(GSPvalue)[Tra])
}

for (mod in 1:ncol(geneModuleMembership)) {
  oldNames = names(geneInfo0)
  geneInfo0 = data.frame(geneInfo0, geneModuleMembership[, mod], MMPvalue[, mod])
  names(geneInfo0) = c(oldNames, names(geneModuleMembership)[mod],
names(MMPvalue)[mod])
}
geneOrder = order(geneInfo0$moduleColor)
geneInfo = geneInfo0[geneOrder, ]
write.table(geneInfo, file = "GS_MM.xls", sep = "\t", row.names = FALSE)
```

Analysis of differential gene expression:

```
# Load necessary packages
library(limma)
library(sva)

# Set working directory
setwd("your_directory_here")   # Set the correct working directory

# Read gene expression data
inputFile = "geneMatrix.txt"   # Expression data file
conFile = "control_samples.txt"   # Control samples file
treatFile = "treated_samples.txt"   # Experimental samples file
geoID = "Study_ID"   # GEO study ID

# Read input data and organize it
rt = read.table(inputFile, header = TRUE, sep = "\t", check.names = FALSE)
rt = as.matrix(rt)
rownames(rt) = rt[, 1]
```

```
exp = rt[, 2:ncol(rt)]
dimnames = list(rownames(exp), colnames(exp))
data = matrix(as.numeric(as.matrix(exp)), nrow = nrow(exp), dimnames = dimnames)
rt = avereps(data)

# If the data is not log2 transformed, log2 transform it
qx = as.numeric(quantile(rt, c(0, 0.25, 0.5, 0.75, 0.99, 1.0), na.rm = TRUE))
LogC = ((qx[5] > 100) || ((qx[6] - qx[1]) > 50 && qx[2] > 0))
if (LogC) {
   rt[rt < 0] = 0
   rt = log2(rt + 1)
}
data = normalizeBetweenArrays(rt)

# Read sample information files (control and experimental)
sample1 = read.table(conFile, header = FALSE, sep = "\t", check.names = FALSE)
sample2 = read.table(treatFile, header = FALSE, sep = "\t", check.names = FALSE)
sampleName1 = gsub("^ | $", "", as.vector(sample1[, 1]))
sampleName2 = gsub("^ | $", "", as.vector(sample2[, 1]))
conData = data[, sampleName1]
treatData = data[, sampleName2]
data = cbind(conData, treatData)
conNum = ncol(conData)
treatNum = ncol(treatData)

# Output normalized expression data
Type = c(rep("Control", conNum), rep("Treat", treatNum))
outData = rbind(id = paste0(colnames(data), "_", Type), data)
write.table(outData, file = paste0(geoID, ".normalize.txt"), sep = "\t", quote = FALSE, col.names
= FALSE)

# --- Batch effect correction ---

# Set working directory for batch correction
setwd("your_batch_correction_directory_here")

# Get the list of files to process
files = dir()
files = grep("normalize.txt$", files, value = TRUE)
geneList = list()

# Read gene information from each file and store in geneList
for (file in files) {
   if (file == "merge.preNorm.txt") { next }
```

```
    if (file == "merge.normalize.txt") { next }
    rt = read.table(file, header = TRUE, sep = "\t", check.names = FALSE)
    geneNames = as.vector(rt[, 1])
    uniqGene = unique(geneNames)
    header = unlist(strsplit(file, "\\.|\\-"))
    geneList[[header[1]]] = uniqGene
}

# Get intersection of genes
interGenes = Reduce(intersect, geneList)

# Data merge
allTab = data.frame()
batchType = c()
for (i in 1:length(files)) {
    inputFile = files[i]
    if (inputFile == "merge.preNorm.txt") { next }
    if (inputFile == "merge.normalize.txt") { next }
    header = unlist(strsplit(inputFile, "\\.|\\-"))
    rt = read.table(inputFile, header = TRUE, sep = "\t", check.names = FALSE)
    rt = as.matrix(rt)
    rownames(rt) = rt[, 1]
    exp = rt[, 2:ncol(rt)]
    dimnames = list(rownames(exp), colnames(exp))
    data = matrix(as.numeric(as.matrix(exp)), nrow = nrow(exp), dimnames = dimnames)
    rt = avereps(data)
    colnames(rt) = paste0(header[1], "_", colnames(rt))

    # Merge data
    if (i == 1) {
        allTab = rt[interGenes, ]
    } else {
        allTab = cbind(allTab, rt[interGenes, ])
    }
    batchType = c(batchType, rep(i, ncol(rt)))
}

# Output merged expression data
outTab = rbind(geneNames = colnames(allTab), allTab)
write.table(outTab, file = "merge.preNorm.txt", sep = "\t", quote = FALSE, col.names = FALSE)

# Perform batch effect correction and output corrected expression data
outTab = ComBat(allTab, batchType, par.prior = TRUE)
outTab = rbind(geneNames = colnames(outTab), outTab)
```

```
write.table(outTab, file = "merge.normalize.txt", sep = "\t", quote = FALSE, col.names = FALSE)
```

Machine learning:

```
# Set working path
work.path <- "your_work_directory_here"    # Set the correct working directory
setwd(work.path)

# Set other paths
code.path <- file.path(work.path, "Codes")
data.path <- file.path(work.path, "InputData")
res.path <- file.path(work.path, "Results")
fig.path <- file.path(work.path, "Figures")

# Create paths if they do not exist
if (!dir.exists(data.path)) dir.create(data.path)
if (!dir.exists(res.path)) dir.create(res.path)
if (!dir.exists(fig.path)) dir.create(fig.path)
if (!dir.exists(code.path)) dir.create(code.path)

# Load necessary packages
library(openxlsx)
library(seqinr)
library(plyr)
library(randomForestSRC)
library(glmnet)
library(plsRglm)
library(gbm)
library(caret)
library(mboost)
library(e1071)
library(BART)
library(MASS)
library(snowfall)
library(xgboost)
library(ComplexHeatmap)
library(RColorBrewer)
library(pROC)

# Load model training and evaluation scripts
source(file.path(code.path, "ML.R"))

# Choose final model type: "panML" for multi-algorithm models or "multiLogistic" for logistic
models
FinalModel <- c("panML", "multiLogistic")[2]
```

```
## Training Cohort --------------------------------------------------------
# Training set gene expression data (genes of interest)
Train_expr <- read.table(file.path(data.path, "Training_expr.txt"), header = TRUE, sep = "\t",
row.names = 1, check.names = FALSE, stringsAsFactors = FALSE)
# Training set class labels (binary variable)
Train_class <- read.table(file.path(data.path, "Training_class.txt"), header = TRUE, sep = "\t",
row.names = 1, check.names = FALSE, stringsAsFactors = FALSE)
# Get common samples between expression data and class labels
comsam <- intersect(rownames(Train_class), colnames(Train_expr))
Train_expr <- Train_expr[, comsam]
Train_class <- Train_class[comsam, , drop = FALSE]

## Validation Cohort ------------------------------------------------------
# Testing set gene expression data (genes of interest)
Test_expr <- read.table(file.path(data.path, "Testing_expr.txt"), header = TRUE, sep = "\t",
row.names = 1, check.names = FALSE, stringsAsFactors = FALSE)
# Testing set class labels (binary variable), with cohort information
Test_class <- read.table(file.path(data.path, "Testing_class.txt"), header = TRUE, sep = "\t",
row.names = 1, check.names = FALSE, stringsAsFactors = FALSE)
# Get common samples between expression data and class labels
comsam <- intersect(rownames(Test_class), colnames(Test_expr))
Test_expr <- Test_expr[, comsam]
Test_class <- Test_class[comsam, , drop = FALSE]

# Get common genes
comgene <- intersect(rownames(Train_expr), rownames(Test_expr))
Train_expr <- t(Train_expr[comgene, ])  # Transpose for model input
Test_expr <- t(Test_expr[comgene, ])  # Transpose for model input

# Normalize data for each cohort
Train_set = scaleData(data = Train_expr, centerFlags = TRUE, scaleFlags = TRUE)
Test_set = scaleData(data = Test_expr, cohort = Test_class$Cohort, centerFlags = TRUE,
scaleFlags = TRUE)

# Model training and validation ------------------------------------------
## Model method list
methods <- read.xlsx(file.path(code.path, "methods.xlsx"), startRow = 2)
methods <- methods$Model
methods <- gsub("-| ", "", methods)

## Pre-training for variable selection
Variable = colnames(Train_set)
preTrain.method = strsplit(methods, "\\+")  # Check all methods for variable selection
```

```r
preTrain.method = lapply(preTrain.method, function(x) rev(x)[-1])    # Remove model algorithms and keep variable selection algorithms
preTrain.method = unique(unlist(preTrain.method))    # Unique variable selection algorithms

# Pre-training variables
preTrain.var <- list()
set.seed(seed = 777)
for (method in preTrain.method) {
  preTrain.var[[method]] = RunML(method = method, Train_set = Train_set, Train_label = Train_class, mode = "Variable", classVar = "outcome")
}
preTrain.var[["simple"]] <- colnames(Train_set)    # Record variable selection results for 'simple' method

## Train the model
model <- list()
set.seed(seed = 777)
Train_set_bk = Train_set    # Backup of training set for compatibility with a specific function
for (method in methods) {
  method_name = method
  method <- strsplit(method, "\\+")[[1]]
  if (length(method) == 1) method <- c("simple", method)

  Variable = preTrain.var[[method[1]]]
  Train_set = Train_set_bk[, Variable]
  Train_label = Train_class
  model[[method_name]] <- RunML(method = method[2], Train_set = Train_set, Train_label = Train_label, mode = "Model", classVar = "outcome")

  if (length(ExtractVar(model[[method_name]])) <= 5) {
    model[[method_name]] <- NULL
  }
}
Train_set = Train_set_bk
saveRDS(model, file.path(res.path, "model.rds"))

## Multi-variable Logistic Regression model if required
if (FinalModel == "multiLogistic") {
  logisticmodel <- lapply(model, function(fit) {
    tmp <- glm(formula = Train_class$outcome ~ ., family = "binomial", data = as.data.frame(Train_set[, ExtractVar(fit)]))
    tmp$subFeature <- ExtractVar(fit)
    return(tmp)
  })
```

```
}
saveRDS(logisticmodel, file.path(res.path, "logisticmodel.rds"))

## Evaluate the model --------------------------------------------------
model <- readRDS(file.path(res.path, "model.rds"))

# Predict risk scores based on expression data
RS_list <- list()
for (method in names(model)) {
  RS_list[[method]] <- CalPredictScore(fit = model[[method]], new_data = rbind.data.frame(Train_set, Test_set))
}
RS_mat <- as.data.frame(t(do.call(rbind, RS_list)))
write.table(RS_mat, file.path(res.path, "RS_mat.txt"), sep = "\t", row.names = TRUE, col.names = NA, quote = FALSE)

# Predict classification based on expression data
Class_list <- list()
for (method in names(model)) {
  Class_list[[method]] <- PredictClass(fit = model[[method]], new_data = rbind.data.frame(Train_set, Test_set))
}
Class_mat <- as.data.frame(t(do.call(rbind, Class_list)))
write.table(Class_mat, file.path(res.path, "Class_mat.txt"), sep = "\t", row.names = TRUE, col.names = NA, quote = FALSE)

# Extract selected variables
fea_list <- list()
for (method in names(model)) {
  fea_list[[method]] <- ExtractVar(model[[method]])
}

fea_df <- lapply(model, function(fit) {
  data.frame(ExtractVar(fit))
})
fea_df <- do.call(rbind, fea_df)
fea_df$algorithm <- gsub("(.+)\\.(.+$)", "\\1", rownames(fea_df))
colnames(fea_df)[1] <- "features"
write.table(fea_df, file.path(res.path, "fea_df.txt"), sep = "\t", row.names = FALSE, col.names = TRUE, quote = FALSE)

# Compute C-index and AUC for model evaluation
AUC_list <- list()
for (method in names(model)) {
```

```r
  AUC_list[[method]] <- RunEval(fit = model[[method]], Test_set = Test_set, Test_label = Test_class, Train_set = Train_set, Train_label = Train_class, Train_name = "GSE65682", cohortVar = "Cohort", classVar = "outcome")
}
AUC_mat <- do.call(rbind, AUC_list)
write.table(AUC_mat, file.path(res.path, "AUC_mat.txt"), sep = "\t", row.names = TRUE, col.names = TRUE, quote = FALSE)

# Plot -----------------------------------------------------------------
AUC_mat <- read.table(file.path(res.path, "AUC_mat.txt"), sep = "\t", row.names = 1, header = TRUE, check.names = FALSE, stringsAsFactors = FALSE)
avg_AUC <- apply(AUC_mat, 1, mean)
avg_AUC <- sort(avg_AUC, decreasing = TRUE)
AUC_mat <- AUC_mat[names(avg_AUC), ]
fea_sel <- fea_list[[rownames(AUC_mat)[1]]]
avg_AUC <- as.numeric(format(avg_AUC, digits = 3, nsmall = 3))

# Choose color scheme for AUC plot
if (ncol(AUC_mat) < 3) {
  CohortCol <- c("red", "blue")
} else {
  CohortCol <- brewer.pal(n = ncol(AUC_mat), name = "Paired")
}
names(CohortCol) <- colnames(AUC_mat)

cellwidth = 1; cellheight = 0.5
hm <- SimpleHeatmap(AUC_mat, avg_AUC, CohortCol, "steelblue", cellwidth = cellwidth, cellheight = cellheight, cluster_columns = FALSE, cluster_rows = FALSE)

pdf(file.path(fig.path, "AUC.pdf"), width = cellwidth * ncol(AUC_mat) + 3, height = cellheight * nrow(AUC_mat) * 0.45)
draw(hm)
invisible(dev.off())


# Set working path
work.path <- "your_work_directory_here"   # Set the correct working directory
setwd(work.path)

# Set other paths
code.path <- file.path(work.path, "Codes")
data.path <- file.path(work.path, "InputData")
res.path <- file.path(work.path, "Results")
fig.path <- file.path(work.path, "Figures")
```

```
# Create paths if they do not exist
if (!dir.exists(data.path)) dir.create(data.path)
if (!dir.exists(res.path)) dir.create(res.path)
if (!dir.exists(fig.path)) dir.create(fig.path)
if (!dir.exists(code.path)) dir.create(code.path)

# Load necessary libraries
library(openxlsx)
library(survival)
library(randomForestSRC)
library(glmnet)
library(plsRcox)
library(gbm)
library(caret)
library(survcomp)
library(ComplexHeatmap)
library(RColorBrewer)

# Load model training and evaluation scripts
source(file.path(code.path, "ML.R"))

# Choose final model type: "panML" for multi-algorithm models or "multiCox" for Cox models
FinalModel <- c("panML", "multiCox")[2]

## Training Cohort -------------------------------------------------------
# Load training set gene expression data and survival data
Train_expr <- read.table(file.path(data.path, "Training_expr.txt"), header = TRUE, sep = "\t",
row.names = 1, check.names = FALSE, stringsAsFactors = FALSE)
Train_surv <- read.table(file.path(data.path, "Training_surv.txt"), header = TRUE, sep = "\t",
row.names = 1, check.names = FALSE, stringsAsFactors = FALSE)
comsam <- intersect(rownames(Train_surv), colnames(Train_expr))
Train_expr <- Train_expr[, comsam]
Train_surv <- Train_surv[comsam, , drop = FALSE]

## Validation Cohort -----------------------------------------------------
# Load test set gene expression data and survival data
Test_expr <- read.table(file.path(data.path, "Testing_expr.txt"), header = TRUE, sep = "\t",
row.names = 1, check.names = FALSE, stringsAsFactors = FALSE)
Test_surv <- read.table(file.path(data.path, "Testing_surv.txt"), header = TRUE, sep = "\t",
row.names = 1, check.names = FALSE, stringsAsFactors = FALSE)
comsam <- intersect(rownames(Test_surv), colnames(Test_expr))
Test_expr <- Test_expr[, comsam]
Test_surv <- Test_surv[comsam, , drop = FALSE]
```

```
# Extract common genes between train and test sets
comgene <- intersect(rownames(Train_expr), rownames(Test_expr))
Train_expr <- t(Train_expr[comgene, ])    # Transpose for model input
Test_expr <- t(Test_expr[comgene, ])    # Transpose for model input

# Normalize data for each cohort
Train_set = scaleData(data = Train_expr, centerFlags = TRUE, scaleFlags = TRUE)
Test_set = scaleData(data = Test_expr, cohort = Test_surv$Cohort, centerFlags = FALSE,
scaleFlags = FALSE)

# Model training and validation -----------------------------------------

## Model method list
methods <- read.xlsx(file.path(code.path, "methods.xlsx"), startRow = 2)
methods <- methods$Model
methods <- gsub("-| ", "", methods)

## Pre-training for variable selection
Variable = colnames(Train_expr)
preTrain.method = strsplit(methods, "\\+")
preTrain.method = lapply(preTrain.method, function(x) rev(x)[-1])
preTrain.method = unique(unlist(preTrain.method))

# Pre-train variables
preTrain.var <- list()
set.seed(seed = 123)
for (method in preTrain.method) {
  preTrain.var[[method]] = RunML(method = method, Train_expr = Train_set, Train_surv =
Train_surv, mode = "Variable", classVar = "outcome")
}
preTrain.var[["simple"]] <- colnames(Train_expr)

## Train the model
model <- list()
set.seed(seed = 123)
Train_set_bk = Train_set
for (method in methods) {
  method_name = method
  method <- strsplit(method, "\\+")[[1]]

  if (length(method) == 1) method <- c("simple", method)

  selected.var = preTrain.var[[method[1]]]
```

```
    if (length(selected.var) <= 5) {
      model[[method_name]] <- NULL
    } else {
      model[[method_name]] <- RunML(method = method[2], Train_expr = Train_expr[, selected.var], Train_surv = Train_surv, mode = "Model", classVar = "outcome")
    }

    if (length(ExtractVar(model[[method_name]])) <= 5) {
      model[[method_name]] <- NULL
    }
}
saveRDS(model, file.path(res.path, "model.rds"))

## Multi-variable Cox model if required
if (FinalModel == "multiCox") {
  coxmodel <- lapply(model, function(fit) {
    tmp <- coxph(formula = Surv(Train_surv$OS.time, Train_surv$OS) ~ ., data = as.data.frame(Train_set[, ExtractVar(fit)]))
    tmp$subFeature <- ExtractVar(fit)
    return(tmp)
  })
}
saveRDS(coxmodel, file.path(res.path, "coxmodel.rds"))

## Evaluate the model ----------------------------------------------------
model <- readRDS(file.path(res.path, "model.rds"))

# Predict risk scores based on expression data
RS_list <- list()
for (method in names(model)) {
  RS_list[[method]] <- CalRiskScore(fit = model[[method]], new_data = rbind.data.frame(Train_set, Test_set))
}
RS_mat <- as.data.frame(t(do.call(rbind, RS_list)))
write.table(RS_mat, file.path(res.path, "RS_mat.txt"), sep = "\t", row.names = TRUE, col.names = NA, quote = FALSE)

# Predict classification based on expression data
Class_list <- list()
for (method in names(model)) {
  Class_list[[method]] <- PredictClass(fit = model[[method]], new_data = rbind.data.frame(Train_set, Test_set))
}
Class_mat <- as.data.frame(t(do.call(rbind, Class_list)))
```

```r
write.table(Class_mat, file.path(res.path, "Class_mat.txt"), sep = "\t", row.names = TRUE, col.names = NA, quote = FALSE)

# Extract selected variables
fea_list <- list()
for (method in names(model)) {
  fea_list[[method]] <- ExtractVar(model[[method]])
}

fea_df <- lapply(model, function(fit) {
  data.frame(ExtractVar(fit))
})
fea_df <- do.call(rbind, fea_df)
fea_df$algorithm <- gsub("(.+)\\.(.+$)", "\\1", rownames(fea_df))
colnames(fea_df)[1] <- "features"
write.table(fea_df, file.path(res.path, "fea_df.txt"), sep = "\t", row.names = FALSE, col.names = TRUE, quote = FALSE)

# Compute C-index and AUC for model evaluation
Cindexlist <- list()
for (method in names(model)) {
  Cindexlist[[method]] <- RunEval(fit = model[[method]], Test_expr = Test_set, Test_surv = Test_surv, Train_expr = NULL, Train_surv = NULL, cohortVar = "Cohort", timeVar = "OS.time", statusVar = "OS")
}
Cindex_mat <- do.call(rbind, Cindexlist)
write.table(Cindex_mat, file.path(res.path, "cindex_mat.txt"), sep = "\t", row.names = TRUE, col.names = TRUE, quote = FALSE)

# Plot ----------------------------------------------------------------------

Cindex_mat <- read.table(file.path(res.path, "cindex_mat.txt"), sep = "\t", row.names = 1, header = TRUE, check.names = FALSE, stringsAsFactors = FALSE)
avg_Cindex <- sort(apply(Cindex_mat, 1, mean), decreasing = TRUE) # Sort by average C-index
Cindex_mat <- Cindex_mat[names(avg_Cindex), ] # Sort C-index matrix
avg_Cindex <- as.numeric(format(avg_Cindex, digits = 3, nsmall = 3)) # Round to 3 decimal places

# Set color scheme for C-index plot
CohortCol <- brewer.pal(n = ncol(Cindex_mat), name = "Paired") # Define color palette
names(CohortCol) <- colnames(Cindex_mat)

cellwidth = 1; cellheight = 0.5
hm <- SimpleHeatmap(Cindex_mat, avg_Cindex, CohortCol, "steelblue", cellwidth = cellwidth,
```

```
cellheight = cellheight, cluster_columns = FALSE, cluster_rows = FALSE)

# Save plot to PDF
pdf(file.path(fig.path, "Cindex_heatmap.pdf"), width = cellwidth * ncol(Cindex_mat) + 3, height
= cellheight * nrow(Cindex_mat) * 0.45)
draw(hm, heatmap_legend_side = "right", annotation_legend_side = "right")
invisible(dev.off())
```

Single-cell sequencing analysis:

```
# Load necessary libraries
library(dplyr)
library(Seurat)
library(patchwork)
library(ggplot2)
library(SingleR)
library(CCA)
library(clustree)
library(cowplot)
library(monocle)
library(tidyverse)
library(SCpubr)
library(UCell)
library(irGSEA)
library(GSVA)
library(GSEABase)
library(harmony)
library(plyr)

# Set directory paths
data_dir <- "J:/sepsis/GSE167363_RAW/standard10Xdata/data"
samples <- list.files(data_dir)
sample_dir <- file.path(data_dir, samples[1])

# Load the 10X data
afdata <- Read10X(data.dir = sample_dir)

# Create a Seurat object
af <- CreateSeuratObject(counts = afdata,
                         project = "SeuratObject",
                         min.cells = 3,
                         min.features = 200)

# Assign identities and metadata
```

```
af$Type <- factor(samples)
af[["percent.mt"]] <- PercentageFeatureSet(af, pattern = "^MT-")
af[["percent.rb"]] <- PercentageFeatureSet(af, pattern = "^RP")

# Filtering cells based on quality control criteria
filter_by_nCount <- af$nCount_RNA >= 1000
filter_by_nFeature <- af$nFeature_RNA >= 200 & af$nFeature_RNA <= 10000
filter_by_percent_mt <- af$percent.mt <= 20
filter_by_percent_rb <- af$percent.rb <= 20

# Apply all filters
filter_mask <- filter_by_nFeature & filter_by_percent_mt & filter_by_percent_rb
af <- af[, filter_mask]

# Save the processed Seurat object
saveRDS(af, "af_processed.rds")

# Set working directory
setwd("J:/sepsis/GSE167363_RAW/merged_samples")

# Load necessary libraries
library(Seurat)
library(tidyverse)
library(ggplot2)
library(patchwork)
library(SingleR)
library(cowplot)
library(monocle)
library(ComplexHeatmap)
library(RColorBrewer)
library(ggpubr)
library(randomcoloR)
library(CellChat)

# Load the Seurat object
af <- readRDS("af.rds")

# QC Visualization: Violin plots for QC metrics
pdf(file = "01.vlnplot.pdf", width = 20, height = 5)
VlnPlot(af, features = c("nFeature_RNA", "nCount_RNA", "percent.mt", "percent.rb"), ncol = 4)
+
  scale_fill_manual(values = c("#58CDD9", "#7A142C", "#5D90BA", "#431A3D", "#91612D"))
dev.off()
```

```r
# QC Correlation: Scatter plots for correlation between QC metrics
plot1 <- FeatureScatter(af, feature1 = "nCount_RNA", feature2 = "percent.mt") + RotatedAxis()
plot2 <- FeatureScatter(af, feature1 = "nCount_RNA", feature2 = "percent.rb") + RotatedAxis()
plot3 <- FeatureScatter(af, feature1 = "nCount_RNA", feature2 = "nFeature_RNA") +
RotatedAxis()

# Combine scatter plots
pdf(file = "01.corqc.pdf", width = 12, height = 5)
plot1 + plot2 + plot3 + plot_layout(ncol = 3)
dev.off()

# Normalize data using LogNormalize method
af <- NormalizeData(af, normalization.method = "LogNormalize", scale.factor = 10000)

# Identify highly variable genes (top 2000)
af <- FindVariableFeatures(af, selection.method = "vst", nfeatures = 2000)

# Get top 10 variable genes
top10 <- head(VariableFeatures(af), 10)

# Plot variable genes
plot1 <- VariableFeaturePlot(af)
plot2 <- LabelPoints(plot = plot1, points = top10, repel = TRUE)

pdf(file = "01.topgene.pdf", width = 7, height = 6)
plot2
dev.off()

# Scale the data (mean = 0, variance = 1)
af <- ScaleData(af)

# PCA and tSNE for dimensionality reduction
af <- RunPCA(af, features = VariableFeatures(object = af))
af <- RunTSNE(af, dims = 1:13)

# Visualize tSNE and PCA
pdf(file = "02.rawtsne.pdf", width = 7, height = 5.5)
DimPlot(af, reduction = "tsne", pt.size = 1) + theme_classic() +
  theme(panel.border = element_rect(fill = NA, color = "black", size = 0.5, linetype = "solid"),
        legend.position = "right")
dev.off()

pdf(file = "02.rawpca.pdf", width = 7, height = 5.5)
DimPlot(af, reduction = "pca", pt.size = 1) + theme_classic() +
```

```
  theme(panel.border = element_rect(fill = NA, color = "black", size = 0.5, linetype = "solid"),
        legend.position = "right")
dev.off()

# Split tSNE by Type
colaa <- distinctColorPalette(100)
pdf(file = "02.raw.tsne.split.pdf", width = 12, height = 10)
do_DimPlot(sample = af, plot.title = "", reduction = "tsne", legend.position = "bottom", dims = c(1,
2), split.by = "Type", pt.size = 0.5)
dev.off()

# Harmony batch effect correction (optional)
af <- RunHarmony(af, group.by.vars = "Type")

# Visualize Harmony corrected results
pdf(file = "03.harmony.pdf", width = 7.5, height = 5.5)
DimPlot(af, reduction = "harmony", pt.size = 1) + theme_classic() +
  theme(panel.border = element_rect(fill = NA, color = "black", size = 0.5, linetype = "solid"),
        legend.position = "right")
dev.off()

af <- RunTSNE(af, dims = 1:13, reduction = 'harmony')
pdf(file = "03.tsne.pdf", width = 7, height = 5.5)
DimPlot(af, reduction = "tsne", pt.size = 1) + theme_classic() +
  theme(panel.border = element_rect(fill = NA, color = "black", size = 0.5, linetype = "solid"),
        legend.position = "right")
dev.off()

# Clustering the cells
af <- FindNeighbors(af, dims = 1:13, reduction = "harmony")
af <- FindClusters(af, resolution = 1.2)

# Visualize clusters in tSNE
pdf(file = "05-cluster.TSEN.pdf", width = 6.5, height = 5)
DimPlot(af, reduction = "tsne", label = TRUE, pt.size = 1) + theme_classic() +
  theme(panel.border = element_rect(fill = NA, color = "black", size = 0.5, linetype = "solid"),
        legend.position = "right")
dev.off()

# Identifying cell markers
af.markers <- FindAllMarkers(af, only.pos = TRUE, min.pct = 0.25, logfc.threshold = 0.25)
write.csv(af.markers, file = "05.cluster_markers.csv")

# Visualize top markers for each cluster
```

```
top5af.markers <- af.markers %>%
   group_by(cluster) %>%
   top_n(n = 5, wt = avg_log2FC)

# Heatmap for top markers
pdf(file = "09-cell_marker.heatmap.pdf", width = 15, height = 6)
DoHeatmap(af, features = top5af.markers$gene, group.colors = colaa) +
   scale_fill_gradient2(low = '#0099CC', mid = 'white', high = '#CC0033', name = 'Z-score')
dev.off()

# Feature plots for selected genes
afgenes <- read.table("ppi.hub.txt", header = FALSE, sep = "\t")[, 1]
pdf(file = "09-cell_FeaturePlot.pdf", width = 27.5, height = 5)
FeaturePlot(af, reduction = "tsne", features = afgenes, cols = c("grey", "red"), pt.size = 0.5, slot =
"counts")
dev.off()

# Violin plot for selected genes
pdf(file = "09-cell_VlnPlot.pdf", width = 15, height = 10)
VlnPlot(af, features = afgenes, group.by = "cellType", stack = TRUE, cols = colaa, slot = "counts")
+ NoLegend()
dev.off()
```

**Package**	**Version**
Seurat	4.4.0
tidyverse	2.0.0
Matrix	1.6.5
stringr	1.5.1
dplyr	1.1.4
patchwork	1.3.0
ggplot2	3.5.2
SingleR	2.4.1
CCA	1.2.2
clustree	0.5.1
cowplot	1.1.3
monocle	2.30.1
SCpubr	2.0.2
UCell	2.13.1
irGSEA	3.3.2
GSVA	1.50.5

Package	Version
GSEABase	1.64.0
harmony	1.2.3
plyr	1.8.9
randomcoloR	1.1.0
CellChat	1.6.1
ggpubr	0.6.1
openxlsx	4.2.8
seqinr	4.2.36
randomForestSRC	2.9.3
glmnet	4.1.9
plsRglm	1.5.1
gbm	2.2.2
mboost	2.9.11
e1071	1.7.16
BART	2.9.9
snowfall	1.84.6.3
xgboost	1.7.11.1
ComplexHeatmap	2.18.0
RColorBrewer	1.1.3
survival	3.8.3
survminer	0.4.9
survcomp	1.50.0
CoxBoost	1.4
survivalsvm	0.0.5
mixOmics	6.24.0
