# Supplementary material for: Machine learning-based identification of leptin-associated biomarkers and prognostic prediction models in sepsis
Source: Front Cell Infect Microbiol. 2025 Sep 29;15:1630446. doi: 10.3389/fcimb.2025.1630446 (PMC12515905; doi:10.3389/fcimb.2025.1630446)
Supplement: Supplementary file 2 [file Table1.doc]

Supplementary Table 1. The primers

| Gene | Forward | Reverse |
| --- | --- | --- |
| TFRC | 5′-ACCATTGTCATATACCCGGTTCA-3′ | 5′-CAATAGCCCAAGTAGCCAATCAT-3′ |
| PILRA | 5′-CTGGAGGCTCAGTAGCACAAC-3′ | 5′-GGAGGCAGATCAGTCCCAAAAT-3′ |
| GAPDH | 5′-GGAGCGAGATCCCTCCAAAAT-3′ | 5′-GGCTGTTGTCATACTTCTCATGG-3′ |
