# Supplementary material for: Machine learning-based identification of leptin-associated biomarkers and prognostic prediction models in sepsis
Source: Front Cell Infect Microbiol. 2025 Sep 29;15:1630446. doi: 10.3389/fcimb.2025.1630446 (PMC12515905; doi:10.3389/fcimb.2025.1630446)
Supplement: Supplementary file 3 [file Table2.doc]

Supplementary Table 2. Basic information of the datasets used in this study. All samples listed have been retained after the exclusion of low-quality or unqualified entries.

| Dataset | Country | Sample Source | No. of Sepsis Cases | No. of Control Cases | Timing of mortality | Mortality |
| --- | --- | --- | --- | --- | --- | --- |
| GSE65682 | Malta | Blood | 479 | 42 | 28-day | 114 |
| GSE63042 | USA | Blood | 106 | 0 | 28-day | 28 |
| E-MTAB-4451 | UK | Blood | 106 | 0 | 28-day | 52 |
| E-MTAB-5273 | USA | Blood | 118 | 0 | 28-day | 22 |
| E-MTAB-7581 | UK | Blood | 176 | 0 | 28-day | 48 |
| GSE54514 | Australia | Blood | 35 | 18 | 5-day | 9 |
| GSE95233 | Germany | Blood | 51 | 22 | 3-day | 17 |
| GSE57065 | France | Blood | 28 | 25 | - | - |
| GSE131761 | Spain | Blood | 81 | 15 | - | - |
