# Supplementary material for: Machine learning-based identification of leptin-associated biomarkers and prognostic prediction models in sepsis
Source: Front Cell Infect Microbiol. 2025 Sep 29;15:1630446. doi: 10.3389/fcimb.2025.1630446 (PMC12515905; doi:10.3389/fcimb.2025.1630446)
Supplement: Supplementary file 4 [file Table3.doc]

Supplementary Table 3. Genes associated with Leptin

| Gene Symbol | Description | Category | Uniprot ID | Gifts | GC Id | Relevance score | GeneCards Link |
| --- | --- | --- | --- | --- | --- | --- | --- |
| LEP | Leptin | Protein Coding | P41159 | 59 | GC07P128241 | 178.312805175781 | https://www.genecards.org/cgi-bin/carddisp.pl?gene=LEP |
| LEPR | Leptin Receptor | Protein Coding | P48357 | 62 | GC01P078814 | 142.979476928711 | https://www.genecards.org/cgi-bin/carddisp.pl?gene=LEPR |
| LEPQTL1 | Leptin, Serum Levels Of | Genetic Locus |  | 10 | GC02U903086 | 63.7883644104004 | https://www.genecards.org/cgi-bin/carddisp.pl?gene=LEPQTL1 |
| PPARG | Peroxisome Proliferator Activated Receptor Gamma | Protein Coding | P37231 | 65 | GC03P012287 | 39.5188064575195 | https://www.genecards.org/cgi-bin/carddisp.pl?gene=PPARG |
| ADIPOQ | Adiponectin, C1Q And Collagen Domain Containing | Protein Coding | Q15848 | 59 | GC03P186842 | 39.2627792358398 | https://www.genecards.org/cgi-bin/carddisp.pl?gene=ADIPOQ |
| LEPROT | Leptin Receptor Overlapping Transcript | Protein Coding | O15243 | 42 | GC01P065420 | 36.6849517822266 | https://www.genecards.org/cgi-bin/carddisp.pl?gene=LEPROT |
| INS | Insulin | Protein Coding | P01308 | 61 | GC11M002159 | 35.8083724975586 | https://www.genecards.org/cgi-bin/carddisp.pl?gene=INS |
| UCP3 | Uncoupling Protein 3 | Protein Coding | P55916 | 54 | GC11M074000 | 32.8001594543457 | https://www.genecards.org/cgi-bin/carddisp.pl?gene=UCP3 |
| GHRL | Ghrelin And Obestatin Prepropeptide | Protein Coding | Q9UBU3 | 54 | GC03M010285 | 27.8977317810059 | https://www.genecards.org/cgi-bin/carddisp.pl?gene=GHRL |
| STAT3 | Signal Transducer And Activator Of Transcription 3 | Protein Coding | P40763 | 67 | GC17M042313 | 23.7448081970215 | https://www.genecards.org/cgi-bin/carddisp.pl?gene=STAT3 |
| IGF1 | Insulin Like Growth Factor 1 | Protein Coding | P05019 | 61 | GC12M102395 | 23.3697109222412 | https://www.genecards.org/cgi-bin/carddisp.pl?gene=IGF1 |
| RETN | Resistin | Protein Coding | Q9HD89 | 55 | GC19P007669 | 22.7095375061035 | https://www.genecards.org/cgi-bin/carddisp.pl?gene=RETN |
| NPY | Neuropeptide Y | Protein Coding | P01303 | 58 | GC07P024850 | 22.4265556335449 | https://www.genecards.org/cgi-bin/carddisp.pl?gene=NPY |
| JAK2 | Janus Kinase 2 | Protein Coding | O60674 | 66 | GC09P004985 | 20.7167873382568 | https://www.genecards.org/cgi-bin/carddisp.pl?gene=JAK2 |
| SOCS3 | Suppressor Of Cytokine Signaling 3 | Protein Coding | O14543 | 57 | GC17M078356 | 20.5928249359131 | https://www.genecards.org/cgi-bin/carddisp.pl?gene=SOCS3 |
| POMC | Proopiomelanocortin | Protein Coding | P01189 | 61 | GC02M025160 | 20.1258563995361 | https://www.genecards.org/cgi-bin/carddisp.pl?gene=POMC |
| LEPROTL1 | Leptin Receptor Overlapping Transcript Like 1 | Protein Coding | O95214 | 41 | GC08P030095 | 19.4866886138916 | https://www.genecards.org/cgi-bin/carddisp.pl?gene=LEPROTL1 |
| CRP | C-Reactive Protein | Protein Coding | P02741 | 58 | GC01M167583 | 19.4573612213135 | https://www.genecards.org/cgi-bin/carddisp.pl?gene=CRP |
| IL6 | Interleukin 6 | Protein Coding | P05231 | 64 | GC07P022725 | 18.2270259857178 | https://www.genecards.org/cgi-bin/carddisp.pl?gene=IL6 |
| TNF | Tumor Necrosis Factor | Protein Coding | P01375 | 65 | GC06P181893 | 17.2504768371582 | https://www.genecards.org/cgi-bin/carddisp.pl?gene=TNF |
| MC4R | Melanocortin 4 Receptor | Protein Coding | P32245 | 58 | GC18M060371 | 16.6867332458496 | https://www.genecards.org/cgi-bin/carddisp.pl?gene=MC4R |
| IRS1 | Insulin Receptor Substrate 1 | Protein Coding | P35568 | 61 | GC02M226731 | 15.287971496582 | https://www.genecards.org/cgi-bin/carddisp.pl?gene=IRS1 |
| IRS2 | Insulin Receptor Substrate 2 | Protein Coding | Q9Y4H2 | 59 | GC13M109752 | 14.4320402145386 | https://www.genecards.org/cgi-bin/carddisp.pl?gene=IRS2 |
| LOC106728418 | LEP 5' Regulatory Region | Functional Element |  | 2 | GC07P128238 | 14.3821506500244 | https://www.genecards.org/cgi-bin/carddisp.pl?gene=LOC106728418 |
| SH2B1 | SH2B Adaptor Protein 1 | Protein Coding | Q9NRF2 | 54 | GC16P120910 | 14.2876567840576 | https://www.genecards.org/cgi-bin/carddisp.pl?gene=SH2B1 |
| LMNA | Lamin A/C | Protein Coding | P02545 | 63 | GC01P156082 | 14.267765045166 | https://www.genecards.org/cgi-bin/carddisp.pl?gene=LMNA |
| INSR | Insulin Receptor | Protein Coding | P06213 | 67 | GC19M007112 | 13.4958772659302 | https://www.genecards.org/cgi-bin/carddisp.pl?gene=INSR |
| NAMPT | Nicotinamide Phosphoribosyltransferase | Protein Coding | P43490 | 60 | GC07M106248 | 13.2557229995728 | https://www.genecards.org/cgi-bin/carddisp.pl?gene=NAMPT |
| LIPE | Lipase E, Hormone Sensitive Type | Protein Coding | Q05469 | 61 | GC19M042401 | 13.2521305084229 | https://www.genecards.org/cgi-bin/carddisp.pl?gene=LIPE |
| ADRB3 | Adrenoceptor Beta 3 | Protein Coding | P13945 | 55 | GC08M037962 | 13.1846408843994 | https://www.genecards.org/cgi-bin/carddisp.pl?gene=ADRB3 |
| ADIPOR1 | Adiponectin Receptor 1 | Protein Coding | Q96A54 | 55 | GC01M202940 | 12.9712886810303 | https://www.genecards.org/cgi-bin/carddisp.pl?gene=ADIPOR1 |
| CCK | Cholecystokinin | Protein Coding | P06307 | 56 | GC03M042274 | 12.9301338195801 | https://www.genecards.org/cgi-bin/carddisp.pl?gene=CCK |
| HCRT | Hypocretin Neuropeptide Precursor | Protein Coding | O43612 | 53 | GC17M097206 | 12.8593692779541 | https://www.genecards.org/cgi-bin/carddisp.pl?gene=HCRT |
| PTPN1 | Protein Tyrosine Phosphatase Non-Receptor Type 1 | Protein Coding | P18031 | 62 | GC20P050510 | 12.8583240509033 | https://www.genecards.org/cgi-bin/carddisp.pl?gene=PTPN1 |
| AGRP | Agouti Related Neuropeptide | Protein Coding | O00253 | 53 | GC16M067482 | 12.4710178375244 | https://www.genecards.org/cgi-bin/carddisp.pl?gene=AGRP |
| CARTPT | CART Prepropeptide | Protein Coding | Q16568 | 55 | GC05P071719 | 11.9750175476074 | https://www.genecards.org/cgi-bin/carddisp.pl?gene=CARTPT |
| LPL | Lipoprotein Lipase | Protein Coding | P06858 | 63 | GC08P019901 | 11.9736528396606 | https://www.genecards.org/cgi-bin/carddisp.pl?gene=LPL |
| GCG | Glucagon | Protein Coding | P01275 | 55 | GC02M162142 | 11.9732131958008 | https://www.genecards.org/cgi-bin/carddisp.pl?gene=GCG |
| FTO | FTO Alpha-Ketoglutarate Dependent Dioxygenase | Protein Coding | Q9C0B1 | 59 | GC16P121535 | 11.935450553894 | https://www.genecards.org/cgi-bin/carddisp.pl?gene=FTO |
| UCP1 | Uncoupling Protein 1 | Protein Coding | P25874 | 56 | GC04M140559 | 11.8503694534302 | https://www.genecards.org/cgi-bin/carddisp.pl?gene=UCP1 |
| SERPINE1 | Serpin Family E Member 1 | Protein Coding | P05121 | 63 | GC07P101127 | 11.810131072998 | https://www.genecards.org/cgi-bin/carddisp.pl?gene=SERPINE1 |
| ADIPOR2 | Adiponectin Receptor 2 | Protein Coding | Q86V24 | 54 | GC12P001670 | 11.7045927047729 | https://www.genecards.org/cgi-bin/carddisp.pl?gene=ADIPOR2 |
| AKT1 | AKT Serine/Threonine Kinase 1 | Protein Coding | P31749 | 66 | GC14M104769 | 11.1201648712158 | https://www.genecards.org/cgi-bin/carddisp.pl?gene=AKT1 |
| BDNF-AS | BDNF Antisense RNA | RNA Gene |  | 31 | GC11P027466 | 11.022102355957 | https://www.genecards.org/cgi-bin/carddisp.pl?gene=BDNF-AS |
| PYY | Peptide YY | Protein Coding | P10082 | 56 | GC17M043952 | 10.9575910568237 | https://www.genecards.org/cgi-bin/carddisp.pl?gene=PYY |
| IL1B | Interleukin 1 Beta | Protein Coding | P01584 | 60 | GC02M112829 | 10.8272972106934 | https://www.genecards.org/cgi-bin/carddisp.pl?gene=IL1B |
| CCL2 | C-C Motif Chemokine Ligand 2 | Protein Coding | P13500 | 62 | GC17P034255 | 10.6242408752441 | https://www.genecards.org/cgi-bin/carddisp.pl?gene=CCL2 |
| IGFBP3 | Insulin Like Growth Factor Binding Protein 3 | Protein Coding | P17936 | 59 | GC07M045912 | 10.3889455795288 | https://www.genecards.org/cgi-bin/carddisp.pl?gene=IGFBP3 |
| CRH | Corticotropin Releasing Hormone | Protein Coding | P06850 | 56 | GC08M066176 | 10.2961235046387 | https://www.genecards.org/cgi-bin/carddisp.pl?gene=CRH |
| GNRH1 | Gonadotropin Releasing Hormone 1 | Protein Coding | P01148 | 54 | GC08M025419 | 9.9046106338501 | https://www.genecards.org/cgi-bin/carddisp.pl?gene=GNRH1 |
| ADCY3 | Adenylate Cyclase 3 | Protein Coding | O60266 | 60 | GC02M024819 | 9.82350444793701 | https://www.genecards.org/cgi-bin/carddisp.pl?gene=ADCY3 |
| IAPP | Islet Amyloid Polypeptide | Protein Coding | P10997 | 54 | GC12P021354 | 9.76397609710693 | https://www.genecards.org/cgi-bin/carddisp.pl?gene=IAPP |
| SHBG | Sex Hormone Binding Globulin | Protein Coding | P04278 | 55 | GC17P007613 | 9.65312767028809 | https://www.genecards.org/cgi-bin/carddisp.pl?gene=SHBG |
| GHSR | Growth Hormone Secretagogue Receptor | Protein Coding | Q92847 | 58 | GC03M172443 | 9.62748622894287 | https://www.genecards.org/cgi-bin/carddisp.pl?gene=GHSR |
| KISS1 | KiSS-1 Metastasis Suppressor | Protein Coding | Q15726 | 53 | GC01M204190 | 9.52762413024902 | https://www.genecards.org/cgi-bin/carddisp.pl?gene=KISS1 |
| BDNF | Brain Derived Neurotrophic Factor | Protein Coding | P23560 | 62 | GC11M027654 | 9.4850549697876 | https://www.genecards.org/cgi-bin/carddisp.pl?gene=BDNF |
| IL10 | Interleukin 10 | Protein Coding | P22301 | 60 | GC01M206767 | 9.41746139526367 | https://www.genecards.org/cgi-bin/carddisp.pl?gene=IL10 |
| GHRH | Growth Hormone Releasing Hormone | Protein Coding | P01286 | 50 | GC20M037251 | 9.21876430511475 | https://www.genecards.org/cgi-bin/carddisp.pl?gene=GHRH |
| SLC2A4 | Solute Carrier Family 2 Member 4 | Protein Coding | P14672 | 60 | GC17P153733 | 9.19722843170166 | https://www.genecards.org/cgi-bin/carddisp.pl?gene=SLC2A4 |
| PPARA | Peroxisome Proliferator Activated Receptor Alpha | Protein Coding | Q07869 | 57 | GC22P046150 | 9.18074512481689 | https://www.genecards.org/cgi-bin/carddisp.pl?gene=PPARA |
| ITLN1 | Intelectin 1 | Protein Coding | Q8WWA0 | 51 | GC01M160876 | 9.16508960723877 | https://www.genecards.org/cgi-bin/carddisp.pl?gene=ITLN1 |
| TRH | Thyrotropin Releasing Hormone | Protein Coding | P20396 | 54 | GC03P129974 | 9.12635231018066 | https://www.genecards.org/cgi-bin/carddisp.pl?gene=TRH |
| GH1 | Growth Hormone 1 | Protein Coding | P01241 | 57 | GC17M063917 | 9.09011554718018 | https://www.genecards.org/cgi-bin/carddisp.pl?gene=GH1 |
| MC3R | Melanocortin 3 Receptor | Protein Coding | P41968 | 53 | GC20P056248 | 8.7249813079834 | https://www.genecards.org/cgi-bin/carddisp.pl?gene=MC3R |
| GPT | Glutamic--Pyruvic Transaminase | Protein Coding | P24298 | 55 | GC08P144502 | 8.71378231048584 | https://www.genecards.org/cgi-bin/carddisp.pl?gene=GPT |
| PPARGC1A | PPARG Coactivator 1 Alpha | Protein Coding | Q9UBK2 | 60 | GC04M023755 | 8.60847663879395 | https://www.genecards.org/cgi-bin/carddisp.pl?gene=PPARGC1A |
| RARRES2 | Retinoic Acid Receptor Responder 2 | Protein Coding | Q99969 | 51 | GC07M150333 | 8.57173252105713 | https://www.genecards.org/cgi-bin/carddisp.pl?gene=RARRES2 |
| OXT | Oxytocin/Neurophysin I Prepropeptide | Protein Coding | P01178 | 50 | GC20P010955 | 8.38292407989502 | https://www.genecards.org/cgi-bin/carddisp.pl?gene=OXT |
| LOC122094844 | Sharpr-MPRA Regulatory Region 5730 | Functional Element |  | 2 | GC01P078826 | 8.37927341461182 | https://www.genecards.org/cgi-bin/carddisp.pl?gene=LOC122094844 |
| PIK3CG | Phosphatidylinositol-4,5-Bisphosphate 3-Kinase Catalytic Subunit Gamma | Protein Coding | P48736 | 62 | GC07P106865 | 8.33601284 | https://www.genecards.org/cgi-bin/carddisp.pl?gene=PIK3CG |
| SREBF1 | Sterol Regulatory Element Binding Transcription Factor 1 | Protein Coding | P36956 | 61 | GC17M017810 | 8.24373149871826 | https://www.genecards.org/cgi-bin/carddisp.pl?gene=SREBF1 |
| PCSK1 | Proprotein Convertase Subtilisin/Kexin Type 1 | Protein Coding | P29120 | 62 | GC05M096391 | 8.2302770614624 | https://www.genecards.org/cgi-bin/carddisp.pl?gene=PCSK1 |
| BBS1 | Bardet-Biedl Syndrome 1 | Protein Coding | Q8NFJ9 | 52 | GC11P107927 | 8.20535850524902 | https://www.genecards.org/cgi-bin/carddisp.pl?gene=BBS1 |
| FGF21 | Fibroblast Growth Factor 21 | Protein Coding | Q9NSA1 | 52 | GC19P152833 | 8.01500129699707 | https://www.genecards.org/cgi-bin/carddisp.pl?gene=FGF21 |
| MAPK1 | Mitogen-Activated Protein Kinase 1 | Protein Coding | P28482 | 65 | GC22M021759 | 7.55777740478516 | https://www.genecards.org/cgi-bin/carddisp.pl?gene=MAPK1 |
| LRP2 | LDL Receptor Related Protein 2 | Protein Coding | P98164 | 61 | GC02M169127 | 7.35645532608032 | https://www.genecards.org/cgi-bin/carddisp.pl?gene=LRP2 |
| MAGEL2 | MAGE Family Member L2 | Protein Coding | Q9UJ55 | 50 | GC15M023643 | 7.17162418365479 | https://www.genecards.org/cgi-bin/carddisp.pl?gene=MAGEL2 |
| AGPAT2 | 1-Acylglycerol-3-Phosphate O-Acyltransferase 2 | Protein Coding | O15120 | 57 | GC09M136673 | 7.1342568397522 | https://www.genecards.org/cgi-bin/carddisp.pl?gene=AGPAT2 |
| SIM1 | SIM BHLH Transcription Factor 1 | Protein Coding | P81133 | 52 | GC06M108600 | 7.03452205657959 | https://www.genecards.org/cgi-bin/carddisp.pl?gene=SIM1 |
| DPP4 | Dipeptidyl Peptidase 4 | Protein Coding | P27487 | 62 | GC02M161992 | 7.02478456497192 | https://www.genecards.org/cgi-bin/carddisp.pl?gene=DPP4 |
| IGFBP1 | Insulin Like Growth Factor Binding Protein 1 | Protein Coding | P08833 | 55 | GC07P051058 | 7.00040721893311 | https://www.genecards.org/cgi-bin/carddisp.pl?gene=IGFBP1 |
| PMCH | Pro-Melanin Concentrating Hormone | Protein Coding | P20382 | 47 | GC12M102196 | 6.96380758285522 | https://www.genecards.org/cgi-bin/carddisp.pl?gene=PMCH |
| UCP2 | Uncoupling Protein 2 | Protein Coding | P55851 | 58 | GC11M073974 | 6.79357051849365 | https://www.genecards.org/cgi-bin/carddisp.pl?gene=UCP2 |
| MCHR1 | Melanin Concentrating Hormone Receptor 1 | Protein Coding | Q99705 | 55 | GC22P040679 | 6.76140069961548 | https://www.genecards.org/cgi-bin/carddisp.pl?gene=MCHR1 |
| PRL | Prolactin | Protein Coding | P01236 | 56 | GC06M022287 | 6.66201305389404 | https://www.genecards.org/cgi-bin/carddisp.pl?gene=PRL |
| BSCL2 | BSCL2 Lipid Droplet Biogenesis Associated, Seipin | Protein Coding | Q96G97 | 55 | GC11M139665 | 6.59772109985352 | https://www.genecards.org/cgi-bin/carddisp.pl?gene=BSCL2 |
| GLP1R | Glucagon Like Peptide 1 Receptor | Protein Coding | P43220 | 60 | GC06P039048 | 6.57056379318237 | https://www.genecards.org/cgi-bin/carddisp.pl?gene=GLP1R |
| PTPN11 | Protein Tyrosine Phosphatase Non-Receptor Type 11 | Protein Coding | Q06124 | 67 | GC12P112418 | 6.55113172531128 | https://www.genecards.org/cgi-bin/carddisp.pl?gene=PTPN11 |
| NDN | Necdin, MAGE Family Member | Protein Coding | Q99608 | 55 | GC15M047478 | 6.51971435546875 | https://www.genecards.org/cgi-bin/carddisp.pl?gene=NDN |
| PRKAB1 | Protein Kinase AMP-Activated Non-Catalytic Subunit Beta 1 | Protein Coding | Q9Y478 | 59 | GC12P119667 | 6.33082246780396 | https://www.genecards.org/cgi-bin/carddisp.pl?gene=PRKAB1 |
| MFN2 | Mitofusin 2 | Protein Coding | O95140 | 61 | GC01P011980 | 6.20144081115723 | https://www.genecards.org/cgi-bin/carddisp.pl?gene=MFN2 |
| STAT5A | Signal Transducer And Activator Of Transcription 5A | Protein Coding | P42229 | 62 | GC17P042287 | 6.18359661102295 | https://www.genecards.org/cgi-bin/carddisp.pl?gene=STAT5A |
| BGLAP | Bone Gamma-Carboxyglutamate Protein | Protein Coding | P02818 | 52 | GC01P156242 | 6.08608055114746 | https://www.genecards.org/cgi-bin/carddisp.pl?gene=BGLAP |
| RBP4 | Retinol Binding Protein 4 | Protein Coding | P02753 | 58 | GC10M093591 | 6.07896757125854 | https://www.genecards.org/cgi-bin/carddisp.pl?gene=RBP4 |
| CNR1 | Cannabinoid Receptor 1 | Protein Coding | P21554 | 59 | GC06M088139 | 6.02786254882813 | https://www.genecards.org/cgi-bin/carddisp.pl?gene=CNR1 |
| GIP | Gastric Inhibitory Polypeptide | Protein Coding | P09681 | 49 | GC17M048958 | 6.00306987762451 | https://www.genecards.org/cgi-bin/carddisp.pl?gene=GIP |
| PPY | Pancreatic Polypeptide | Protein Coding | P01298 | 51 | GC17M043940 | 5.97999572753906 | https://www.genecards.org/cgi-bin/carddisp.pl?gene=PPY |
| MRAP2 | Melanocortin 2 Receptor Accessory Protein 2 | Protein Coding | Q96G30 | 44 | GC06P182418 | 5.96093416213989 | https://www.genecards.org/cgi-bin/carddisp.pl?gene=MRAP2 |
| SIGLEC6 | Sialic Acid Binding Ig Like Lectin 6 | Protein Coding | O43699 | 45 | GC19M051517 | 5.90052175521851 | https://www.genecards.org/cgi-bin/carddisp.pl?gene=SIGLEC6 |
| ALMS1 | ALMS1 Centrosome And Basal Body Associated Protein | Protein Coding | Q8TCU4 | 51 | GC02P073385 | 5.85068321228027 | https://www.genecards.org/cgi-bin/carddisp.pl?gene=ALMS1 |
| CXCL8 | C-X-C Motif Chemokine Ligand 8 | Protein Coding | P10145 | 57 | GC04P073740 | 5.83303213119507 | https://www.genecards.org/cgi-bin/carddisp.pl?gene=CXCL8 |
| KSR2 | Kinase Suppressor Of Ras 2 | Protein Coding | Q6VAB6 | 48 | GC12M117453 | 5.79970550537109 | https://www.genecards.org/cgi-bin/carddisp.pl?gene=KSR2 |
| VEGFA | Vascular Endothelial Growth Factor A | Protein Coding | P15692 | 61 | GC06P043770 | 5.75886821746826 | https://www.genecards.org/cgi-bin/carddisp.pl?gene=VEGFA |
| TNFRSF11B | TNF Receptor Superfamily Member 11b | Protein Coding | O00300 | 60 | GC08M118923 | 5.75711011886597 | https://www.genecards.org/cgi-bin/carddisp.pl?gene=TNFRSF11B |
| ZMPSTE24 | Zinc Metallopeptidase STE24 | Protein Coding | O75844 | 54 | GC01P040258 | 5.71638679504395 | https://www.genecards.org/cgi-bin/carddisp.pl?gene=ZMPSTE24 |
| NTRK2 | Neurotrophic Receptor Tyrosine Kinase 2 | Protein Coding | Q16620 | 66 | GC09P084668 | 5.62243461608887 | https://www.genecards.org/cgi-bin/carddisp.pl?gene=NTRK2 |
| NPY2R | Neuropeptide Y Receptor Y2 | Protein Coding | P49146 | 55 | GC04P155173 | 5.60445976257324 | https://www.genecards.org/cgi-bin/carddisp.pl?gene=NPY2R |
| NEGR1 | Neuronal Growth Regulator 1 | Protein Coding | Q7Z3B1 | 50 | GC01M071395 | 5.55342483520508 | https://www.genecards.org/cgi-bin/carddisp.pl?gene=NEGR1 |
| KHDRBS1 | KH RNA Binding Domain Containing, Signal Transduction Associated 1 | Protein Coding | Q07666 | 54 | GC01P032013 | 5.48459959030151 | https://www.genecards.org/cgi-bin/carddisp.pl?gene=KHDRBS1 |
| CIDEC | Cell Death Inducing DFFA Like Effector C | Protein Coding | Q96AQ7 | 52 | GC03M009866 | 5.34551763534546 | https://www.genecards.org/cgi-bin/carddisp.pl?gene=CIDEC |
| APOB | Apolipoprotein B | Protein Coding | P04114 | 59 | GC02M020956 | 5.3403205871582 | https://www.genecards.org/cgi-bin/carddisp.pl?gene=APOB |
| GAL | Galanin And GMAP Prepropeptide | Protein Coding | P22466 | 57 | GC11P108040 | 5.33471012115479 | https://www.genecards.org/cgi-bin/carddisp.pl?gene=GAL |
| SOCS1 | Suppressor Of Cytokine Signaling 1 | Protein Coding | O15524 | 58 | GC16M050219 | 5.2843976020813 | https://www.genecards.org/cgi-bin/carddisp.pl?gene=SOCS1 |
| CD4 | CD4 Molecule | Protein Coding | P01730 | 63 | GC12P006786 | 5.25616598129272 | https://www.genecards.org/cgi-bin/carddisp.pl?gene=CD4 |
| SNRPN | Small Nuclear Ribonucleoprotein Polypeptide N | Protein Coding | P63162 | 57 | GC15P024823 | 5.25616598129272 | https://www.genecards.org/cgi-bin/carddisp.pl?gene=SNRPN |
| TMEM18 | Transmembrane Protein 18 | Protein Coding | Q96B42 | 46 | GC02M000660 | 5.25616598129272 | https://www.genecards.org/cgi-bin/carddisp.pl?gene=TMEM18 |
| RETNLB | Resistin Like Beta | Protein Coding | Q9BQ08 | 45 | GC03M108743 | 5.25616598129272 | https://www.genecards.org/cgi-bin/carddisp.pl?gene=RETNLB |
| MBOAT4 | Membrane Bound Ghrelin O-Acyltransferase MBOAT4 | Protein Coding | Q96T53 | 39 | GC08M030131 | 5.25616598129272 | https://www.genecards.org/cgi-bin/carddisp.pl?gene=MBOAT4 |
| SOCS2 | Suppressor Of Cytokine Signaling 2 | Protein Coding | O14508 | 56 | GC12P093569 | 5.16797542572021 | https://www.genecards.org/cgi-bin/carddisp.pl?gene=SOCS2 |
| HIF1A | Hypoxia Inducible Factor 1 Subunit Alpha | Protein Coding | Q16665 | 61 | GC14P061695 | 5.14820289611816 | https://www.genecards.org/cgi-bin/carddisp.pl?gene=HIF1A |
| PRKAA2 | Protein Kinase AMP-Activated Catalytic Subunit Alpha 2 | Protein Coding | P54646 | 61 | GC01P056645 | 5.11124038696289 | https://www.genecards.org/cgi-bin/carddisp.pl?gene=PRKAA2 |
| CNTF | Ciliary Neurotrophic Factor | Protein Coding | P26441 | 54 | GC11P058622 | 5.09436845779419 | https://www.genecards.org/cgi-bin/carddisp.pl?gene=CNTF |
| APOA1 | Apolipoprotein A1 | Protein Coding | P02647 | 63 | GC11M116835 | 5.08405590057373 | https://www.genecards.org/cgi-bin/carddisp.pl?gene=APOA1 |
| LMNB2 | Lamin B2 | Protein Coding | Q03252 | 55 | GC19M107830 | 4.99040937423706 | https://www.genecards.org/cgi-bin/carddisp.pl?gene=LMNB2 |
| ESR1 | Estrogen Receptor 1 | Protein Coding | P03372 | 67 | GC06P151656 | 4.94340896606445 | https://www.genecards.org/cgi-bin/carddisp.pl?gene=ESR1 |
| GHR | Growth Hormone Receptor | Protein Coding | P10912 | 58 | GC05P042429 | 4.90885639190674 | https://www.genecards.org/cgi-bin/carddisp.pl?gene=GHR |
| IRS4 | Insulin Receptor Substrate 4 | Protein Coding | O14654 | 54 | GC0XM108720 | 4.88196468353271 | https://www.genecards.org/cgi-bin/carddisp.pl?gene=IRS4 |
| STAT5B | Signal Transducer And Activator Of Transcription 5B | Protein Coding | P51692 | 62 | GC17M042199 | 4.87410163879395 | https://www.genecards.org/cgi-bin/carddisp.pl?gene=STAT5B |
| SIRT1 | Sirtuin 1 | Protein Coding | Q96EB6 | 62 | GC10P067884 | 4.81735610961914 | https://www.genecards.org/cgi-bin/carddisp.pl?gene=SIRT1 |
| CYP19A1 | Cytochrome P450 Family 19 Subfamily A Member 1 | Protein Coding | P11511 | 61 | GC15M051208 | 4.80641794204712 | https://www.genecards.org/cgi-bin/carddisp.pl?gene=CYP19A1 |
| ALB | Albumin | Protein Coding | P02768 | 61 | GC04P073397 | 4.74710655212402 | https://www.genecards.org/cgi-bin/carddisp.pl?gene=ALB |
| PIK3R1 | Phosphoinositide-3-Kinase Regulatory Subunit 1 | Protein Coding | P27986 | 65 | GC05P068215 | 4.72687244415283 | https://www.genecards.org/cgi-bin/carddisp.pl?gene=PIK3R1 |
| SST | Somatostatin | Protein Coding | P61278 | 54 | GC03M187668 | 4.72386932373047 | https://www.genecards.org/cgi-bin/carddisp.pl?gene=SST |
| PRKAA1 | Protein Kinase AMP-Activated Catalytic Subunit Alpha 1 | Protein Coding | Q13131 | 59 | GC05M040759 | 4.70790767669678 | https://www.genecards.org/cgi-bin/carddisp.pl?gene=PRKAA1 |
| AKT2 | AKT Serine/Threonine Kinase 2 | Protein Coding | P31751 | 67 | GC19M040230 | 4.70193767547607 | https://www.genecards.org/cgi-bin/carddisp.pl?gene=AKT2 |
| ACACA | Acetyl-CoA Carboxylase Alpha | Protein Coding | Q13085 | 62 | GC17M037084 | 4.65712738037109 | https://www.genecards.org/cgi-bin/carddisp.pl?gene=ACACA |
| IGFBP2 | Insulin Like Growth Factor Binding Protein 2 | Protein Coding | P18065 | 57 | GC02P216632 | 4.61988544464111 | https://www.genecards.org/cgi-bin/carddisp.pl?gene=IGFBP2 |
| ADRB2 | Adrenoceptor Beta 2 | Protein Coding | P07550 | 62 | GC05P159202 | 4.59092473983765 | https://www.genecards.org/cgi-bin/carddisp.pl?gene=ADRB2 |
| APOE | Apolipoprotein E | Protein Coding | P02649 | 62 | GC19P152697 | 4.58066606521606 | https://www.genecards.org/cgi-bin/carddisp.pl?gene=APOE |
| SOCS7 | Suppressor Of Cytokine Signaling 7 | Protein Coding | O14512 | 38 | GC17P154691 | 4.49550867080688 | https://www.genecards.org/cgi-bin/carddisp.pl?gene=SOCS7 |
| CERNA3 | Competing Endogenous LncRNA 3 For MiR-645 | RNA Gene |  | 21 | GC08P056444 | 4.48809623718262 | https://www.genecards.org/cgi-bin/carddisp.pl?gene=CERNA3 |
| TGFB1 | Transforming Growth Factor Beta 1 | Protein Coding | P01137 | 65 | GC19M041301 | 4.37988185882568 | https://www.genecards.org/cgi-bin/carddisp.pl?gene=TGFB1 |
| PIK3CA | Phosphatidylinositol-4,5-Bisphosphate 3-Kinase Catalytic Subunit Alpha | Protein Coding | P42336 | 65 | GC03P179148 | 4.3336009979248 | https://www.genecards.org/cgi-bin/carddisp.pl?gene=PIK3CA |
| NOS3 | Nitric Oxide Synthase 3 | Protein Coding | P29474 | 62 | GC07P165997 | 4.30389881134033 | https://www.genecards.org/cgi-bin/carddisp.pl?gene=NOS3 |
| CAV1 | Caveolin 1 | Protein Coding | Q03135 | 61 | GC07P116524 | 4.28898954391479 | https://www.genecards.org/cgi-bin/carddisp.pl?gene=CAV1 |
| HTR2C | 5-Hydroxytryptamine Receptor 2C | Protein Coding | P28335 | 57 | GC0XP114584 | 4.11941289901733 | https://www.genecards.org/cgi-bin/carddisp.pl?gene=HTR2C |
| PLIN1 | Perilipin 1 | Protein Coding | O60240 | 60 | GC15M089664 | 4.11526107788086 | https://www.genecards.org/cgi-bin/carddisp.pl?gene=PLIN1 |
| IGF2 | Insulin Like Growth Factor 2 | Protein Coding | P01344 | 61 | GC11M015123 | 4.08954238891602 | https://www.genecards.org/cgi-bin/carddisp.pl?gene=IGF2 |
| PTEN | Phosphatase And Tensin Homolog | Protein Coding | P60484 | 65 | GC10P124078 | 4.04794836044312 | https://www.genecards.org/cgi-bin/carddisp.pl?gene=PTEN |
| CETP | Cholesteryl Ester Transfer Protein | Protein Coding | P11597 | 60 | GC16P056961 | 4.03568696975708 | https://www.genecards.org/cgi-bin/carddisp.pl?gene=CETP |
| CCND1 | Cyclin D1 | Protein Coding | P24385 | 65 | GC11P069641 | 4.01730537414551 | https://www.genecards.org/cgi-bin/carddisp.pl?gene=CCND1 |
| TP53 | Tumor Protein P53 | Protein Coding | P04637 | 66 | GC17M007661 | 4.01009225845337 | https://www.genecards.org/cgi-bin/carddisp.pl?gene=TP53 |
| BANF1 | Barrier To Autointegration Nuclear Assembly Factor 1 | Protein Coding | O75531 | 55 | GC11P066002 | 3.99692749977112 | https://www.genecards.org/cgi-bin/carddisp.pl?gene=BANF1 |
| MTOR | Mechanistic Target Of Rapamycin Kinase | Protein Coding | P42345 | 68 | GC01M011106 | 3.96491050720215 | https://www.genecards.org/cgi-bin/carddisp.pl?gene=MTOR |
| PLEKHN1 | Pleckstrin Homology Domain Containing N1 | Protein Coding | Q494U1 | 41 | GC01P000966 | 3.92273306846619 | https://www.genecards.org/cgi-bin/carddisp.pl?gene=PLEKHN1 |
| LCAT | Lecithin-Cholesterol Acyltransferase | Protein Coding | P04180 | 60 | GC16M067939 | 3.90981602668762 | https://www.genecards.org/cgi-bin/carddisp.pl?gene=LCAT |
| C3 | Complement C3 | Protein Coding | P01024 | 62 | GC19M006677 | 3.88054609298706 | https://www.genecards.org/cgi-bin/carddisp.pl?gene=C3 |
| ERBB2 | Erb-B2 Receptor Tyrosine Kinase 2 | Protein Coding | P04626 | 68 | GC17P039687 | 3.86123466491699 | https://www.genecards.org/cgi-bin/carddisp.pl?gene=ERBB2 |
| CD36 | CD36 Molecule (CD36 Blood Group) | Protein Coding | P16671 | 63 | GC07P080369 | 3.85940885543823 | https://www.genecards.org/cgi-bin/carddisp.pl?gene=CD36 |
| MIR4443 | MicroRNA 4443 | RNA Gene |  | 12 | GC03P064945 | 3.85917997360229 | https://www.genecards.org/cgi-bin/carddisp.pl?gene=MIR4443 |
| EDN1 | Endothelin 1 | Protein Coding | P05305 | 61 | GC06P012910 | 3.83581829071045 | https://www.genecards.org/cgi-bin/carddisp.pl?gene=EDN1 |
| SERPINA12 | Serpin Family A Member 12 | Protein Coding | Q8IW75 | 51 | GC14M094487 | 3.82915639877319 | https://www.genecards.org/cgi-bin/carddisp.pl?gene=SERPINA12 |
| LIF | LIF Interleukin 6 Family Cytokine | Protein Coding | P15018 | 56 | GC22M030240 | 3.81346487998962 | https://www.genecards.org/cgi-bin/carddisp.pl?gene=LIF |
| ENPP1 | Ectonucleotide Pyrophosphatase/Phosphodiesterase 1 | Protein Coding | P22413 | 62 | GC06P131808 | 3.79426431655884 | https://www.genecards.org/cgi-bin/carddisp.pl?gene=ENPP1 |
| STAR | Steroidogenic Acute Regulatory Protein | Protein Coding | P49675 | 58 | GC08M038836 | 3.78019285202026 | https://www.genecards.org/cgi-bin/carddisp.pl?gene=STAR |
| MECP2 | Methyl-CpG Binding Protein 2 | Protein Coding | P51608 | 60 | GC0XM154021 | 3.77751755714417 | https://www.genecards.org/cgi-bin/carddisp.pl?gene=MECP2 |
| NUCB2 | Nucleobindin 2 | Protein Coding | P80303 | 50 | GC11P021358 | 3.75616312026978 | https://www.genecards.org/cgi-bin/carddisp.pl?gene=NUCB2 |
| PON1 | Paraoxonase 1 | Protein Coding | P27169 | 60 | GC07M095297 | 3.75183415412903 | https://www.genecards.org/cgi-bin/carddisp.pl?gene=PON1 |
| EGFR | Epidermal Growth Factor Receptor | Protein Coding | P00533 | 68 | GC07P055019 | 3.74932360649109 | https://www.genecards.org/cgi-bin/carddisp.pl?gene=EGFR |
| KCNJ6 | Potassium Inwardly Rectifying Channel Subfamily J Member 6 | Protein Coding | P48051 | 60 | GC21M037607 | 3.74834036827087 | https://www.genecards.org/cgi-bin/carddisp.pl?gene=KCNJ6 |
| LINC02605 | Long Intergenic Non-Protein Coding RNA 2605 | RNA Gene |  | 21 | GC08P078838 | 3.72860765457153 | https://www.genecards.org/cgi-bin/carddisp.pl?gene=LINC02605 |
| CAT | Catalase | Protein Coding | P04040 | 64 | GC11P034460 | 3.68528318405151 | https://www.genecards.org/cgi-bin/carddisp.pl?gene=CAT |
| TNFRSF1B | TNF Receptor Superfamily Member 1B | Protein Coding | P20333 | 61 | GC01P075466 | 3.65997624397278 | https://www.genecards.org/cgi-bin/carddisp.pl?gene=TNFRSF1B |
| LOC110386951 | CYP19A1 Promoter II/1.3 | Functional Element |  | 5 | GC15P193100 | 3.60327386856079 | https://www.genecards.org/cgi-bin/carddisp.pl?gene=LOC110386951 |
| SRC | SRC Proto-Oncogene, Non-Receptor Tyrosine Kinase | Protein Coding | P12931 | 64 | GC20P037344 | 3.59380960464478 | https://www.genecards.org/cgi-bin/carddisp.pl?gene=SRC |
| NOS2 | Nitric Oxide Synthase 2 | Protein Coding | P35228 | 62 | GC17M027756 | 3.54497909545898 | https://www.genecards.org/cgi-bin/carddisp.pl?gene=NOS2 |
| MAPK3 | Mitogen-Activated Protein Kinase 3 | Protein Coding | P27361 | 62 | GC16M051076 | 3.51904058456421 | https://www.genecards.org/cgi-bin/carddisp.pl?gene=MAPK3 |
| AR | Androgen Receptor | Protein Coding | P10275 | 65 | GC0XP067544 | 3.50283408164978 | https://www.genecards.org/cgi-bin/carddisp.pl?gene=AR |
| TTR | Transthyretin | Protein Coding | P02766 | 61 | GC18P031557 | 3.49053454399109 | https://www.genecards.org/cgi-bin/carddisp.pl?gene=TTR |
| FAAH | Fatty Acid Amide Hydrolase | Protein Coding | O00519 | 61 | GC01P046394 | 3.4691104888916 | https://www.genecards.org/cgi-bin/carddisp.pl?gene=FAAH |
| CGB5 | Chorionic Gonadotropin Subunit Beta 5 | Protein Coding | P0DN86 | 41 | GC19P049043 | 3.46803903579712 | https://www.genecards.org/cgi-bin/carddisp.pl?gene=CGB5 |
| PGR-AS1 | PGR Antisense RNA 1 | RNA Gene |  | 20 | GC11P108894 | 3.44696474075317 | https://www.genecards.org/cgi-bin/carddisp.pl?gene=PGR-AS1 |
| DGKZ | Diacylglycerol Kinase Zeta | Protein Coding | Q13574 | 56 | GC11P046332 | 3.42019367218018 | https://www.genecards.org/cgi-bin/carddisp.pl?gene=DGKZ |
| DRD2 | Dopamine Receptor D2 | Protein Coding | P14416 | 62 | GC11M113409 | 3.41023302078247 | https://www.genecards.org/cgi-bin/carddisp.pl?gene=DRD2 |
| CREB1 | CAMP Responsive Element Binding Protein 1 | Protein Coding | P16220 | 62 | GC02P207529 | 3.40049576759338 | https://www.genecards.org/cgi-bin/carddisp.pl?gene=CREB1 |
| CPT1A | Carnitine Palmitoyltransferase 1A | Protein Coding | P50416 | 61 | GC11M068754 | 3.39552068710327 | https://www.genecards.org/cgi-bin/carddisp.pl?gene=CPT1A |
| FABP4 | Fatty Acid Binding Protein 4 | Protein Coding | P15090 | 56 | GC08M081478 | 3.37632513046265 | https://www.genecards.org/cgi-bin/carddisp.pl?gene=FABP4 |
| VDR | Vitamin D Receptor | Protein Coding | P11473 | 61 | GC12M047841 | 3.37586617469788 | https://www.genecards.org/cgi-bin/carddisp.pl?gene=VDR |
| SCD | Stearoyl-CoA Desaturase | Protein Coding | O00767 | 61 | GC10P100347 | 3.35938405990601 | https://www.genecards.org/cgi-bin/carddisp.pl?gene=SCD |
| FNDC5 | Fibronectin Type III Domain Containing 5 | Protein Coding | Q8NAU1 | 47 | GC01M035669 | 3.33073663711548 | https://www.genecards.org/cgi-bin/carddisp.pl?gene=FNDC5 |
| GCKR | Glucokinase Regulator | Protein Coding | Q14397 | 52 | GC02P027496 | 3.31342935562134 | https://www.genecards.org/cgi-bin/carddisp.pl?gene=GCKR |
| SNORA1 | Small Nucleolar RNA, H/ACA Box 1 | RNA Gene |  | 24 | GC11M140304 | 3.31281781196594 | https://www.genecards.org/cgi-bin/carddisp.pl?gene=SNORA1 |
| SOD2 | Superoxide Dismutase 2 | Protein Coding | P04179 | 61 | GC06M159669 | 3.29764652252197 | https://www.genecards.org/cgi-bin/carddisp.pl?gene=SOD2 |
| ELAVL1 | ELAV Like RNA Binding Protein 1 | Protein Coding | Q15717 | 54 | GC19M007958 | 3.29457330703735 | https://www.genecards.org/cgi-bin/carddisp.pl?gene=ELAVL1 |
| IGF1R | Insulin Like Growth Factor 1 Receptor | Protein Coding | P08069 | 68 | GC15P098648 | 3.29193687438965 | https://www.genecards.org/cgi-bin/carddisp.pl?gene=IGF1R |
| STAT1 | Signal Transducer And Activator Of Transcription 1 | Protein Coding | P42224 | 65 | GC02M190908 | 3.29187798500061 | https://www.genecards.org/cgi-bin/carddisp.pl?gene=STAT1 |
| MKKS | MKKS Centrosomal Shuttling Protein | Protein Coding | Q9NPJ1 | 51 | GC20M010739 | 3.2827353477478 | https://www.genecards.org/cgi-bin/carddisp.pl?gene=MKKS |
| SOD2-OT1 | SOD2 Overlapping Transcript 1 | RNA Gene |  | 20 | GC06M159772 | 3.24211168289185 | https://www.genecards.org/cgi-bin/carddisp.pl?gene=SOD2-OT1 |
| MUC5AC | Mucin 5AC, Oligomeric Mucus/Gel-Forming | Protein Coding | P98088 | 51 | GC11P020281 | 3.23277568817139 | https://www.genecards.org/cgi-bin/carddisp.pl?gene=MUC5AC |
| IL1RN | Interleukin 1 Receptor Antagonist | Protein Coding | P18510 | 62 | GC02P154111 | 3.18897676467896 | https://www.genecards.org/cgi-bin/carddisp.pl?gene=IL1RN |
| PTGS2 | Prostaglandin-Endoperoxide Synthase 2 | Protein Coding | P35354 | 62 | GC01M186671 | 3.17578530311584 | https://www.genecards.org/cgi-bin/carddisp.pl?gene=PTGS2 |
| APLN | Apelin | Protein Coding | Q9ULZ1 | 48 | GC0XM129645 | 3.15900754928589 | https://www.genecards.org/cgi-bin/carddisp.pl?gene=APLN |
| PCSK9 | Proprotein Convertase Subtilisin/Kexin Type 9 | Protein Coding | Q8NBP7 | 62 | GC01P055039 | 3.14648103713989 | https://www.genecards.org/cgi-bin/carddisp.pl?gene=PCSK9 |
| PIK3CB | Phosphatidylinositol-4,5-Bisphosphate 3-Kinase Catalytic Subunit Beta | Protein Coding | P42338 | 61 | GC03M138652 | 3.11017107963562 | https://www.genecards.org/cgi-bin/carddisp.pl?gene=PIK3CB |
| MAPK8 | Mitogen-Activated Protein Kinase 8 | Protein Coding | P45983 | 62 | GC10P048306 | 3.10703611373901 | https://www.genecards.org/cgi-bin/carddisp.pl?gene=MAPK8 |
| SOD1 | Superoxide Dismutase 1 | Protein Coding | P00441 | 66 | GC21P031659 | 3.10559964179993 | https://www.genecards.org/cgi-bin/carddisp.pl?gene=SOD1 |
| SLC2A1 | Solute Carrier Family 2 Member 1 | Protein Coding | P11166 | 66 | GC01M042925 | 3.09477138519287 | https://www.genecards.org/cgi-bin/carddisp.pl?gene=SLC2A1 |
| SOAT1 | Sterol O-Acyltransferase 1 | Protein Coding | P35610 | 56 | GC01P179320 | 3.09331607818604 | https://www.genecards.org/cgi-bin/carddisp.pl?gene=SOAT1 |
| MMP9 | Matrix Metallopeptidase 9 | Protein Coding | P14780 | 66 | GC20P046008 | 3.08930349349976 | https://www.genecards.org/cgi-bin/carddisp.pl?gene=MMP9 |
| ABCA1 | ATP Binding Cassette Subfamily A Member 1 | Protein Coding | O95477 | 62 | GC09M104781 | 3.07630777359009 | https://www.genecards.org/cgi-bin/carddisp.pl?gene=ABCA1 |
| PTH | Parathyroid Hormone | Protein Coding | P01270 | 58 | GC11M013492 | 3.06679105758667 | https://www.genecards.org/cgi-bin/carddisp.pl?gene=PTH |
| CDKN3 | Cyclin Dependent Kinase Inhibitor 3 | Protein Coding | Q16667 | 54 | GC14P058243 | 3.06595230102539 | https://www.genecards.org/cgi-bin/carddisp.pl?gene=CDKN3 |
| IFNG | Interferon Gamma | Protein Coding | P01579 | 63 | GC12M068154 | 3.06390166282654 | https://www.genecards.org/cgi-bin/carddisp.pl?gene=IFNG |
| CFD | Complement Factor D | Protein Coding | P00746 | 57 | GC19P000859 | 3.04830551147461 | https://www.genecards.org/cgi-bin/carddisp.pl?gene=CFD |
| NGF | Nerve Growth Factor | Protein Coding | P01138 | 64 | GC01M115285 | 3.04752588272095 | https://www.genecards.org/cgi-bin/carddisp.pl?gene=NGF |
| IL18 | Interleukin 18 | Protein Coding | Q14116 | 57 | GC11M112143 | 3.04542756080627 | https://www.genecards.org/cgi-bin/carddisp.pl?gene=IL18 |
| EPO | Erythropoietin | Protein Coding | P01588 | 55 | GC07P100720 | 3.04037237167358 | https://www.genecards.org/cgi-bin/carddisp.pl?gene=EPO |
| GCK | Glucokinase | Protein Coding | P35557 | 62 | GC07M045688 | 3.03616237640381 | https://www.genecards.org/cgi-bin/carddisp.pl?gene=GCK |
| NR3C1 | Nuclear Receptor Subfamily 3 Group C Member 1 | Protein Coding | P04150 | 62 | GC05M143277 | 3.03538417816162 | https://www.genecards.org/cgi-bin/carddisp.pl?gene=NR3C1 |
| BCL2L1 | BCL2 Like 1 | Protein Coding | Q07817 | 62 | GC20M031664 | 3.03500151634216 | https://www.genecards.org/cgi-bin/carddisp.pl?gene=BCL2L1 |
| GALP | Galanin Like Peptide | Protein Coding | Q9UBC7 | 44 | GC19P056176 | 3.0328152179718 | https://www.genecards.org/cgi-bin/carddisp.pl?gene=GALP |
| GPD1 | Glycerol-3-Phosphate Dehydrogenase 1 | Protein Coding | P21695 | 56 | GC12P075094 | 3.02967667579651 | https://www.genecards.org/cgi-bin/carddisp.pl?gene=GPD1 |
| RAC1 | Rac Family Small GTPase 1 | Protein Coding | P63000 | 62 | GC07P019033 | 3.02856874465942 | https://www.genecards.org/cgi-bin/carddisp.pl?gene=RAC1 |
| FABP2 | Fatty Acid Binding Protein 2 | Protein Coding | P12104 | 54 | GC04M119317 | 3.02065300941467 | https://www.genecards.org/cgi-bin/carddisp.pl?gene=FABP2 |
| SERPINF1 | Serpin Family F Member 1 | Protein Coding | P36955 | 58 | GC17P153476 | 3.01713275909424 | https://www.genecards.org/cgi-bin/carddisp.pl?gene=SERPINF1 |
| IL2RA | Interleukin 2 Receptor Subunit Alpha | Protein Coding | P01589 | 63 | GC10M006010 | 3.01290011405945 | https://www.genecards.org/cgi-bin/carddisp.pl?gene=IL2RA |
| CISH | Cytokine Inducible SH2 Containing Protein | Protein Coding | Q9NSE2 | 58 | GC03M054843 | 3.00152587890625 | https://www.genecards.org/cgi-bin/carddisp.pl?gene=CISH |
| TIMP1 | TIMP Metallopeptidase Inhibitor 1 | Protein Coding | P01033 | 57 | GC0XP060812 | 2.98067355155945 | https://www.genecards.org/cgi-bin/carddisp.pl?gene=TIMP1 |
| NFKBIA | NFKB Inhibitor Alpha | Protein Coding | P25963 | 64 | GC14M035401 | 2.97459316253662 | https://www.genecards.org/cgi-bin/carddisp.pl?gene=NFKBIA |
| TJP3 | Tight Junction Protein 3 | Protein Coding | O95049 | 50 | GC19P003708 | 2.96851849555969 | https://www.genecards.org/cgi-bin/carddisp.pl?gene=TJP3 |
| MMP2 | Matrix Metallopeptidase 2 | Protein Coding | P08253 | 66 | GC16P121565 | 2.95546340942383 | https://www.genecards.org/cgi-bin/carddisp.pl?gene=MMP2 |
| MAPK14 | Mitogen-Activated Protein Kinase 14 | Protein Coding | Q16539 | 64 | GC06P182012 | 2.95355272293091 | https://www.genecards.org/cgi-bin/carddisp.pl?gene=MAPK14 |
| TNFSF11 | TNF Superfamily Member 11 | Protein Coding | O14788 | 62 | GC13P042562 | 2.94164180755615 | https://www.genecards.org/cgi-bin/carddisp.pl?gene=TNFSF11 |
| UGCG | UDP-Glucose Ceramide Glucosyltransferase | Protein Coding | Q16739 | 57 | GC09P111896 | 2.93406915664673 | https://www.genecards.org/cgi-bin/carddisp.pl?gene=UGCG |
| FFAR3 | Free Fatty Acid Receptor 3 | Protein Coding | O14843 | 48 | GC19P153610 | 2.92726612091064 | https://www.genecards.org/cgi-bin/carddisp.pl?gene=FFAR3 |
| RHOA | Ras Homolog Family Member A | Protein Coding | P61586 | 62 | GC03M049359 | 2.92274165153503 | https://www.genecards.org/cgi-bin/carddisp.pl?gene=RHOA |
| BBS2 | Bardet-Biedl Syndrome 2 | Protein Coding | Q9BXC9 | 53 | GC16M056467 | 2.92181539535522 | https://www.genecards.org/cgi-bin/carddisp.pl?gene=BBS2 |
| MT-TP | Mitochondrially Encoded TRNA-Pro (CCN) | RNA Gene |  | 19 | GCMTM015957 | 2.92003774642944 | https://www.genecards.org/cgi-bin/carddisp.pl?gene=MT-TP |
| NR5A1 | Nuclear Receptor Subfamily 5 Group A Member 1 | Protein Coding | Q13285 | 62 | GC09M124481 | 2.90373039245605 | https://www.genecards.org/cgi-bin/carddisp.pl?gene=NR5A1 |
| APOM | Apolipoprotein M | Protein Coding | O95445 | 51 | GC06P181900 | 2.89202189445496 | https://www.genecards.org/cgi-bin/carddisp.pl?gene=APOM |
| ICAM1 | Intercellular Adhesion Molecule 1 | Protein Coding | P05362 | 63 | GC19P151796 | 2.87718939781189 | https://www.genecards.org/cgi-bin/carddisp.pl?gene=ICAM1 |
| CYCS | Cytochrome C, Somatic | Protein Coding | P99999 | 61 | GC07M025118 | 2.87448835372925 | https://www.genecards.org/cgi-bin/carddisp.pl?gene=CYCS |
| AHSG | Alpha 2-HS Glycoprotein | Protein Coding | P02765 | 57 | GC03P186721 | 2.85646867752075 | https://www.genecards.org/cgi-bin/carddisp.pl?gene=AHSG |
| IL18R1 | Interleukin 18 Receptor 1 | Protein Coding | Q13478 | 56 | GC02P102311 | 2.85348868370056 | https://www.genecards.org/cgi-bin/carddisp.pl?gene=IL18R1 |
| PAPPA | Pappalysin 1 | Protein Coding | Q13219 | 55 | GC09P152394 | 2.85348868370056 | https://www.genecards.org/cgi-bin/carddisp.pl?gene=PAPPA |
| ZNF318 | Zinc Finger Protein 318 | Protein Coding | Q5VUA4 | 44 | GC06M106750 | 2.85281133651733 | https://www.genecards.org/cgi-bin/carddisp.pl?gene=ZNF318 |
| LBR | Lamin B Receptor | Protein Coding | Q14739 | 59 | GC01M225401 | 2.84345102310181 | https://www.genecards.org/cgi-bin/carddisp.pl?gene=LBR |
| SNX4 | Sorting Nexin 4 | Protein Coding | O95219 | 47 | GC03M125446 | 2.82953405380249 | https://www.genecards.org/cgi-bin/carddisp.pl?gene=SNX4 |
| SLC15A1 | Solute Carrier Family 15 Member 1 | Protein Coding | P46059 | 54 | GC13M098683 | 2.82910060882568 | https://www.genecards.org/cgi-bin/carddisp.pl?gene=SLC15A1 |
| SP1 | Sp1 Transcription Factor | Protein Coding | P08047 | 60 | GC12P053380 | 2.81106901168823 | https://www.genecards.org/cgi-bin/carddisp.pl?gene=SP1 |
| BCL2 | BCL2 Apoptosis Regulator | Protein Coding | P10415 | 64 | GC18M063123 | 2.80197978019714 | https://www.genecards.org/cgi-bin/carddisp.pl?gene=BCL2 |
| MMP13 | Matrix Metallopeptidase 13 | Protein Coding | P45452 | 62 | GC11M102942 | 2.79004716873169 | https://www.genecards.org/cgi-bin/carddisp.pl?gene=MMP13 |
| NPY5R | Neuropeptide Y Receptor Y5 | Protein Coding | Q15761 | 55 | GC04P163343 | 2.7819836139679 | https://www.genecards.org/cgi-bin/carddisp.pl?gene=NPY5R |
| FOXO1 | Forkhead Box O1 | Protein Coding | Q12778 | 64 | GC13M040555 | 2.77794289588928 | https://www.genecards.org/cgi-bin/carddisp.pl?gene=FOXO1 |
| ANGPTL4 | Angiopoietin Like 4 | Protein Coding | Q9BY76 | 56 | GC19P008363 | 2.75883483886719 | https://www.genecards.org/cgi-bin/carddisp.pl?gene=ANGPTL4 |
| TNFRSF1A | TNF Receptor Superfamily Member 1A | Protein Coding | P19438 | 62 | GC12M006328 | 2.73978614807129 | https://www.genecards.org/cgi-bin/carddisp.pl?gene=TNFRSF1A |
| FASN | Fatty Acid Synthase | Protein Coding | P49327 | 61 | GC17M082078 | 2.73513412475586 | https://www.genecards.org/cgi-bin/carddisp.pl?gene=FASN |
| SLC9A1 | Solute Carrier Family 9 Member A1 | Protein Coding | P19634 | 63 | GC01M034801 | 2.73180651664734 | https://www.genecards.org/cgi-bin/carddisp.pl?gene=SLC9A1 |
| PCNA | Proliferating Cell Nuclear Antigen | Protein Coding | P12004 | 63 | GC20M005114 | 2.71675634384155 | https://www.genecards.org/cgi-bin/carddisp.pl?gene=PCNA |
| GAPDH | Glyceraldehyde-3-Phosphate Dehydrogenase | Protein Coding | P04406 | 64 | GC12P074057 | 2.70776963233948 | https://www.genecards.org/cgi-bin/carddisp.pl?gene=GAPDH |
| PGR | Progesterone Receptor | Protein Coding | P06401 | 62 | GC11M140371 | 2.70123839378357 | https://www.genecards.org/cgi-bin/carddisp.pl?gene=PGR |
| CYP11A1 | Cytochrome P450 Family 11 Subfamily A Member 1 | Protein Coding | P05108 | 62 | GC15M074337 | 2.69829344749451 | https://www.genecards.org/cgi-bin/carddisp.pl?gene=CYP11A1 |
| JUN | Jun Proto-Oncogene, AP-1 Transcription Factor Subunit | Protein Coding | P05412 | 62 | GC01M058780 | 2.69548583030701 | https://www.genecards.org/cgi-bin/carddisp.pl?gene=JUN |
| CLU | Clusterin | Protein Coding | P10909 | 60 | GC08M027596 | 2.69025897979736 | https://www.genecards.org/cgi-bin/carddisp.pl?gene=CLU |
| CSF3 | Colony Stimulating Factor 3 | Protein Coding | P09919 | 55 | GC17P040015 | 2.67412161827087 | https://www.genecards.org/cgi-bin/carddisp.pl?gene=CSF3 |
| TGFBR2 | Transforming Growth Factor Beta Receptor 2 | Protein Coding | P37173 | 65 | GC03P030609 | 2.64980554580688 | https://www.genecards.org/cgi-bin/carddisp.pl?gene=TGFBR2 |
| ADM | Adrenomedullin | Protein Coding | P35318 | 57 | GC11P021130 | 2.64458608627319 | https://www.genecards.org/cgi-bin/carddisp.pl?gene=ADM |
| APOA4 | Apolipoprotein A4 | Protein Coding | P06727 | 55 | GC11M116820 | 2.6374192237854 | https://www.genecards.org/cgi-bin/carddisp.pl?gene=APOA4 |
| APOC3 | Apolipoprotein C3 | Protein Coding | P02656 | 58 | GC11P116829 | 2.63602161407471 | https://www.genecards.org/cgi-bin/carddisp.pl?gene=APOC3 |
| KISS1R | KISS1 Receptor | Protein Coding | Q969F8 | 57 | GC19P000917 | 2.60447669029236 | https://www.genecards.org/cgi-bin/carddisp.pl?gene=KISS1R |
| INHBB | Inhibin Subunit Beta B | Protein Coding | P09529 | 53 | GC02P154212 | 2.58940625190735 | https://www.genecards.org/cgi-bin/carddisp.pl?gene=INHBB |
| USP8 | Ubiquitin Specific Peptidase 8 | Protein Coding | P40818 | 61 | GC15P050424 | 2.58104062080383 | https://www.genecards.org/cgi-bin/carddisp.pl?gene=USP8 |
| LDLR | Low Density Lipoprotein Receptor | Protein Coding | P01130 | 64 | GC19P151814 | 2.57788252830505 | https://www.genecards.org/cgi-bin/carddisp.pl?gene=LDLR |
| IL2RB | Interleukin 2 Receptor Subunit Beta | Protein Coding | P14784 | 62 | GC22M086125 | 2.57153153419495 | https://www.genecards.org/cgi-bin/carddisp.pl?gene=IL2RB |
| HSD11B1 | Hydroxysteroid 11-Beta Dehydrogenase 1 | Protein Coding | P28845 | 63 | GC01P209686 | 2.57109880447388 | https://www.genecards.org/cgi-bin/carddisp.pl?gene=HSD11B1 |
| IGFBP4 | Insulin Like Growth Factor Binding Protein 4 | Protein Coding | P22692 | 56 | GC17P040443 | 2.56863117218018 | https://www.genecards.org/cgi-bin/carddisp.pl?gene=IGFBP4 |
| UCN | Urocortin | Protein Coding | P55089 | 48 | GC02M027308 | 2.56562519073486 | https://www.genecards.org/cgi-bin/carddisp.pl?gene=UCN |
| GGT1 | Gamma-Glutamyltransferase 1 | Protein Coding | P19440 | 62 | GC22P024583 | 2.55560326576233 | https://www.genecards.org/cgi-bin/carddisp.pl?gene=GGT1 |
| LOC126805877 | MED14-Independent Group 3 Enhancer GRCh37_chr1:156099693-156100892 | Functional Element |  | 11 | GC01P176810 | 2.55334663391113 | https://www.genecards.org/cgi-bin/carddisp.pl?gene=LOC126805877 |
| FAS | Fas Cell Surface Death Receptor | Protein Coding | P25445 | 64 | GC10P124091 | 2.55118799209595 | https://www.genecards.org/cgi-bin/carddisp.pl?gene=FAS |
| ARHGAP35 | Rho GTPase Activating Protein 35 | Protein Coding | Q9NRY4 | 51 | GC19P046860 | 2.55118799209595 | https://www.genecards.org/cgi-bin/carddisp.pl?gene=ARHGAP35 |
| TF | Transferrin | Protein Coding | P02787 | 64 | GC03P143239 | 2.54783082008362 | https://www.genecards.org/cgi-bin/carddisp.pl?gene=TF |
| FABP3 | Fatty Acid Binding Protein 3 | Protein Coding | P05413 | 57 | GC01M034928 | 2.53510355949402 | https://www.genecards.org/cgi-bin/carddisp.pl?gene=FABP3 |
| HCRTR1 | Hypocretin Receptor 1 | Protein Coding | O43613 | 55 | GC01P031617 | 2.53447103500366 | https://www.genecards.org/cgi-bin/carddisp.pl?gene=HCRTR1 |
| FLT1 | Fms Related Receptor Tyrosine Kinase 1 | Protein Coding | P17948 | 64 | GC13M028300 | 2.52360677719116 | https://www.genecards.org/cgi-bin/carddisp.pl?gene=FLT1 |
| NCOA1 | Nuclear Receptor Coactivator 1 | Protein Coding | Q15788 | 58 | GC02P024540 | 2.51563739776611 | https://www.genecards.org/cgi-bin/carddisp.pl?gene=NCOA1 |
| CGA | Glycoprotein Hormones, Alpha Polypeptide | Protein Coding | P01215 | 57 | GC06M087085 | 2.51563739776611 | https://www.genecards.org/cgi-bin/carddisp.pl?gene=CGA |
| SELE | Selectin E | Protein Coding | P16581 | 57 | GC01M169722 | 2.51132321357727 | https://www.genecards.org/cgi-bin/carddisp.pl?gene=SELE |
| MAP2K1 | Mitogen-Activated Protein Kinase Kinase 1 | Protein Coding | Q02750 | 67 | GC15P066386 | 2.50182604789734 | https://www.genecards.org/cgi-bin/carddisp.pl?gene=MAP2K1 |
| CCKAR | Cholecystokinin A Receptor | Protein Coding | P32238 | 58 | GC04M026483 | 2.50120425224304 | https://www.genecards.org/cgi-bin/carddisp.pl?gene=CCKAR |
| ACE | Angiotensin I Converting Enzyme | Protein Coding | P12821 | 64 | GC17P063477 | 2.48930335044861 | https://www.genecards.org/cgi-bin/carddisp.pl?gene=ACE |
| HGF | Hepatocyte Growth Factor | Protein Coding | P14210 | 65 | GC07M081699 | 2.48543000221252 | https://www.genecards.org/cgi-bin/carddisp.pl?gene=HGF |
| C1QTNF3 | C1q And TNF Related 3 | Protein Coding | Q9BXJ4 | 47 | GC05M034017 | 2.47383499145508 | https://www.genecards.org/cgi-bin/carddisp.pl?gene=C1QTNF3 |
| MIR7-3HG | MIR7-3 Host Gene | RNA Gene | Q8N6C7 | 34 | GC19P151538 | 2.46818065643311 | https://www.genecards.org/cgi-bin/carddisp.pl?gene=MIR7-3HG |
| CYP17A1 | Cytochrome P450 Family 17 Subfamily A Member 1 | Protein Coding | P05093 | 62 | GC10M102830 | 2.45871567726135 | https://www.genecards.org/cgi-bin/carddisp.pl?gene=CYP17A1 |
| LPA | Lipoprotein(A) | Protein Coding | P08519 | 55 | GC06M160531 | 2.45607614517212 | https://www.genecards.org/cgi-bin/carddisp.pl?gene=LPA |
| IGFBP6 | Insulin Like Growth Factor Binding Protein 6 | Protein Coding | P24592 | 56 | GC12P053097 | 2.4434986114502 | https://www.genecards.org/cgi-bin/carddisp.pl?gene=IGFBP6 |
| CASP3 | Caspase 3 | Protein Coding | P42574 | 62 | GC04M184627 | 2.431321144 | https://www.genecards.org/cgi-bin/carddisp.pl?gene=CASP3 |
| MMP14 | Matrix Metallopeptidase 14 | Protein Coding | P50281 | 64 | GC14P057548 | 2.40577983856201 | https://www.genecards.org/cgi-bin/carddisp.pl?gene=MMP14 |
| LCN2 | Lipocalin 2 | Protein Coding | P80188 | 58 | GC09P128149 | 2.40187978744507 | https://www.genecards.org/cgi-bin/carddisp.pl?gene=LCN2 |
| ENG | Endoglin | Protein Coding | P17813 | 60 | GC09M131710 | 2.40040183067322 | https://www.genecards.org/cgi-bin/carddisp.pl?gene=ENG |
| PLTP | Phospholipid Transfer Protein | Protein Coding | P55058 | 57 | GC20M045898 | 2.39658808708191 | https://www.genecards.org/cgi-bin/carddisp.pl?gene=PLTP |
| GSK3B | Glycogen Synthase Kinase 3 Beta | Protein Coding | P49841 | 64 | GC03M119821 | 2.37937164306641 | https://www.genecards.org/cgi-bin/carddisp.pl?gene=GSK3B |
| PLAT | Plasminogen Activator, Tissue Type | Protein Coding | P00750 | 62 | GC08M042174 | 2.37937164306641 | https://www.genecards.org/cgi-bin/carddisp.pl?gene=PLAT |
| MUC5B | Mucin 5B, Oligomeric Mucus/Gel-Forming | Protein Coding | Q9HC84 | 54 | GC11P020284 | 2.37937164306641 | https://www.genecards.org/cgi-bin/carddisp.pl?gene=MUC5B |
| VIP | Vasoactive Intestinal Peptide | Protein Coding | P01282 | 57 | GC06P152750 | 2.3707332611084 | https://www.genecards.org/cgi-bin/carddisp.pl?gene=VIP |
| CASP1 | Caspase 1 | Protein Coding | P29466 | 61 | GC11M105025 | 2.3658139705658 | https://www.genecards.org/cgi-bin/carddisp.pl?gene=CASP1 |
| EGF | Epidermal Growth Factor | Protein Coding | P01133 | 64 | GC04P109912 | 2.35200262069702 | https://www.genecards.org/cgi-bin/carddisp.pl?gene=EGF |
| MIR34A | MicroRNA 34a | RNA Gene |  | 31 | GC01M009151 | 2.35200262069702 | https://www.genecards.org/cgi-bin/carddisp.pl?gene=MIR34A |
| IL4 | Interleukin 4 | Protein Coding | P05112 | 59 | GC05P132673 | 2.34711313247681 | https://www.genecards.org/cgi-bin/carddisp.pl?gene=IL4 |
| PLCG2 | Phospholipase C Gamma 2 | Protein Coding | P16885 | 65 | GC16P122357 | 2.34042882919312 | https://www.genecards.org/cgi-bin/carddisp.pl?gene=PLCG2 |
| RUNX2 | RUNX Family Transcription Factor 2 | Protein Coding | Q13950 | 60 | GC06P182087 | 2.33792304992676 | https://www.genecards.org/cgi-bin/carddisp.pl?gene=RUNX2 |
| IL2 | Interleukin 2 | Protein Coding | P60568 | 58 | GC04M122451 | 2.33792304992676 | https://www.genecards.org/cgi-bin/carddisp.pl?gene=IL2 |
| GALT | Galactose-1-Phosphate Uridylyltransferase | Protein Coding | P07902 | 59 | GC09P074560 | 2.33165168762207 | https://www.genecards.org/cgi-bin/carddisp.pl?gene=GALT |
| GRB2 | Growth Factor Receptor Bound Protein 2 | Protein Coding | P62993 | 60 | GC17M075318 | 2.32749557495117 | https://www.genecards.org/cgi-bin/carddisp.pl?gene=GRB2 |
| ACACB | Acetyl-CoA Carboxylase Beta | Protein Coding | O00763 | 58 | GC12P109116 | 2.3264148235321 | https://www.genecards.org/cgi-bin/carddisp.pl?gene=ACACB |
| MAT2A | Methionine Adenosyltransferase 2A | Protein Coding | P31153 | 59 | GC02P094481 | 2.32512044906616 | https://www.genecards.org/cgi-bin/carddisp.pl?gene=MAT2A |
| TRE-TTC3-1 | TRNA-Glu (Anticodon TTC) 3-1 | RNA Gene |  | 17 | GC01P075884 | 2.31159591674805 | https://www.genecards.org/cgi-bin/carddisp.pl?gene=TRE-TTC3-1 |
| SPP1 | Secreted Phosphoprotein 1 | Protein Coding | P10451 | 57 | GC04P087975 | 2.31156253814697 | https://www.genecards.org/cgi-bin/carddisp.pl?gene=SPP1 |
| IL1RAPL2 | Interleukin 1 Receptor Accessory Protein Like 2 | Protein Coding | Q9NP60 | 51 | GC0XP104566 | 2.2939031124115 | https://www.genecards.org/cgi-bin/carddisp.pl?gene=IL1RAPL2 |
| AGTR1 | Angiotensin II Receptor Type 1 | Protein Coding | P30556 | 65 | GC03P148697 | 2.29288721084595 | https://www.genecards.org/cgi-bin/carddisp.pl?gene=AGTR1 |
| CYP27A1 | Cytochrome P450 Family 27 Subfamily A Member 1 | Protein Coding | Q02318 | 60 | GC02P218781 | 2.28062391281128 | https://www.genecards.org/cgi-bin/carddisp.pl?gene=CYP27A1 |
| CXCL10 | C-X-C Motif Chemokine Ligand 10 | Protein Coding | P02778 | 57 | GC04M076021 | 2.27538275718689 | https://www.genecards.org/cgi-bin/carddisp.pl?gene=CXCL10 |
| DEFB4A | Defensin Beta 4A | Protein Coding | O15263 | 48 | GC08P011308 | 2.25494718551636 | https://www.genecards.org/cgi-bin/carddisp.pl?gene=DEFB4A |
| SAA1 | Serum Amyloid A1 | Protein Coding | P0DJI8 | 51 | GC11P021404 | 2.25464081764221 | https://www.genecards.org/cgi-bin/carddisp.pl?gene=SAA1 |
| MUC1 | Mucin 1, Cell Surface Associated | Protein Coding | P15941 | 61 | GC01M155185 | 2.24676465988159 | https://www.genecards.org/cgi-bin/carddisp.pl?gene=MUC1 |
| VWF | Von Willebrand Factor | Protein Coding | P04275 | 61 | GC12M035631 | 2.24138927459717 | https://www.genecards.org/cgi-bin/carddisp.pl?gene=VWF |
| NR3C2 | Nuclear Receptor Subfamily 3 Group C Member 2 | Protein Coding | P08235 | 59 | GC04M148078 | 2.23942375183105 | https://www.genecards.org/cgi-bin/carddisp.pl?gene=NR3C2 |
| JAK1 | Janus Kinase 1 | Protein Coding | P23458 | 66 | GC01M064833 | 2.23023366928101 | https://www.genecards.org/cgi-bin/carddisp.pl?gene=JAK1 |
| MIR21 | MicroRNA 21 | RNA Gene |  | 33 | GC17P059841 | 2.22907900810242 | https://www.genecards.org/cgi-bin/carddisp.pl?gene=MIR21 |
| NOTCH1 | Notch Receptor 1 | Protein Coding | P46531 | 66 | GC09M139264 | 2.22757792472839 | https://www.genecards.org/cgi-bin/carddisp.pl?gene=NOTCH1 |
| SLC17A5 | Solute Carrier Family 17 Member 5 | Protein Coding | Q9NRA2 | 58 | GC06M073593 | 2.22757792472839 | https://www.genecards.org/cgi-bin/carddisp.pl?gene=SLC17A5 |
| STK11 | Serine/Threonine Kinase 11 | Protein Coding | Q15831 | 62 | GC19P001177 | 2.22724628448486 | https://www.genecards.org/cgi-bin/carddisp.pl?gene=STK11 |
| HTR2A | 5-Hydroxytryptamine Receptor 2A | Protein Coding | P28223 | 61 | GC13M046831 | 2.22431778907776 | https://www.genecards.org/cgi-bin/carddisp.pl?gene=HTR2A |
| THBD | Thrombomodulin | Protein Coding | P07204 | 58 | GC20M023026 | 2.22431778907776 | https://www.genecards.org/cgi-bin/carddisp.pl?gene=THBD |
| VCAM1 | Vascular Cell Adhesion Molecule 1 | Protein Coding | P19320 | 58 | GC01P100719 | 2.20861434936523 | https://www.genecards.org/cgi-bin/carddisp.pl?gene=VCAM1 |
| GAST | Gastrin | Protein Coding | P01350 | 52 | GC17P041712 | 2.20493674278259 | https://www.genecards.org/cgi-bin/carddisp.pl?gene=GAST |
| IL6R | Interleukin 6 Receptor | Protein Coding | P08887 | 63 | GC01P154405 | 2.19913411140442 | https://www.genecards.org/cgi-bin/carddisp.pl?gene=IL6R |
| SLC5A1 | Solute Carrier Family 5 Member 1 | Protein Coding | P13866 | 60 | GC22P032043 | 2.19913411140442 | https://www.genecards.org/cgi-bin/carddisp.pl?gene=SLC5A1 |
| PCK2 | Phosphoenolpyruvate Carboxykinase 2, Mitochondrial | Protein Coding | Q16822 | 58 | GC14P024094 | 2.19913411140442 | https://www.genecards.org/cgi-bin/carddisp.pl?gene=PCK2 |
| PNPLA3 | Patatin Like Domain 3, 1-Acylglycerol-3-Phosphate O-Acyltransferase | Protein Coding | Q9NST1 | 54 | GC22P043923 | 2.19426465034485 | https://www.genecards.org/cgi-bin/carddisp.pl?gene=PNPLA3 |
| SERPINA1 | Serpin Family A Member 1 | Protein Coding | P01009 | 62 | GC14M094376 | 2.18861484527588 | https://www.genecards.org/cgi-bin/carddisp.pl?gene=SERPINA1 |
| NTS | Neurotensin | Protein Coding | P30990 | 53 | GC12P085876 | 2.18861484527588 | https://www.genecards.org/cgi-bin/carddisp.pl?gene=NTS |
| IL13 | Interleukin 13 | Protein Coding | P35225 | 57 | GC05P132656 | 2.18446755409241 | https://www.genecards.org/cgi-bin/carddisp.pl?gene=IL13 |
| PDE3B | Phosphodiesterase 3B | Protein Coding | Q13370 | 57 | GC11P014643 | 2.18408942222595 | https://www.genecards.org/cgi-bin/carddisp.pl?gene=PDE3B |
| FST | Follistatin | Protein Coding | P19883 | 61 | GC05P053480 | 2.17767190933228 | https://www.genecards.org/cgi-bin/carddisp.pl?gene=FST |
| BAX | BCL2 Associated X, Apoptosis Regulator | Protein Coding | Q07812 | 64 | GC19P048954 | 2.17529678344727 | https://www.genecards.org/cgi-bin/carddisp.pl?gene=BAX |
| ROCK1 | Rho Associated Coiled-Coil Containing Protein Kinase 1 | Protein Coding | Q13464 | 61 | GC18M038236 | 2.1696457862854 | https://www.genecards.org/cgi-bin/carddisp.pl?gene=ROCK1 |
| FGF19 | Fibroblast Growth Factor 19 | Protein Coding | O95750 | 57 | GC11M139938 | 2.16818881034851 | https://www.genecards.org/cgi-bin/carddisp.pl?gene=FGF19 |
| PDPK1 | 3-Phosphoinositide Dependent Protein Kinase 1 | Protein Coding | O15530 | 61 | GC16P002537 | 2.16291403770447 | https://www.genecards.org/cgi-bin/carddisp.pl?gene=PDPK1 |
| PIK3R3 | Phosphoinositide-3-Kinase Regulatory Subunit 3 | Protein Coding | Q92569 | 55 | GC01M046041 | 2.16291403770447 | https://www.genecards.org/cgi-bin/carddisp.pl?gene=PIK3R3 |
| IKZF1 | IKAROS Family Zinc Finger 1 | Protein Coding | Q13422 | 61 | GC07P050303 | 2.15626668930054 | https://www.genecards.org/cgi-bin/carddisp.pl?gene=IKZF1 |
| BBS9 | Bardet-Biedl Syndrome 9 | Protein Coding | Q3SYG4 | 52 | GC07P034291 | 2.14883589744568 | https://www.genecards.org/cgi-bin/carddisp.pl?gene=BBS9 |
| LIFR | LIF Receptor Subunit Alpha | Protein Coding | P42702 | 60 | GC05M038827 | 2.14792776107788 | https://www.genecards.org/cgi-bin/carddisp.pl?gene=LIFR |
| GRIN2A | Glutamate Ionotropic Receptor NMDA Type Subunit 2A | Protein Coding | Q12879 | 65 | GC16M009753 | 2.13690400123596 | https://www.genecards.org/cgi-bin/carddisp.pl?gene=GRIN2A |
| GRIN2B | Glutamate Ionotropic Receptor NMDA Type Subunit 2B | Protein Coding | Q13224 | 65 | GC12M013437 | 2.13690400123596 | https://www.genecards.org/cgi-bin/carddisp.pl?gene=GRIN2B |
| FGF7 | Fibroblast Growth Factor 7 | Protein Coding | P21781 | 57 | GC15P049423 | 2.13384819030762 | https://www.genecards.org/cgi-bin/carddisp.pl?gene=FGF7 |
| ZDHHC24 | Zinc Finger DHHC-Type Containing 24 | Protein Coding | Q6UX98 | 44 | GC11M139833 | 2.12872314453125 | https://www.genecards.org/cgi-bin/carddisp.pl?gene=ZDHHC24 |
| GHRHR | Growth Hormone Releasing Hormone Receptor | Protein Coding | Q02643 | 55 | GC07P030938 | 2.12233996391296 | https://www.genecards.org/cgi-bin/carddisp.pl?gene=GHRHR |
| CCL3 | C-C Motif Chemokine Ligand 3 | Protein Coding | P10147 | 54 | GC17M036088 | 2.12233996391296 | https://www.genecards.org/cgi-bin/carddisp.pl?gene=CCL3 |
| BRCA1 | BRCA1 DNA Repair Associated | Protein Coding | P38398 | 63 | GC17M043044 | 2.10580921173096 | https://www.genecards.org/cgi-bin/carddisp.pl?gene=BRCA1 |
| CD69 | CD69 Molecule | Protein Coding | Q07108 | 54 | GC12M035789 | 2.10481739044189 | https://www.genecards.org/cgi-bin/carddisp.pl?gene=CD69 |
| NPY1R | Neuropeptide Y Receptor Y1 | Protein Coding | P25929 | 57 | GC04M163323 | 2.10474729537964 | https://www.genecards.org/cgi-bin/carddisp.pl?gene=NPY1R |
| GRIA1 | Glutamate Ionotropic Receptor AMPA Type Subunit 1 | Protein Coding | P42261 | 62 | GC05P153489 | 2.09398746490479 | https://www.genecards.org/cgi-bin/carddisp.pl?gene=GRIA1 |
| PNPLA2 | Patatin Like Domain 2, Triacylglycerol Lipase | Protein Coding | Q96AD5 | 57 | GC11P020269 | 2.09398746490479 | https://www.genecards.org/cgi-bin/carddisp.pl?gene=PNPLA2 |
| RNU1-4 | RNA, U1 Small Nuclear 4 | RNA Gene |  | 14 | GC01P016740 | 2.08881235122681 | https://www.genecards.org/cgi-bin/carddisp.pl?gene=RNU1-4 |
| CEBPA | CCAAT Enhancer Binding Protein Alpha | Protein Coding | P49715 | 60 | GC19M033299 | 2.08014416694641 | https://www.genecards.org/cgi-bin/carddisp.pl?gene=CEBPA |
| FOXO3 | Forkhead Box O3 | Protein Coding | O43524 | 60 | GC06P108559 | 2.07449436187744 | https://www.genecards.org/cgi-bin/carddisp.pl?gene=FOXO3 |
| SULT1A3 | Sulfotransferase Family 1A Member 3 | Protein Coding | P0DMM9 | 47 | GC16P030199 | 2.07130789756775 | https://www.genecards.org/cgi-bin/carddisp.pl?gene=SULT1A3 |
| PRKAG1 | Protein Kinase AMP-Activated Non-Catalytic Subunit Gamma 1 | Protein Coding | P54619 | 57 | GC12M049002 | 2.06693458557129 | https://www.genecards.org/cgi-bin/carddisp.pl?gene=PRKAG1 |
| ITGAV | Integrin Subunit Alpha V | Protein Coding | P06756 | 61 | GC02P186589 | 2.05324745178223 | https://www.genecards.org/cgi-bin/carddisp.pl?gene=ITGAV |
| IRAK1 | Interleukin 1 Receptor Associated Kinase 1 | Protein Coding | P51617 | 61 | GC0XM154010 | 2.04651713371277 | https://www.genecards.org/cgi-bin/carddisp.pl?gene=IRAK1 |
| ESR2 | Estrogen Receptor 2 | Protein Coding | Q92731 | 61 | GC14M064084 | 2.04268980026245 | https://www.genecards.org/cgi-bin/carddisp.pl?gene=ESR2 |
| FGR | FGR Proto-Oncogene, Src Family Tyrosine Kinase | Protein Coding | P09769 | 60 | GC01M034833 | 2.04268980026245 | https://www.genecards.org/cgi-bin/carddisp.pl?gene=FGR |
| MUC16 | Mucin 16, Cell Surface Associated | Protein Coding | Q8WXI7 | 51 | GC19M008848 | 2.04268980026245 | https://www.genecards.org/cgi-bin/carddisp.pl?gene=MUC16 |
| LOC110806263 | TERT 5' Regulatory Region | Functional Element |  | 10 | GC05P001294 | 2.04268980026245 | https://www.genecards.org/cgi-bin/carddisp.pl?gene=LOC110806263 |
| CGB3 | Chorionic Gonadotropin Subunit Beta 3 | Protein Coding | P0DN86 | 45 | GC19M109024 | 2.03936815261841 | https://www.genecards.org/cgi-bin/carddisp.pl?gene=CGB3 |
| TFAP2A | Transcription Factor AP-2 Alpha | Protein Coding | P05549 | 60 | GC06M010393 | 2.03180885314941 | https://www.genecards.org/cgi-bin/carddisp.pl?gene=TFAP2A |
| B2M | Beta-2-Microglobulin | Protein Coding | P61769 | 62 | GC15P044711 | 2.02500414848328 | https://www.genecards.org/cgi-bin/carddisp.pl?gene=B2M |
| HSPG2 | Heparan Sulfate Proteoglycan 2 | Protein Coding | P98160 | 61 | GC01M021822 | 2.02500414848328 | https://www.genecards.org/cgi-bin/carddisp.pl?gene=HSPG2 |
| NOX5 | NADPH Oxidase 5 | Protein Coding | Q96PH1 | 48 | GC15P193382 | 2.02128791809082 | https://www.genecards.org/cgi-bin/carddisp.pl?gene=NOX5 |
| IL1A | Interleukin 1 Alpha | Protein Coding | P01583 | 58 | GC02M112773 | 2.01539945602417 | https://www.genecards.org/cgi-bin/carddisp.pl?gene=IL1A |
| LIPC | Lipase C, Hepatic Type | Protein Coding | P11150 | 59 | GC15P058410 | 2.015221119 | https://www.genecards.org/cgi-bin/carddisp.pl?gene=LIPC |
| MAPK10 | Mitogen-Activated Protein Kinase 10 | Protein Coding | P53779 | 60 | GC04M085990 | 2.01033735275269 | https://www.genecards.org/cgi-bin/carddisp.pl?gene=MAPK10 |
| MTA1 | Metastasis Associated 1 | Protein Coding | Q13330 | 54 | GC14P105419 | 2.01033735275269 | https://www.genecards.org/cgi-bin/carddisp.pl?gene=MTA1 |
| KCNJ5 | Potassium Inwardly Rectifying Channel Subfamily J Member 5 | Protein Coding | P48544 | 61 | GC11P128891 | 2.00916218757629 | https://www.genecards.org/cgi-bin/carddisp.pl?gene=KCNJ5 |
| RXRA | Retinoid X Receptor Alpha | Protein Coding | P19793 | 61 | GC09P134317 | 1.99534833431244 | https://www.genecards.org/cgi-bin/carddisp.pl?gene=RXRA |
| NPY4R | Neuropeptide Y Receptor Y4 | Protein Coding | P50391 | 52 | GC10M046461 | 1.99534833431244 | https://www.genecards.org/cgi-bin/carddisp.pl?gene=NPY4R |
| OMA1 | OMA1 Zinc Metallopeptidase | Protein Coding | Q96E52 | 48 | GC01M058415 | 1.99534833431244 | https://www.genecards.org/cgi-bin/carddisp.pl?gene=OMA1 |
| COL1A1 | Collagen Type I Alpha 1 Chain | Protein Coding | P02452 | 62 | GC17M097561 | 1.99510836601257 | https://www.genecards.org/cgi-bin/carddisp.pl?gene=COL1A1 |
| HK2 | Hexokinase 2 | Protein Coding | P52789 | 58 | GC02P075491 | 1.99165773391724 | https://www.genecards.org/cgi-bin/carddisp.pl?gene=HK2 |
| CCL20 | C-C Motif Chemokine Ligand 20 | Protein Coding | P78556 | 54 | GC02P227846 | 1.99165773391724 | https://www.genecards.org/cgi-bin/carddisp.pl?gene=CCL20 |
| COG2 | Component Of Oligomeric Golgi Complex 2 | Protein Coding | Q14746 | 51 | GC01P230642 | 1.99165773391724 | https://www.genecards.org/cgi-bin/carddisp.pl?gene=COG2 |
| HP | Haptoglobin | Protein Coding | P00738 | 58 | GC16P122099 | 1.988685131 | https://www.genecards.org/cgi-bin/carddisp.pl?gene=HP |
| IL11 | Interleukin 11 | Protein Coding | P20809 | 53 | GC19M055364 | 1.98001444339752 | https://www.genecards.org/cgi-bin/carddisp.pl?gene=IL11 |
| MIR874 | MicroRNA 874 | RNA Gene |  | 27 | GC05M137647 | 1.98001444339752 | https://www.genecards.org/cgi-bin/carddisp.pl?gene=MIR874 |
| APOC1 | Apolipoprotein C1 | Protein Coding | P02654 | 53 | GC19P044914 | 1.97526502609253 | https://www.genecards.org/cgi-bin/carddisp.pl?gene=APOC1 |
| SIGLEC5 | Sialic Acid Binding Ig Like Lectin 5 | Protein Coding | O15389 | 50 | GC19M109164 | 1.97489476203918 | https://www.genecards.org/cgi-bin/carddisp.pl?gene=SIGLEC5 |
| OPA1 | OPA1 Mitochondrial Dynamin Like GTPase | Protein Coding | O60313 | 57 | GC03P193594 | 1.964311004 | https://www.genecards.org/cgi-bin/carddisp.pl?gene=OPA1 |
| MC2R | Melanocortin 2 Receptor | Protein Coding | Q01718 | 58 | GC18M038218 | 1.95492386817932 | https://www.genecards.org/cgi-bin/carddisp.pl?gene=MC2R |
| MC5R | Melanocortin 5 Receptor | Protein Coding | P33032 | 54 | GC18P013824 | 1.95492386817932 | https://www.genecards.org/cgi-bin/carddisp.pl?gene=MC5R |
| GTF3A | General Transcription Factor IIIA | Protein Coding | Q92664 | 45 | GC13P028953 | 1.9521918296814 | https://www.genecards.org/cgi-bin/carddisp.pl?gene=GTF3A |
| THBS1 | Thrombospondin 1 | Protein Coding | P07996 | 59 | GC15P039581 | 1.94820976257324 | https://www.genecards.org/cgi-bin/carddisp.pl?gene=THBS1 |
| NPPB | Natriuretic Peptide B | Protein Coding | P16860 | 55 | GC01M022466 | 1.94820976257324 | https://www.genecards.org/cgi-bin/carddisp.pl?gene=NPPB |
| LOC108167315 | POMC Promoter Region | Functional Element |  | 6 | GC02P029576 | 1.9389671087265 | https://www.genecards.org/cgi-bin/carddisp.pl?gene=LOC108167315 |
| EP300 | E1A Binding Protein P300 | Protein Coding | Q09472 | 65 | GC22P092249 | 1.93557095527649 | https://www.genecards.org/cgi-bin/carddisp.pl?gene=EP300 |
| CD40 | CD40 Molecule | Protein Coding | P25942 | 63 | GC20P046118 | 1.93167901039124 | https://www.genecards.org/cgi-bin/carddisp.pl?gene=CD40 |
| BIRC5 | Baculoviral IAP Repeat Containing 5 | Protein Coding | O15392 | 58 | GC17P078214 | 1.93167901039124 | https://www.genecards.org/cgi-bin/carddisp.pl?gene=BIRC5 |
| TJP1 | Tight Junction Protein 1 | Protein Coding | Q07157 | 58 | GC15M029699 | 1.93167901039124 | https://www.genecards.org/cgi-bin/carddisp.pl?gene=TJP1 |
| MBD4 | Methyl-CpG Binding Domain 4, DNA Glycosylase | Protein Coding | O95243 | 56 | GC03M129430 | 1.92872285842896 | https://www.genecards.org/cgi-bin/carddisp.pl?gene=MBD4 |
| NES | Nestin | Protein Coding | P48681 | 52 | GC01M156668 | 1.92872285842896 | https://www.genecards.org/cgi-bin/carddisp.pl?gene=NES |
| MIR140 | MicroRNA 140 | RNA Gene |  | 32 | GC16P122015 | 1.92872285842896 | https://www.genecards.org/cgi-bin/carddisp.pl?gene=MIR140 |
| TCF7L2 | Transcription Factor 7 Like 2 | Protein Coding | Q9NQB0 | 59 | GC10P112950 | 1.92273044586182 | https://www.genecards.org/cgi-bin/carddisp.pl?gene=TCF7L2 |
| GDF15 | Growth Differentiation Factor 15 | Protein Coding | Q99988 | 56 | GC19P152105 | 1.92110800743103 | https://www.genecards.org/cgi-bin/carddisp.pl?gene=GDF15 |
| EGR1 | Early Growth Response 1 | Protein Coding | P18146 | 57 | GC05P138465 | 1.91545808315277 | https://www.genecards.org/cgi-bin/carddisp.pl?gene=EGR1 |
| NPPA | Natriuretic Peptide A | Protein Coding | P01160 | 57 | GC01M022465 | 1.91468226909637 | https://www.genecards.org/cgi-bin/carddisp.pl?gene=NPPA |
| CCL5 | C-C Motif Chemokine Ligand 5 | Protein Coding | P13501 | 56 | GC17M035871 | 1.91468226909637 | https://www.genecards.org/cgi-bin/carddisp.pl?gene=CCL5 |
| P2RX5-TAX1BP3 | P2RX5-TAX1BP3 Readthrough (NMD Candidate) | RNA Gene |  | 20 | GC17M096016 | 1.91468226909637 | https://www.genecards.org/cgi-bin/carddisp.pl?gene=P2RX5-TAX1BP3 |
| TFRC | Transferrin Receptor | Protein Coding | P02786 | 64 | GC03M196703 | 1.89717781543732 | https://www.genecards.org/cgi-bin/carddisp.pl?gene=TFRC |
| PLA2G4A | Phospholipase A2 Group IVA | Protein Coding | P47712 | 62 | GC01P186798 | 1.89717781543732 | https://www.genecards.org/cgi-bin/carddisp.pl?gene=PLA2G4A |
| SLC2A5 | Solute Carrier Family 2 Member 5 | Protein Coding | P22732 | 54 | GC01M022399 | 1.89717781543732 | https://www.genecards.org/cgi-bin/carddisp.pl?gene=SLC2A5 |
| SLC38A2 | Solute Carrier Family 38 Member 2 | Protein Coding | Q96QD8 | 51 | GC12M046358 | 1.89717781543732 | https://www.genecards.org/cgi-bin/carddisp.pl?gene=SLC38A2 |
| APOH | Apolipoprotein H | Protein Coding | P02749 | 58 | GC17M066212 | 1.89448833465576 | https://www.genecards.org/cgi-bin/carddisp.pl?gene=APOH |
| CPT1B | Carnitine Palmitoyltransferase 1B | Protein Coding | Q92523 | 58 | GC22M086281 | 1.89448833465576 | https://www.genecards.org/cgi-bin/carddisp.pl?gene=CPT1B |
| TAC3 | Tachykinin Precursor 3 | Protein Coding | Q9UHF0 | 54 | GC12M060450 | 1.89448833465576 | https://www.genecards.org/cgi-bin/carddisp.pl?gene=TAC3 |
| LIPG | Lipase G, Endothelial Type | Protein Coding | Q9Y5X9 | 57 | GC18P049560 | 1.89289259910583 | https://www.genecards.org/cgi-bin/carddisp.pl?gene=LIPG |
| SPARC | Secreted Protein Acidic And Cysteine Rich | Protein Coding | P09486 | 62 | GC05M151661 | 1.8791172504425 | https://www.genecards.org/cgi-bin/carddisp.pl?gene=SPARC |
| HSPD1 | Heat Shock Protein Family D (Hsp60) Member 1 | Protein Coding | P10809 | 61 | GC02M197486 | 1.8791172504425 | https://www.genecards.org/cgi-bin/carddisp.pl?gene=HSPD1 |
| SNX6 | Sorting Nexin 6 | Protein Coding | Q9UNH7 | 47 | GC14M034561 | 1.87849235534668 | https://www.genecards.org/cgi-bin/carddisp.pl?gene=SNX6 |
| ACBD7 | Acyl-CoA Binding Domain Containing 7 | Protein Coding | Q8N6N7 | 40 | GC10M015348 | 1.87849235534668 | https://www.genecards.org/cgi-bin/carddisp.pl?gene=ACBD7 |
| BMP6 | Bone Morphogenetic Protein 6 | Protein Coding | P22004 | 57 | GC06P007726 | 1.87552571296692 | https://www.genecards.org/cgi-bin/carddisp.pl?gene=BMP6 |
| RPS6 | Ribosomal Protein S6 | Protein Coding | P62753 | 57 | GC09M019375 | 1.87254166603088 | https://www.genecards.org/cgi-bin/carddisp.pl?gene=RPS6 |
| CRHR1 | Corticotropin Releasing Hormone Receptor 1 | Protein Coding | P34998 | 57 | GC17P045784 | 1.87248551845551 | https://www.genecards.org/cgi-bin/carddisp.pl?gene=CRHR1 |
| BBS4 | Bardet-Biedl Syndrome 4 | Protein Coding | Q96RK4 | 53 | GC15P072686 | 1.86562347412109 | https://www.genecards.org/cgi-bin/carddisp.pl?gene=BBS4 |
| DLK1 | Delta Like Non-Canonical Notch Ligand 1 | Protein Coding | P80370 | 58 | GC14P120459 | 1.86276745796204 | https://www.genecards.org/cgi-bin/carddisp.pl?gene=DLK1 |
| HLA-G | Major Histocompatibility Complex, Class I, G | Protein Coding | P17693 | 57 | GC06P181845 | 1.8604439496994 | https://www.genecards.org/cgi-bin/carddisp.pl?gene=HLA-G |
| BBS10 | Bardet-Biedl Syndrome 10 | Protein Coding | Q8TAM1 | 52 | GC12M076344 | 1.8604439496994 | https://www.genecards.org/cgi-bin/carddisp.pl?gene=BBS10 |
| BBS5 | Bardet-Biedl Syndrome 5 | Protein Coding | Q8N3I7 | 50 | GC02P169498 | 1.8604439496994 | https://www.genecards.org/cgi-bin/carddisp.pl?gene=BBS5 |
| ACP1 | Acid Phosphatase 1 | Protein Coding | P24666 | 55 | GC02P001239 | 1.85746502876282 | https://www.genecards.org/cgi-bin/carddisp.pl?gene=ACP1 |
| IL1R1 | Interleukin 1 Receptor Type 1 | Protein Coding | P14778 | 60 | GC02P102136 | 1.85352838039398 | https://www.genecards.org/cgi-bin/carddisp.pl?gene=IL1R1 |
| MMP3 | Matrix Metallopeptidase 3 | Protein Coding | P08254 | 62 | GC11M102835 | 1.84109103679657 | https://www.genecards.org/cgi-bin/carddisp.pl?gene=MMP3 |
| SIRPA | Signal Regulatory Protein Alpha | Protein Coding | P78324 | 58 | GC20P001894 | 1.84109103679657 | https://www.genecards.org/cgi-bin/carddisp.pl?gene=SIRPA |
| AGT | Angiotensinogen | Protein Coding | P01019 | 62 | GC01M230690 | 1.83985352516174 | https://www.genecards.org/cgi-bin/carddisp.pl?gene=AGT |
| TGFB2 | Transforming Growth Factor Beta 2 | Protein Coding | P61812 | 64 | GC01P218345 | 1.82507133483887 | https://www.genecards.org/cgi-bin/carddisp.pl?gene=TGFB2 |
| CDX2 | Caudal Type Homeobox 2 | Protein Coding | Q99626 | 54 | GC13M027962 | 1.82097816467285 | https://www.genecards.org/cgi-bin/carddisp.pl?gene=CDX2 |
| BBS7 | Bardet-Biedl Syndrome 7 | Protein Coding | Q8IWZ6 | 50 | GC04M121824 | 1.82097816467285 | https://www.genecards.org/cgi-bin/carddisp.pl?gene=BBS7 |
| LOC129935068 | ATAC-STARR-Seq Lymphoblastoid Active Region 16738 | Functional Element |  | 7 | GC02P169501 | 1.82097816467285 | https://www.genecards.org/cgi-bin/carddisp.pl?gene=LOC129935068 |
| ADRB1 | Adrenoceptor Beta 1 | Protein Coding | P08588 | 62 | GC10P125703 | 1.80853879451752 | https://www.genecards.org/cgi-bin/carddisp.pl?gene=ADRB1 |
| MAPT | Microtubule Associated Protein Tau | Protein Coding | P10636 | 62 | GC17P045894 | 1.80000841617584 | https://www.genecards.org/cgi-bin/carddisp.pl?gene=MAPT |
| ARHGEF7 | Rho Guanine Nucleotide Exchange Factor 7 | Protein Coding | Q14155 | 55 | GC13P111114 | 1.80000841617584 | https://www.genecards.org/cgi-bin/carddisp.pl?gene=ARHGEF7 |
| UBC | Ubiquitin C | Protein Coding | P0CG48 | 56 | GC12M124911 | 1.79909658432007 | https://www.genecards.org/cgi-bin/carddisp.pl?gene=UBC |
| GRN | Granulin Precursor | Protein Coding | P28799 | 61 | GC17P044345 | 1.79677677154541 | https://www.genecards.org/cgi-bin/carddisp.pl?gene=GRN |
| TTF2 | Transcription Termination Factor 2 | Protein Coding | Q9UNY4 | 51 | GC01P117060 | 1.77806174755096 | https://www.genecards.org/cgi-bin/carddisp.pl?gene=TTF2 |
| MIR27A | MicroRNA 27a | RNA Gene |  | 32 | GC19M108247 | 1.77806174755096 | https://www.genecards.org/cgi-bin/carddisp.pl?gene=MIR27A |
| SLC6A3 | Solute Carrier Family 6 Member 3 | Protein Coding | Q01959 | 63 | GC05M001392 | 1.77119016647339 | https://www.genecards.org/cgi-bin/carddisp.pl?gene=SLC6A3 |
| KLF7 | KLF Transcription Factor 7 | Protein Coding | O75840 | 51 | GC02M207074 | 1.76279246807098 | https://www.genecards.org/cgi-bin/carddisp.pl?gene=KLF7 |
| CCL11 | C-C Motif Chemokine Ligand 11 | Protein Coding | P51671 | 58 | GC17P034285 | 1.76020336151123 | https://www.genecards.org/cgi-bin/carddisp.pl?gene=CCL11 |
| GRP | Gastrin Releasing Peptide | Protein Coding | P07492 | 52 | GC18P059220 | 1.75904846191406 | https://www.genecards.org/cgi-bin/carddisp.pl?gene=GRP |
| LPIN1 | Lipin 1 | Protein Coding | Q14693 | 59 | GC02P011677 | 1.75818276405334 | https://www.genecards.org/cgi-bin/carddisp.pl?gene=LPIN1 |
| CHUK | Component Of Inhibitor Of Nuclear Factor Kappa B Kinase Complex | Protein Coding | O15111 | 63 | GC10M100597 | 1.75498747825623 | https://www.genecards.org/cgi-bin/carddisp.pl?gene=CHUK |
| SLC2A3 | Solute Carrier Family 2 Member 3 | Protein Coding | P11169 | 61 | GC12M007919 | 1.75498747825623 | https://www.genecards.org/cgi-bin/carddisp.pl?gene=SLC2A3 |
| CHI3L1 | Chitinase 3 Like 1 | Protein Coding | P36222 | 59 | GC01M203148 | 1.75498747825623 | https://www.genecards.org/cgi-bin/carddisp.pl?gene=CHI3L1 |
| NMB | Neuromedin B | Protein Coding | P08949 | 52 | GC15M084655 | 1.74926555156708 | https://www.genecards.org/cgi-bin/carddisp.pl?gene=NMB |
| H19 | H19 Imprinted Maternally Expressed Transcript | RNA Gene |  | 37 | GC11M001995 | 1.74926555156708 | https://www.genecards.org/cgi-bin/carddisp.pl?gene=H19 |
| APLNR | Apelin Receptor | Protein Coding | P35414 | 57 | GC11M057233 | 1.7408435344696 | https://www.genecards.org/cgi-bin/carddisp.pl?gene=APLNR |
| TPH1 | Tryptophan Hydroxylase 1 | Protein Coding | P17752 | 58 | GC11M018171 | 1.73731541633606 | https://www.genecards.org/cgi-bin/carddisp.pl?gene=TPH1 |
| PAX4 | Paired Box 4 | Protein Coding | O43316 | 52 | GC07M127610 | 1.73731541633606 | https://www.genecards.org/cgi-bin/carddisp.pl?gene=PAX4 |
| SMAD3 | SMAD Family Member 3 | Protein Coding | P84022 | 66 | GC15P067063 | 1.73333525657654 | https://www.genecards.org/cgi-bin/carddisp.pl?gene=SMAD3 |
| AGPAT1 | 1-Acylglycerol-3-Phosphate O-Acyltransferase 1 | Protein Coding | Q99943 | 51 | GC06M032168 | 1.73069655895233 | https://www.genecards.org/cgi-bin/carddisp.pl?gene=AGPAT1 |
| CNTFR | Ciliary Neurotrophic Factor Receptor | Protein Coding | P26992 | 57 | GC09M034551 | 1.73059141635895 | https://www.genecards.org/cgi-bin/carddisp.pl?gene=CNTFR |
| HSD11B2 | Hydroxysteroid 11-Beta Dehydrogenase 2 | Protein Coding | P80365 | 58 | GC16P121870 | 1.72915279865265 | https://www.genecards.org/cgi-bin/carddisp.pl?gene=HSD11B2 |
| MAT1A | Methionine Adenosyltransferase 1A | Protein Coding | Q00266 | 60 | GC10M080271 | 1.72570216655731 | https://www.genecards.org/cgi-bin/carddisp.pl?gene=MAT1A |
| NR1H4 | Nuclear Receptor Subfamily 1 Group H Member 4 | Protein Coding | Q96RI1 | 60 | GC12P100473 | 1.72570216655731 | https://www.genecards.org/cgi-bin/carddisp.pl?gene=NR1H4 |
| IL5 | Interleukin 5 | Protein Coding | P05113 | 58 | GC05M132541 | 1.70818305015564 | https://www.genecards.org/cgi-bin/carddisp.pl?gene=IL5 |
| PDE3A | Phosphodiesterase 3A | Protein Coding | Q14432 | 60 | GC12P074416 | 1.70461666584015 | https://www.genecards.org/cgi-bin/carddisp.pl?gene=PDE3A |
| ENPP3 | Ectonucleotide Pyrophosphatase/Phosphodiesterase 3 | Protein Coding | O14638 | 55 | GC06P183105 | 1.70461666584015 | https://www.genecards.org/cgi-bin/carddisp.pl?gene=ENPP3 |
| GPLD1 | Glycosylphosphatidylinositol Specific Phospholipase D1 | Protein Coding | P80108 | 55 | GC06M024573 | 1.69835543632507 | https://www.genecards.org/cgi-bin/carddisp.pl?gene=GPLD1 |
| AGER | Advanced Glycosylation End-Product Specific Receptor | Protein Coding | Q15109 | 59 | GC06M032180 | 1.6889683008194 | https://www.genecards.org/cgi-bin/carddisp.pl?gene=AGER |
| DGAT1 | Diacylglycerol O-Acyltransferase 1 | Protein Coding | O75907 | 60 | GC08M147388 | 1.68296456336975 | https://www.genecards.org/cgi-bin/carddisp.pl?gene=DGAT1 |
| SNORD44 | Small Nucleolar RNA, C/D Box 44 | RNA Gene |  | 21 | GC01M175154 | 1.67671024799347 | https://www.genecards.org/cgi-bin/carddisp.pl?gene=SNORD44 |
| ADIPQTL4 | Adiponectin, Serum Level Of, QTL4 | Genetic Locus |  | 2 | GC11U901278 | 1.66961526870728 | https://www.genecards.org/cgi-bin/carddisp.pl?gene=ADIPQTL4 |
| IL17A | Interleukin 17A | Protein Coding | Q16552 | 55 | GC06P052186 | 1.66572344303131 | https://www.genecards.org/cgi-bin/carddisp.pl?gene=IL17A |
| PTPRJ | Protein Tyrosine Phosphatase Receptor Type J | Protein Coding | Q12913 | 60 | GC11P049139 | 1.65493321418762 | https://www.genecards.org/cgi-bin/carddisp.pl?gene=PTPRJ |
| TRC-GCA24-1 | TRNA-Cys (GCA) 24-1 | RNA Gene |  | 14 | GC17M097073 | 1.65493321418762 | https://www.genecards.org/cgi-bin/carddisp.pl?gene=TRC-GCA24-1 |
| PLA2G2A | Phospholipase A2 Group IIA | Protein Coding | P14555 | 60 | GC01M019975 | 1.64950251579285 | https://www.genecards.org/cgi-bin/carddisp.pl?gene=PLA2G2A |
| PNLIP | Pancreatic Lipase | Protein Coding | P16233 | 60 | GC10P116545 | 1.64950251579285 | https://www.genecards.org/cgi-bin/carddisp.pl?gene=PNLIP |
| APOA2 | Apolipoprotein A2 | Protein Coding | P02652 | 58 | GC01M161222 | 1.64950251579285 | https://www.genecards.org/cgi-bin/carddisp.pl?gene=APOA2 |
| PLIN2 | Perilipin 2 | Protein Coding | Q99541 | 54 | GC09M019319 | 1.64950251579285 | https://www.genecards.org/cgi-bin/carddisp.pl?gene=PLIN2 |
| BAK1 | BCL2 Antagonist/Killer 1 | Protein Coding | Q16611 | 57 | GC06M033572 | 1.64872670173645 | https://www.genecards.org/cgi-bin/carddisp.pl?gene=BAK1 |
| FGF9 | Fibroblast Growth Factor 9 | Protein Coding | P31371 | 57 | GC13P021671 | 1.64872670173645 | https://www.genecards.org/cgi-bin/carddisp.pl?gene=FGF9 |
| INSL3 | Insulin Like 3 | Protein Coding | P51460 | 52 | GC19M017816 | 1.64636373519897 | https://www.genecards.org/cgi-bin/carddisp.pl?gene=INSL3 |
| VAV1 | Vav Guanine Nucleotide Exchange Factor 1 | Protein Coding | P15498 | 58 | GC19P006772 | 1.63185882568359 | https://www.genecards.org/cgi-bin/carddisp.pl?gene=VAV1 |
| TGFBR1 | Transforming Growth Factor Beta Receptor 1 | Protein Coding | P36897 | 67 | GC09P107186 | 1.63122224807739 | https://www.genecards.org/cgi-bin/carddisp.pl?gene=TGFBR1 |
| ACVRL1 | Activin A Receptor Like Type 1 | Protein Coding | P37023 | 63 | GC12P051906 | 1.63122224807739 | https://www.genecards.org/cgi-bin/carddisp.pl?gene=ACVRL1 |
| CPT2 | Carnitine Palmitoyltransferase 2 | Protein Coding | P23786 | 62 | GC01P053196 | 1.63122224807739 | https://www.genecards.org/cgi-bin/carddisp.pl?gene=CPT2 |
| REN | Renin | Protein Coding | P00797 | 61 | GC01M204154 | 1.63122224807739 | https://www.genecards.org/cgi-bin/carddisp.pl?gene=REN |
| HBEGF | Heparin Binding EGF Like Growth Factor | Protein Coding | Q99075 | 57 | GC05M140332 | 1.63122224807739 | https://www.genecards.org/cgi-bin/carddisp.pl?gene=HBEGF |
| RHOD | Ras Homolog Family Member D | Protein Coding | O00212 | 50 | GC11P107960 | 1.63122224807739 | https://www.genecards.org/cgi-bin/carddisp.pl?gene=RHOD |
| BMP2 | Bone Morphogenetic Protein 2 | Protein Coding | P12643 | 58 | GC20P006767 | 1.62853276729584 | https://www.genecards.org/cgi-bin/carddisp.pl?gene=BMP2 |
| KDR | Kinase Insert Domain Receptor | Protein Coding | P35968 | 67 | GC04M055078 | 1.61316168308258 | https://www.genecards.org/cgi-bin/carddisp.pl?gene=KDR |
| CDC42 | Cell Division Cycle 42 | Protein Coding | P60953 | 63 | GC01P022052 | 1.61316168308258 | https://www.genecards.org/cgi-bin/carddisp.pl?gene=CDC42 |
| MTTP | Microsomal Triglyceride Transfer Protein | Protein Coding | P55157 | 57 | GC04P099563 | 1.61316168308258 | https://www.genecards.org/cgi-bin/carddisp.pl?gene=MTTP |
| TSC22D3 | TSC22 Domain Family Member 3 | Protein Coding | Q99576 | 52 | GC0XM107713 | 1.61316168308258 | https://www.genecards.org/cgi-bin/carddisp.pl?gene=TSC22D3 |
| SNCG | Synuclein Gamma | Protein Coding | O76070 | 55 | GC10P123977 | 1.61279714107513 | https://www.genecards.org/cgi-bin/carddisp.pl?gene=SNCG |
| NFKB1 | Nuclear Factor Kappa B Subunit 1 | Protein Coding | P19838 | 66 | GC04P102501 | 1.61279129981995 | https://www.genecards.org/cgi-bin/carddisp.pl?gene=NFKB1 |
| CXCL12 | C-X-C Motif Chemokine Ligand 12 | Protein Coding | P48061 | 59 | GC10M044370 | 1.61279129981995 | https://www.genecards.org/cgi-bin/carddisp.pl?gene=CXCL12 |
| TMED9 | Transmembrane P24 Trafficking Protein 9 | Protein Coding | Q9BVK6 | 42 | GC05P190372 | 1.60746288299561 | https://www.genecards.org/cgi-bin/carddisp.pl?gene=TMED9 |
| PLAU | Plasminogen Activator, Urokinase | Protein Coding | P00749 | 64 | GC10P073909 | 1.60658609867096 | https://www.genecards.org/cgi-bin/carddisp.pl?gene=PLAU |
| ANGPT2 | Angiopoietin 2 | Protein Coding | O15123 | 61 | GC08M006499 | 1.60658609867096 | https://www.genecards.org/cgi-bin/carddisp.pl?gene=ANGPT2 |
| PGF | Placental Growth Factor | Protein Coding | P49763 | 56 | GC14M074941 | 1.60658609867096 | https://www.genecards.org/cgi-bin/carddisp.pl?gene=PGF |
| PTHLH | Parathyroid Hormone Like Hormone | Protein Coding | P12272 | 58 | GC12M036019 | 1.59448838233948 | https://www.genecards.org/cgi-bin/carddisp.pl?gene=PTHLH |
| PTX3 | Pentraxin 3 | Protein Coding | P26022 | 55 | GC03P157436 | 1.59448838233948 | https://www.genecards.org/cgi-bin/carddisp.pl?gene=PTX3 |
| IGHE | Immunoglobulin Heavy Constant Epsilon | Protein Coding | P01854 | 42 | GC14M124465 | 1.59448838233948 | https://www.genecards.org/cgi-bin/carddisp.pl?gene=IGHE |
| APOA5 | Apolipoprotein A5 | Protein Coding | Q6Q788 | 55 | GC11M116789 | 1.58706450462341 | https://www.genecards.org/cgi-bin/carddisp.pl?gene=APOA5 |
| CCKBR | Cholecystokinin B Receptor | Protein Coding | P32239 | 57 | GC11P006259 | 1.58351182937622 | https://www.genecards.org/cgi-bin/carddisp.pl?gene=CCKBR |
| LBP | Lipopolysaccharide Binding Protein | Protein Coding | P18428 | 56 | GC20P038346 | 1.58351182937622 | https://www.genecards.org/cgi-bin/carddisp.pl?gene=LBP |
| RHEB | Ras Homolog, MTORC1 Binding | Protein Coding | Q15382 | 63 | GC07M151466 | 1.58148813247681 | https://www.genecards.org/cgi-bin/carddisp.pl?gene=RHEB |
| XDH | Xanthine Dehydrogenase | Protein Coding | P47989 | 60 | GC02M031334 | 1.57513546943665 | https://www.genecards.org/cgi-bin/carddisp.pl?gene=XDH |
| SFRP5 | Secreted Frizzled Related Protein 5 | Protein Coding | Q5T4F7 | 48 | GC10M097766 | 1.57513546943665 | https://www.genecards.org/cgi-bin/carddisp.pl?gene=SFRP5 |
| MPO | Myeloperoxidase | Protein Coding | P05164 | 65 | GC17M058269 | 1.55911576747894 | https://www.genecards.org/cgi-bin/carddisp.pl?gene=MPO |
| ATM | ATM Serine/Threonine Kinase | Protein Coding | Q13315 | 66 | GC11P108222 | 1.55502271652222 | https://www.genecards.org/cgi-bin/carddisp.pl?gene=ATM |
| F8 | Coagulation Factor VIII | Protein Coding | P00451 | 61 | GC0XM154835 | 1.55502271652222 | https://www.genecards.org/cgi-bin/carddisp.pl?gene=F8 |
| HSPA5 | Heat Shock Protein Family A (Hsp70) Member 5 | Protein Coding | P11021 | 61 | GC09M125234 | 1.55502271652222 | https://www.genecards.org/cgi-bin/carddisp.pl?gene=HSPA5 |
| VLDLR | Very Low Density Lipoprotein Receptor | Protein Coding | P98155 | 61 | GC09P002611 | 1.55502271652222 | https://www.genecards.org/cgi-bin/carddisp.pl?gene=VLDLR |
| A2M | Alpha-2-Macroglobulin | Protein Coding | P01023 | 59 | GC12M009067 | 1.55502271652222 | https://www.genecards.org/cgi-bin/carddisp.pl?gene=A2M |
| LRP8 | LDL Receptor Related Protein 8 | Protein Coding | Q14114 | 56 | GC01M053243 | 1.55502271652222 | https://www.genecards.org/cgi-bin/carddisp.pl?gene=LRP8 |
| BAG1 | BAG Cochaperone 1 | Protein Coding | Q99933 | 55 | GC09M033245 | 1.55502271652222 | https://www.genecards.org/cgi-bin/carddisp.pl?gene=BAG1 |
| CRHR2 | Corticotropin Releasing Hormone Receptor 2 | Protein Coding | Q13324 | 55 | GC07M030651 | 1.55502271652222 | https://www.genecards.org/cgi-bin/carddisp.pl?gene=CRHR2 |
| MUC2 | Mucin 2, Oligomeric Mucus/Gel-Forming | Protein Coding | Q02817 | 52 | GC11P001074 | 1.55502271652222 | https://www.genecards.org/cgi-bin/carddisp.pl?gene=MUC2 |
| MUC4 | Mucin 4, Cell Surface Associated | Protein Coding | Q99102 | 52 | GC03M195746 | 1.55502271652222 | https://www.genecards.org/cgi-bin/carddisp.pl?gene=MUC4 |
| MUC3A | Mucin 3A, Cell Surface Associated | Protein Coding | Q02505 | 46 | GC07P100949 | 1.55502271652222 | https://www.genecards.org/cgi-bin/carddisp.pl?gene=MUC3A |
| SCARB1 | Scavenger Receptor Class B Member 1 | Protein Coding | Q8WTV0 | 60 | GC12M124776 | 1.55349791049957 | https://www.genecards.org/cgi-bin/carddisp.pl?gene=SCARB1 |
| RELA | RELA Proto-Oncogene, NF-KB Subunit | Protein Coding | Q04206 | 64 | GC11M065653 | 1.53405284881592 | https://www.genecards.org/cgi-bin/carddisp.pl?gene=RELA |
| CSNK2A1 | Casein Kinase 2 Alpha 1 | Protein Coding | P68400 | 63 | GC20M000472 | 1.53405284881592 | https://www.genecards.org/cgi-bin/carddisp.pl?gene=CSNK2A1 |
| IRF1 | Interferon Regulatory Factor 1 | Protein Coding | P10914 | 60 | GC05M132440 | 1.53405284881592 | https://www.genecards.org/cgi-bin/carddisp.pl?gene=IRF1 |
| AMH | Anti-Mullerian Hormone | Protein Coding | P03971 | 57 | GC19P151440 | 1.53405284881592 | https://www.genecards.org/cgi-bin/carddisp.pl?gene=AMH |
| HAMP | Hepcidin Antimicrobial Peptide | Protein Coding | P81172 | 56 | GC19P152385 | 1.53405284881592 | https://www.genecards.org/cgi-bin/carddisp.pl?gene=HAMP |
| SLC25A27 | Solute Carrier Family 25 Member 27 | Protein Coding | O95847 | 51 | GC06P046652 | 1.53405284881592 | https://www.genecards.org/cgi-bin/carddisp.pl?gene=SLC25A27 |
| MIR146B | MicroRNA 146b | RNA Gene |  | 29 | GC10P102436 | 1.53405284881592 | https://www.genecards.org/cgi-bin/carddisp.pl?gene=MIR146B |
| MIR218-1 | MicroRNA 218-1 | RNA Gene |  | 27 | GC04P030129 | 1.53405284881592 | https://www.genecards.org/cgi-bin/carddisp.pl?gene=MIR218-1 |
| MIR218-2 | MicroRNA 218-2 | RNA Gene |  | 25 | GC05M168768 | 1.53405284881592 | https://www.genecards.org/cgi-bin/carddisp.pl?gene=MIR218-2 |
| HCRTR2 | Hypocretin Receptor 2 | Protein Coding | O43614 | 57 | GC06P055106 | 1.53314101696014 | https://www.genecards.org/cgi-bin/carddisp.pl?gene=HCRTR2 |
| RNF41 | Ring Finger Protein 41 | Protein Coding | Q9H4P4 | 48 | GC12M056202 | 1.53314101696014 | https://www.genecards.org/cgi-bin/carddisp.pl?gene=RNF41 |
| PLAAT3 | Phospholipase A And Acyltransferase 3 | Protein Coding | P53816 | 50 | GC11M139694 | 1.52950394153595 | https://www.genecards.org/cgi-bin/carddisp.pl?gene=PLAAT3 |
| MDK | Midkine | Protein Coding | P21741 | 56 | GC11P046380 | 1.52559804916382 | https://www.genecards.org/cgi-bin/carddisp.pl?gene=MDK |
| IL15 | Interleukin 15 | Protein Coding | P40933 | 55 | GC04P141636 | 1.52559804916382 | https://www.genecards.org/cgi-bin/carddisp.pl?gene=IL15 |
| FETUB | Fetuin B | Protein Coding | Q9UGM5 | 45 | GC03P186635 | 1.52323508262634 | https://www.genecards.org/cgi-bin/carddisp.pl?gene=FETUB |
| ANGPTL3 | Angiopoietin Like 3 | Protein Coding | Q9Y5C1 | 60 | GC01P062597 | 1.52218890190125 | https://www.genecards.org/cgi-bin/carddisp.pl?gene=ANGPTL3 |
| TRAF4 | TNF Receptor Associated Factor 4 | Protein Coding | Q9BUZ4 | 54 | GC17P154300 | 1.52160286903381 | https://www.genecards.org/cgi-bin/carddisp.pl?gene=TRAF4 |
| ENHO | Energy Homeostasis Associated | Protein Coding | Q6UWT2 | 39 | GC09M035843 | 1.52097165584564 | https://www.genecards.org/cgi-bin/carddisp.pl?gene=ENHO |
| PSMB8 | Proteasome 20S Subunit Beta 8 | Protein Coding | P28062 | 62 | GC06M032840 | 1.51792585849762 | https://www.genecards.org/cgi-bin/carddisp.pl?gene=PSMB8 |
| FFAR2 | Free Fatty Acid Receptor 2 | Protein Coding | O15552 | 53 | GC19P152388 | 1.51651155948639 | https://www.genecards.org/cgi-bin/carddisp.pl?gene=FFAR2 |
| SUCNR1 | Succinate Receptor 1 | Protein Coding | Q9BXA5 | 47 | GC03P151873 | 1.51651155948639 | https://www.genecards.org/cgi-bin/carddisp.pl?gene=SUCNR1 |
| CTNNB1 | Catenin Beta 1 | Protein Coding | P35222 | 67 | GC03P041194 | 1.51210618019104 | https://www.genecards.org/cgi-bin/carddisp.pl?gene=CTNNB1 |
| CASP8 | Caspase 8 | Protein Coding | Q14790 | 65 | GC02P201233 | 1.51210618019104 | https://www.genecards.org/cgi-bin/carddisp.pl?gene=CASP8 |
| MMP1 | Matrix Metallopeptidase 1 | Protein Coding | P03956 | 62 | GC11M140392 | 1.51210618019104 | https://www.genecards.org/cgi-bin/carddisp.pl?gene=MMP1 |
| VEGFC | Vascular Endothelial Growth Factor C | Protein Coding | P49767 | 60 | GC04M176683 | 1.51210618019104 | https://www.genecards.org/cgi-bin/carddisp.pl?gene=VEGFC |
| MTNR1B | Melatonin Receptor 1B | Protein Coding | P49286 | 57 | GC11P092969 | 1.51210618019104 | https://www.genecards.org/cgi-bin/carddisp.pl?gene=MTNR1B |
| TFAP2B | Transcription Factor AP-2 Beta | Protein Coding | Q92481 | 57 | GC06P182131 | 1.51210618019104 | https://www.genecards.org/cgi-bin/carddisp.pl?gene=TFAP2B |
| SCARNA5 | Small Cajal Body-Specific RNA 5 | RNA Gene |  | 28 | GC02P233275 | 1.51210618019104 | https://www.genecards.org/cgi-bin/carddisp.pl?gene=SCARNA5 |
| MIF | Macrophage Migration Inhibitory Factor | Protein Coding | P14174 | 61 | GC22P023894 | 1.51150405406952 | https://www.genecards.org/cgi-bin/carddisp.pl?gene=MIF |
| SMAD2 | SMAD Family Member 2 | Protein Coding | Q15796 | 65 | GC18M047809 | 1.50523447990417 | https://www.genecards.org/cgi-bin/carddisp.pl?gene=SMAD2 |
| GAD1 | Glutamate Decarboxylase 1 | Protein Coding | Q99259 | 62 | GC02P170813 | 1.50159752368927 | https://www.genecards.org/cgi-bin/carddisp.pl?gene=GAD1 |
| RARA | Retinoic Acid Receptor Alpha | Protein Coding | P10276 | 62 | GC17P040309 | 1.49003314971924 | https://www.genecards.org/cgi-bin/carddisp.pl?gene=RARA |
| AXIN1 | Axin 1 | Protein Coding | O15169 | 60 | GC16M000287 | 1.49003314971924 | https://www.genecards.org/cgi-bin/carddisp.pl?gene=AXIN1 |
| WNT1 | Wnt Family Member 1 | Protein Coding | P04628 | 60 | GC12P075041 | 1.49003314971924 | https://www.genecards.org/cgi-bin/carddisp.pl?gene=WNT1 |
| CCR3 | C-C Motif Chemokine Receptor 3 | Protein Coding | P51677 | 59 | GC03P064845 | 1.49003314971924 | https://www.genecards.org/cgi-bin/carddisp.pl?gene=CCR3 |
| FN1 | Fibronectin 1 | Protein Coding | P02751 | 63 | GC02M215360 | 1.4890319108963 | https://www.genecards.org/cgi-bin/carddisp.pl?gene=FN1 |
| CSF3R | Colony Stimulating Factor 3 Receptor | Protein Coding | Q99062 | 60 | GC01M036466 | 1.4890319108963 | https://www.genecards.org/cgi-bin/carddisp.pl?gene=CSF3R |
| ANGPTL6 | Angiopoietin Like 6 | Protein Coding | Q8NI99 | 51 | GC19M010092 | 1.4890319108963 | https://www.genecards.org/cgi-bin/carddisp.pl?gene=ANGPTL6 |
| LRP1 | LDL Receptor Related Protein 1 | Protein Coding | Q07954 | 61 | GC12P057128 | 1.47488796710968 | https://www.genecards.org/cgi-bin/carddisp.pl?gene=LRP1 |
| ABL1 | ABL Proto-Oncogene 1, Non-Receptor Tyrosine Kinase | Protein Coding | P00519 | 65 | GC09P130713 | 1.47135984897614 | https://www.genecards.org/cgi-bin/carddisp.pl?gene=ABL1 |
| BCR | BCR Activator Of RhoGEF And GTPase | Protein Coding | P11274 | 65 | GC22P023179 | 1.47135984897614 | https://www.genecards.org/cgi-bin/carddisp.pl?gene=BCR |
| JAK3 | Janus Kinase 3 | Protein Coding | P52333 | 65 | GC19M017824 | 1.47135984897614 | https://www.genecards.org/cgi-bin/carddisp.pl?gene=JAK3 |
| TGFA | Transforming Growth Factor Alpha | Protein Coding | P01135 | 58 | GC02M070447 | 1.47135984897614 | https://www.genecards.org/cgi-bin/carddisp.pl?gene=TGFA |
| AREG | Amphiregulin | Protein Coding | P15514 | 55 | GC04P074445 | 1.47135984897614 | https://www.genecards.org/cgi-bin/carddisp.pl?gene=AREG |
| LOC107980440 | ABL Breakpoint Recombination Region | Functional Element |  | 8 | GC09P130710 | 1.47135984897614 | https://www.genecards.org/cgi-bin/carddisp.pl?gene=LOC107980440 |
| LOC107963955 | BCR-ABL Major-Breakpoint Cluster Region | Functional Element |  | 7 | GC22P092903 | 1.47135984897614 | https://www.genecards.org/cgi-bin/carddisp.pl?gene=LOC107963955 |
| CBS | Cystathionine Beta-Synthase | Protein Coding | P35520 | 64 | GC21M043053 | 1.4661580324173 | https://www.genecards.org/cgi-bin/carddisp.pl?gene=CBS |
| ITGB3 | Integrin Subunit Beta 3 | Protein Coding | P05106 | 65 | GC17P155043 | 1.46463584899902 | https://www.genecards.org/cgi-bin/carddisp.pl?gene=ITGB3 |
| SPHK1 | Sphingosine Kinase 1 | Protein Coding | Q9NYA1 | 58 | GC17P076376 | 1.46463584899902 | https://www.genecards.org/cgi-bin/carddisp.pl?gene=SPHK1 |
| H6PD | Hexose-6-Phosphate Dehydrogenase/Glucose 1-Dehydrogenase | Protein Coding | O95479 | 56 | GC01P009234 | 1.46463584899902 | https://www.genecards.org/cgi-bin/carddisp.pl?gene=H6PD |
| SREBF2 | Sterol Regulatory Element Binding Transcription Factor 2 | Protein Coding | Q12772 | 55 | GC22P041833 | 1.46463584899902 | https://www.genecards.org/cgi-bin/carddisp.pl?gene=SREBF2 |
| PAPSS1 | 3'-Phosphoadenosine 5'-Phosphosulfate Synthase 1 | Protein Coding | O43252 | 52 | GC04M107590 | 1.46463584899902 | https://www.genecards.org/cgi-bin/carddisp.pl?gene=PAPSS1 |
| ELF3 | E74 Like ETS Transcription Factor 3 | Protein Coding | P78545 | 51 | GC01P202007 | 1.46463584899902 | https://www.genecards.org/cgi-bin/carddisp.pl?gene=ELF3 |
| LSR | Lipolysis Stimulated Lipoprotein Receptor | Protein Coding | Q86X29 | 51 | GC19P152384 | 1.46463584899902 | https://www.genecards.org/cgi-bin/carddisp.pl?gene=LSR |
| EIF2AK2 | Eukaryotic Translation Initiation Factor 2 Alpha Kinase 2 | Protein Coding | P19525 | 62 | GC02M037099 | 1.45200681686401 | https://www.genecards.org/cgi-bin/carddisp.pl?gene=EIF2AK2 |
| PKM | Pyruvate Kinase M1/2 | Protein Coding | P14618 | 62 | GC15M072199 | 1.45200681686401 | https://www.genecards.org/cgi-bin/carddisp.pl?gene=PKM |
| CSF2 | Colony Stimulating Factor 2 | Protein Coding | P04141 | 58 | GC05P132073 | 1.45200681686401 | https://www.genecards.org/cgi-bin/carddisp.pl?gene=CSF2 |
| CCDC88A | Coiled-Coil Domain Containing 88A | Protein Coding | Q3V6T2 | 54 | GC02M055287 | 1.45200681686401 | https://www.genecards.org/cgi-bin/carddisp.pl?gene=CCDC88A |
| CXCL2 | C-X-C Motif Chemokine Ligand 2 | Protein Coding | P19875 | 54 | GC04M074097 | 1.45200681686401 | https://www.genecards.org/cgi-bin/carddisp.pl?gene=CXCL2 |
| DKK2 | Dickkopf WNT Signaling Pathway Inhibitor 2 | Protein Coding | Q9UBU2 | 54 | GC04M106921 | 1.45200681686401 | https://www.genecards.org/cgi-bin/carddisp.pl?gene=DKK2 |
| CCN5 | Cellular Communication Network Factor 5 | Protein Coding | O76076 | 47 | GC20P053994 | 1.45200681686401 | https://www.genecards.org/cgi-bin/carddisp.pl?gene=CCN5 |
| MIR122 | MicroRNA 122 | RNA Gene |  | 30 | GC18P058451 | 1.45200681686401 | https://www.genecards.org/cgi-bin/carddisp.pl?gene=MIR122 |
| LOC110599580 | CYP1B1 Promoter | Functional Element |  | 4 | GC02P038077 | 1.45200681686401 | https://www.genecards.org/cgi-bin/carddisp.pl?gene=LOC110599580 |
| GFAP | Glial Fibrillary Acidic Protein | Protein Coding | P14136 | 61 | GC17M097317 | 1.4433445930481 | https://www.genecards.org/cgi-bin/carddisp.pl?gene=GFAP |
| RGS9 | Regulator Of G Protein Signaling 9 | Protein Coding | O75916 | 54 | GC17P155416 | 1.43867182731628 | https://www.genecards.org/cgi-bin/carddisp.pl?gene=RGS9 |
| F7 | Coagulation Factor VII | Protein Coding | P08709 | 61 | GC13P113105 | 1.43866109848022 | https://www.genecards.org/cgi-bin/carddisp.pl?gene=F7 |
| FYN | FYN Proto-Oncogene, Src Family Tyrosine Kinase | Protein Coding | P06241 | 60 | GC06M111660 | 1.43866109848022 | https://www.genecards.org/cgi-bin/carddisp.pl?gene=FYN |
| PRLR | Prolactin Receptor | Protein Coding | P16471 | 60 | GC05M035048 | 1.43866109848022 | https://www.genecards.org/cgi-bin/carddisp.pl?gene=PRLR |
| COL6A3 | Collagen Type VI Alpha 3 Chain | Protein Coding | P12111 | 57 | GC02M237324 | 1.43866109848022 | https://www.genecards.org/cgi-bin/carddisp.pl?gene=COL6A3 |
| ITGB5 | Integrin Subunit Beta 5 | Protein Coding | P18084 | 56 | GC03M124761 | 1.43866109848022 | https://www.genecards.org/cgi-bin/carddisp.pl?gene=ITGB5 |
| DGKQ | Diacylglycerol Kinase Theta | Protein Coding | P52824 | 54 | GC04M000958 | 1.43866109848022 | https://www.genecards.org/cgi-bin/carddisp.pl?gene=DGKQ |
| PSMC6 | Proteasome 26S Subunit, ATPase 6 | Protein Coding | P62333 | 52 | GC14P052707 | 1.43866109848022 | https://www.genecards.org/cgi-bin/carddisp.pl?gene=PSMC6 |
| CAVIN1 | Caveolae Associated Protein 1 | Protein Coding | Q6NZI2 | 54 | GC17M097212 | 1.43648362159729 | https://www.genecards.org/cgi-bin/carddisp.pl?gene=CAVIN1 |
| PDGFRA | Platelet Derived Growth Factor Receptor Alpha | Protein Coding | P16234 | 66 | GC04P054229 | 1.43189406394958 | https://www.genecards.org/cgi-bin/carddisp.pl?gene=PDGFRA |
| DDIT3 | DNA Damage Inducible Transcript 3 | Protein Coding | P35638 | 59 | GC12M057516 | 1.43189406394958 | https://www.genecards.org/cgi-bin/carddisp.pl?gene=DDIT3 |
| ADRA2A | Adrenoceptor Alpha 2A | Protein Coding | P08913 | 60 | GC10P111077 | 1.43036925792694 | https://www.genecards.org/cgi-bin/carddisp.pl?gene=ADRA2A |
| PDGFRB | Platelet Derived Growth Factor Receptor Beta | Protein Coding | P09619 | 67 | GC05M150113 | 1.41092431545258 | https://www.genecards.org/cgi-bin/carddisp.pl?gene=PDGFRB |
| MYC | MYC Proto-Oncogene, BHLH Transcription Factor | Protein Coding | P01106 | 65 | GC08P127735 | 1.41092431545258 | https://www.genecards.org/cgi-bin/carddisp.pl?gene=MYC |
| HSP90AA1 | Heat Shock Protein 90 Alpha Family Class A Member 1 | Protein Coding | P07900 | 64 | GC14M102080 | 1.41092431545258 | https://www.genecards.org/cgi-bin/carddisp.pl?gene=HSP90AA1 |
| TERT | Telomerase Reverse Transcriptase | Protein Coding | O14746 | 64 | GC05M001253 | 1.41092431545258 | https://www.genecards.org/cgi-bin/carddisp.pl?gene=TERT |
| KAT5 | Lysine Acetyltransferase 5 | Protein Coding | Q92993 | 63 | GC11P065711 | 1.41092431545258 | https://www.genecards.org/cgi-bin/carddisp.pl?gene=KAT5 |
| CASP9 | Caspase 9 | Protein Coding | P55211 | 61 | GC01M022575 | 1.41092431545258 | https://www.genecards.org/cgi-bin/carddisp.pl?gene=CASP9 |
| ABCB7 | ATP Binding Cassette Subfamily B Member 7 | Protein Coding | O75027 | 57 | GC0XM075053 | 1.41092431545258 | https://www.genecards.org/cgi-bin/carddisp.pl?gene=ABCB7 |
| MAPKAP1 | MAPK Associated Protein 1 | Protein Coding | Q9BPZ7 | 57 | GC09M125437 | 1.41092431545258 | https://www.genecards.org/cgi-bin/carddisp.pl?gene=MAPKAP1 |
| YME1L1 | YME1 Like 1 ATPase | Protein Coding | Q96TA2 | 55 | GC10M027110 | 1.41092431545258 | https://www.genecards.org/cgi-bin/carddisp.pl?gene=YME1L1 |
| AKAP5 | A-Kinase Anchoring Protein 5 | Protein Coding | P24588 | 51 | GC14P064465 | 1.41092431545258 | https://www.genecards.org/cgi-bin/carddisp.pl?gene=AKAP5 |
| MAT2B | Methionine Adenosyltransferase 2 Non-Catalytic Beta Subunit | Protein Coding | Q9NZL9 | 51 | GC05P163504 | 1.41092431545258 | https://www.genecards.org/cgi-bin/carddisp.pl?gene=MAT2B |
| CGB7 | Chorionic Gonadotropin Subunit Beta 7 | Protein Coding | P0DN87 | 41 | GC19M049054 | 1.41092431545258 | https://www.genecards.org/cgi-bin/carddisp.pl?gene=CGB7 |
| FAS-AS1 | FAS Antisense RNA 1 | RNA Gene |  | 21 | GC10M088991 | 1.41092431545258 | https://www.genecards.org/cgi-bin/carddisp.pl?gene=FAS-AS1 |
| NCOA3 | Nuclear Receptor Coactivator 3 | Protein Coding | Q9Y6Q9 | 60 | GC20P047501 | 1.41075468063354 | https://www.genecards.org/cgi-bin/carddisp.pl?gene=NCOA3 |
| SIK2 | Salt Inducible Kinase 2 | Protein Coding | Q9H0K1 | 58 | GC11P113445 | 1.41075468063354 | https://www.genecards.org/cgi-bin/carddisp.pl?gene=SIK2 |
| NCOA2 | Nuclear Receptor Coactivator 2 | Protein Coding | Q15596 | 57 | GC08M070109 | 1.41075468063354 | https://www.genecards.org/cgi-bin/carddisp.pl?gene=NCOA2 |
| GRIP1 | Glutamate Receptor Interacting Protein 1 | Protein Coding | Q9Y3R0 | 56 | GC12M066347 | 1.41075468063354 | https://www.genecards.org/cgi-bin/carddisp.pl?gene=GRIP1 |
| PELP1 | Proline, Glutamate And Leucine Rich Protein 1 | Protein Coding | Q8IZL8 | 50 | GC17M004669 | 1.41075468063354 | https://www.genecards.org/cgi-bin/carddisp.pl?gene=PELP1 |
| GPR182 | G Protein-Coupled Receptor 182 | Protein Coding | O15218 | 48 | GC12P056994 | 1.41075468063354 | https://www.genecards.org/cgi-bin/carddisp.pl?gene=GPR182 |
| FGF23 | Fibroblast Growth Factor 23 | Protein Coding | Q9GZV9 | 58 | GC12M004368 | 1.40460395812988 | https://www.genecards.org/cgi-bin/carddisp.pl?gene=FGF23 |
| VPS52 | VPS52 Subunit Of GARP Complex | Protein Coding | Q8N1B4 | 47 | GC06M106578 | 1.40322160720825 | https://www.genecards.org/cgi-bin/carddisp.pl?gene=VPS52 |
| MIR200C | MicroRNA 200c | RNA Gene |  | 31 | GC12P074084 | 1.40322160720825 | https://www.genecards.org/cgi-bin/carddisp.pl?gene=MIR200C |
| APOC2 | Apolipoprotein C2 | Protein Coding | P02655 | 57 | GC19P152701 | 1.39226949214935 | https://www.genecards.org/cgi-bin/carddisp.pl?gene=APOC2 |
| PRKACA | Protein Kinase CAMP-Activated Catalytic Subunit Alpha | Protein Coding | P17612 | 65 | GC19M108254 | 1.3889776468277 | https://www.genecards.org/cgi-bin/carddisp.pl?gene=PRKACA |
| WNT5A | Wnt Family Member 5A | Protein Coding | P41221 | 64 | GC03M055465 | 1.3889776468277 | https://www.genecards.org/cgi-bin/carddisp.pl?gene=WNT5A |
| HMGB1 | High Mobility Group Box 1 | Protein Coding | P09429 | 62 | GC13M030456 | 1.3889776468277 | https://www.genecards.org/cgi-bin/carddisp.pl?gene=HMGB1 |
| SETD2 | SET Domain Containing 2, Histone Lysine Methyltransferase | Protein Coding | Q9BYW2 | 60 | GC03M047033 | 1.3889776468277 | https://www.genecards.org/cgi-bin/carddisp.pl?gene=SETD2 |
| SFTPB | Surfactant Protein B | Protein Coding | P07988 | 56 | GC02M085657 | 1.3889776468277 | https://www.genecards.org/cgi-bin/carddisp.pl?gene=SFTPB |
| KIF1B | Kinesin Family Member 1B | Protein Coding | O60333 | 55 | GC01P010210 | 1.3889776468277 | https://www.genecards.org/cgi-bin/carddisp.pl?gene=KIF1B |
| NCOA6 | Nuclear Receptor Coactivator 6 | Protein Coding | Q14686 | 52 | GC20M036811 | 1.3889776468277 | https://www.genecards.org/cgi-bin/carddisp.pl?gene=NCOA6 |
| GTF2F1 | General Transcription Factor IIF Subunit 1 | Protein Coding | P35269 | 45 | GC19M107954 | 1.3889776468277 | https://www.genecards.org/cgi-bin/carddisp.pl?gene=GTF2F1 |
| TRG-GCC2-6 | TRNA-Gly (Anticodon GCC) 2-6 | RNA Gene |  | 10 | GC17P153781 | 1.3889776468277 | https://www.genecards.org/cgi-bin/carddisp.pl?gene=TRG-GCC2-6 |
| TRG-TCC1-1 | TRNA-Gly (Anticodon TCC) 1-1 | RNA Gene |  | 10 | GC19P151533 | 1.3889776468277 | https://www.genecards.org/cgi-bin/carddisp.pl?gene=TRG-TCC1-1 |
| TRG-CCC2-2 | TRNA-Gly (Anticodon CCC) 2-2 | RNA Gene |  | 9 | GC16M000637 | 1.3889776468277 | https://www.genecards.org/cgi-bin/carddisp.pl?gene=TRG-CCC2-2 |
| TRG-GCC3-1 | TRNA-Gly (Anticodon GCC) 3-1 | RNA Gene |  | 8 | GC16M070778 | 1.3889776468277 | https://www.genecards.org/cgi-bin/carddisp.pl?gene=TRG-GCC3-1 |
| CDK5 | Cyclin Dependent Kinase 5 | Protein Coding | Q00535 | 66 | GC07M151053 | 1.38040804862976 | https://www.genecards.org/cgi-bin/carddisp.pl?gene=CDK5 |
| XIAP | X-Linked Inhibitor Of Apoptosis | Protein Coding | P98170 | 64 | GC0XP123859 | 1.38040804862976 | https://www.genecards.org/cgi-bin/carddisp.pl?gene=XIAP |
| PTGDS | Prostaglandin D2 Synthase | Protein Coding | P41222 | 59 | GC09P153331 | 1.38040804862976 | https://www.genecards.org/cgi-bin/carddisp.pl?gene=PTGDS |
| CDK5R1 | Cyclin Dependent Kinase 5 Regulatory Subunit 1 | Protein Coding | Q15078 | 58 | GC17P032486 | 1.38040804862976 | https://www.genecards.org/cgi-bin/carddisp.pl?gene=CDK5R1 |
| SORT1 | Sortilin 1 | Protein Coding | Q99523 | 58 | GC01M109310 | 1.38040804862976 | https://www.genecards.org/cgi-bin/carddisp.pl?gene=SORT1 |
| USP2 | Ubiquitin Specific Peptidase 2 | Protein Coding | O75604 | 55 | GC11M119355 | 1.38040804862976 | https://www.genecards.org/cgi-bin/carddisp.pl?gene=USP2 |
| TSHB | Thyroid Stimulating Hormone Subunit Beta | Protein Coding | P01222 | 54 | GC01P115029 | 1.38040804862976 | https://www.genecards.org/cgi-bin/carddisp.pl?gene=TSHB |
| GAS5 | Growth Arrest Specific 5 | RNA Gene |  | 32 | GC01M175146 | 1.38040804862976 | https://www.genecards.org/cgi-bin/carddisp.pl?gene=GAS5 |
| CYBB | Cytochrome B-245 Beta Chain | Protein Coding | P04839 | 61 | GC0XP037780 | 1.36590325832367 | https://www.genecards.org/cgi-bin/carddisp.pl?gene=CYBB |
| NCF2 | Neutrophil Cytosolic Factor 2 | Protein Coding | P19878 | 61 | GC01M186893 | 1.36590325832367 | https://www.genecards.org/cgi-bin/carddisp.pl?gene=NCF2 |
| PPP1CA | Protein Phosphatase 1 Catalytic Subunit Alpha | Protein Coding | P62136 | 61 | GC11M139864 | 1.36590325832367 | https://www.genecards.org/cgi-bin/carddisp.pl?gene=PPP1CA |
| EIF4G1 | Eukaryotic Translation Initiation Factor 4 Gamma 1 | Protein Coding | Q04637 | 60 | GC03P184314 | 1.36590325832367 | https://www.genecards.org/cgi-bin/carddisp.pl?gene=EIF4G1 |
| ARNT | Aryl Hydrocarbon Receptor Nuclear Translocator | Protein Coding | P27540 | 58 | GC01M150809 | 1.36590325832367 | https://www.genecards.org/cgi-bin/carddisp.pl?gene=ARNT |
| F3 | Coagulation Factor III, Tissue Factor | Protein Coding | P13726 | 58 | GC01M095040 | 1.36590325832367 | https://www.genecards.org/cgi-bin/carddisp.pl?gene=F3 |
| ICOS | Inducible T Cell Costimulator | Protein Coding | Q9Y6W8 | 57 | GC02P204862 | 1.36590325832367 | https://www.genecards.org/cgi-bin/carddisp.pl?gene=ICOS |
| IGFBP5 | Insulin Like Growth Factor Binding Protein 5 | Protein Coding | P24593 | 55 | GC02M216672 | 1.36590325832367 | https://www.genecards.org/cgi-bin/carddisp.pl?gene=IGFBP5 |
| NOX4 | NADPH Oxidase 4 | Protein Coding | Q9NPH5 | 55 | GC11M089324 | 1.36590325832367 | https://www.genecards.org/cgi-bin/carddisp.pl?gene=NOX4 |
| CXCR5 | C-X-C Motif Chemokine Receptor 5 | Protein Coding | P32302 | 54 | GC11P125200 | 1.36590325832367 | https://www.genecards.org/cgi-bin/carddisp.pl?gene=CXCR5 |
| UHRF1 | Ubiquitin Like With PHD And Ring Finger Domains 1 | Protein Coding | Q96T88 | 53 | GC19P151545 | 1.36590325832367 | https://www.genecards.org/cgi-bin/carddisp.pl?gene=UHRF1 |
| NHLH2 | Nescient Helix-Loop-Helix 2 | Protein Coding | Q02577 | 49 | GC01M115836 | 1.36590325832367 | https://www.genecards.org/cgi-bin/carddisp.pl?gene=NHLH2 |
| GNRH2 | Gonadotropin Releasing Hormone 2 | Protein Coding | O43555 | 44 | GC20P003043 | 1.36590325832367 | https://www.genecards.org/cgi-bin/carddisp.pl?gene=GNRH2 |
| MIR182 | MicroRNA 182 | RNA Gene |  | 30 | GC07M129770 | 1.36590325832367 | https://www.genecards.org/cgi-bin/carddisp.pl?gene=MIR182 |
| MIR27B | MicroRNA 27b | RNA Gene |  | 30 | GC09P107001 | 1.36590325832367 | https://www.genecards.org/cgi-bin/carddisp.pl?gene=MIR27B |
| MIR96 | MicroRNA 96 | RNA Gene |  | 30 | GC07M129774 | 1.36590325832367 | https://www.genecards.org/cgi-bin/carddisp.pl?gene=MIR96 |
| SLC30A8 | Solute Carrier Family 30 Member 8 | Protein Coding | Q8IWU4 | 54 | GC08P116950 | 1.35802817344666 | https://www.genecards.org/cgi-bin/carddisp.pl?gene=SLC30A8 |
| PLCG1 | Phospholipase C Gamma 1 | Protein Coding | P19174 | 62 | GC20P041136 | 1.34684157371521 | https://www.genecards.org/cgi-bin/carddisp.pl?gene=PLCG1 |
| SHC1 | SHC Adaptor Protein 1 | Protein Coding | P29353 | 57 | GC01M154962 | 1.34684157371521 | https://www.genecards.org/cgi-bin/carddisp.pl?gene=SHC1 |
| TRPC4 | Transient Receptor Potential Cation Channel Subfamily C Member 4 | Protein Coding | Q9UBN4 | 56 | GC13M043034 | 1.34684157371521 | https://www.genecards.org/cgi-bin/carddisp.pl?gene=TRPC4 |
| ABCG1 | ATP Binding Cassette Subfamily G Member 1 | Protein Coding | P45844 | 55 | GC21P042199 | 1.34684157371521 | https://www.genecards.org/cgi-bin/carddisp.pl?gene=ABCG1 |
| SIGLEC1 | Sialic Acid Binding Ig Like Lectin 1 | Protein Coding | Q9BZZ2 | 52 | GC20M003686 | 1.34684157371521 | https://www.genecards.org/cgi-bin/carddisp.pl?gene=SIGLEC1 |
| PRLH | Prolactin Releasing Hormone | Protein Coding | P81277 | 36 | GC02P237566 | 1.34684157371521 | https://www.genecards.org/cgi-bin/carddisp.pl?gene=PRLH |
| ITGB2 | Integrin Subunit Beta 2 | Protein Coding | P05107 | 63 | GC21M044885 | 1.34150731563568 | https://www.genecards.org/cgi-bin/carddisp.pl?gene=ITGB2 |
| NOS1 | Nitric Oxide Synthase 1 | Protein Coding | P29475 | 62 | GC12M117208 | 1.34150731563568 | https://www.genecards.org/cgi-bin/carddisp.pl?gene=NOS1 |
| BACE1 | Beta-Secretase 1 | Protein Coding | P56817 | 60 | GC11M117285 | 1.34150731563568 | https://www.genecards.org/cgi-bin/carddisp.pl?gene=BACE1 |
| EPHA3 | EPH Receptor A3 | Protein Coding | P29320 | 60 | GC03P089077 | 1.34150731563568 | https://www.genecards.org/cgi-bin/carddisp.pl?gene=EPHA3 |
| MAPK11 | Mitogen-Activated Protein Kinase 11 | Protein Coding | Q15759 | 60 | GC22M050263 | 1.34150731563568 | https://www.genecards.org/cgi-bin/carddisp.pl?gene=MAPK11 |
| MMP8 | Matrix Metallopeptidase 8 | Protein Coding | P22894 | 60 | GC11M140389 | 1.34150731563568 | https://www.genecards.org/cgi-bin/carddisp.pl?gene=MMP8 |
| ATF1 | Activating Transcription Factor 1 | Protein Coding | P18846 | 58 | GC12P050763 | 1.34150731563568 | https://www.genecards.org/cgi-bin/carddisp.pl?gene=ATF1 |
| GAD2 | Glutamate Decarboxylase 2 | Protein Coding | Q05329 | 57 | GC10P026216 | 1.34150731563568 | https://www.genecards.org/cgi-bin/carddisp.pl?gene=GAD2 |
| SMAD7 | SMAD Family Member 7 | Protein Coding | O15105 | 57 | GC18M048919 | 1.34150731563568 | https://www.genecards.org/cgi-bin/carddisp.pl?gene=SMAD7 |
| SELL | Selectin L | Protein Coding | P14151 | 56 | GC01M169690 | 1.34150731563568 | https://www.genecards.org/cgi-bin/carddisp.pl?gene=SELL |
| SRF | Serum Response Factor | Protein Coding | P11831 | 56 | GC06P043171 | 1.34150731563568 | https://www.genecards.org/cgi-bin/carddisp.pl?gene=SRF |
| ICAM3 | Intercellular Adhesion Molecule 3 | Protein Coding | P32942 | 54 | GC19M108113 | 1.34150731563568 | https://www.genecards.org/cgi-bin/carddisp.pl?gene=ICAM3 |
| CCL1 | C-C Motif Chemokine Ligand 1 | Protein Coding | P22362 | 50 | GC17M096837 | 1.34150731563568 | https://www.genecards.org/cgi-bin/carddisp.pl?gene=CCL1 |
| ERVW-1 | Endogenous Retrovirus Group W Member 1, Envelope | Protein Coding | Q9UQF0 | 47 | GC07M092468 | 1.34150731563568 | https://www.genecards.org/cgi-bin/carddisp.pl?gene=ERVW-1 |
| MIR98 | MicroRNA 98 | RNA Gene |  | 27 | GC0XM053998 | 1.34150731563568 | https://www.genecards.org/cgi-bin/carddisp.pl?gene=MIR98 |
| KLF14 | KLF Transcription Factor 14 | Protein Coding | Q8TD94 | 43 | GC07M130731 | 1.33481335639954 | https://www.genecards.org/cgi-bin/carddisp.pl?gene=KLF14 |
| CDK2 | Cyclin Dependent Kinase 2 | Protein Coding | P24941 | 64 | GC12P055966 | 1.31553256511688 | https://www.genecards.org/cgi-bin/carddisp.pl?gene=CDK2 |
| CTSB | Cathepsin B | Protein Coding | P07858 | 64 | GC08M011842 | 1.31553256511688 | https://www.genecards.org/cgi-bin/carddisp.pl?gene=CTSB |
| MMP7 | Matrix Metallopeptidase 7 | Protein Coding | P09237 | 60 | GC11M140388 | 1.31553256511688 | https://www.genecards.org/cgi-bin/carddisp.pl?gene=MMP7 |
| SCNN1G | Sodium Channel Epithelial 1 Subunit Gamma | Protein Coding | P51170 | 60 | GC16P023182 | 1.31553256511688 | https://www.genecards.org/cgi-bin/carddisp.pl?gene=SCNN1G |
| GPC3 | Glypican 3 | Protein Coding | P51654 | 59 | GC0XM133535 | 1.31553256511688 | https://www.genecards.org/cgi-bin/carddisp.pl?gene=GPC3 |
| TET2 | Tet Methylcytosine Dioxygenase 2 | Protein Coding | Q6N021 | 58 | GC04P105145 | 1.31553256511688 | https://www.genecards.org/cgi-bin/carddisp.pl?gene=TET2 |
| PTPRN | Protein Tyrosine Phosphatase Receptor Type N | Protein Coding | Q16849 | 57 | GC02M219289 | 1.31553256511688 | https://www.genecards.org/cgi-bin/carddisp.pl?gene=PTPRN |
| TGIF1 | TGFB Induced Factor Homeobox 1 | Protein Coding | Q15583 | 57 | GC18P003411 | 1.31553256511688 | https://www.genecards.org/cgi-bin/carddisp.pl?gene=TGIF1 |
| CXCL1 | C-X-C Motif Chemokine Ligand 1 | Protein Coding | P09341 | 56 | GC04P073869 | 1.31553256511688 | https://www.genecards.org/cgi-bin/carddisp.pl?gene=CXCL1 |
| SRD5A1 | Steroid 5 Alpha-Reductase 1 | Protein Coding | P18405 | 56 | GC05P006633 | 1.31553256511688 | https://www.genecards.org/cgi-bin/carddisp.pl?gene=SRD5A1 |
| AQP9 | Aquaporin 9 | Protein Coding | O43315 | 55 | GC15P058138 | 1.31553256511688 | https://www.genecards.org/cgi-bin/carddisp.pl?gene=AQP9 |
| CYP21A2 | Cytochrome P450 Family 21 Subfamily A Member 2 | Protein Coding | P08686 | 55 | GC06P181915 | 1.31553256511688 | https://www.genecards.org/cgi-bin/carddisp.pl?gene=CYP21A2 |
| HTR1B | 5-Hydroxytryptamine Receptor 1B | Protein Coding | P28222 | 55 | GC06M107837 | 1.31553256511688 | https://www.genecards.org/cgi-bin/carddisp.pl?gene=HTR1B |
| PDCD4 | Programmed Cell Death 4 | Protein Coding | Q53EL6 | 55 | GC10P110871 | 1.31553256511688 | https://www.genecards.org/cgi-bin/carddisp.pl?gene=PDCD4 |
| DUSP19 | Dual Specificity Phosphatase 19 | Protein Coding | Q8WTR2 | 49 | GC02P183078 | 1.31553256511688 | https://www.genecards.org/cgi-bin/carddisp.pl?gene=DUSP19 |
| CCL27 | C-C Motif Chemokine Ligand 27 | Protein Coding | Q9Y4X3 | 47 | GC09M035856 | 1.31553256511688 | https://www.genecards.org/cgi-bin/carddisp.pl?gene=CCL27 |
| MIR1301 | MicroRNA 1301 | RNA Gene |  | 17 | GC02M025328 | 1.31553256511688 | https://www.genecards.org/cgi-bin/carddisp.pl?gene=MIR1301 |
| C16orf74 | Chromosome 16 Open Reading Frame 74 | Protein Coding | Q96GX8 | 34 | GC16M085690 | 1.31020414829254 | https://www.genecards.org/cgi-bin/carddisp.pl?gene=C16orf74 |
| IFNB1 | Interferon Beta 1 | Protein Coding | P01574 | 55 | GC09M021077 | 1.30874168872833 | https://www.genecards.org/cgi-bin/carddisp.pl?gene=IFNB1 |
| MIR378A | MicroRNA 378a | RNA Gene |  | 31 | GC05P149732 | 1.30874168872833 | https://www.genecards.org/cgi-bin/carddisp.pl?gene=MIR378A |
| MIR451A | MicroRNA 451a | RNA Gene |  | 25 | GC17M028861 | 1.30874168872833 | https://www.genecards.org/cgi-bin/carddisp.pl?gene=MIR451A |
| IGHJ1 | Immunoglobulin Heavy Joining 1 | Protein Coding | A0A0C4DH62 | 17 | GC14M124490 | 1.30874168872833 | https://www.genecards.org/cgi-bin/carddisp.pl?gene=IGHJ1 |
| NFE2L2 | NFE2 Like BZIP Transcription Factor 2 | Protein Coding | Q16236 | 64 | GC02M177227 | 1.28762602806091 | https://www.genecards.org/cgi-bin/carddisp.pl?gene=NFE2L2 |
| PRKCA | Protein Kinase C Alpha | Protein Coding | P17252 | 64 | GC17P066302 | 1.28762602806091 | https://www.genecards.org/cgi-bin/carddisp.pl?gene=PRKCA |
| KCNMA1 | Potassium Calcium-Activated Channel Subfamily M Alpha 1 | Protein Coding | Q12791 | 61 | GC10M076869 | 1.28762602806091 | https://www.genecards.org/cgi-bin/carddisp.pl?gene=KCNMA1 |
| CBFB | Core-Binding Factor Subunit Beta | Protein Coding | Q13951 | 60 | GC16P121838 | 1.28762602806091 | https://www.genecards.org/cgi-bin/carddisp.pl?gene=CBFB |
| CDH5 | Cadherin 5 | Protein Coding | P33151 | 60 | GC16P066366 | 1.28762602806091 | https://www.genecards.org/cgi-bin/carddisp.pl?gene=CDH5 |
| IL12B | Interleukin 12B | Protein Coding | P29460 | 57 | GC05M159314 | 1.28762602806091 | https://www.genecards.org/cgi-bin/carddisp.pl?gene=IL12B |
| PHB2 | Prohibitin 2 | Protein Coding | Q99623 | 56 | GC12M006965 | 1.28762602806091 | https://www.genecards.org/cgi-bin/carddisp.pl?gene=PHB2 |
| SRA1 | Steroid Receptor RNA Activator 1 | Protein Coding | Q9HD15 | 49 | GC05M140537 | 1.28762602806091 | https://www.genecards.org/cgi-bin/carddisp.pl?gene=SRA1 |
| BBS12 | Bardet-Biedl Syndrome 12 | Protein Coding | Q6ZW61 | 46 | GC04P122702 | 1.28762602806091 | https://www.genecards.org/cgi-bin/carddisp.pl?gene=BBS12 |
| MIR33B | MicroRNA 33b | RNA Gene |  | 27 | GC17M017813 | 1.28762602806091 | https://www.genecards.org/cgi-bin/carddisp.pl?gene=MIR33B |
| MIR539 | MicroRNA 539 | RNA Gene |  | 26 | GC14P121387 | 1.28762602806091 | https://www.genecards.org/cgi-bin/carddisp.pl?gene=MIR539 |
| FMR1 | Fragile X Messenger Ribonucleoprotein 1 | Protein Coding | Q06787 | 57 | GC0XP148000 | 1.26758766174316 | https://www.genecards.org/cgi-bin/carddisp.pl?gene=FMR1 |
| USP7 | Ubiquitin Specific Peptidase 7 | Protein Coding | Q93009 | 62 | GC16M008892 | 1.26354837417603 | https://www.genecards.org/cgi-bin/carddisp.pl?gene=USP7 |
| EHMT2 | Euchromatic Histone Lysine Methyltransferase 2 | Protein Coding | Q96KQ7 | 58 | GC06M031879 | 1.26354837417603 | https://www.genecards.org/cgi-bin/carddisp.pl?gene=EHMT2 |
| FABP7 | Fatty Acid Binding Protein 7 | Protein Coding | O15540 | 56 | GC06P182970 | 1.26354837417603 | https://www.genecards.org/cgi-bin/carddisp.pl?gene=FABP7 |
| APOD | Apolipoprotein D | Protein Coding | P05090 | 55 | GC03M195568 | 1.26354837417603 | https://www.genecards.org/cgi-bin/carddisp.pl?gene=APOD |
| CD68 | CD68 Molecule | Protein Coding | P34810 | 52 | GC17P007579 | 1.26354837417603 | https://www.genecards.org/cgi-bin/carddisp.pl?gene=CD68 |
| TRIM27 | Tripartite Motif Containing 27 | Protein Coding | P14373 | 52 | GC06M028903 | 1.26354837417603 | https://www.genecards.org/cgi-bin/carddisp.pl?gene=TRIM27 |
| ATR | ATR Serine/Threonine Kinase | Protein Coding | Q13535 | 67 | GC03M142449 | 1.25727951526642 | https://www.genecards.org/cgi-bin/carddisp.pl?gene=ATR |
| RPS6KA1 | Ribosomal Protein S6 Kinase A1 | Protein Coding | Q15418 | 63 | GC01P076421 | 1.25727951526642 | https://www.genecards.org/cgi-bin/carddisp.pl?gene=RPS6KA1 |
| TPH2 | Tryptophan Hydroxylase 2 | Protein Coding | Q8IWU9 | 62 | GC12P071938 | 1.25727951526642 | https://www.genecards.org/cgi-bin/carddisp.pl?gene=TPH2 |
| TXN | Thioredoxin | Protein Coding | P10599 | 59 | GC09M110243 | 1.25727951526642 | https://www.genecards.org/cgi-bin/carddisp.pl?gene=TXN |
| LOXL3 | Lysyl Oxidase Like 3 | Protein Coding | P58215 | 56 | GC02M074532 | 1.25727951526642 | https://www.genecards.org/cgi-bin/carddisp.pl?gene=LOXL3 |
| SLPI | Secretory Leukocyte Peptidase Inhibitor | Protein Coding | P03973 | 53 | GC20M045252 | 1.25727951526642 | https://www.genecards.org/cgi-bin/carddisp.pl?gene=SLPI |
| SH2D4B | SH2 Domain Containing 4B | Protein Coding | Q5SQS7 | 40 | GC10P123927 | 1.25727951526642 | https://www.genecards.org/cgi-bin/carddisp.pl?gene=SH2D4B |
| HNF1A-AS1 | HNF1A Antisense RNA 1 | RNA Gene |  | 28 | GC12M124665 | 1.25727951526642 | https://www.genecards.org/cgi-bin/carddisp.pl?gene=HNF1A-AS1 |
| LINC01565 | Long Intergenic Non-Protein Coding RNA 1565 | RNA Gene | O15544 | 28 | GC03M132421 | 1.25727951526642 | https://www.genecards.org/cgi-bin/carddisp.pl?gene=LINC01565 |
| APP | Amyloid Beta Precursor Protein | Protein Coding | P05067 | 63 | GC21M025880 | 1.24854958057404 | https://www.genecards.org/cgi-bin/carddisp.pl?gene=APP |
| COBLL1 | Cordon-Bleu WH2 Repeat Protein Like 1 | Protein Coding | Q53SF7 | 45 | GC02M164654 | 1.23864221572876 | https://www.genecards.org/cgi-bin/carddisp.pl?gene=COBLL1 |
| SNX1 | Sorting Nexin 1 | Protein Coding | Q13596 | 53 | GC15P064094 | 1.2354222536087 | https://www.genecards.org/cgi-bin/carddisp.pl?gene=SNX1 |
| SNX2 | Sorting Nexin 2 | Protein Coding | O60749 | 50 | GC05P122774 | 1.2354222536087 | https://www.genecards.org/cgi-bin/carddisp.pl?gene=SNX2 |
| IRX3 | Iroquois Homeobox 3 | Protein Coding | P78415 | 47 | GC16M054283 | 1.23200821876526 | https://www.genecards.org/cgi-bin/carddisp.pl?gene=IRX3 |
| PRKCD | Protein Kinase C Delta | Protein Coding | Q05655 | 65 | GC03P053156 | 1.22371292114258 | https://www.genecards.org/cgi-bin/carddisp.pl?gene=PRKCD |
| ACTB | Actin Beta | Protein Coding | P60709 | 63 | GC07M005854 | 1.22371292114258 | https://www.genecards.org/cgi-bin/carddisp.pl?gene=ACTB |
| JAG1 | Jagged Canonical Notch Ligand 1 | Protein Coding | P78504 | 63 | GC20M010637 | 1.22371292114258 | https://www.genecards.org/cgi-bin/carddisp.pl?gene=JAG1 |
| KL | Klotho | Protein Coding | Q9UEF7 | 61 | GC13P033016 | 1.22371292114258 | https://www.genecards.org/cgi-bin/carddisp.pl?gene=KL |
| ATF4 | Activating Transcription Factor 4 | Protein Coding | P18848 | 60 | GC22P039519 | 1.22371292114258 | https://www.genecards.org/cgi-bin/carddisp.pl?gene=ATF4 |
| KCNB1 | Potassium Voltage-Gated Channel Subfamily B Member 1 | Protein Coding | Q14721 | 60 | GC20M049293 | 1.22371292114258 | https://www.genecards.org/cgi-bin/carddisp.pl?gene=KCNB1 |
| PIK3C2A | Phosphatidylinositol-4-Phosphate 3-Kinase Catalytic Subunit Type 2 Alpha | Protein Coding | O00443 | 60 | GC11M018151 | 1.22371292114258 | https://www.genecards.org/cgi-bin/carddisp.pl?gene=PIK3C2A |
| VTN | Vitronectin | Protein Coding | P04004 | 57 | GC17M096696 | 1.22371292114258 | https://www.genecards.org/cgi-bin/carddisp.pl?gene=VTN |
| FOSL2 | FOS Like 2, AP-1 Transcription Factor Subunit | Protein Coding | P15408 | 55 | GC02P028392 | 1.22371292114258 | https://www.genecards.org/cgi-bin/carddisp.pl?gene=FOSL2 |
| PPBP | Pro-Platelet Basic Protein | Protein Coding | P02775 | 55 | GC04M073986 | 1.22371292114258 | https://www.genecards.org/cgi-bin/carddisp.pl?gene=PPBP |
| RPS27 | Ribosomal Protein S27 | Protein Coding | P42677 | 55 | GC01P176670 | 1.22371292114258 | https://www.genecards.org/cgi-bin/carddisp.pl?gene=RPS27 |
| IL17F | Interleukin 17F | Protein Coding | Q96PD4 | 54 | GC06M106847 | 1.22371292114258 | https://www.genecards.org/cgi-bin/carddisp.pl?gene=IL17F |
| ARMS2 | Age-Related Maculopathy Susceptibility 2 | Protein Coding | P0C7Q2 | 41 | GC10P122454 | 1.22371292114258 | https://www.genecards.org/cgi-bin/carddisp.pl?gene=ARMS2 |
| MIR519D | MicroRNA 519d | RNA Gene |  | 25 | GC19P053713 | 1.22371292114258 | https://www.genecards.org/cgi-bin/carddisp.pl?gene=MIR519D |
| ADIPQTL2 | Circulating Adiponectin QTL On Chromosome 5 | Genetic Locus |  | 2 | GC05U901929 | 1.22371292114258 | https://www.genecards.org/cgi-bin/carddisp.pl?gene=ADIPQTL2 |
| AIR | Acute Insulin Response (2) | Genetic Locus |  | 2 | GC01U990199 | 1.22371292114258 | https://www.genecards.org/cgi-bin/carddisp.pl?gene=AIR |
| COMT | Catechol-O-Methyltransferase | Protein Coding | P21964 | 64 | GC22P019941 | 1.21304368972778 | https://www.genecards.org/cgi-bin/carddisp.pl?gene=COMT |
| MIR210 | MicroRNA 210 | RNA Gene |  | 30 | GC11M015053 | 1.20465111732483 | https://www.genecards.org/cgi-bin/carddisp.pl?gene=MIR210 |
| HTT | Huntingtin | Protein Coding | P42858 | 57 | GC04P003041 | 1.20335614681244 | https://www.genecards.org/cgi-bin/carddisp.pl?gene=HTT |
| HNRNPUL2-BSCL2 | HNRNPUL2-BSCL2 Readthrough (NMD Candidate) | RNA Gene |  | 25 | GC11M139666 | 1.19702363014221 | https://www.genecards.org/cgi-bin/carddisp.pl?gene=HNRNPUL2-BSCL2 |
| GNB3 | G Protein Subunit Beta 3 | Protein Coding | P16520 | 60 | GC12P006839 | 1.19678819179535 | https://www.genecards.org/cgi-bin/carddisp.pl?gene=GNB3 |
| BMP4 | Bone Morphogenetic Protein 4 | Protein Coding | P12644 | 62 | GC14M053949 | 1.18561315536499 | https://www.genecards.org/cgi-bin/carddisp.pl?gene=BMP4 |
| PLOD2 | Procollagen-Lysine,2-Oxoglutarate 5-Dioxygenase 2 | Protein Coding | O00469 | 58 | GC03M146035 | 1.18561315536499 | https://www.genecards.org/cgi-bin/carddisp.pl?gene=PLOD2 |
| SRD5A2 | Steroid 5 Alpha-Reductase 2 | Protein Coding | P31213 | 55 | GC02M031522 | 1.18561315536499 | https://www.genecards.org/cgi-bin/carddisp.pl?gene=SRD5A2 |
| HPGDS | Hematopoietic Prostaglandin D Synthase | Protein Coding | O60760 | 54 | GC04M094298 | 1.18561315536499 | https://www.genecards.org/cgi-bin/carddisp.pl?gene=HPGDS |
| HK3 | Hexokinase 3 | Protein Coding | P52790 | 52 | GC05M183271 | 1.18561315536499 | https://www.genecards.org/cgi-bin/carddisp.pl?gene=HK3 |
| ZBTB17 | Zinc Finger And BTB Domain Containing 17 | Protein Coding | Q13105 | 52 | GC01M022596 | 1.18561315536499 | https://www.genecards.org/cgi-bin/carddisp.pl?gene=ZBTB17 |
| RASD1 | Ras Related Dexamethasone Induced 1 | Protein Coding | Q9Y272 | 49 | GC17M017494 | 1.18561315536499 | https://www.genecards.org/cgi-bin/carddisp.pl?gene=RASD1 |
| TAS1R3 | Taste 1 Receptor Member 3 | Protein Coding | Q7RTX0 | 48 | GC01P001331 | 1.18561315536499 | https://www.genecards.org/cgi-bin/carddisp.pl?gene=TAS1R3 |
| TAS1R2 | Taste 1 Receptor Member 2 | Protein Coding | Q8TE23 | 46 | GC01M018839 | 1.18561315536499 | https://www.genecards.org/cgi-bin/carddisp.pl?gene=TAS1R2 |
| MIR342 | MicroRNA 342 | RNA Gene |  | 30 | GC14P100109 | 1.18561315536499 | https://www.genecards.org/cgi-bin/carddisp.pl?gene=MIR342 |
| BCYRN1 | Brain Cytoplasmic RNA 1 | RNA Gene |  | 28 | GC02P047253 | 1.18561315536499 | https://www.genecards.org/cgi-bin/carddisp.pl?gene=BCYRN1 |
| MIR498 | MicroRNA 498 | RNA Gene |  | 24 | GC19P153069 | 1.18561315536499 | https://www.genecards.org/cgi-bin/carddisp.pl?gene=MIR498 |
| CPVL | Carboxypeptidase Vitellogenic Like | Protein Coding | Q9H3G5 | 48 | GC07M028995 | 1.17311096191406 | https://www.genecards.org/cgi-bin/carddisp.pl?gene=CPVL |
| FLNA | Filamin A | Protein Coding | P21333 | 62 | GC0XM154348 | 1.14445900917053 | https://www.genecards.org/cgi-bin/carddisp.pl?gene=FLNA |
| UBE2I | Ubiquitin Conjugating Enzyme E2 I | Protein Coding | P63279 | 60 | GC16P120125 | 1.14445900917053 | https://www.genecards.org/cgi-bin/carddisp.pl?gene=UBE2I |
| CFL1 | Cofilin 1 | Protein Coding | P23528 | 58 | GC11M065823 | 1.14445900917053 | https://www.genecards.org/cgi-bin/carddisp.pl?gene=CFL1 |
| FXR1 | FMR1 Autosomal Homolog 1 | Protein Coding | P51114 | 58 | GC03P180868 | 1.14445900917053 | https://www.genecards.org/cgi-bin/carddisp.pl?gene=FXR1 |
| ELN | Elastin | Protein Coding | P15502 | 56 | GC07P074027 | 1.14445900917053 | https://www.genecards.org/cgi-bin/carddisp.pl?gene=ELN |
| SRI | Sorcin | Protein Coding | P30626 | 54 | GC07M088205 | 1.14445900917053 | https://www.genecards.org/cgi-bin/carddisp.pl?gene=SRI |
| FXR2 | FMR1 Autosomal Homolog 2 | Protein Coding | P51116 | 51 | GC17M096212 | 1.14445900917053 | https://www.genecards.org/cgi-bin/carddisp.pl?gene=FXR2 |
| HMGCR | 3-Hydroxy-3-Methylglutaryl-CoA Reductase | Protein Coding | P04035 | 61 | GC05P075336 | 1.14041972160339 | https://www.genecards.org/cgi-bin/carddisp.pl?gene=HMGCR |
| SLC16A1 | Solute Carrier Family 16 Member 1 | Protein Coding | P53985 | 61 | GC01M113155 | 1.14041972160339 | https://www.genecards.org/cgi-bin/carddisp.pl?gene=SLC16A1 |
| BSG | Basigin (Ok Blood Group) | Protein Coding | P35613 | 57 | GC19P000571 | 1.14041972160339 | https://www.genecards.org/cgi-bin/carddisp.pl?gene=BSG |
| OGN | Osteoglycin | Protein Coding | P20774 | 52 | GC09M092383 | 1.14041972160339 | https://www.genecards.org/cgi-bin/carddisp.pl?gene=OGN |
| LRG1 | Leucine Rich Alpha-2-Glycoprotein 1 | Protein Coding | P02750 | 50 | GC19M107899 | 1.14041972160339 | https://www.genecards.org/cgi-bin/carddisp.pl?gene=LRG1 |
| TRU-TCA1-1 | TRNA-SeC (Anticodon TCA) 1-1 | RNA Gene |  | 18 | GC19M045478 | 1.14041972160339 | https://www.genecards.org/cgi-bin/carddisp.pl?gene=TRU-TCA1-1 |
| CENPO | Centromere Protein O | Protein Coding | Q9BU64 | 43 | GC02P024793 | 1.13311052322388 | https://www.genecards.org/cgi-bin/carddisp.pl?gene=CENPO |
| SPX | Spexin Hormone | Protein Coding | Q9BT56 | 41 | GC12P021526 | 1.10719203948975 | https://www.genecards.org/cgi-bin/carddisp.pl?gene=SPX |
| OPRM1 | Opioid Receptor Mu 1 | Protein Coding | P35372 | 61 | GC06P183705 | 1.08152258396149 | https://www.genecards.org/cgi-bin/carddisp.pl?gene=OPRM1 |
| HBA1 | Hemoglobin Subunit Alpha 1 | Protein Coding | P69905 | 56 | GC16P120095 | 1.08152258396149 | https://www.genecards.org/cgi-bin/carddisp.pl?gene=HBA1 |
| TENT5A | Terminal Nucleotidyltransferase 5A | Protein Coding | Q96IP4 | 46 | GC06M107887 | 1.08152258396149 | https://www.genecards.org/cgi-bin/carddisp.pl?gene=TENT5A |
| INSIG2 | Insulin Induced Gene 2 | Protein Coding | Q9Y5U4 | 47 | GC02P118088 | 1.06686568260193 | https://www.genecards.org/cgi-bin/carddisp.pl?gene=INSIG2 |
| CCN2 | Cellular Communication Network Factor 2 | Protein Coding | P29279 | 58 | GC06M131948 | 1.06610941886902 | https://www.genecards.org/cgi-bin/carddisp.pl?gene=CCN2 |
| PPARD | Peroxisome Proliferator Activated Receptor Delta | Protein Coding | Q03181 | 59 | GC06P182001 | 1.04416275024414 | https://www.genecards.org/cgi-bin/carddisp.pl?gene=PPARD |
| IMPDH1 | Inosine Monophosphate Dehydrogenase 1 | Protein Coding | P20839 | 61 | GC07M128392 | 1.03755462169647 | https://www.genecards.org/cgi-bin/carddisp.pl?gene=IMPDH1 |
| SND1 | Staphylococcal Nuclease And Tudor Domain Containing 1 | Protein Coding | Q7KZF4 | 52 | GC07P127652 | 1.03755462169647 | https://www.genecards.org/cgi-bin/carddisp.pl?gene=SND1 |
| LRRC4 | Leucine Rich Repeat Containing 4 | Protein Coding | Q9HBW1 | 50 | GC07M128027 | 1.03755462169647 | https://www.genecards.org/cgi-bin/carddisp.pl?gene=LRRC4 |
| GCC1 | GRIP And Coiled-Coil Domain Containing 1 | Protein Coding | Q96CN9 | 44 | GC07M127580 | 1.03755462169647 | https://www.genecards.org/cgi-bin/carddisp.pl?gene=GCC1 |
| METTL2B | Methyltransferase 2B, TRNA N3-Cytidine | Protein Coding | Q6P1Q9 | 44 | GC07P165493 | 1.03755462169647 | https://www.genecards.org/cgi-bin/carddisp.pl?gene=METTL2B |
| ZNF800 | Zinc Finger Protein 800 | Protein Coding | Q2TB10 | 38 | GC07M127346 | 1.03755462169647 | https://www.genecards.org/cgi-bin/carddisp.pl?gene=ZNF800 |
| ARHGAP40 | Rho GTPase Activating Protein 40 | Protein Coding | Q5TG30 | 34 | GC20P038601 | 1.03755462169647 | https://www.genecards.org/cgi-bin/carddisp.pl?gene=ARHGAP40 |
| LINC-PINT | Long Intergenic Non-Protein Coding RNA, P53 Induced Transcript | RNA Gene | A0A455ZAR2 | 27 | GC07M130795 | 1.03755462169647 | https://www.genecards.org/cgi-bin/carddisp.pl?gene=LINC-PINT |
| MIR129-1 | MicroRNA 129-1 | RNA Gene |  | 26 | GC07P128207 | 1.03755462169647 | https://www.genecards.org/cgi-bin/carddisp.pl?gene=MIR129-1 |
| LINC00880 | Long Intergenic Non-Protein Coding RNA 880 | RNA Gene |  | 18 | GC03M157081 | 1.03755462169647 | https://www.genecards.org/cgi-bin/carddisp.pl?gene=LINC00880 |
| LINC02029 | Long Intergenic Non-Protein Coding RNA 2029 | RNA Gene |  | 17 | GC03P157083 | 1.03755462169647 | https://www.genecards.org/cgi-bin/carddisp.pl?gene=LINC02029 |
| SNORA70F | Small Nucleolar RNA, H/ACA Box 70F | RNA Gene |  | 16 | GC02M164688 | 1.03755462169647 | https://www.genecards.org/cgi-bin/carddisp.pl?gene=SNORA70F |
| ENSG00000224138 | Novel Transcript | RNA Gene |  | 11 | GC07P127350 | 1.03755462169647 | https://www.genecards.org/cgi-bin/carddisp.pl?gene=ENSG00000224138 |
| ENSG00000242588 | Novel Transcript | RNA Gene |  | 10 | GC07P165494 | 1.03755462169647 | https://www.genecards.org/cgi-bin/carddisp.pl?gene=ENSG00000242588 |
| RPS10P15 | Ribosomal Protein S10 Pseudogene 15 | Pseudogene |  | 10 | GC07M128570 | 1.03755462169647 | https://www.genecards.org/cgi-bin/carddisp.pl?gene=RPS10P15 |
| ENSG00000229413 | Novel Pseudogene | Pseudogene |  | 7 | GC07M128653 | 1.03755462169647 | https://www.genecards.org/cgi-bin/carddisp.pl?gene=ENSG00000229413 |
| ENSG00000230715 | Novel Pseudogene | Pseudogene |  | 7 | GC07P165498 | 1.03755462169647 | https://www.genecards.org/cgi-bin/carddisp.pl?gene=ENSG00000230715 |
| ENSG00000243302 | Novel Pseudogene | Pseudogene |  | 7 | GC07P128651 | 1.03755462169647 | https://www.genecards.org/cgi-bin/carddisp.pl?gene=ENSG00000243302 |
| ENSG00000243679 | Novel Pseudogene | Pseudogene |  | 7 | GC07P165499 | 1.03755462169647 | https://www.genecards.org/cgi-bin/carddisp.pl?gene=ENSG00000243679 |
| ENSG00000292309 | Novel Transcript | RNA Gene |  | 7 | GC07P166285 | 1.03755462169647 | https://www.genecards.org/cgi-bin/carddisp.pl?gene=ENSG00000292309 |
| HSALNG0061179 |  | RNA Gene |  | 5 | GC07P165484 | 1.03755462169647 | https://www.genecards.org/cgi-bin/carddisp.pl?gene=HSALNG0061179 |
| HSALNG0150056 |  | RNA Gene |  | 4 | GC07M128212 | 1.03755462169647 | https://www.genecards.org/cgi-bin/carddisp.pl?gene=HSALNG0150056 |
| LOC124901744 | Uncharacterized LOC124901744 | RNA Gene |  | 4 | GC07P165483 | 1.03755462169647 | https://www.genecards.org/cgi-bin/carddisp.pl?gene=LOC124901744 |
| LOC105375508 | Uncharacterized LOC105375508 | RNA Gene |  | 3 | GC07P130734 | 1.03755462169647 | https://www.genecards.org/cgi-bin/carddisp.pl?gene=LOC105375508 |
| FGFR2 | Fibroblast Growth Factor Receptor 2 | Protein Coding | P21802 | 68 | GC10M121478 | 1.03092062473297 | https://www.genecards.org/cgi-bin/carddisp.pl?gene=FGFR2 |
| DNAJC6 | DnaJ Heat Shock Protein Family (Hsp40) Member C6 | Protein Coding | O75061 | 55 | GC01P065248 | 1.03092062473297 | https://www.genecards.org/cgi-bin/carddisp.pl?gene=DNAJC6 |
| RPGRIP1L | RPGRIP1 Like | Protein Coding | Q68CZ1 | 54 | GC16M053808 | 1.03092062473297 | https://www.genecards.org/cgi-bin/carddisp.pl?gene=RPGRIP1L |
| GC | GC Vitamin D Binding Protein | Protein Coding | P02774 | 58 | GC04M071741 | 1.02754187583923 | https://www.genecards.org/cgi-bin/carddisp.pl?gene=GC |
| TLR4 | Toll Like Receptor 4 | Protein Coding | O00206 | 64 | GC09P117704 | 1.0210884809494 | https://www.genecards.org/cgi-bin/carddisp.pl?gene=TLR4 |
| MTHFR | Methylenetetrahydrofolate Reductase | Protein Coding | P42898 | 61 | GC01M011785 | 1.0210884809494 | https://www.genecards.org/cgi-bin/carddisp.pl?gene=MTHFR |
| MSTN | Myostatin | Protein Coding | O14793 | 59 | GC02M190055 | 1.00742900371552 | https://www.genecards.org/cgi-bin/carddisp.pl?gene=MSTN |
| DNMT1 | DNA Methyltransferase 1 | Protein Coding | P26358 | 66 | GC19M010133 | 1.00226867198944 | https://www.genecards.org/cgi-bin/carddisp.pl?gene=DNMT1 |
| GRPR | Gastrin Releasing Peptide Receptor | Protein Coding | P30550 | 57 | GC0XP016124 | 1.00226867198944 | https://www.genecards.org/cgi-bin/carddisp.pl?gene=GRPR |
| NCOR1 | Nuclear Receptor Corepressor 1 | Protein Coding | O75376 | 57 | GC17M016029 | 1.00226867198944 | https://www.genecards.org/cgi-bin/carddisp.pl?gene=NCOR1 |
| HDAC2 | Histone Deacetylase 2 | Protein Coding | Q92769 | 65 | GC06M113933 | 0.99703460931778 | https://www.genecards.org/cgi-bin/carddisp.pl?gene=HDAC2 |
| HDAC1 | Histone Deacetylase 1 | Protein Coding | Q13547 | 63 | GC01P032292 | 0.99703460931778 | https://www.genecards.org/cgi-bin/carddisp.pl?gene=HDAC1 |
| GZMB | Granzyme B | Protein Coding | P10144 | 60 | GC14M024630 | 0.99703460931778 | https://www.genecards.org/cgi-bin/carddisp.pl?gene=GZMB |
| WRN | WRN RecQ Like Helicase | Protein Coding | Q14191 | 60 | GC08P031033 | 0.99703460931778 | https://www.genecards.org/cgi-bin/carddisp.pl?gene=WRN |
| LMNB1 | Lamin B1 | Protein Coding | P20700 | 59 | GC05P126776 | 0.99703460931778 | https://www.genecards.org/cgi-bin/carddisp.pl?gene=LMNB1 |
| PCYT1A | Phosphate Cytidylyltransferase 1A, Choline | Protein Coding | P49585 | 59 | GC03M196214 | 0.99703460931778 | https://www.genecards.org/cgi-bin/carddisp.pl?gene=PCYT1A |
| EMD | Emerin | Protein Coding | P50402 | 58 | GC0XP154379 | 0.99703460931778 | https://www.genecards.org/cgi-bin/carddisp.pl?gene=EMD |
| DGAT2 | Diacylglycerol O-Acyltransferase 2 | Protein Coding | Q96PD7 | 55 | GC11P075759 | 0.99703460931778 | https://www.genecards.org/cgi-bin/carddisp.pl?gene=DGAT2 |
| SUN1 | Sad1 And UNC84 Domain Containing 1 | Protein Coding | O94901 | 52 | GC07P000815 | 0.99703460931778 | https://www.genecards.org/cgi-bin/carddisp.pl?gene=SUN1 |
| H3-3B | H3.3 Histone B | Protein Coding | P84243 | 51 | GC17M098063 | 0.99703460931778 | https://www.genecards.org/cgi-bin/carddisp.pl?gene=H3-3B |
| GPAT3 | Glycerol-3-Phosphate Acyltransferase 3 | Protein Coding | Q53EU6 | 50 | GC04P083771 | 0.99703460931778 | https://www.genecards.org/cgi-bin/carddisp.pl?gene=GPAT3 |
| GPAT4 | Glycerol-3-Phosphate Acyltransferase 4 | Protein Coding | Q86UL3 | 48 | GC08P041577 | 0.99703460931778 | https://www.genecards.org/cgi-bin/carddisp.pl?gene=GPAT4 |
| FITM2 | Fat Storage Inducing Transmembrane Protein 2 | Protein Coding | Q8N6M3 | 47 | GC20M044302 | 0.99703460931778 | https://www.genecards.org/cgi-bin/carddisp.pl?gene=FITM2 |
| H3C12 | H3 Clustered Histone 12 | Protein Coding | P68431 | 47 | GC06M109200 | 0.99703460931778 | https://www.genecards.org/cgi-bin/carddisp.pl?gene=H3C12 |
| H3C13 | H3 Clustered Histone 13 | Protein Coding | Q71DI3 | 45 | GC01M167216 | 0.99703460931778 | https://www.genecards.org/cgi-bin/carddisp.pl?gene=H3C13 |
| MIR9-1 | MicroRNA 9-1 | RNA Gene |  | 30 | GC01M156420 | 0.99703460931778 | https://www.genecards.org/cgi-bin/carddisp.pl?gene=MIR9-1 |
| MT3 | Metallothionein 3 | Protein Coding | P25713 | 53 | GC16P056589 | 0.990485429763794 | https://www.genecards.org/cgi-bin/carddisp.pl?gene=MT3 |
| FOXP2 | Forkhead Box P2 | Protein Coding | O15409 | 56 | GC07P114086 | 0.989390671253204 | https://www.genecards.org/cgi-bin/carddisp.pl?gene=FOXP2 |
| PAPPA2 | Pappalysin 2 | Protein Coding | Q9BXP8 | 52 | GC01P176463 | 0.964512586593628 | https://www.genecards.org/cgi-bin/carddisp.pl?gene=PAPPA2 |
| MIR1290 | MicroRNA 1290 | RNA Gene |  | 20 | GC01M018897 | 0.960020780563354 | https://www.genecards.org/cgi-bin/carddisp.pl?gene=MIR1290 |
| SLC6A4 | Solute Carrier Family 6 Member 4 | Protein Coding | P31645 | 61 | GC17M030194 | 0.949071407318115 | https://www.genecards.org/cgi-bin/carddisp.pl?gene=SLC6A4 |
| TSHR | Thyroid Stimulating Hormone Receptor | Protein Coding | P16473 | 61 | GC14P080954 | 0.941438257694244 | https://www.genecards.org/cgi-bin/carddisp.pl?gene=TSHR |
| NR0B1 | Nuclear Receptor Subfamily 0 Group B Member 1 | Protein Coding | P51843 | 59 | GC0XM030304 | 0.941438257694244 | https://www.genecards.org/cgi-bin/carddisp.pl?gene=NR0B1 |
| C1QTNF9 | C1q And TNF Related 9 | Protein Coding | P0C862 | 44 | GC13P024307 | 0.941438257694244 | https://www.genecards.org/cgi-bin/carddisp.pl?gene=C1QTNF9 |
| ARL15 | ARF Like GTPase 15 | Protein Coding | Q9NXU5 | 45 | GC05M053883 | 0.934749484062195 | https://www.genecards.org/cgi-bin/carddisp.pl?gene=ARL15 |
| TH | Tyrosine Hydroxylase | Protein Coding | P07101 | 63 | GC11M002163 | 0.90609747171402 | https://www.genecards.org/cgi-bin/carddisp.pl?gene=TH |
| MIR132 | MicroRNA 132 | RNA Gene |  | 30 | GC17M002049 | 0.90609747171402 | https://www.genecards.org/cgi-bin/carddisp.pl?gene=MIR132 |
| HNF1A | HNF1 Homeobox A | Protein Coding | P20823 | 60 | GC12P120978 | 0.891979336738586 | https://www.genecards.org/cgi-bin/carddisp.pl?gene=HNF1A |
| CNR2 | Cannabinoid Receptor 2 | Protein Coding | P34972 | 57 | GC01M023870 | 0.891979336738586 | https://www.genecards.org/cgi-bin/carddisp.pl?gene=CNR2 |
| PIBF1 | Progesterone Immunomodulatory Binding Factor 1 | Protein Coding | Q8WXW3 | 55 | GC13P072782 | 0.891979336738586 | https://www.genecards.org/cgi-bin/carddisp.pl?gene=PIBF1 |
| FBN1 | Fibrillin 1 | Protein Coding | P35555 | 60 | GC15M048408 | 0.891067504882813 | https://www.genecards.org/cgi-bin/carddisp.pl?gene=FBN1 |
| SLC27A1 | Solute Carrier Family 27 Member 1 | Protein Coding | Q6PCB7 | 51 | GC19P152087 | 0.891067504882813 | https://www.genecards.org/cgi-bin/carddisp.pl?gene=SLC27A1 |
| MIR4505 | MicroRNA 4505 | RNA Gene |  | 17 | GC14P073758 | 0.888354420661926 | https://www.genecards.org/cgi-bin/carddisp.pl?gene=MIR4505 |
| RNY5 | RNA, Ro60-Associated Y5 | RNA Gene |  | 11 | GC07P148941 | 0.888354420661926 | https://www.genecards.org/cgi-bin/carddisp.pl?gene=RNY5 |
| CEBPB | CCAAT Enhancer Binding Protein Beta | Protein Coding | P17676 | 58 | GC20P050190 | 0.878898143768311 | https://www.genecards.org/cgi-bin/carddisp.pl?gene=CEBPB |
| LOC110806262 | Solute Carrier Family 6 Member 4 Gene Promoter | Functional Element |  | 9 | GC17P030235 | 0.878898143768311 | https://www.genecards.org/cgi-bin/carddisp.pl?gene=LOC110806262 |
| KCNJ11 | Potassium Inwardly Rectifying Channel Subfamily J Member 11 | Protein Coding | Q14654 | 59 | GC11M018109 | 0.875852346420288 | https://www.genecards.org/cgi-bin/carddisp.pl?gene=KCNJ11 |
| HLA-DQB1 | Major Histocompatibility Complex, Class II, DQ Beta 1 | Protein Coding | P01920 | 57 | GC06M106540 | 0.875852346420288 | https://www.genecards.org/cgi-bin/carddisp.pl?gene=HLA-DQB1 |
| NPBWR1 | Neuropeptides B And W Receptor 1 | Protein Coding | P48145 | 44 | GC08P052938 | 0.875852346420288 | https://www.genecards.org/cgi-bin/carddisp.pl?gene=NPBWR1 |
| ACLY | ATP Citrate Lyase | Protein Coding | P53396 | 59 | GC17M041866 | 0.870032668113708 | https://www.genecards.org/cgi-bin/carddisp.pl?gene=ACLY |
| AZGP1 | Alpha-2-Glycoprotein 1, Zinc-Binding | Protein Coding | P25311 | 55 | GC07M106710 | 0.870032668113708 | https://www.genecards.org/cgi-bin/carddisp.pl?gene=AZGP1 |
| SOST | Sclerostin | Protein Coding | Q9BQB4 | 55 | GC17M043753 | 0.870032668113708 | https://www.genecards.org/cgi-bin/carddisp.pl?gene=SOST |
| RELN | Reelin | Protein Coding | P78509 | 57 | GC07M103471 | 0.863457262516022 | https://www.genecards.org/cgi-bin/carddisp.pl?gene=RELN |
| DIO2 | Iodothyronine Deiodinase 2 | Protein Coding | Q92813 | 52 | GC14M080197 | 0.863161027431488 | https://www.genecards.org/cgi-bin/carddisp.pl?gene=DIO2 |
| LIPA | Lipase A, Lysosomal Acid Type | Protein Coding | P38571 | 62 | GC10M089213 | 0.848729848861694 | https://www.genecards.org/cgi-bin/carddisp.pl?gene=LIPA |
| MEF2C | Myocyte Enhancer Factor 2C | Protein Coding | Q06413 | 62 | GC05M088718 | 0.848729848861694 | https://www.genecards.org/cgi-bin/carddisp.pl?gene=MEF2C |
| SCN8A | Sodium Voltage-Gated Channel Alpha Subunit 8 | Protein Coding | Q9UQD0 | 62 | GC12P051590 | 0.848729848861694 | https://www.genecards.org/cgi-bin/carddisp.pl?gene=SCN8A |
| GABBR2 | Gamma-Aminobutyric Acid Type B Receptor Subunit 2 | Protein Coding | O75899 | 61 | GC09M098288 | 0.848729848861694 | https://www.genecards.org/cgi-bin/carddisp.pl?gene=GABBR2 |
| SMC1A | Structural Maintenance Of Chromosomes 1A | Protein Coding | Q14683 | 61 | GC0XM053374 | 0.848729848861694 | https://www.genecards.org/cgi-bin/carddisp.pl?gene=SMC1A |
| STXBP1 | Syntaxin Binding Protein 1 | Protein Coding | P61764 | 61 | GC09P152828 | 0.848729848861694 | https://www.genecards.org/cgi-bin/carddisp.pl?gene=STXBP1 |
| ADSL | Adenylosuccinate Lyase | Protein Coding | P30566 | 60 | GC22P040346 | 0.848729848861694 | https://www.genecards.org/cgi-bin/carddisp.pl?gene=ADSL |
| ALDH5A1 | Aldehyde Dehydrogenase 5 Family Member A1 | Protein Coding | P51649 | 60 | GC06P024494 | 0.848729848861694 | https://www.genecards.org/cgi-bin/carddisp.pl?gene=ALDH5A1 |
| ERCC4 | ERCC Excision Repair 4, Endonuclease Catalytic Subunit | Protein Coding | Q92889 | 59 | GC16P013920 | 0.848729848861694 | https://www.genecards.org/cgi-bin/carddisp.pl?gene=ERCC4 |
| ABCG5 | ATP Binding Cassette Subfamily G Member 5 | Protein Coding | Q9H222 | 58 | GC02M043806 | 0.848729848861694 | https://www.genecards.org/cgi-bin/carddisp.pl?gene=ABCG5 |
| FOXG1 | Forkhead Box G1 | Protein Coding | P55316 | 57 | GC14P057625 | 0.848729848861694 | https://www.genecards.org/cgi-bin/carddisp.pl?gene=FOXG1 |
| PSMA3 | Proteasome 20S Subunit Alpha 3 | Protein Coding | P25788 | 57 | GC14P058244 | 0.848729848861694 | https://www.genecards.org/cgi-bin/carddisp.pl?gene=PSMA3 |
| CDKL5 | Cyclin Dependent Kinase Like 5 | Protein Coding | O76039 | 56 | GC0XP018425 | 0.848729848861694 | https://www.genecards.org/cgi-bin/carddisp.pl?gene=CDKL5 |
| NPC1L1 | NPC1 Like Intracellular Cholesterol Transporter 1 | Protein Coding | Q9UHC9 | 56 | GC07M044512 | 0.848729848861694 | https://www.genecards.org/cgi-bin/carddisp.pl?gene=NPC1L1 |
| CELA2A | Chymotrypsin Like Elastase 2A | Protein Coding | P08217 | 55 | GC01P015456 | 0.848729848861694 | https://www.genecards.org/cgi-bin/carddisp.pl?gene=CELA2A |
| ABCG8 | ATP Binding Cassette Subfamily G Member 8 | Protein Coding | Q9H221 | 54 | GC02P045317 | 0.848729848861694 | https://www.genecards.org/cgi-bin/carddisp.pl?gene=ABCG8 |
| NTNG1 | Netrin G1 | Protein Coding | Q9Y2I2 | 54 | GC01P107140 | 0.848729848861694 | https://www.genecards.org/cgi-bin/carddisp.pl?gene=NTNG1 |
| SAR1B | Secretion Associated Ras Related GTPase 1B | Protein Coding | Q9Y6B6 | 54 | GC05M134601 | 0.848729848861694 | https://www.genecards.org/cgi-bin/carddisp.pl?gene=SAR1B |
| IQSEC2 | IQ Motif And Sec7 Domain ArfGEF 2 | Protein Coding | Q5JU85 | 52 | GC0XM053225 | 0.848729848861694 | https://www.genecards.org/cgi-bin/carddisp.pl?gene=IQSEC2 |
| LDLRAP1 | Low Density Lipoprotein Receptor Adaptor Protein 1 | Protein Coding | Q5SW96 | 52 | GC01P025543 | 0.848729848861694 | https://www.genecards.org/cgi-bin/carddisp.pl?gene=LDLRAP1 |
| DOK7 | Docking Protein 7 | Protein Coding | Q18PE1 | 51 | GC04P029596 | 0.848729848861694 | https://www.genecards.org/cgi-bin/carddisp.pl?gene=DOK7 |
| RHOBTB2 | Rho Related BTB Domain Containing 2 | Protein Coding | Q9BYZ6 | 51 | GC08P026101 | 0.848729848861694 | https://www.genecards.org/cgi-bin/carddisp.pl?gene=RHOBTB2 |
| CREB3L3 | CAMP Responsive Element Binding Protein 3 Like 3 | Protein Coding | Q68CJ9 | 50 | GC19P004153 | 0.848729848861694 | https://www.genecards.org/cgi-bin/carddisp.pl?gene=CREB3L3 |
| LMF1 | Lipase Maturation Factor 1 | Protein Coding | Q96S06 | 50 | GC16M000853 | 0.848729848861694 | https://www.genecards.org/cgi-bin/carddisp.pl?gene=LMF1 |
| STAP1 | Signal Transducing Adaptor Family Member 1 | Protein Coding | Q9ULZ2 | 50 | GC04P067558 | 0.848729848861694 | https://www.genecards.org/cgi-bin/carddisp.pl?gene=STAP1 |
| GPIHBP1 | Glycosylphosphatidylinositol Anchored High Density Lipoprotein Binding Protein 1 | Protein Coding | Q8IV16 | 48 | GC08P143213 | 0.848729848861694 | https://www.genecards.org/cgi-bin/carddisp.pl?gene=GPIHBP1 |
| EIF1AD | Eukaryotic Translation Initiation Factor 1A Domain Containing | Protein Coding | Q8N9N8 | 41 | GC11M065996 | 0.848729848861694 | https://www.genecards.org/cgi-bin/carddisp.pl?gene=EIF1AD |
| LIPE-AS1 | LIPE Antisense RNA 1 | RNA Gene |  | 19 | GC19P152613 | 0.848729848861694 | https://www.genecards.org/cgi-bin/carddisp.pl?gene=LIPE-AS1 |
| LOC101930071 | Uncharacterized LOC101930071 | RNA Gene |  | 12 | GC19P152614 | 0.848729848861694 | https://www.genecards.org/cgi-bin/carddisp.pl?gene=LOC101930071 |
| LOC130068854 | ATAC-STARR-Seq Lymphoblastoid Silent Region 21085 | Functional Element |  | 9 | GC0XP155161 | 0.848729848861694 | https://www.genecards.org/cgi-bin/carddisp.pl?gene=LOC130068854 |
| TSC2 | TSC Complex Subunit 2 | Protein Coding | P49815 | 64 | GC16P120169 | 0.847200274467468 | https://www.genecards.org/cgi-bin/carddisp.pl?gene=TSC2 |
| HDAC9 | Histone Deacetylase 9 | Protein Coding | Q9UKV0 | 63 | GC07P018086 | 0.847200274467468 | https://www.genecards.org/cgi-bin/carddisp.pl?gene=HDAC9 |
| CFB | Complement Factor B | Protein Coding | P00751 | 60 | GC06P031945 | 0.847200274467468 | https://www.genecards.org/cgi-bin/carddisp.pl?gene=CFB |
| E2F1 | E2F Transcription Factor 1 | Protein Coding | Q01094 | 57 | GC20M033675 | 0.847200274467468 | https://www.genecards.org/cgi-bin/carddisp.pl?gene=E2F1 |
| LPAR3 | Lysophosphatidic Acid Receptor 3 | Protein Coding | Q9UBY5 | 56 | GC01M084811 | 0.847200274467468 | https://www.genecards.org/cgi-bin/carddisp.pl?gene=LPAR3 |
| SRY | Sex Determining Region Y | Protein Coding | Q05066 | 51 | GC0YM002698 | 0.847200274467468 | https://www.genecards.org/cgi-bin/carddisp.pl?gene=SRY |
| TET1 | Tet Methylcytosine Dioxygenase 1 | Protein Coding | Q8NFU7 | 51 | GC10P068560 | 0.847200274467468 | https://www.genecards.org/cgi-bin/carddisp.pl?gene=TET1 |
| DKK1 | Dickkopf WNT Signaling Pathway Inhibitor 1 | Protein Coding | O94907 | 57 | GC10P052314 | 0.84695839881897 | https://www.genecards.org/cgi-bin/carddisp.pl?gene=DKK1 |
| SORBS1 | Sorbin And SH3 Domain Containing 1 | Protein Coding | Q9BX66 | 53 | GC10M095311 | 0.84695839881897 | https://www.genecards.org/cgi-bin/carddisp.pl?gene=SORBS1 |
| LTA | Lymphotoxin Alpha | Protein Coding | P01374 | 55 | GC06P181891 | 0.840798318386078 | https://www.genecards.org/cgi-bin/carddisp.pl?gene=LTA |
| ADD1 | Adducin 1 | Protein Coding | P35611 | 58 | GC04P029564 | 0.832814455032349 | https://www.genecards.org/cgi-bin/carddisp.pl?gene=ADD1 |
| LGALS3 | Galectin 3 | Protein Coding | P17931 | 57 | GC14P055124 | 0.832814455032349 | https://www.genecards.org/cgi-bin/carddisp.pl?gene=LGALS3 |
| PROP1 | PROP Paired-Like Homeobox 1 | Protein Coding | O75360 | 51 | GC05M177992 | 0.832814455032349 | https://www.genecards.org/cgi-bin/carddisp.pl?gene=PROP1 |
| FGF2 | Fibroblast Growth Factor 2 | Protein Coding | P09038 | 59 | GC04P122826 | 0.822562336921692 | https://www.genecards.org/cgi-bin/carddisp.pl?gene=FGF2 |
| PDYN | Prodynorphin | Protein Coding | P01213 | 57 | GC20M001978 | 0.822562336921692 | https://www.genecards.org/cgi-bin/carddisp.pl?gene=PDYN |
| FABP5 | Fatty Acid Binding Protein 5 | Protein Coding | Q01469 | 55 | GC08P081282 | 0.822562336921692 | https://www.genecards.org/cgi-bin/carddisp.pl?gene=FABP5 |
| LIPF | Lipase F, Gastric Type | Protein Coding | P07098 | 54 | GC10P088664 | 0.822562336921692 | https://www.genecards.org/cgi-bin/carddisp.pl?gene=LIPF |
| UCN2 | Urocortin 2 | Protein Coding | Q96RP3 | 40 | GC03M048561 | 0.822562336921692 | https://www.genecards.org/cgi-bin/carddisp.pl?gene=UCN2 |
| PON2 | Paraoxonase 2 | Protein Coding | Q15165 | 57 | GC07M095404 | 0.799247920513153 | https://www.genecards.org/cgi-bin/carddisp.pl?gene=PON2 |
| LHX3 | LIM Homeobox 3 | Protein Coding | Q9UBR4 | 55 | GC09M136196 | 0.799247920513153 | https://www.genecards.org/cgi-bin/carddisp.pl?gene=LHX3 |
| C1QTNF1 | C1q And TNF Related 1 | Protein Coding | Q9BXJ1 | 50 | GC17P079022 | 0.799247920513153 | https://www.genecards.org/cgi-bin/carddisp.pl?gene=C1QTNF1 |
| HESX1 | HESX Homeobox 1 | Protein Coding | Q9UBX0 | 49 | GC03M057207 | 0.799247920513153 | https://www.genecards.org/cgi-bin/carddisp.pl?gene=HESX1 |
| PTH1R | Parathyroid Hormone 1 Receptor | Protein Coding | Q03431 | 62 | GC03P046877 | 0.796587586402893 | https://www.genecards.org/cgi-bin/carddisp.pl?gene=PTH1R |
| GDF2 | Growth Differentiation Factor 2 | Protein Coding | Q9UK05 | 57 | GC10P047322 | 0.796587586402893 | https://www.genecards.org/cgi-bin/carddisp.pl?gene=GDF2 |
| CHGA | Chromogranin A | Protein Coding | P10645 | 56 | GC14P095285 | 0.796587586402893 | https://www.genecards.org/cgi-bin/carddisp.pl?gene=CHGA |
| MIR125A | MicroRNA 125a | RNA Gene |  | 30 | GC19P152978 | 0.796587586402893 | https://www.genecards.org/cgi-bin/carddisp.pl?gene=MIR125A |
| FGB | Fibrinogen Beta Chain | Protein Coding | P02675 | 60 | GC04P154727 | 0.789397895336151 | https://www.genecards.org/cgi-bin/carddisp.pl?gene=FGB |
| NR1D1 | Nuclear Receptor Subfamily 1 Group D Member 1 | Protein Coding | P20393 | 60 | GC17M040092 | 0.789397895336151 | https://www.genecards.org/cgi-bin/carddisp.pl?gene=NR1D1 |
| CCNA2 | Cyclin A2 | Protein Coding | P20248 | 58 | GC04M121816 | 0.789397895336151 | https://www.genecards.org/cgi-bin/carddisp.pl?gene=CCNA2 |
| NR4A3 | Nuclear Receptor Subfamily 4 Group A Member 3 | Protein Coding | Q92570 | 55 | GC09P099821 | 0.789397895336151 | https://www.genecards.org/cgi-bin/carddisp.pl?gene=NR4A3 |
| PID1 | Phosphotyrosine Interaction Domain Containing 1 | Protein Coding | Q7Z2X4 | 39 | GC02M228850 | 0.789397895336151 | https://www.genecards.org/cgi-bin/carddisp.pl?gene=PID1 |
| IKBKB | Inhibitor Of Nuclear Factor Kappa B Kinase Subunit Beta | Protein Coding | O14920 | 67 | GC08P042271 | 0.768681168556213 | https://www.genecards.org/cgi-bin/carddisp.pl?gene=IKBKB |
| ALOX15 | Arachidonate 15-Lipoxygenase | Protein Coding | P16050 | 58 | GC17M004630 | 0.768681168556213 | https://www.genecards.org/cgi-bin/carddisp.pl?gene=ALOX15 |
| CSF1 | Colony Stimulating Factor 1 | Protein Coding | P09603 | 57 | GC01P111947 | 0.768681168556213 | https://www.genecards.org/cgi-bin/carddisp.pl?gene=CSF1 |
| CAPNS1 | Calpain Small Subunit 1 | Protein Coding | P04632 | 55 | GC19P152412 | 0.768681168556213 | https://www.genecards.org/cgi-bin/carddisp.pl?gene=CAPNS1 |
| MCAM | Melanoma Cell Adhesion Molecule | Protein Coding | P43121 | 54 | GC11M119308 | 0.768681168556213 | https://www.genecards.org/cgi-bin/carddisp.pl?gene=MCAM |
| RNU1-1 | RNA, U1 Small Nuclear 1 | RNA Gene |  | 22 | GC01M016514 | 0.768681168556213 | https://www.genecards.org/cgi-bin/carddisp.pl?gene=RNU1-1 |
| MTR | 5-Methyltetrahydrofolate-Homocysteine Methyltransferase | Protein Coding | Q99707 | 61 | GC01P236795 | 0.76114809513092 | https://www.genecards.org/cgi-bin/carddisp.pl?gene=MTR |
| MTRR | 5-Methyltetrahydrofolate-Homocysteine Methyltransferase Reductase | Protein Coding | Q9UBK8 | 57 | GC05P007851 | 0.76114809513092 | https://www.genecards.org/cgi-bin/carddisp.pl?gene=MTRR |
| PER2 | Period Circadian Regulator 2 | Protein Coding | O15055 | 56 | GC02M238244 | 0.76114809513092 | https://www.genecards.org/cgi-bin/carddisp.pl?gene=PER2 |
| CEBPD | CCAAT Enhancer Binding Protein Delta | Protein Coding | P49716 | 52 | GC08M047765 | 0.76114809513092 | https://www.genecards.org/cgi-bin/carddisp.pl?gene=CEBPD |
| GGA1 | Golgi Associated, Gamma Adaptin Ear Containing, ARF Binding Protein 1 | Protein Coding | Q9UJY5 | 49 | GC22P037608 | 0.76114809513092 | https://www.genecards.org/cgi-bin/carddisp.pl?gene=GGA1 |
| ANGPTL8 | Angiopoietin Like 8 | Protein Coding | Q6UXH0 | 45 | GC19P151823 | 0.76114809513092 | https://www.genecards.org/cgi-bin/carddisp.pl?gene=ANGPTL8 |
| CALCA | Calcitonin Related Polypeptide Alpha | Protein Coding | P06881 | 57 | GC11M014966 | 0.746904134750366 | https://www.genecards.org/cgi-bin/carddisp.pl?gene=CALCA |
| ANPEP | Alanyl Aminopeptidase, Membrane | Protein Coding | P15144 | 62 | GC15M089784 | 0.738334596157074 | https://www.genecards.org/cgi-bin/carddisp.pl?gene=ANPEP |
| ENO2 | Enolase 2 | Protein Coding | P09104 | 60 | GC12P006913 | 0.738334596157074 | https://www.genecards.org/cgi-bin/carddisp.pl?gene=ENO2 |
| CCL4 | C-C Motif Chemokine Ligand 4 | Protein Coding | P13236 | 52 | GC17P036103 | 0.738334596157074 | https://www.genecards.org/cgi-bin/carddisp.pl?gene=CCL4 |
| GH2 | Growth Hormone 2 | Protein Coding | P01242 | 50 | GC17M063880 | 0.738334596157074 | https://www.genecards.org/cgi-bin/carddisp.pl?gene=GH2 |
| TRA-TGC7-1 | TRNA-Ala (Anticodon TGC) 7-1 | RNA Gene |  | 14 | GC06M106311 | 0.738334596157074 | https://www.genecards.org/cgi-bin/carddisp.pl?gene=TRA-TGC7-1 |
| TAP1 | Transporter 1, ATP Binding Cassette Subfamily B Member | Protein Coding | Q03518 | 61 | GC06M106547 | 0.733661949634552 | https://www.genecards.org/cgi-bin/carddisp.pl?gene=TAP1 |
| HLA-DRB1 | Major Histocompatibility Complex, Class II, DR Beta 1 | Protein Coding | P01911 | 60 | GC06M106528 | 0.733661949634552 | https://www.genecards.org/cgi-bin/carddisp.pl?gene=HLA-DRB1 |
| PSMB9 | Proteasome 20S Subunit Beta 9 | Protein Coding | P28065 | 59 | GC06P181924 | 0.733661949634552 | https://www.genecards.org/cgi-bin/carddisp.pl?gene=PSMB9 |
| DRD1 | Dopamine Receptor D1 | Protein Coding | P21728 | 58 | GC05M175440 | 0.733661949634552 | https://www.genecards.org/cgi-bin/carddisp.pl?gene=DRD1 |
| HABP2 | Hyaluronan Binding Protein 2 | Protein Coding | Q14520 | 58 | GC10P113550 | 0.733661949634552 | https://www.genecards.org/cgi-bin/carddisp.pl?gene=HABP2 |
| HLA-DRA | Major Histocompatibility Complex, Class II, DR Alpha | Protein Coding | P01903 | 58 | GC06P032439 | 0.733661949634552 | https://www.genecards.org/cgi-bin/carddisp.pl?gene=HLA-DRA |
| TAP2 | Transporter 2, ATP Binding Cassette Subfamily B Member | Protein Coding | Q03519 | 58 | GC06M032821 | 0.733661949634552 | https://www.genecards.org/cgi-bin/carddisp.pl?gene=TAP2 |
| TP63 | Tumor Protein P63 | Protein Coding | Q9H3D4 | 58 | GC03P189598 | 0.733661949634552 | https://www.genecards.org/cgi-bin/carddisp.pl?gene=TP63 |
| BRD2 | Bromodomain Containing 2 | Protein Coding | P25440 | 57 | GC06P181927 | 0.733661949634552 | https://www.genecards.org/cgi-bin/carddisp.pl?gene=BRD2 |
| C4A | Complement C4A (Chido/Rodgers Blood Group) | Protein Coding | P0C0L4 | 57 | GC06P181914 | 0.733661949634552 | https://www.genecards.org/cgi-bin/carddisp.pl?gene=C4A |
| DOCK2 | Dedicator Of Cytokinesis 2 | Protein Coding | Q92608 | 57 | GC05P169637 | 0.733661949634552 | https://www.genecards.org/cgi-bin/carddisp.pl?gene=DOCK2 |
| HLA-DPB1 | Major Histocompatibility Complex, Class II, DP Beta 1 | Protein Coding | P04440 | 57 | GC06P181928 | 0.733661949634552 | https://www.genecards.org/cgi-bin/carddisp.pl?gene=HLA-DPB1 |
| MCCC1 | Methylcrotonyl-CoA Carboxylase Subunit 1 | Protein Coding | Q96RQ3 | 57 | GC03M183015 | 0.733661949634552 | https://www.genecards.org/cgi-bin/carddisp.pl?gene=MCCC1 |
| PDE4B | Phosphodiesterase 4B | Protein Coding | Q07343 | 57 | GC01P065792 | 0.733661949634552 | https://www.genecards.org/cgi-bin/carddisp.pl?gene=PDE4B |
| TFAM | Transcription Factor A, Mitochondrial | Protein Coding | Q00059 | 57 | GC10P058385 | 0.733661949634552 | https://www.genecards.org/cgi-bin/carddisp.pl?gene=TFAM |
| NRXN3 | Neurexin 3 | Protein Coding | Q9Y4C0 | 56 | GC14P078170 | 0.733661949634552 | https://www.genecards.org/cgi-bin/carddisp.pl?gene=NRXN3 |
| RB1CC1 | RB1 Inducible Coiled-Coil 1 | Protein Coding | Q8TDY2 | 56 | GC08M052622 | 0.733661949634552 | https://www.genecards.org/cgi-bin/carddisp.pl?gene=RB1CC1 |
| GNA13 | G Protein Subunit Alpha 13 | Protein Coding | Q14344 | 55 | GC17M065009 | 0.733661949634552 | https://www.genecards.org/cgi-bin/carddisp.pl?gene=GNA13 |
| HLA-DQA1 | Major Histocompatibility Complex, Class II, DQ Alpha 1 | Protein Coding | P01909 | 55 | GC06P181921 | 0.733661949634552 | https://www.genecards.org/cgi-bin/carddisp.pl?gene=HLA-DQA1 |
| POSTN | Periostin | Protein Coding | Q15063 | 55 | GC13M037562 | 0.733661949634552 | https://www.genecards.org/cgi-bin/carddisp.pl?gene=POSTN |
| SLIT3 | Slit Guidance Ligand 3 | Protein Coding | O75094 | 55 | GC05M168661 | 0.733661949634552 | https://www.genecards.org/cgi-bin/carddisp.pl?gene=SLIT3 |
| MTHFS | Methenyltetrahydrofolate Synthetase | Protein Coding | P49914 | 54 | GC15M079833 | 0.733661949634552 | https://www.genecards.org/cgi-bin/carddisp.pl?gene=MTHFS |
| SPTB | Spectrin Beta, Erythrocytic | Protein Coding | P11277 | 54 | GC14M064746 | 0.733661949634552 | https://www.genecards.org/cgi-bin/carddisp.pl?gene=SPTB |
| CHN2 | Chimerin 2 | Protein Coding | P52757 | 53 | GC07P030201 | 0.733661949634552 | https://www.genecards.org/cgi-bin/carddisp.pl?gene=CHN2 |
| LRP1B | LDL Receptor Related Protein 1B | Protein Coding | Q9NZR2 | 53 | GC02M140231 | 0.733661949634552 | https://www.genecards.org/cgi-bin/carddisp.pl?gene=LRP1B |
| OPCML | Opioid Binding Protein/Cell Adhesion Molecule Like | Protein Coding | Q14982 | 53 | GC11M132414 | 0.733661949634552 | https://www.genecards.org/cgi-bin/carddisp.pl?gene=OPCML |
| CAMK1D | Calcium/Calmodulin Dependent Protein Kinase ID | Protein Coding | Q8IU85 | 52 | GC10P012349 | 0.733661949634552 | https://www.genecards.org/cgi-bin/carddisp.pl?gene=CAMK1D |
| CD93 | CD93 Molecule | Protein Coding | Q9NPY3 | 52 | GC20M023067 | 0.733661949634552 | https://www.genecards.org/cgi-bin/carddisp.pl?gene=CD93 |
| HLA-DMB | Major Histocompatibility Complex, Class II, DM Beta | Protein Coding | P28068 | 52 | GC06M032934 | 0.733661949634552 | https://www.genecards.org/cgi-bin/carddisp.pl?gene=HLA-DMB |
| HLA-DPA1 | Major Histocompatibility Complex, Class II, DP Alpha 1 | Protein Coding | P20036 | 52 | GC06M033064 | 0.733661949634552 | https://www.genecards.org/cgi-bin/carddisp.pl?gene=HLA-DPA1 |
| HLA-DRB5 | Major Histocompatibility Complex, Class II, DR Beta 5 | Protein Coding | Q30154 | 52 | GC06M106524 | 0.733661949634552 | https://www.genecards.org/cgi-bin/carddisp.pl?gene=HLA-DRB5 |
| RNF125 | Ring Finger Protein 125 | Protein Coding | Q96EQ8 | 52 | GC18P032633 | 0.733661949634552 | https://www.genecards.org/cgi-bin/carddisp.pl?gene=RNF125 |
| SORCS1 | Sortilin Related VPS10 Domain Containing Receptor 1 | Protein Coding | Q8WY21 | 52 | GC10M106573 | 0.733661949634552 | https://www.genecards.org/cgi-bin/carddisp.pl?gene=SORCS1 |
| SYN2 | Synapsin II | Protein Coding | Q92777 | 52 | GC03P027051 | 0.733661949634552 | https://www.genecards.org/cgi-bin/carddisp.pl?gene=SYN2 |
| TIMP4 | TIMP Metallopeptidase Inhibitor 4 | Protein Coding | Q99727 | 52 | GC03M012153 | 0.733661949634552 | https://www.genecards.org/cgi-bin/carddisp.pl?gene=TIMP4 |
| HLA-DMA | Major Histocompatibility Complex, Class II, DM Alpha | Protein Coding | P28067 | 51 | GC06M106554 | 0.733661949634552 | https://www.genecards.org/cgi-bin/carddisp.pl?gene=HLA-DMA |
| HLA-DOB | Major Histocompatibility Complex, Class II, DO Beta | Protein Coding | P13765 | 51 | GC06M106545 | 0.733661949634552 | https://www.genecards.org/cgi-bin/carddisp.pl?gene=HLA-DOB |
| NAPB | NSF Attachment Protein Beta | Protein Coding | Q9H115 | 51 | GC20M023374 | 0.733661949634552 | https://www.genecards.org/cgi-bin/carddisp.pl?gene=NAPB |
| RBM28 | RNA Binding Motif Protein 28 | Protein Coding | Q9NW13 | 51 | GC07M129312 | 0.733661949634552 | https://www.genecards.org/cgi-bin/carddisp.pl?gene=RBM28 |
| SMOC2 | SPARC Related Modular Calcium Binding 2 | Protein Coding | Q9H3U7 | 51 | GC06P168441 | 0.733661949634552 | https://www.genecards.org/cgi-bin/carddisp.pl?gene=SMOC2 |
| UPF2 | UPF2 Regulator Of Nonsense Mediated MRNA Decay | Protein Coding | Q9HAU5 | 51 | GC10M011920 | 0.733661949634552 | https://www.genecards.org/cgi-bin/carddisp.pl?gene=UPF2 |
| CHD9 | Chromodomain Helicase DNA Binding Protein 9 | Protein Coding | Q3L8U1 | 50 | GC16P053054 | 0.733661949634552 | https://www.genecards.org/cgi-bin/carddisp.pl?gene=CHD9 |
| CSAD | Cysteine Sulfinic Acid Decarboxylase | Protein Coding | Q9Y600 | 50 | GC12M053160 | 0.733661949634552 | https://www.genecards.org/cgi-bin/carddisp.pl?gene=CSAD |
| DNAH8 | Dynein Axonemal Heavy Chain 8 | Protein Coding | Q96JB1 | 50 | GC06P182037 | 0.733661949634552 | https://www.genecards.org/cgi-bin/carddisp.pl?gene=DNAH8 |
| EXOC6 | Exocyst Complex Component 6 | Protein Coding | Q8TAG9 | 50 | GC10P092826 | 0.733661949634552 | https://www.genecards.org/cgi-bin/carddisp.pl?gene=EXOC6 |
| HLA-DQA2 | Major Histocompatibility Complex, Class II, DQ Alpha 2 | Protein Coding | P01906 | 50 | GC06P032741 | 0.733661949634552 | https://www.genecards.org/cgi-bin/carddisp.pl?gene=HLA-DQA2 |
| CDC123 | Cell Division Cycle 123 | Protein Coding | O75794 | 49 | GC10P012195 | 0.733661949634552 | https://www.genecards.org/cgi-bin/carddisp.pl?gene=CDC123 |
| HLA-DQB2 | Major Histocompatibility Complex, Class II, DQ Beta 2 | Protein Coding | P05538 | 49 | GC06M032756 | 0.733661949634552 | https://www.genecards.org/cgi-bin/carddisp.pl?gene=HLA-DQB2 |
| CSNK1A1L | Casein Kinase 1 Alpha 1 Like | Protein Coding | Q8N752 | 48 | GC13M037103 | 0.733661949634552 | https://www.genecards.org/cgi-bin/carddisp.pl?gene=CSNK1A1L |
| G6PC2 | Glucose-6-Phosphatase Catalytic Subunit 2 | Protein Coding | Q9NQR9 | 48 | GC02P168901 | 0.733661949634552 | https://www.genecards.org/cgi-bin/carddisp.pl?gene=G6PC2 |
| GLIS3 | GLIS Family Zinc Finger 3 | Protein Coding | Q8NEA6 | 48 | GC09M003816 | 0.733661949634552 | https://www.genecards.org/cgi-bin/carddisp.pl?gene=GLIS3 |
| PDZD2 | PDZ Domain Containing 2 | Protein Coding | O15018 | 48 | GC05P031639 | 0.733661949634552 | https://www.genecards.org/cgi-bin/carddisp.pl?gene=PDZD2 |
| SFXN1 | Sideroflexin 1 | Protein Coding | Q9H9B4 | 48 | GC05P175477 | 0.733661949634552 | https://www.genecards.org/cgi-bin/carddisp.pl?gene=SFXN1 |
| ABCA9 | ATP Binding Cassette Subfamily A Member 9 | Protein Coding | Q8IUA7 | 47 | GC17M068974 | 0.733661949634552 | https://www.genecards.org/cgi-bin/carddisp.pl?gene=ABCA9 |
| BICC1 | BicC Family RNA Binding Protein 1 | Protein Coding | Q9H694 | 47 | GC10P058513 | 0.733661949634552 | https://www.genecards.org/cgi-bin/carddisp.pl?gene=BICC1 |
| DCUN1D1 | Defective In Cullin Neddylation 1 Domain Containing 1 | Protein Coding | Q96GG9 | 47 | GC03M182938 | 0.733661949634552 | https://www.genecards.org/cgi-bin/carddisp.pl?gene=DCUN1D1 |
| HLA-DOA | Major Histocompatibility Complex, Class II, DO Alpha | Protein Coding | P06340 | 47 | GC06M033004 | 0.733661949634552 | https://www.genecards.org/cgi-bin/carddisp.pl?gene=HLA-DOA |
| PATJ | PATJ Crumbs Cell Polarity Complex Component | Protein Coding | Q8NI35 | 47 | GC01P078703 | 0.733661949634552 | https://www.genecards.org/cgi-bin/carddisp.pl?gene=PATJ |
| CPNE4 | Copine 4 | Protein Coding | Q96A23 | 46 | GC03M131533 | 0.733661949634552 | https://www.genecards.org/cgi-bin/carddisp.pl?gene=CPNE4 |
| NXT1 | Nuclear Transport Factor 2 Like Export Factor 1 | Protein Coding | Q9UKK6 | 46 | GC20P023350 | 0.733661949634552 | https://www.genecards.org/cgi-bin/carddisp.pl?gene=NXT1 |
| GPR15 | G Protein-Coupled Receptor 15 | Protein Coding | P49685 | 45 | GC03P098531 | 0.733661949634552 | https://www.genecards.org/cgi-bin/carddisp.pl?gene=GPR15 |
| NCR3LG1 | Natural Killer Cell Cytotoxicity Receptor 3 Ligand 1 | Protein Coding | Q68D85 | 45 | GC11P017351 | 0.733661949634552 | https://www.genecards.org/cgi-bin/carddisp.pl?gene=NCR3LG1 |
| TRAPPC8 | Trafficking Protein Particle Complex Subunit 8 | Protein Coding | Q9Y2L5 | 45 | GC18M031829 | 0.733661949634552 | https://www.genecards.org/cgi-bin/carddisp.pl?gene=TRAPPC8 |
| ALG10 | ALG10 Alpha-1,2-Glucosyltransferase | Protein Coding | Q5BKT4 | 44 | GC12P034022 | 0.733661949634552 | https://www.genecards.org/cgi-bin/carddisp.pl?gene=ALG10 |
| KLHL31 | Kelch Like Family Member 31 | Protein Coding | Q9H511 | 44 | GC06M053647 | 0.733661949634552 | https://www.genecards.org/cgi-bin/carddisp.pl?gene=KLHL31 |
| SEC61A2 | SEC61 Translocon Subunit Alpha 2 | Protein Coding | Q9H9S3 | 44 | GC10P012130 | 0.733661949634552 | https://www.genecards.org/cgi-bin/carddisp.pl?gene=SEC61A2 |
| GPSM3 | G Protein Signaling Modulator 3 | Protein Coding | Q9Y4H4 | 43 | GC06M106507 | 0.733661949634552 | https://www.genecards.org/cgi-bin/carddisp.pl?gene=GPSM3 |
| ACTL9 | Actin Like 9 | Protein Coding | Q8TC94 | 42 | GC19M008697 | 0.733661949634552 | https://www.genecards.org/cgi-bin/carddisp.pl?gene=ACTL9 |
| SPDL1 | Spindle Apparatus Coiled-Coil Protein 1 | Protein Coding | Q96EA4 | 42 | GC05P169583 | 0.733661949634552 | https://www.genecards.org/cgi-bin/carddisp.pl?gene=SPDL1 |
| L1TD1 | LINE1 Type Transposase Domain Containing 1 | Protein Coding | Q5T7N2 | 41 | GC01P062194 | 0.733661949634552 | https://www.genecards.org/cgi-bin/carddisp.pl?gene=L1TD1 |
| NYAP2 | Neuronal Tyrosine-Phosphorylated Phosphoinositide-3-Kinase Adaptor 2 | Protein Coding | Q9P242 | 41 | GC02P225400 | 0.733661949634552 | https://www.genecards.org/cgi-bin/carddisp.pl?gene=NYAP2 |
| PRSS37 | Serine Protease 37 | Protein Coding | A4D1T9 | 40 | GC07M141836 | 0.733661949634552 | https://www.genecards.org/cgi-bin/carddisp.pl?gene=PRSS37 |
| SPC25 | SPC25 Component Of NDC80 Kinetochore Complex | Protein Coding | Q9HBM1 | 40 | GC02M168834 | 0.733661949634552 | https://www.genecards.org/cgi-bin/carddisp.pl?gene=SPC25 |
| ADGB | Androglobin | Protein Coding | Q8N7X0 | 38 | GC06P146598 | 0.733661949634552 | https://www.genecards.org/cgi-bin/carddisp.pl?gene=ADGB |
| C1orf94 | Chromosome 1 Open Reading Frame 94 | Protein Coding | Q6P1W5 | 36 | GC01P034166 | 0.733661949634552 | https://www.genecards.org/cgi-bin/carddisp.pl?gene=C1orf94 |
| INSYN2B | Inhibitory Synaptic Factor Family Member 2B | Protein Coding | A6NMK8 | 31 | GC05M169861 | 0.733661949634552 | https://www.genecards.org/cgi-bin/carddisp.pl?gene=INSYN2B |
| ST20-MTHFS | ST20-MTHFS Readthrough | Protein Coding |  | 27 | GC15M162932 | 0.733661949634552 | https://www.genecards.org/cgi-bin/carddisp.pl?gene=ST20-MTHFS |
| LINC01564 | Long Intergenic Non-Protein Coding RNA 1564 | RNA Gene |  | 24 | GC06P182184 | 0.733661949634552 | https://www.genecards.org/cgi-bin/carddisp.pl?gene=LINC01564 |
| MIR552 | MicroRNA 552 | RNA Gene |  | 21 | GC01M034669 | 0.733661949634552 | https://www.genecards.org/cgi-bin/carddisp.pl?gene=MIR552 |
| LINC01307 | Long Intergenic Non-Protein Coding RNA 1307 | RNA Gene |  | 18 | GC01P101323 | 0.733661949634552 | https://www.genecards.org/cgi-bin/carddisp.pl?gene=LINC01307 |
| HLA-DRB6 | Major Histocompatibility Complex, Class II, DR Beta 6 (Pseudogene) | Pseudogene |  | 17 | GC06M106526 | 0.733661949634552 | https://www.genecards.org/cgi-bin/carddisp.pl?gene=HLA-DRB6 |
| DNAH8-AS1 | DNAH8 Antisense RNA 1 | RNA Gene |  | 16 | GC06M108641 | 0.733661949634552 | https://www.genecards.org/cgi-bin/carddisp.pl?gene=DNAH8-AS1 |
| HLA-DQB1-AS1 | HLA-DQB1 Antisense RNA 1 | RNA Gene |  | 16 | GC06P032659 | 0.733661949634552 | https://www.genecards.org/cgi-bin/carddisp.pl?gene=HLA-DQB1-AS1 |
| MIR548Q | MicroRNA 548q | RNA Gene |  | 16 | GC10M012725 | 0.733661949634552 | https://www.genecards.org/cgi-bin/carddisp.pl?gene=MIR548Q |
| LINC00656 | Long Intergenic Non-Protein Coding RNA 656 | RNA Gene |  | 15 | GC20M023122 | 0.733661949634552 | https://www.genecards.org/cgi-bin/carddisp.pl?gene=LINC00656 |
| ABCA9-AS1 | ABCA9 Antisense RNA 1 | RNA Gene |  | 14 | GC17P068944 | 0.733661949634552 | https://www.genecards.org/cgi-bin/carddisp.pl?gene=ABCA9-AS1 |
| AMZ2P1 | AMZ2 Pseudogene 1 | Pseudogene |  | 14 | GC17M064966 | 0.733661949634552 | https://www.genecards.org/cgi-bin/carddisp.pl?gene=AMZ2P1 |
| ELOA-AS1 | ELOA Antisense RNA 1 | RNA Gene |  | 14 | GC01M034561 | 0.733661949634552 | https://www.genecards.org/cgi-bin/carddisp.pl?gene=ELOA-AS1 |
| HSFY1P1 | HSFY1 Pseudogene 1 | Pseudogene |  | 14 | GC22P016827 | 0.733661949634552 | https://www.genecards.org/cgi-bin/carddisp.pl?gene=HSFY1P1 |
| LINC01048 | Long Intergenic Non-Protein Coding RNA 1048 | RNA Gene |  | 14 | GC13M043033 | 0.733661949634552 | https://www.genecards.org/cgi-bin/carddisp.pl?gene=LINC01048 |
| LINC02885 | Long Intergenic Non-Protein Coding RNA 2885 | RNA Gene |  | 14 | GC22M086078 | 0.733661949634552 | https://www.genecards.org/cgi-bin/carddisp.pl?gene=LINC02885 |
| MIR4432HG | MIR4432 Host Gene | RNA Gene |  | 14 | GC02M060318 | 0.733661949634552 | https://www.genecards.org/cgi-bin/carddisp.pl?gene=MIR4432HG |
| LINC02987 | Long Intergenic Non-Protein Coding RNA 2987 | RNA Gene |  | 13 | GC19P154068 | 0.733661949634552 | https://www.genecards.org/cgi-bin/carddisp.pl?gene=LINC02987 |
| LINC01709 | Long Intergenic Non-Protein Coding RNA 1709 | RNA Gene |  | 12 | GC01P101600 | 0.733661949634552 | https://www.genecards.org/cgi-bin/carddisp.pl?gene=LINC01709 |
| LINC02370 | Long Intergenic Non-Protein Coding RNA 2370 | RNA Gene |  | 12 | GC12P139098 | 0.733661949634552 | https://www.genecards.org/cgi-bin/carddisp.pl?gene=LINC02370 |
| LINC02414 | Long Intergenic Non-Protein Coding RNA 2414 | RNA Gene |  | 12 | GC12M131642 | 0.733661949634552 | https://www.genecards.org/cgi-bin/carddisp.pl?gene=LINC02414 |
| MIR5702 | MicroRNA 5702 | RNA Gene |  | 12 | GC02M226658 | 0.733661949634552 | https://www.genecards.org/cgi-bin/carddisp.pl?gene=MIR5702 |
| ARHGAP28-AS1 | ARHGAP28 Antisense RNA 1 | RNA Gene |  | 11 | GC18M006728 | 0.733661949634552 | https://www.genecards.org/cgi-bin/carddisp.pl?gene=ARHGAP28-AS1 |
| KATNBL1P6 | Katanin Regulatory Subunit B1 Like 1 Pseudogene 6 | Pseudogene |  | 11 | GC06M146801 | 0.733661949634552 | https://www.genecards.org/cgi-bin/carddisp.pl?gene=KATNBL1P6 |
| LINC02743 | Long Intergenic Non-Protein Coding RNA 2743 | RNA Gene |  | 11 | GC11M140886 | 0.733661949634552 | https://www.genecards.org/cgi-bin/carddisp.pl?gene=LINC02743 |
| RNU6-1177P | RNA, U6 Small Nuclear 1177, Pseudogene | Pseudogene |  | 11 | GC01P061852 | 0.733661949634552 | https://www.genecards.org/cgi-bin/carddisp.pl?gene=RNU6-1177P |
| RPS15AP30 | Ribosomal Protein S15a Pseudogene 30 | Pseudogene |  | 11 | GC10P112987 | 0.733661949634552 | https://www.genecards.org/cgi-bin/carddisp.pl?gene=RPS15AP30 |
| SLIT3-AS1 | SLIT3 Antisense RNA 1 | RNA Gene |  | 11 | GC05P169014 | 0.733661949634552 | https://www.genecards.org/cgi-bin/carddisp.pl?gene=SLIT3-AS1 |
| ENSG00000237852 | Novel Transcript | RNA Gene |  | 10 | GC01P078819 | 0.733661949634552 | https://www.genecards.org/cgi-bin/carddisp.pl?gene=ENSG00000237852 |
| ENSG00000253269 | Novel Transcript | RNA Gene |  | 10 | GC05M169772 | 0.733661949634552 | https://www.genecards.org/cgi-bin/carddisp.pl?gene=ENSG00000253269 |
| PGDP1 | Phosphogluconate Dehydrogenase Pseudogene 1 | Pseudogene |  | 10 | GC18P031962 | 0.733661949634552 | https://www.genecards.org/cgi-bin/carddisp.pl?gene=PGDP1 |
| RN7SL282P | RNA, 7SL, Cytoplasmic 282, Pseudogene | Pseudogene |  | 10 | GC18P006602 | 0.733661949634552 | https://www.genecards.org/cgi-bin/carddisp.pl?gene=RN7SL282P |
| SLC16A6P1 | SLC16A6 Pseudogene 1 | Pseudogene |  | 10 | GC17P064953 | 0.733661949634552 | https://www.genecards.org/cgi-bin/carddisp.pl?gene=SLC16A6P1 |
| OR9A3P | Olfactory Receptor Family 9 Subfamily A Member 3 Pseudogene | Pseudogene |  | 8 | GC07P167670 | 0.733661949634552 | https://www.genecards.org/cgi-bin/carddisp.pl?gene=OR9A3P |
| RN7SKP167 | RN7SK Pseudogene 167 | Pseudogene |  | 8 | GC10P121410 | 0.733661949634552 | https://www.genecards.org/cgi-bin/carddisp.pl?gene=RN7SKP167 |
| RN7SL198P | RNA, 7SL, Cytoplasmic 198, Pseudogene | Pseudogene |  | 8 | GC10M012334 | 0.733661949634552 | https://www.genecards.org/cgi-bin/carddisp.pl?gene=RN7SL198P |
| RSL24D1P4 | Ribosomal L24 Domain Containing 1 Pseudogene 4 | Pseudogene |  | 8 | GC01P242767 | 0.733661949634552 | https://www.genecards.org/cgi-bin/carddisp.pl?gene=RSL24D1P4 |
| SPESP1-NOX5 | SPESP1-NOX5 Readthrough | Protein Coding |  | 8 | GC15P200888 | 0.733661949634552 | https://www.genecards.org/cgi-bin/carddisp.pl?gene=SPESP1-NOX5 |
| ACO2P1 | ACO2 Pseudogene 1 | Pseudogene |  | 7 | GC22M086288 | 0.733661949634552 | https://www.genecards.org/cgi-bin/carddisp.pl?gene=ACO2P1 |
| GPM6BP3 | Glycoprotein M6B Pseudogene 3 | Pseudogene |  | 7 | GC22P016837 | 0.733661949634552 | https://www.genecards.org/cgi-bin/carddisp.pl?gene=GPM6BP3 |
| RN7SL854P | RNA, 7SL, Cytoplasmic 854, Pseudogene | Pseudogene |  | 7 | GC01M065761 | 0.733661949634552 | https://www.genecards.org/cgi-bin/carddisp.pl?gene=RN7SL854P |
| SEPTIN14P21 | Septin 14 Pseudogene 21 | Pseudogene |  | 7 | GC01M244794 | 0.733661949634552 | https://www.genecards.org/cgi-bin/carddisp.pl?gene=SEPTIN14P21 |
| TUBB8P4 | Tubulin Beta 8 Class VIII Pseudogene 4 | Pseudogene |  | 7 | GC12M034164 | 0.733661949634552 | https://www.genecards.org/cgi-bin/carddisp.pl?gene=TUBB8P4 |
| MAN1A2P1 | Mannosidase Alpha Class 1A Member 2 Pseudogene 1 | Pseudogene |  | 6 | GC19M028790 | 0.733661949634552 | https://www.genecards.org/cgi-bin/carddisp.pl?gene=MAN1A2P1 |
| RNA5SP94 | RNA, 5S Ribosomal Pseudogene 94 | Pseudogene |  | 6 | GC02P059694 | 0.733661949634552 | https://www.genecards.org/cgi-bin/carddisp.pl?gene=RNA5SP94 |
| RPL38P4 | RPL38 Pseudogene 4 | Pseudogene |  | 6 | GC03M098525 | 0.733661949634552 | https://www.genecards.org/cgi-bin/carddisp.pl?gene=RPL38P4 |
| lnc-CDC123-2 |  | RNA Gene |  | 6 | GC10P012264 | 0.733661949634552 | https://www.genecards.org/cgi-bin/carddisp.pl?gene=lnc-CDC123-2 |
| lnc-EXOSC8-1 |  | RNA Gene |  | 6 | GC13P037091 | 0.733661949634552 | https://www.genecards.org/cgi-bin/carddisp.pl?gene=lnc-EXOSC8-1 |
| HSALNG0149897 |  | RNA Gene |  | 5 | GC07M029163 | 0.733661949634552 | https://www.genecards.org/cgi-bin/carddisp.pl?gene=HSALNG0149897 |
| UBE2V1P14 | UBE2V1 Pseudogene 14 | Pseudogene |  | 5 | GC02P154588 | 0.733661949634552 | https://www.genecards.org/cgi-bin/carddisp.pl?gene=UBE2V1P14 |
| lnc-CD93-6 |  | RNA Gene |  | 5 | GC20M023114 | 0.733661949634552 | https://www.genecards.org/cgi-bin/carddisp.pl?gene=lnc-CD93-6 |
| lnc-EXOSC8-5 |  | RNA Gene |  | 5 | GC13P037465 | 0.733661949634552 | https://www.genecards.org/cgi-bin/carddisp.pl?gene=lnc-EXOSC8-5 |
| lnc-HLA-DQA1-9 |  | RNA Gene |  | 5 | GC06P185321 | 0.733661949634552 | https://www.genecards.org/cgi-bin/carddisp.pl?gene=lnc-HLA-DQA1-9 |
| lnc-HLA-DRB1-7 |  | RNA Gene |  | 5 | GC06M032609 | 0.733661949634552 | https://www.genecards.org/cgi-bin/carddisp.pl?gene=lnc-HLA-DRB1-7 |
| lnc-RGS9-6 |  | RNA Gene |  | 5 | GC17P065076 | 0.733661949634552 | https://www.genecards.org/cgi-bin/carddisp.pl?gene=lnc-RGS9-6 |
| lnc-RGS9-7 |  | RNA Gene |  | 5 | GC17P155415 | 0.733661949634552 | https://www.genecards.org/cgi-bin/carddisp.pl?gene=lnc-RGS9-7 |
| lnc-TIMP4-4 |  | RNA Gene |  | 5 | GC03M012212 | 0.733661949634552 | https://www.genecards.org/cgi-bin/carddisp.pl?gene=lnc-TIMP4-4 |
| piR-39858-354 |  | RNA Gene |  | 5 | GC16M053827 | 0.733661949634552 | https://www.genecards.org/cgi-bin/carddisp.pl?gene=piR-39858-354 |
| piR-53029-031 |  | RNA Gene |  | 5 | GC13M037505 | 0.733661949634552 | https://www.genecards.org/cgi-bin/carddisp.pl?gene=piR-53029-031 |
| piR-57133-040 |  | RNA Gene |  | 5 | GC10M012271 | 0.733661949634552 | https://www.genecards.org/cgi-bin/carddisp.pl?gene=piR-57133-040 |
| ENSG00000280380 | TEC | Uncategorized |  | 4 | GC18P031982 | 0.733661949634552 | https://www.genecards.org/cgi-bin/carddisp.pl?gene=ENSG00000280380 |
| ENSG00000298947 | Novel Transcript | RNA Gene |  | 4 | GC17M098455 | 0.733661949634552 | https://www.genecards.org/cgi-bin/carddisp.pl?gene=ENSG00000298947 |
| ENSG00000301169 | Novel Transcript, Sense Intronic To PATJ | RNA Gene |  | 4 | GC01P079095 | 0.733661949634552 | https://www.genecards.org/cgi-bin/carddisp.pl?gene=ENSG00000301169 |
| ENSG00000307651 | Novel Transcript | RNA Gene |  | 4 | GC13P037203 | 0.733661949634552 | https://www.genecards.org/cgi-bin/carddisp.pl?gene=ENSG00000307651 |
| ENSG00000307774 | Novel Transcript | RNA Gene |  | 4 | GC20P023106 | 0.733661949634552 | https://www.genecards.org/cgi-bin/carddisp.pl?gene=ENSG00000307774 |
| ENSG00000307907 | Novel Transcript | RNA Gene |  | 4 | GC06P184637 | 0.733661949634552 | https://www.genecards.org/cgi-bin/carddisp.pl?gene=ENSG00000307907 |
| HSALNG0024198 |  | RNA Gene |  | 4 | GC03P012148 | 0.733661949634552 | https://www.genecards.org/cgi-bin/carddisp.pl?gene=HSALNG0024198 |
| HSALNG0031133 |  | RNA Gene |  | 4 | GC03P185694 | 0.733661949634552 | https://www.genecards.org/cgi-bin/carddisp.pl?gene=HSALNG0031133 |
| HSALNG0049423 |  | RNA Gene |  | 4 | GC06P184866 | 0.733661949634552 | https://www.genecards.org/cgi-bin/carddisp.pl?gene=HSALNG0049423 |
| HSALNG0049424 |  | RNA Gene |  | 4 | GC06M109373 | 0.733661949634552 | https://www.genecards.org/cgi-bin/carddisp.pl?gene=HSALNG0049424 |
| HSALNG0080722 |  | RNA Gene |  | 4 | GC10M113004 | 0.733661949634552 | https://www.genecards.org/cgi-bin/carddisp.pl?gene=HSALNG0080722 |
| HSALNG0111532 |  | RNA Gene |  | 4 | GC16M053741 | 0.733661949634552 | https://www.genecards.org/cgi-bin/carddisp.pl?gene=HSALNG0111532 |
| LOC105378316 | Uncharacterized LOC105378316 | RNA Gene |  | 4 | GC10P058433 | 0.733661949634552 | https://www.genecards.org/cgi-bin/carddisp.pl?gene=LOC105378316 |
| LOC124903159 | Uncharacterized LOC124903159 | RNA Gene |  | 4 | GC13P037362 | 0.733661949634552 | https://www.genecards.org/cgi-bin/carddisp.pl?gene=LOC124903159 |
| LOC124903691 | Uncharacterized LOC124903691 | RNA Gene |  | 4 | GC16M053822 | 0.733661949634552 | https://www.genecards.org/cgi-bin/carddisp.pl?gene=LOC124903691 |
| lnc-ABCC8-1 |  | RNA Gene |  | 4 | GC11M017386 | 0.733661949634552 | https://www.genecards.org/cgi-bin/carddisp.pl?gene=lnc-ABCC8-1 |
| piR-61288-001 |  | RNA Gene |  | 4 | GC01M065531 | 0.733661949634552 | https://www.genecards.org/cgi-bin/carddisp.pl?gene=piR-61288-001 |
| HSALNG0004202 |  | RNA Gene |  | 3 | GC01M065473 | 0.733661949634552 | https://www.genecards.org/cgi-bin/carddisp.pl?gene=HSALNG0004202 |
| HSALNG0141786 |  | RNA Gene |  | 3 | GC01P065471 | 0.733661949634552 | https://www.genecards.org/cgi-bin/carddisp.pl?gene=HSALNG0141786 |
| LOC105370084 | Uncharacterized LOC105370084 | RNA Gene |  | 3 | GC12M131583 | 0.733661949634552 | https://www.genecards.org/cgi-bin/carddisp.pl?gene=LOC105370084 |
| piR-30188-083 |  | RNA Gene |  | 3 | GC18P032000 | 0.733661949634552 | https://www.genecards.org/cgi-bin/carddisp.pl?gene=piR-30188-083 |
| piR-61240-006 |  | RNA Gene |  | 3 | GC01P065467 | 0.733661949634552 | https://www.genecards.org/cgi-bin/carddisp.pl?gene=piR-61240-006 |
| LOC105375835 | Uncharacterized LOC105375835 | RNA Gene |  | 2 | GC08P052744 | 0.733661949634552 | https://www.genecards.org/cgi-bin/carddisp.pl?gene=LOC105375835 |
| LOC105378641 | Uncharacterized LOC105378641 | RNA Gene |  | 2 | GC01M034457 | 0.733661949634552 | https://www.genecards.org/cgi-bin/carddisp.pl?gene=LOC105378641 |
| lnc-ABCA8-4 |  | RNA Gene |  | 2 | GC17M068989 | 0.733661949634552 | https://www.genecards.org/cgi-bin/carddisp.pl?gene=lnc-ABCA8-4 |
| NR1H2 | Nuclear Receptor Subfamily 1 Group H Member 2 | Protein Coding | P55055 | 59 | GC19P050329 | 0.723829746246338 | https://www.genecards.org/cgi-bin/carddisp.pl?gene=NR1H2 |
| CMKLR1 | Chemerin Chemokine-Like Receptor 1 | Protein Coding | Q99788 | 50 | GC12M108288 | 0.723829746246338 | https://www.genecards.org/cgi-bin/carddisp.pl?gene=CMKLR1 |
| FOXC1 | Forkhead Box C1 | Protein Coding | Q12948 | 55 | GC06P002084 | 0.715954720973969 | https://www.genecards.org/cgi-bin/carddisp.pl?gene=FOXC1 |
| CAMK2A | Calcium/Calmodulin Dependent Protein Kinase II Alpha | Protein Coding | Q9UQM7 | 65 | GC05M150219 | 0.705009937286377 | https://www.genecards.org/cgi-bin/carddisp.pl?gene=CAMK2A |
| DNMT3A | DNA Methyltransferase 3 Alpha | Protein Coding | Q9Y6K1 | 65 | GC02M025228 | 0.705009937286377 | https://www.genecards.org/cgi-bin/carddisp.pl?gene=DNMT3A |
| DNMT3B | DNA Methyltransferase 3 Beta | Protein Coding | Q9UBC3 | 64 | GC20P032762 | 0.705009937286377 | https://www.genecards.org/cgi-bin/carddisp.pl?gene=DNMT3B |
| GRIA2 | Glutamate Ionotropic Receptor AMPA Type Subunit 2 | Protein Coding | P42262 | 64 | GC04P157204 | 0.705009937286377 | https://www.genecards.org/cgi-bin/carddisp.pl?gene=GRIA2 |
| PRKDC | Protein Kinase, DNA-Activated, Catalytic Subunit | Protein Coding | P78527 | 63 | GC08M047773 | 0.705009937286377 | https://www.genecards.org/cgi-bin/carddisp.pl?gene=PRKDC |
| PRKG1 | Protein Kinase CGMP-Dependent 1 | Protein Coding | Q13976 | 63 | GC10P052996 | 0.705009937286377 | https://www.genecards.org/cgi-bin/carddisp.pl?gene=PRKG1 |
| CACNA1C | Calcium Voltage-Gated Channel Subunit Alpha1 C | Protein Coding | Q13936 | 62 | GC12P001970 | 0.705009937286377 | https://www.genecards.org/cgi-bin/carddisp.pl?gene=CACNA1C |
| CAMK2B | Calcium/Calmodulin Dependent Protein Kinase II Beta | Protein Coding | Q13554 | 62 | GC07M045687 | 0.705009937286377 | https://www.genecards.org/cgi-bin/carddisp.pl?gene=CAMK2B |
| GABRB3 | Gamma-Aminobutyric Acid Type A Receptor Subunit Beta3 | Protein Coding | P28472 | 62 | GC15M026543 | 0.705009937286377 | https://www.genecards.org/cgi-bin/carddisp.pl?gene=GABRB3 |
| GSR | Glutathione-Disulfide Reductase | Protein Coding | P00390 | 62 | GC08M030678 | 0.705009937286377 | https://www.genecards.org/cgi-bin/carddisp.pl?gene=GSR |
| ITPR1 | Inositol 1,4,5-Trisphosphate Receptor Type 1 | Protein Coding | Q14643 | 62 | GC03P004496 | 0.705009937286377 | https://www.genecards.org/cgi-bin/carddisp.pl?gene=ITPR1 |
| NTRK1 | Neurotrophic Receptor Tyrosine Kinase 1 | Protein Coding | P04629 | 62 | GC01P156815 | 0.705009937286377 | https://www.genecards.org/cgi-bin/carddisp.pl?gene=NTRK1 |
| PRKN | Parkin RBR E3 Ubiquitin Protein Ligase | Protein Coding | O60260 | 62 | GC06M161348 | 0.705009937286377 | https://www.genecards.org/cgi-bin/carddisp.pl?gene=PRKN |
| SLC12A2 | Solute Carrier Family 12 Member 2 | Protein Coding | P55011 | 62 | GC05P128083 | 0.705009937286377 | https://www.genecards.org/cgi-bin/carddisp.pl?gene=SLC12A2 |
| SMARCA2 | SWI/SNF Related BAF Chromatin Remodeling Complex Subunit ATPase 2 | Protein Coding | P51531 | 62 | GC09P001980 | 0.705009937286377 | https://www.genecards.org/cgi-bin/carddisp.pl?gene=SMARCA2 |
| CHAT | Choline O-Acetyltransferase | Protein Coding | P28329 | 61 | GC10P049609 | 0.705009937286377 | https://www.genecards.org/cgi-bin/carddisp.pl?gene=CHAT |
| DLG4 | Discs Large MAGUK Scaffold Protein 4 | Protein Coding | P78352 | 61 | GC17M096179 | 0.705009937286377 | https://www.genecards.org/cgi-bin/carddisp.pl?gene=DLG4 |
| HPRT1 | Hypoxanthine Phosphoribosyltransferase 1 | Protein Coding | P00492 | 61 | GC0XP134460 | 0.705009937286377 | https://www.genecards.org/cgi-bin/carddisp.pl?gene=HPRT1 |
| KCNQ2 | Potassium Voltage-Gated Channel Subfamily Q Member 2 | Protein Coding | O43526 | 61 | GC20M063400 | 0.705009937286377 | https://www.genecards.org/cgi-bin/carddisp.pl?gene=KCNQ2 |
| KDM1A | Lysine Demethylase 1A | Protein Coding | O60341 | 61 | GC01P023019 | 0.705009937286377 | https://www.genecards.org/cgi-bin/carddisp.pl?gene=KDM1A |
| LEF1 | Lymphoid Enhancer Binding Factor 1 | Protein Coding | Q9UJU2 | 61 | GC04M108047 | 0.705009937286377 | https://www.genecards.org/cgi-bin/carddisp.pl?gene=LEF1 |
| NRXN1 | Neurexin 1 | Protein Coding | Q9ULB1 | 61 | GC02M049918 | 0.705009937286377 | https://www.genecards.org/cgi-bin/carddisp.pl?gene=NRXN1 |
| RB1 | RB Transcriptional Corepressor 1 | Protein Coding | P06400 | 61 | GC13P048303 | 0.705009937286377 | https://www.genecards.org/cgi-bin/carddisp.pl?gene=RB1 |
| SCN1A | Sodium Voltage-Gated Channel Alpha Subunit 1 | Protein Coding | P35498 | 61 | GC02M165989 | 0.705009937286377 | https://www.genecards.org/cgi-bin/carddisp.pl?gene=SCN1A |
| SCN2A | Sodium Voltage-Gated Channel Alpha Subunit 2 | Protein Coding | Q99250 | 61 | GC02P165194 | 0.705009937286377 | https://www.genecards.org/cgi-bin/carddisp.pl?gene=SCN2A |
| SLC12A5 | Solute Carrier Family 12 Member 5 | Protein Coding | Q9H2X9 | 61 | GC20P046021 | 0.705009937286377 | https://www.genecards.org/cgi-bin/carddisp.pl?gene=SLC12A5 |
| UBA1 | Ubiquitin Like Modifier Activating Enzyme 1 | Protein Coding | P22314 | 61 | GC0XP047190 | 0.705009937286377 | https://www.genecards.org/cgi-bin/carddisp.pl?gene=UBA1 |
| UBE3A | Ubiquitin Protein Ligase E3A | Protein Coding | Q05086 | 61 | GC15M025333 | 0.705009937286377 | https://www.genecards.org/cgi-bin/carddisp.pl?gene=UBE3A |
| ACAN | Aggrecan | Protein Coding | P16112 | 60 | GC15P193892 | 0.705009937286377 | https://www.genecards.org/cgi-bin/carddisp.pl?gene=ACAN |
| ATRX | ATRX Chromatin Remodeler | Protein Coding | P46100 | 60 | GC0XM077504 | 0.705009937286377 | https://www.genecards.org/cgi-bin/carddisp.pl?gene=ATRX |
| AVPR2 | Arginine Vasopressin Receptor 2 | Protein Coding | P30518 | 60 | GC0XP153902 | 0.705009937286377 | https://www.genecards.org/cgi-bin/carddisp.pl?gene=AVPR2 |
| COL4A1 | Collagen Type IV Alpha 1 Chain | Protein Coding | P02462 | 60 | GC13M110148 | 0.705009937286377 | https://www.genecards.org/cgi-bin/carddisp.pl?gene=COL4A1 |
| GRM5 | Glutamate Metabotropic Receptor 5 | Protein Coding | P41594 | 60 | GC11M088504 | 0.705009937286377 | https://www.genecards.org/cgi-bin/carddisp.pl?gene=GRM5 |
| L1CAM | L1 Cell Adhesion Molecule | Protein Coding | P32004 | 60 | GC0XM153864 | 0.705009937286377 | https://www.genecards.org/cgi-bin/carddisp.pl?gene=L1CAM |
| OTC | Ornithine Transcarbamylase | Protein Coding | P00480 | 60 | GC0XP038345 | 0.705009937286377 | https://www.genecards.org/cgi-bin/carddisp.pl?gene=OTC |
| PDHA1 | Pyruvate Dehydrogenase E1 Subunit Alpha 1 | Protein Coding | P08559 | 60 | GC0XP019343 | 0.705009937286377 | https://www.genecards.org/cgi-bin/carddisp.pl?gene=PDHA1 |
| PPT1 | Palmitoyl-Protein Thioesterase 1 | Protein Coding | P50897 | 60 | GC01M040273 | 0.705009937286377 | https://www.genecards.org/cgi-bin/carddisp.pl?gene=PPT1 |
| SIN3A | SIN3 Transcription Regulator Family Member A | Protein Coding | Q96ST3 | 60 | GC15M075369 | 0.705009937286377 | https://www.genecards.org/cgi-bin/carddisp.pl?gene=SIN3A |
| SYNGAP1 | Synaptic Ras GTPase Activating Protein 1 | Protein Coding | Q96PV0 | 60 | GC06P181961 | 0.705009937286377 | https://www.genecards.org/cgi-bin/carddisp.pl?gene=SYNGAP1 |
| TGFBR3 | Transforming Growth Factor Beta Receptor 3 | Protein Coding | Q03167 | 60 | GC01M091680 | 0.705009937286377 | https://www.genecards.org/cgi-bin/carddisp.pl?gene=TGFBR3 |
| VRK1 | VRK Serine/Threonine Kinase 1 | Protein Coding | Q99986 | 60 | GC14P096797 | 0.705009937286377 | https://www.genecards.org/cgi-bin/carddisp.pl?gene=VRK1 |
| XPO1 | Exportin 1 | Protein Coding | O14980 | 60 | GC02M061445 | 0.705009937286377 | https://www.genecards.org/cgi-bin/carddisp.pl?gene=XPO1 |
| ECHS1 | Enoyl-CoA Hydratase, Short Chain 1 | Protein Coding | P30084 | 59 | GC10M133362 | 0.705009937286377 | https://www.genecards.org/cgi-bin/carddisp.pl?gene=ECHS1 |
| HCFC1 | Host Cell Factor C1 | Protein Coding | P51610 | 59 | GC0XM153947 | 0.705009937286377 | https://www.genecards.org/cgi-bin/carddisp.pl?gene=HCFC1 |
| KDM5C | Lysine Demethylase 5C | Protein Coding | P41229 | 59 | GC0XM053176 | 0.705009937286377 | https://www.genecards.org/cgi-bin/carddisp.pl?gene=KDM5C |
| PRPS1 | Phosphoribosyl Pyrophosphate Synthetase 1 | Protein Coding | P60891 | 59 | GC0XP107628 | 0.705009937286377 | https://www.genecards.org/cgi-bin/carddisp.pl?gene=PRPS1 |
| CHD4 | Chromodomain Helicase DNA Binding Protein 4 | Protein Coding | Q14839 | 58 | GC12M006570 | 0.705009937286377 | https://www.genecards.org/cgi-bin/carddisp.pl?gene=CHD4 |
| CNTNAP2 | Contactin Associated Protein 2 | Protein Coding | Q9UHC6 | 58 | GC07P146116 | 0.705009937286377 | https://www.genecards.org/cgi-bin/carddisp.pl?gene=CNTNAP2 |
| EHMT1 | Euchromatic Histone Lysine Methyltransferase 1 | Protein Coding | Q9H9B1 | 58 | GC09P137618 | 0.705009937286377 | https://www.genecards.org/cgi-bin/carddisp.pl?gene=EHMT1 |
| GDI1 | GDP Dissociation Inhibitor 1 | Protein Coding | P31150 | 58 | GC0XP154436 | 0.705009937286377 | https://www.genecards.org/cgi-bin/carddisp.pl?gene=GDI1 |
| GLRA2 | Glycine Receptor Alpha 2 | Protein Coding | P23416 | 58 | GC0XP014448 | 0.705009937286377 | https://www.genecards.org/cgi-bin/carddisp.pl?gene=GLRA2 |
| LAMA1 | Laminin Subunit Alpha 1 | Protein Coding | P25391 | 58 | GC18M006941 | 0.705009937286377 | https://www.genecards.org/cgi-bin/carddisp.pl?gene=LAMA1 |
| NLGN1 | Neuroligin 1 | Protein Coding | Q8N2Q7 | 58 | GC03P173399 | 0.705009937286377 | https://www.genecards.org/cgi-bin/carddisp.pl?gene=NLGN1 |
| RFC1 | Replication Factor C Subunit 1 | Protein Coding | P35251 | 58 | GC04M039291 | 0.705009937286377 | https://www.genecards.org/cgi-bin/carddisp.pl?gene=RFC1 |
| SLC9A6 | Solute Carrier Family 9 Member A6 | Protein Coding | Q92581 | 58 | GC0XP135985 | 0.705009937286377 | https://www.genecards.org/cgi-bin/carddisp.pl?gene=SLC9A6 |
| SOX2 | SRY-Box Transcription Factor 2 | Protein Coding | P48431 | 58 | GC03P181711 | 0.705009937286377 | https://www.genecards.org/cgi-bin/carddisp.pl?gene=SOX2 |
| DLX5 | Distal-Less Homeobox 5 | Protein Coding | P56178 | 57 | GC07M097020 | 0.705009937286377 | https://www.genecards.org/cgi-bin/carddisp.pl?gene=DLX5 |
| H3-3A | H3.3 Histone A | Protein Coding | P84243 | 57 | GC01P226062 | 0.705009937286377 | https://www.genecards.org/cgi-bin/carddisp.pl?gene=H3-3A |
| MAP2 | Microtubule Associated Protein 2 | Protein Coding | P11137 | 57 | GC02P209424 | 0.705009937286377 | https://www.genecards.org/cgi-bin/carddisp.pl?gene=MAP2 |
| NAA10 | N-Alpha-Acetyltransferase 10, NatA Catalytic Subunit | Protein Coding | P41227 | 57 | GC0XM153929 | 0.705009937286377 | https://www.genecards.org/cgi-bin/carddisp.pl?gene=NAA10 |
| NLGN3 | Neuroligin 3 | Protein Coding | Q9NZ94 | 57 | GC0XP071144 | 0.705009937286377 | https://www.genecards.org/cgi-bin/carddisp.pl?gene=NLGN3 |
| SPG7 | SPG7 Matrix AAA Peptidase Subunit, Paraplegin | Protein Coding | Q9UQ90 | 57 | GC16P122938 | 0.705009937286377 | https://www.genecards.org/cgi-bin/carddisp.pl?gene=SPG7 |
| SYN1 | Synapsin I | Protein Coding | P17600 | 57 | GC0XM047571 | 0.705009937286377 | https://www.genecards.org/cgi-bin/carddisp.pl?gene=SYN1 |
| ANK3 | Ankyrin 3 | Protein Coding | Q12955 | 56 | GC10M060026 | 0.705009937286377 | https://www.genecards.org/cgi-bin/carddisp.pl?gene=ANK3 |
| CHD3 | Chromodomain Helicase DNA Binding Protein 3 | Protein Coding | Q12873 | 56 | GC17P153769 | 0.705009937286377 | https://www.genecards.org/cgi-bin/carddisp.pl?gene=CHD3 |
| CHD8 | Chromodomain Helicase DNA Binding Protein 8 | Protein Coding | Q9HCK8 | 56 | GC14M021385 | 0.705009937286377 | https://www.genecards.org/cgi-bin/carddisp.pl?gene=CHD8 |
| COX10 | Cytochrome C Oxidase Assembly Factor Heme A:Farnesyltransferase COX10 | Protein Coding | Q12887 | 56 | GC17P014069 | 0.705009937286377 | https://www.genecards.org/cgi-bin/carddisp.pl?gene=COX10 |
| FUT8 | Fucosyltransferase 8 | Protein Coding | Q9BYC5 | 56 | GC14P065356 | 0.705009937286377 | https://www.genecards.org/cgi-bin/carddisp.pl?gene=FUT8 |
| H2AX | H2A.X Variant Histone | Protein Coding | P16104 | 56 | GC11M140653 | 0.705009937286377 | https://www.genecards.org/cgi-bin/carddisp.pl?gene=H2AX |
| RAN | RAN, Member RAS Oncogene Family | Protein Coding | P62826 | 56 | GC12P139078 | 0.705009937286377 | https://www.genecards.org/cgi-bin/carddisp.pl?gene=RAN |
| SLC32A1 | Solute Carrier Family 32 Member 1 | Protein Coding | Q9H598 | 56 | GC20P038724 | 0.705009937286377 | https://www.genecards.org/cgi-bin/carddisp.pl?gene=SLC32A1 |
| ARHGAP4 | Rho GTPase Activating Protein 4 | Protein Coding | P98171 | 55 | GC0XM153907 | 0.705009937286377 | https://www.genecards.org/cgi-bin/carddisp.pl?gene=ARHGAP4 |
| DISC1 | DISC1 Scaffold Protein | Protein Coding | Q9NRI5 | 55 | GC01P231626 | 0.705009937286377 | https://www.genecards.org/cgi-bin/carddisp.pl?gene=DISC1 |
| HCCS | Holocytochrome C Synthase | Protein Coding | P53701 | 55 | GC0XP011111 | 0.705009937286377 | https://www.genecards.org/cgi-bin/carddisp.pl?gene=HCCS |
| MSH3 | MutS Homolog 3 | Protein Coding | P20585 | 55 | GC05P080654 | 0.705009937286377 | https://www.genecards.org/cgi-bin/carddisp.pl?gene=MSH3 |
| NLGN4X | Neuroligin 4 X-Linked | Protein Coding | Q8N0W4 | 55 | GC0XM005840 | 0.705009937286377 | https://www.genecards.org/cgi-bin/carddisp.pl?gene=NLGN4X |
| PCDH19 | Protocadherin 19 | Protein Coding | Q8TAB3 | 55 | GC0XM100291 | 0.705009937286377 | https://www.genecards.org/cgi-bin/carddisp.pl?gene=PCDH19 |
| RBBP4 | RB Binding Protein 4, Chromatin Remodeling Factor | Protein Coding | Q09028 | 55 | GC01P032651 | 0.705009937286377 | https://www.genecards.org/cgi-bin/carddisp.pl?gene=RBBP4 |
| REST | RE1 Silencing Transcription Factor | Protein Coding | Q13127 | 55 | GC04P056907 | 0.705009937286377 | https://www.genecards.org/cgi-bin/carddisp.pl?gene=REST |
| SOX3 | SRY-Box Transcription Factor 3 | Protein Coding | P41225 | 55 | GC0XM140502 | 0.705009937286377 | https://www.genecards.org/cgi-bin/carddisp.pl?gene=SOX3 |
| SUV39H1 | SUV39H1 Histone Lysine Methyltransferase | Protein Coding | O43463 | 55 | GC0XP060888 | 0.705009937286377 | https://www.genecards.org/cgi-bin/carddisp.pl?gene=SUV39H1 |
| TWIST2 | Twist Family BHLH Transcription Factor 2 | Protein Coding | Q8WVJ9 | 55 | GC02P238848 | 0.705009937286377 | https://www.genecards.org/cgi-bin/carddisp.pl?gene=TWIST2 |
| CBX1 | Chromobox 1 | Protein Coding | P83916 | 54 | GC17M048070 | 0.705009937286377 | https://www.genecards.org/cgi-bin/carddisp.pl?gene=CBX1 |
| CBX3 | Chromobox 3 | Protein Coding | Q13185 | 54 | GC07P026201 | 0.705009937286377 | https://www.genecards.org/cgi-bin/carddisp.pl?gene=CBX3 |
| FUT6 | Fucosyltransferase 6 | Protein Coding | P51993 | 54 | GC19M005830 | 0.705009937286377 | https://www.genecards.org/cgi-bin/carddisp.pl?gene=FUT6 |
| GLUD2 | Glutamate Dehydrogenase 2 | Protein Coding | P49448 | 54 | GC0XP121047 | 0.705009937286377 | https://www.genecards.org/cgi-bin/carddisp.pl?gene=GLUD2 |
| GRID1 | Glutamate Ionotropic Receptor Delta Type Subunit 1 | Protein Coding | Q9ULK0 | 54 | GC10M085599 | 0.705009937286377 | https://www.genecards.org/cgi-bin/carddisp.pl?gene=GRID1 |
| MBD1 | Methyl-CpG Binding Domain Protein 1 | Protein Coding | Q9UIS9 | 54 | GC18M050266 | 0.705009937286377 | https://www.genecards.org/cgi-bin/carddisp.pl?gene=MBD1 |
| NLGN2 | Neuroligin 2 | Protein Coding | Q8NFZ4 | 54 | GC17P153744 | 0.705009937286377 | https://www.genecards.org/cgi-bin/carddisp.pl?gene=NLGN2 |
| PLIN3 | Perilipin 3 | Protein Coding | O60664 | 54 | GC19M107908 | 0.705009937286377 | https://www.genecards.org/cgi-bin/carddisp.pl?gene=PLIN3 |
| SLC17A7 | Solute Carrier Family 17 Member 7 | Protein Coding | Q9P2U7 | 54 | GC19M049429 | 0.705009937286377 | https://www.genecards.org/cgi-bin/carddisp.pl?gene=SLC17A7 |
| SLC25A24 | Solute Carrier Family 25 Member 24 | Protein Coding | Q6NUK1 | 54 | GC01M108134 | 0.705009937286377 | https://www.genecards.org/cgi-bin/carddisp.pl?gene=SLC25A24 |
| SLC37A4 | Solute Carrier Family 37 Member 4 | Protein Coding | O43826 | 54 | GC11M140642 | 0.705009937286377 | https://www.genecards.org/cgi-bin/carddisp.pl?gene=SLC37A4 |
| TBR1 | T-Box Brain Transcription Factor 1 | Protein Coding | Q16650 | 54 | GC02P161416 | 0.705009937286377 | https://www.genecards.org/cgi-bin/carddisp.pl?gene=TBR1 |
| VRK2 | VRK Serine/Threonine Kinase 2 | Protein Coding | Q86Y07 | 54 | GC02P057907 | 0.705009937286377 | https://www.genecards.org/cgi-bin/carddisp.pl?gene=VRK2 |
| AUTS2 | Activator Of Transcription And Developmental Regulator AUTS2 | Protein Coding | Q8WXX7 | 53 | GC07P069598 | 0.705009937286377 | https://www.genecards.org/cgi-bin/carddisp.pl?gene=AUTS2 |
| CBX5 | Chromobox 5 | Protein Coding | P45973 | 53 | GC12M054230 | 0.705009937286377 | https://www.genecards.org/cgi-bin/carddisp.pl?gene=CBX5 |
| MTA3 | Metastasis Associated 1 Family Member 3 | Protein Coding | Q9BTC8 | 53 | GC02P042494 | 0.705009937286377 | https://www.genecards.org/cgi-bin/carddisp.pl?gene=MTA3 |
| NRXN2 | Neurexin 2 | Protein Coding | Q9P2S2 | 53 | GC11M139728 | 0.705009937286377 | https://www.genecards.org/cgi-bin/carddisp.pl?gene=NRXN2 |
| SHANK2 | SH3 And Multiple Ankyrin Repeat Domains 2 | Protein Coding | Q9UPX8 | 53 | GC11M070467 | 0.705009937286377 | https://www.genecards.org/cgi-bin/carddisp.pl?gene=SHANK2 |
| UBA7 | Ubiquitin Like Modifier Activating Enzyme 7 | Protein Coding | P41226 | 53 | GC03M049805 | 0.705009937286377 | https://www.genecards.org/cgi-bin/carddisp.pl?gene=UBA7 |
| ARHGAP32 | Rho GTPase Activating Protein 32 | Protein Coding | A7KAX9 | 52 | GC11M128965 | 0.705009937286377 | https://www.genecards.org/cgi-bin/carddisp.pl?gene=ARHGAP32 |
| CXXC1 | CXXC Finger Protein 1 | Protein Coding | Q9P0U4 | 52 | GC18M050282 | 0.705009937286377 | https://www.genecards.org/cgi-bin/carddisp.pl?gene=CXXC1 |
| ICMT | Isoprenylcysteine Carboxyl Methyltransferase | Protein Coding | O60725 | 52 | GC01M022284 | 0.705009937286377 | https://www.genecards.org/cgi-bin/carddisp.pl?gene=ICMT |
| LEMD3 | LEM Domain Containing 3 | Protein Coding | Q9Y2U8 | 52 | GC12P065169 | 0.705009937286377 | https://www.genecards.org/cgi-bin/carddisp.pl?gene=LEMD3 |
| MTX2 | Metaxin 2 | Protein Coding | O75431 | 52 | GC02P176269 | 0.705009937286377 | https://www.genecards.org/cgi-bin/carddisp.pl?gene=MTX2 |
| NTNG2 | Netrin G2 | Protein Coding | Q96CW9 | 52 | GC09P132161 | 0.705009937286377 | https://www.genecards.org/cgi-bin/carddisp.pl?gene=NTNG2 |
| PCDH10 | Protocadherin 10 | Protein Coding | Q9P2E7 | 52 | GC04P133149 | 0.705009937286377 | https://www.genecards.org/cgi-bin/carddisp.pl?gene=PCDH10 |
| PVALB | Parvalbumin | Protein Coding | P20472 | 52 | GC22M036800 | 0.705009937286377 | https://www.genecards.org/cgi-bin/carddisp.pl?gene=PVALB |
| RBBP7 | RB Binding Protein 7, Chromatin Remodeling Factor | Protein Coding | Q16576 | 52 | GC0XM016839 | 0.705009937286377 | https://www.genecards.org/cgi-bin/carddisp.pl?gene=RBBP7 |
| REEP1 | Receptor Accessory Protein 1 | Protein Coding | Q9H902 | 52 | GC02M086213 | 0.705009937286377 | https://www.genecards.org/cgi-bin/carddisp.pl?gene=REEP1 |
| RENBP | Renin Binding Protein | Protein Coding | P51606 | 52 | GC0XM153935 | 0.705009937286377 | https://www.genecards.org/cgi-bin/carddisp.pl?gene=RENBP |
| RS1 | Retinoschisin 1 | Protein Coding | O15537 | 52 | GC0XM018639 | 0.705009937286377 | https://www.genecards.org/cgi-bin/carddisp.pl?gene=RS1 |
| SHANK3 | SH3 And Multiple Ankyrin Repeat Domains 3 | Protein Coding | Q9BYB0 | 52 | GC22P092484 | 0.705009937286377 | https://www.genecards.org/cgi-bin/carddisp.pl?gene=SHANK3 |
| SYNE2 | Spectrin Repeat Containing Nuclear Envelope Protein 2 | Protein Coding | Q8WXH0 | 52 | GC14P063761 | 0.705009937286377 | https://www.genecards.org/cgi-bin/carddisp.pl?gene=SYNE2 |
| WDR45 | WD Repeat Domain 45 | Protein Coding | Q9Y484 | 52 | GC0XM049074 | 0.705009937286377 | https://www.genecards.org/cgi-bin/carddisp.pl?gene=WDR45 |
| XRCC2 | X-Ray Repair Cross Complementing 2 | Protein Coding | O43543 | 52 | GC07M152644 | 0.705009937286377 | https://www.genecards.org/cgi-bin/carddisp.pl?gene=XRCC2 |
| ANKLE2 | Ankyrin Repeat And LEM Domain Containing 2 | Protein Coding | Q86XL3 | 51 | GC12M132725 | 0.705009937286377 | https://www.genecards.org/cgi-bin/carddisp.pl?gene=ANKLE2 |
| DLX6 | Distal-Less Homeobox 6 | Protein Coding | P56179 | 51 | GC07P097005 | 0.705009937286377 | https://www.genecards.org/cgi-bin/carddisp.pl?gene=DLX6 |
| H3-4 | H3.4 Histone, Cluster Member | Protein Coding | Q16695 | 51 | GC01M230599 | 0.705009937286377 | https://www.genecards.org/cgi-bin/carddisp.pl?gene=H3-4 |
| LRRC4C | Leucine Rich Repeat Containing 4C | Protein Coding | Q9HCJ2 | 51 | GC11M040119 | 0.705009937286377 | https://www.genecards.org/cgi-bin/carddisp.pl?gene=LRRC4C |
| MCTP2 | Multiple C2 And Transmembrane Domain Containing 2 | Protein Coding | Q6DN12 | 51 | GC15P199104 | 0.705009937286377 | https://www.genecards.org/cgi-bin/carddisp.pl?gene=MCTP2 |
| PLPP3 | Phospholipid Phosphatase 3 | Protein Coding | O14495 | 51 | GC01M056495 | 0.705009937286377 | https://www.genecards.org/cgi-bin/carddisp.pl?gene=PLPP3 |
| SHANK1 | SH3 And Multiple Ankyrin Repeat Domains 1 | Protein Coding | Q9Y566 | 51 | GC19M050659 | 0.705009937286377 | https://www.genecards.org/cgi-bin/carddisp.pl?gene=SHANK1 |
| SUN2 | Sad1 And UNC84 Domain Containing 2 | Protein Coding | Q9UH99 | 51 | GC22M086139 | 0.705009937286377 | https://www.genecards.org/cgi-bin/carddisp.pl?gene=SUN2 |
| FXYD1 | FXYD Domain Containing Ion Transport Regulator 1 | Protein Coding | O00168 | 50 | GC19P152383 | 0.705009937286377 | https://www.genecards.org/cgi-bin/carddisp.pl?gene=FXYD1 |
| H3C1 | H3 Clustered Histone 1 | Protein Coding | P68431 | 50 | GC06P181698 | 0.705009937286377 | https://www.genecards.org/cgi-bin/carddisp.pl?gene=H3C1 |
| LEMD2 | LEM Domain Nuclear Envelope Protein 2 | Protein Coding | Q8NC56 | 50 | GC06M106603 | 0.705009937286377 | https://www.genecards.org/cgi-bin/carddisp.pl?gene=LEMD2 |
| MBD5 | Methyl-CpG Binding Domain Protein 5 | Protein Coding | Q9P267 | 50 | GC02P154649 | 0.705009937286377 | https://www.genecards.org/cgi-bin/carddisp.pl?gene=MBD5 |
| NAT10 | N-Acetyltransferase 10 | Protein Coding | Q9H0A0 | 50 | GC11P034105 | 0.705009937286377 | https://www.genecards.org/cgi-bin/carddisp.pl?gene=NAT10 |
| PLPP2 | Phospholipid Phosphatase 2 | Protein Coding | O43688 | 50 | GC19M107685 | 0.705009937286377 | https://www.genecards.org/cgi-bin/carddisp.pl?gene=PLPP2 |
| RBFOX3 | RNA Binding Fox-1 Homolog 3 | Protein Coding | A6NFN3 | 50 | GC17M079089 | 0.705009937286377 | https://www.genecards.org/cgi-bin/carddisp.pl?gene=RBFOX3 |
| SIM2 | SIM BHLH Transcription Factor 2 | Protein Coding | Q14190 | 50 | GC21P036699 | 0.705009937286377 | https://www.genecards.org/cgi-bin/carddisp.pl?gene=SIM2 |
| TKTL1 | Transketolase Like 1 | Protein Coding | P51854 | 50 | GC0XP154295 | 0.705009937286377 | https://www.genecards.org/cgi-bin/carddisp.pl?gene=TKTL1 |
| GPM6B | Glycoprotein M6B | Protein Coding | Q13491 | 49 | GC0XM016318 | 0.705009937286377 | https://www.genecards.org/cgi-bin/carddisp.pl?gene=GPM6B |
| NLGN4Y | Neuroligin 4 Y-Linked | Protein Coding | Q8NFZ3 | 49 | GC0YP014522 | 0.705009937286377 | https://www.genecards.org/cgi-bin/carddisp.pl?gene=NLGN4Y |
| PIGW | Phosphatidylinositol Glycan Anchor Biosynthesis Class W | Protein Coding | Q7Z7B1 | 49 | GC17P036534 | 0.705009937286377 | https://www.genecards.org/cgi-bin/carddisp.pl?gene=PIGW |
| TXNDC9 | Thioredoxin Domain Containing 9 | Protein Coding | O14530 | 49 | GC02M102363 | 0.705009937286377 | https://www.genecards.org/cgi-bin/carddisp.pl?gene=TXNDC9 |
| NOSIP | Nitric Oxide Synthase Interacting Protein | Protein Coding | Q9Y314 | 48 | GC19M049555 | 0.705009937286377 | https://www.genecards.org/cgi-bin/carddisp.pl?gene=NOSIP |
| TNN | Tenascin N | Protein Coding | Q9UQP3 | 48 | GC01P175067 | 0.705009937286377 | https://www.genecards.org/cgi-bin/carddisp.pl?gene=TNN |
| GNG3 | G Protein Subunit Gamma 3 | Protein Coding | P63215 | 47 | GC11P107534 | 0.705009937286377 | https://www.genecards.org/cgi-bin/carddisp.pl?gene=GNG3 |
| OPN1LW | Opsin 1, Long Wave Sensitive | Protein Coding | P04000 | 47 | GC0XP154144 | 0.705009937286377 | https://www.genecards.org/cgi-bin/carddisp.pl?gene=OPN1LW |
| OPN1MW | Opsin 1, Medium Wave Sensitive | Protein Coding | P04001 | 47 | GC0XP154182 | 0.705009937286377 | https://www.genecards.org/cgi-bin/carddisp.pl?gene=OPN1MW |
| PRPF40A | Pre-MRNA Processing Factor 40 Homolog A | Protein Coding | O75400 | 47 | GC02M152651 | 0.705009937286377 | https://www.genecards.org/cgi-bin/carddisp.pl?gene=PRPF40A |
| TAMM41 | TAM41 Mitochondrial Translocator Assembly And Maintenance Homolog | Protein Coding | Q96BW9 | 47 | GC03M011721 | 0.705009937286377 | https://www.genecards.org/cgi-bin/carddisp.pl?gene=TAMM41 |
| TRMT10A | TRNA Methyltransferase 10A | Protein Coding | Q8TBZ6 | 47 | GC04M099546 | 0.705009937286377 | https://www.genecards.org/cgi-bin/carddisp.pl?gene=TRMT10A |
| UBL4A | Ubiquitin Like 4A | Protein Coding | P11441 | 47 | GC0XM154483 | 0.705009937286377 | https://www.genecards.org/cgi-bin/carddisp.pl?gene=UBL4A |
| ZNHIT3 | Zinc Finger HIT-Type Containing 3 | Protein Coding | Q15649 | 47 | GC17P036486 | 0.705009937286377 | https://www.genecards.org/cgi-bin/carddisp.pl?gene=ZNHIT3 |
| GPR179 | G Protein-Coupled Receptor 179 | Protein Coding | Q6PRD1 | 46 | GC17M097047 | 0.705009937286377 | https://www.genecards.org/cgi-bin/carddisp.pl?gene=GPR179 |
| H3C2 | H3 Clustered Histone 2 | Protein Coding | P68431 | 45 | GC06M106106 | 0.705009937286377 | https://www.genecards.org/cgi-bin/carddisp.pl?gene=H3C2 |
| ARHGAP21 | Rho GTPase Activating Protein 21 | Protein Coding | Q5T5U3 | 44 | GC10M024583 | 0.705009937286377 | https://www.genecards.org/cgi-bin/carddisp.pl?gene=ARHGAP21 |
| BANF2 | BANF Family Member 2 | Protein Coding | Q9H503 | 44 | GC20P017706 | 0.705009937286377 | https://www.genecards.org/cgi-bin/carddisp.pl?gene=BANF2 |
| H3-5 | H3.5 Histone | Protein Coding | Q6NXT2 | 44 | GC12M036090 | 0.705009937286377 | https://www.genecards.org/cgi-bin/carddisp.pl?gene=H3-5 |
| H3C3 | H3 Clustered Histone 3 | Protein Coding | P68431 | 44 | GC06P181701 | 0.705009937286377 | https://www.genecards.org/cgi-bin/carddisp.pl?gene=H3C3 |
| LGALSL | Galectin Like | Protein Coding | Q3ZCW2 | 44 | GC02P064453 | 0.705009937286377 | https://www.genecards.org/cgi-bin/carddisp.pl?gene=LGALSL |
| MOGAT1 | Monoacylglycerol O-Acyltransferase 1 | Protein Coding | Q96PD6 | 44 | GC02P222671 | 0.705009937286377 | https://www.genecards.org/cgi-bin/carddisp.pl?gene=MOGAT1 |
| H3C4 | H3 Clustered Histone 4 | Protein Coding | P68431 | 43 | GC06M109201 | 0.705009937286377 | https://www.genecards.org/cgi-bin/carddisp.pl?gene=H3C4 |
| H3C6 | H3 Clustered Histone 6 | Protein Coding | P68431 | 43 | GC06P184730 | 0.705009937286377 | https://www.genecards.org/cgi-bin/carddisp.pl?gene=H3C6 |
| H3C10 | H3 Clustered Histone 10 | Protein Coding | P68431 | 42 | GC06P181761 | 0.705009937286377 | https://www.genecards.org/cgi-bin/carddisp.pl?gene=H3C10 |
| H3C11 | H3 Clustered Histone 11 | Protein Coding | P68431 | 42 | GC06M109199 | 0.705009937286377 | https://www.genecards.org/cgi-bin/carddisp.pl?gene=H3C11 |
| H3C14 | H3 Clustered Histone 14 | Protein Coding | Q71DI3 | 42 | GC01M167225 | 0.705009937286377 | https://www.genecards.org/cgi-bin/carddisp.pl?gene=H3C14 |
| H3C7 | H3 Clustered Histone 7 | Protein Coding | P68431 | 42 | GC06M109202 | 0.705009937286377 | https://www.genecards.org/cgi-bin/carddisp.pl?gene=H3C7 |
| H3C8 | H3 Clustered Histone 8 | Protein Coding | P68431 | 42 | GC06M109203 | 0.705009937286377 | https://www.genecards.org/cgi-bin/carddisp.pl?gene=H3C8 |
| MED16 | Mediator Complex Subunit 16 | Protein Coding | Q9Y2X0 | 42 | GC19M107728 | 0.705009937286377 | https://www.genecards.org/cgi-bin/carddisp.pl?gene=MED16 |
| CIMAP2 | Ciliary Microtubule Associated Protein 2 | Protein Coding | Q3ZCV2 | 41 | GC01P078560 | 0.705009937286377 | https://www.genecards.org/cgi-bin/carddisp.pl?gene=CIMAP2 |
| TEX28 | Testis Expressed 28 | Protein Coding | O15482 | 41 | GC0XM154271 | 0.705009937286377 | https://www.genecards.org/cgi-bin/carddisp.pl?gene=TEX28 |
| LDAF1 | Lipid Droplet Assembly Factor 1 | Protein Coding | Q96B96 | 40 | GC16P120632 | 0.705009937286377 | https://www.genecards.org/cgi-bin/carddisp.pl?gene=LDAF1 |
| H3C15 | H3 Clustered Histone 15 | Protein Coding | Q71DI3 | 38 | GC01P176450 | 0.705009937286377 | https://www.genecards.org/cgi-bin/carddisp.pl?gene=H3C15 |
| TMEM187 | Transmembrane Protein 187 | Protein Coding | Q14656 | 38 | GC0XP153972 | 0.705009937286377 | https://www.genecards.org/cgi-bin/carddisp.pl?gene=TMEM187 |
| OPN1MW2 | Opsin 1, Medium Wave Sensitive 2 | Protein Coding | P0DN77 | 33 | GC0XP154219 | 0.705009937286377 | https://www.genecards.org/cgi-bin/carddisp.pl?gene=OPN1MW2 |
| H3-7 | H3.7 Histone (Putative) | Protein Coding | Q5TEC6 | 32 | GC01M166852 | 0.705009937286377 | https://www.genecards.org/cgi-bin/carddisp.pl?gene=H3-7 |
| MIR24-1 | MicroRNA 24-1 | RNA Gene |  | 27 | GC09P095086 | 0.705009937286377 | https://www.genecards.org/cgi-bin/carddisp.pl?gene=MIR24-1 |
| MIR19B1 | MicroRNA 19b-1 | RNA Gene |  | 26 | GC13P091745 | 0.705009937286377 | https://www.genecards.org/cgi-bin/carddisp.pl?gene=MIR19B1 |
| OPN1MW3 | Opsin 1, Medium Wave Sensitive 3 | Protein Coding | P0DN78 | 26 | GC0XP155165 | 0.705009937286377 | https://www.genecards.org/cgi-bin/carddisp.pl?gene=OPN1MW3 |
| SNHG14 | Small Nucleolar RNA Host Gene 14 | RNA Gene |  | 25 | GC15P192315 | 0.705009937286377 | https://www.genecards.org/cgi-bin/carddisp.pl?gene=SNHG14 |
| HCFC1-AS1 | HCFC1 Antisense RNA 1 | RNA Gene |  | 22 | GC0XP153967 | 0.705009937286377 | https://www.genecards.org/cgi-bin/carddisp.pl?gene=HCFC1-AS1 |
| L1CAM-AS1 | L1CAM Antisense RNA 1 | RNA Gene |  | 21 | GC0XP155129 | 0.705009937286377 | https://www.genecards.org/cgi-bin/carddisp.pl?gene=L1CAM-AS1 |
| MIR3202-2 | MicroRNA 3202-2 | RNA Gene |  | 21 | GC0XM153981 | 0.705009937286377 | https://www.genecards.org/cgi-bin/carddisp.pl?gene=MIR3202-2 |
| MIR718 | MicroRNA 718 | RNA Gene |  | 21 | GC0XM154019 | 0.705009937286377 | https://www.genecards.org/cgi-bin/carddisp.pl?gene=MIR718 |
| MIR3202-1 | MicroRNA 3202-1 | RNA Gene |  | 20 | GC0XP153981 | 0.705009937286377 | https://www.genecards.org/cgi-bin/carddisp.pl?gene=MIR3202-1 |
| TRD-GTC9-1 | TRNA-Asp (Anticodon GTC) 9-1 | RNA Gene |  | 14 | GC01P177022 | 0.705009937286377 | https://www.genecards.org/cgi-bin/carddisp.pl?gene=TRD-GTC9-1 |
| LOC107988032 | Xq28 Proximal FLNA-EMD Recombination Region | Functional Element |  | 10 | GC0XP154335 | 0.705009937286377 | https://www.genecards.org/cgi-bin/carddisp.pl?gene=LOC107988032 |
| LOC130068840 | ATAC-STARR-Seq Lymphoblastoid Silent Region 21075 | Functional Element |  | 9 | GC0XP155145 | 0.705009937286377 | https://www.genecards.org/cgi-bin/carddisp.pl?gene=LOC130068840 |
| OPSIN-LCR | Opsin Locus Control Region | Functional Element |  | 9 | GC0XP154137 | 0.705009937286377 | https://www.genecards.org/cgi-bin/carddisp.pl?gene=OPSIN-LCR |
| LOC111365170 | NFE2L2 Motif-Containing MPRA Enhancer 190/191 | Functional Element |  | 8 | GC0XP154002 | 0.705009937286377 | https://www.genecards.org/cgi-bin/carddisp.pl?gene=LOC111365170 |
| LOC116309162 | CRISPRi-Validated Cis-Regulatory Element ChrX.2695 | Functional Element |  | 8 | GC0XP153985 | 0.705009937286377 | https://www.genecards.org/cgi-bin/carddisp.pl?gene=LOC116309162 |
| LOC125467793 | Sharpr-MPRA Regulatory Region 3572 | Functional Element |  | 8 | GC0XP154136 | 0.705009937286377 | https://www.genecards.org/cgi-bin/carddisp.pl?gene=LOC125467793 |
| LOC129929050 | ATAC-STARR-Seq Lymphoblastoid Active Region 30056 | Functional Element |  | 8 | GC0XP154347 | 0.705009937286377 | https://www.genecards.org/cgi-bin/carddisp.pl?gene=LOC129929050 |
| LOC129929051 | ATAC-STARR-Seq Lymphoblastoid Active Region 30057 | Functional Element |  | 8 | GC0XP155170 | 0.705009937286377 | https://www.genecards.org/cgi-bin/carddisp.pl?gene=LOC129929051 |
| LOC130068837 | ATAC-STARR-Seq Lymphoblastoid Active Region 30042 | Functional Element |  | 8 | GC0XP155142 | 0.705009937286377 | https://www.genecards.org/cgi-bin/carddisp.pl?gene=LOC130068837 |
| LOC130068838 | ATAC-STARR-Seq Lymphoblastoid Silent Region 21073 | Functional Element |  | 8 | GC0XP155143 | 0.705009937286377 | https://www.genecards.org/cgi-bin/carddisp.pl?gene=LOC130068838 |
| LOC130068839 | ATAC-STARR-Seq Lymphoblastoid Silent Region 21074 | Functional Element |  | 8 | GC0XP155144 | 0.705009937286377 | https://www.genecards.org/cgi-bin/carddisp.pl?gene=LOC130068839 |
| LOC130068841 | ATAC-STARR-Seq Lymphoblastoid Active Region 30043 | Functional Element |  | 8 | GC0XP155247 | 0.705009937286377 | https://www.genecards.org/cgi-bin/carddisp.pl?gene=LOC130068841 |
| LOC130068842 | ATAC-STARR-Seq Lymphoblastoid Active Region 30044 | Functional Element |  | 8 | GC0XP155248 | 0.705009937286377 | https://www.genecards.org/cgi-bin/carddisp.pl?gene=LOC130068842 |
| LOC130068843 | ATAC-STARR-Seq Lymphoblastoid Active Region 30045 | Functional Element |  | 8 | GC0XP155251 | 0.705009937286377 | https://www.genecards.org/cgi-bin/carddisp.pl?gene=LOC130068843 |
| LOC130068844 | ATAC-STARR-Seq Lymphoblastoid Silent Region 21077 | Functional Element |  | 8 | GC0XP155252 | 0.705009937286377 | https://www.genecards.org/cgi-bin/carddisp.pl?gene=LOC130068844 |
| LOC130068845 | ATAC-STARR-Seq Lymphoblastoid Silent Region 21078 | Functional Element |  | 8 | GC0XP155253 | 0.705009937286377 | https://www.genecards.org/cgi-bin/carddisp.pl?gene=LOC130068845 |
| LOC130068846 | ATAC-STARR-Seq Lymphoblastoid Silent Region 21079 | Functional Element |  | 8 | GC0XP155254 | 0.705009937286377 | https://www.genecards.org/cgi-bin/carddisp.pl?gene=LOC130068846 |
| LOC130068847 | ATAC-STARR-Seq Lymphoblastoid Active Region 30047 | Functional Element |  | 8 | GC0XP155139 | 0.705009937286377 | https://www.genecards.org/cgi-bin/carddisp.pl?gene=LOC130068847 |
| LOC130068848 | ATAC-STARR-Seq Lymphoblastoid Active Region 30048 | Functional Element |  | 8 | GC0XP155141 | 0.705009937286377 | https://www.genecards.org/cgi-bin/carddisp.pl?gene=LOC130068848 |
| LOC130068849 | ATAC-STARR-Seq Lymphoblastoid Silent Region 21080 | Functional Element |  | 8 | GC0XP155156 | 0.705009937286377 | https://www.genecards.org/cgi-bin/carddisp.pl?gene=LOC130068849 |
| LOC130068850 | ATAC-STARR-Seq Lymphoblastoid Silent Region 21081 | Functional Element |  | 8 | GC0XP155157 | 0.705009937286377 | https://www.genecards.org/cgi-bin/carddisp.pl?gene=LOC130068850 |
| LOC130068851 | ATAC-STARR-Seq Lymphoblastoid Active Region 30052 | Functional Element |  | 8 | GC0XP155158 | 0.705009937286377 | https://www.genecards.org/cgi-bin/carddisp.pl?gene=LOC130068851 |
| LOC130068852 | ATAC-STARR-Seq Lymphoblastoid Active Region 30053 | Functional Element |  | 8 | GC0XP154096 | 0.705009937286377 | https://www.genecards.org/cgi-bin/carddisp.pl?gene=LOC130068852 |
| LOC130068853 | ATAC-STARR-Seq Lymphoblastoid Silent Region 21083 | Functional Element |  | 8 | GC0XP155160 | 0.705009937286377 | https://www.genecards.org/cgi-bin/carddisp.pl?gene=LOC130068853 |
| LOC130068855 | ATAC-STARR-Seq Lymphoblastoid Active Region 30055 | Functional Element |  | 8 | GC0XP154334 | 0.705009937286377 | https://www.genecards.org/cgi-bin/carddisp.pl?gene=LOC130068855 |
| ALPL | Alkaline Phosphatase, Biomineralization Associated | Protein Coding | P05186 | 63 | GC01P021508 | 0.704768061637878 | https://www.genecards.org/cgi-bin/carddisp.pl?gene=ALPL |
| IL6ST | Interleukin 6 Cytokine Family Signal Transducer | Protein Coding | P40189 | 63 | GC05M055935 | 0.704768061637878 | https://www.genecards.org/cgi-bin/carddisp.pl?gene=IL6ST |
| ESRRB | Estrogen Related Receptor Beta | Protein Coding | O95718 | 61 | GC14P076310 | 0.704768061637878 | https://www.genecards.org/cgi-bin/carddisp.pl?gene=ESRRB |
| MC1R | Melanocortin 1 Receptor | Protein Coding | Q01726 | 60 | GC16P089912 | 0.704768061637878 | https://www.genecards.org/cgi-bin/carddisp.pl?gene=MC1R |
| AKR1C3 | Aldo-Keto Reductase Family 1 Member C3 | Protein Coding | P42330 | 57 | GC10P005035 | 0.704768061637878 | https://www.genecards.org/cgi-bin/carddisp.pl?gene=AKR1C3 |
| ANKH | ANKH Inorganic Pyrophosphate Transport Regulator | Protein Coding | Q9HCJ1 | 51 | GC05M014860 | 0.704768061637878 | https://www.genecards.org/cgi-bin/carddisp.pl?gene=ANKH |
| MT-ND3 | Mitochondrially Encoded NADH:Ubiquinone Oxidoreductase Core Subunit 3 | Protein Coding | P03897 | 45 | GCMTP010061 | 0.704768061637878 | https://www.genecards.org/cgi-bin/carddisp.pl?gene=MT-ND3 |
| GARIN1B | Golgi Associated RAB2 Interactor 1B | Protein Coding | Q96KD3 | 39 | GC07P166388 | 0.704768061637878 | https://www.genecards.org/cgi-bin/carddisp.pl?gene=GARIN1B |
| SELP | Selectin P | Protein Coding | P16109 | 59 | GC01M169558 | 0.69943380355835 | https://www.genecards.org/cgi-bin/carddisp.pl?gene=SELP |
| DBH | Dopamine Beta-Hydroxylase | Protein Coding | P09172 | 64 | GC09P133636 | 0.673459053039551 | https://www.genecards.org/cgi-bin/carddisp.pl?gene=DBH |
| HRH1 | Histamine Receptor H1 | Protein Coding | P35367 | 58 | GC03P027032 | 0.673459053039551 | https://www.genecards.org/cgi-bin/carddisp.pl?gene=HRH1 |
| FGFR1 | Fibroblast Growth Factor Receptor 1 | Protein Coding | P11362 | 68 | GC08M038400 | 0.666668236255646 | https://www.genecards.org/cgi-bin/carddisp.pl?gene=FGFR1 |
| KITLG | KIT Ligand | Protein Coding | P21583 | 60 | GC12M088492 | 0.666668236255646 | https://www.genecards.org/cgi-bin/carddisp.pl?gene=KITLG |
| AGTR2 | Angiotensin II Receptor Type 2 | Protein Coding | P50052 | 57 | GC0XP116170 | 0.666668236255646 | https://www.genecards.org/cgi-bin/carddisp.pl?gene=AGTR2 |
| CX3CL1 | C-X3-C Motif Chemokine Ligand 1 | Protein Coding | P78423 | 57 | GC16P057372 | 0.666668236255646 | https://www.genecards.org/cgi-bin/carddisp.pl?gene=CX3CL1 |
| BHMT | Betaine--Homocysteine S-Methyltransferase | Protein Coding | Q93088 | 55 | GC05P079111 | 0.666668236255646 | https://www.genecards.org/cgi-bin/carddisp.pl?gene=BHMT |
| TLR10 | Toll Like Receptor 10 | Protein Coding | Q9BXR5 | 50 | GC04M038773 | 0.666668236255646 | https://www.genecards.org/cgi-bin/carddisp.pl?gene=TLR10 |
| NMU | Neuromedin U | Protein Coding | P48645 | 48 | GC04M055595 | 0.666668236255646 | https://www.genecards.org/cgi-bin/carddisp.pl?gene=NMU |
| HK1 | Hexokinase 1 | Protein Coding | P19367 | 63 | GC10P069269 | 0.657057523727417 | https://www.genecards.org/cgi-bin/carddisp.pl?gene=HK1 |
| CDKN2A | Cyclin Dependent Kinase Inhibitor 2A | Protein Coding | Q8N726 | 64 | GC09M021967 | 0.645552515983582 | https://www.genecards.org/cgi-bin/carddisp.pl?gene=CDKN2A |
| NR1H3 | Nuclear Receptor Subfamily 1 Group H Member 3 | Protein Coding | Q13133 | 59 | GC11P047248 | 0.645552515983582 | https://www.genecards.org/cgi-bin/carddisp.pl?gene=NR1H3 |
| PDX1 | Pancreatic And Duodenal Homeobox 1 | Protein Coding | P52945 | 59 | GC13P028977 | 0.645552515983582 | https://www.genecards.org/cgi-bin/carddisp.pl?gene=PDX1 |
| TNFSF10 | TNF Superfamily Member 10 | Protein Coding | P50591 | 58 | GC03M172505 | 0.645552515983582 | https://www.genecards.org/cgi-bin/carddisp.pl?gene=TNFSF10 |
| PRKAB2 | Protein Kinase AMP-Activated Non-Catalytic Subunit Beta 2 | Protein Coding | O43741 | 57 | GC01M147155 | 0.645552515983582 | https://www.genecards.org/cgi-bin/carddisp.pl?gene=PRKAB2 |
| SMAD4 | SMAD Family Member 4 | Protein Coding | Q13485 | 65 | GC18P051028 | 0.621474862098694 | https://www.genecards.org/cgi-bin/carddisp.pl?gene=SMAD4 |
| RPS6KB1 | Ribosomal Protein S6 Kinase B1 | Protein Coding | P23443 | 63 | GC17P059893 | 0.621474862098694 | https://www.genecards.org/cgi-bin/carddisp.pl?gene=RPS6KB1 |
| FBP1 | Fructose-Bisphosphatase 1 | Protein Coding | P09467 | 62 | GC09M094603 | 0.621474862098694 | https://www.genecards.org/cgi-bin/carddisp.pl?gene=FBP1 |
| FGF8 | Fibroblast Growth Factor 8 | Protein Coding | P55075 | 61 | GC10M101770 | 0.621474862098694 | https://www.genecards.org/cgi-bin/carddisp.pl?gene=FGF8 |
| CPE | Carboxypeptidase E | Protein Coding | P16870 | 60 | GC04P165361 | 0.621474862098694 | https://www.genecards.org/cgi-bin/carddisp.pl?gene=CPE |
| S100B | S100 Calcium Binding Protein B | Protein Coding | P04271 | 58 | GC21M054960 | 0.621474862098694 | https://www.genecards.org/cgi-bin/carddisp.pl?gene=S100B |
| SIX1 | SIX Homeobox 1 | Protein Coding | Q15475 | 55 | GC14M060643 | 0.621474862098694 | https://www.genecards.org/cgi-bin/carddisp.pl?gene=SIX1 |
| USF1 | Upstream Transcription Factor 1 | Protein Coding | P22415 | 55 | GC01M161039 | 0.621474862098694 | https://www.genecards.org/cgi-bin/carddisp.pl?gene=USF1 |
| PER1 | Period Circadian Regulator 1 | Protein Coding | O15534 | 54 | GC17M096232 | 0.621474862098694 | https://www.genecards.org/cgi-bin/carddisp.pl?gene=PER1 |
| PER3 | Period Circadian Regulator 3 | Protein Coding | P56645 | 54 | GC01P075169 | 0.621474862098694 | https://www.genecards.org/cgi-bin/carddisp.pl?gene=PER3 |
| AMD1 | Adenosylmethionine Decarboxylase 1 | Protein Coding | P17707 | 53 | GC06P110814 | 0.621474862098694 | https://www.genecards.org/cgi-bin/carddisp.pl?gene=AMD1 |
| STEAP4 | STEAP4 Metalloreductase | Protein Coding | Q687X5 | 53 | GC07M088597 | 0.621474862098694 | https://www.genecards.org/cgi-bin/carddisp.pl?gene=STEAP4 |
| NFKBIB | NFKB Inhibitor Beta | Protein Coding | Q15653 | 52 | GC19P038899 | 0.621474862098694 | https://www.genecards.org/cgi-bin/carddisp.pl?gene=NFKBIB |
| PEMT | Phosphatidylethanolamine N-Methyltransferase | Protein Coding | Q9UBM1 | 51 | GC17M096443 | 0.621474862098694 | https://www.genecards.org/cgi-bin/carddisp.pl?gene=PEMT |
| CTNNAL1 | Catenin Alpha Like 1 | Protein Coding | Q9UBT7 | 48 | GC09M108942 | 0.621474862098694 | https://www.genecards.org/cgi-bin/carddisp.pl?gene=CTNNAL1 |
| CRPP1 | C-Reactive Protein Pseudogene 1 | Pseudogene |  | 10 | GC01M167582 | 0.621474862098694 | https://www.genecards.org/cgi-bin/carddisp.pl?gene=CRPP1 |
| CALCR | Calcitonin Receptor | Protein Coding | P30988 | 61 | GC07M093424 | 0.61979341506958 | https://www.genecards.org/cgi-bin/carddisp.pl?gene=CALCR |
| NGFR | Nerve Growth Factor Receptor | Protein Coding | P08138 | 60 | GC17P049495 | 0.615206003189087 | https://www.genecards.org/cgi-bin/carddisp.pl?gene=NGFR |
| CLOCK | Clock Circadian Regulator | Protein Coding | O15516 | 57 | GC04M055427 | 0.615206003189087 | https://www.genecards.org/cgi-bin/carddisp.pl?gene=CLOCK |
| ISL1 | ISL LIM Homeobox 1 | Protein Coding | P61371 | 57 | GC05P051383 | 0.615206003189087 | https://www.genecards.org/cgi-bin/carddisp.pl?gene=ISL1 |
| COPA | COPI Coat Complex Subunit Alpha | Protein Coding | P53621 | 55 | GC01M160288 | 0.615206003189087 | https://www.genecards.org/cgi-bin/carddisp.pl?gene=COPA |
| STC2 | Stanniocalcin 2 | Protein Coding | O76061 | 54 | GC05M173314 | 0.615206003189087 | https://www.genecards.org/cgi-bin/carddisp.pl?gene=STC2 |
| ASIP | Agouti Signaling Protein | Protein Coding | P42127 | 51 | GC20P053193 | 0.615206003189087 | https://www.genecards.org/cgi-bin/carddisp.pl?gene=ASIP |
| CLPS | Colipase | Protein Coding | P04118 | 51 | GC06M106629 | 0.615206003189087 | https://www.genecards.org/cgi-bin/carddisp.pl?gene=CLPS |
| UTS2 | Urotensin 2 | Protein Coding | O95399 | 51 | GC01M007843 | 0.615206003189087 | https://www.genecards.org/cgi-bin/carddisp.pl?gene=UTS2 |
| IL23A | Interleukin 23 Subunit Alpha | Protein Coding | Q9NPF7 | 50 | GC12P075442 | 0.615206003189087 | https://www.genecards.org/cgi-bin/carddisp.pl?gene=IL23A |
| PM20D1 | Peptidase M20 Domain Containing 1 | Protein Coding | Q6GTS8 | 48 | GC01M205829 | 0.615206003189087 | https://www.genecards.org/cgi-bin/carddisp.pl?gene=PM20D1 |
| MIAT | Myocardial Infarction Associated Transcript | RNA Gene |  | 34 | GC22P026646 | 0.615206003189087 | https://www.genecards.org/cgi-bin/carddisp.pl?gene=MIAT |
| KCNJ6-AS1 | KCNJ6 Antisense RNA 1 | RNA Gene |  | 14 | GC21P037560 | 0.600142657756805 | https://www.genecards.org/cgi-bin/carddisp.pl?gene=KCNJ6-AS1 |
| RNASEL | Ribonuclease L | Protein Coding | Q05823 | 57 | GC01M182573 | 0.594517409801483 | https://www.genecards.org/cgi-bin/carddisp.pl?gene=RNASEL |
| CP | Ceruloplasmin | Protein Coding | P00450 | 62 | GC03M149162 | 0.581639409065247 | https://www.genecards.org/cgi-bin/carddisp.pl?gene=CP |
| COMP | Cartilage Oligomeric Matrix Protein | Protein Coding | P49747 | 60 | GC19M108410 | 0.581639409065247 | https://www.genecards.org/cgi-bin/carddisp.pl?gene=COMP |
| NPR3 | Natriuretic Peptide Receptor 3 | Protein Coding | P17342 | 57 | GC05P032689 | 0.581639409065247 | https://www.genecards.org/cgi-bin/carddisp.pl?gene=NPR3 |
| PLAUR | Plasminogen Activator, Urokinase Receptor | Protein Coding | Q03405 | 57 | GC19M043646 | 0.581639409065247 | https://www.genecards.org/cgi-bin/carddisp.pl?gene=PLAUR |
| MGP | Matrix Gla Protein | Protein Coding | P08493 | 56 | GC12M035863 | 0.581639409065247 | https://www.genecards.org/cgi-bin/carddisp.pl?gene=MGP |
| ST3GAL4 | ST3 Beta-Galactoside Alpha-2,3-Sialyltransferase 4 | Protein Coding | Q11206 | 56 | GC11P126355 | 0.581639409065247 | https://www.genecards.org/cgi-bin/carddisp.pl?gene=ST3GAL4 |
| ANOS1 | Anosmin 1 | Protein Coding | P23352 | 52 | GC0XM008528 | 0.581639409065247 | https://www.genecards.org/cgi-bin/carddisp.pl?gene=ANOS1 |
| POLR2D | RNA Polymerase II Subunit D | Protein Coding | O15514 | 52 | GC02M128781 | 0.581639409065247 | https://www.genecards.org/cgi-bin/carddisp.pl?gene=POLR2D |
| LPXN | Leupaxin | Protein Coding | O60711 | 51 | GC11M139524 | 0.581639409065247 | https://www.genecards.org/cgi-bin/carddisp.pl?gene=LPXN |
| CCL8 | C-C Motif Chemokine Ligand 8 | Protein Coding | P80075 | 50 | GC17P034319 | 0.581639409065247 | https://www.genecards.org/cgi-bin/carddisp.pl?gene=CCL8 |
| MIR320A | MicroRNA 320a | RNA Gene |  | 31 | GC08M022455 | 0.581639409065247 | https://www.genecards.org/cgi-bin/carddisp.pl?gene=MIR320A |
| KRT18 | Keratin 18 | Protein Coding | P05783 | 62 | GC12P052948 | 0.562577664852142 | https://www.genecards.org/cgi-bin/carddisp.pl?gene=KRT18 |
| TLR1 | Toll Like Receptor 1 | Protein Coding | Q15399 | 61 | GC04M038793 | 0.562577664852142 | https://www.genecards.org/cgi-bin/carddisp.pl?gene=TLR1 |
| CAST | Calpastatin | Protein Coding | P20810 | 60 | GC05P095975 | 0.562577664852142 | https://www.genecards.org/cgi-bin/carddisp.pl?gene=CAST |
| IGFL3 | IGF Like Family Member 3 | Protein Coding | Q6UXB1 | 40 | GC19M046120 | 0.562577664852142 | https://www.genecards.org/cgi-bin/carddisp.pl?gene=IGFL3 |
| PCA3 | Prostate Cancer Associated 3 | RNA Gene |  | 31 | GC09P076691 | 0.562577664852142 | https://www.genecards.org/cgi-bin/carddisp.pl?gene=PCA3 |
| RMST | Rhabdomyosarcoma 2 Associated Transcript | RNA Gene |  | 30 | GC12P096991 | 0.562577664852142 | https://www.genecards.org/cgi-bin/carddisp.pl?gene=RMST |
| MAFTRR | MAF Transcriptional Regulator RNA | RNA Gene |  | 15 | GC16M079657 | 0.562577664852142 | https://www.genecards.org/cgi-bin/carddisp.pl?gene=MAFTRR |
| HMOX1 | Heme Oxygenase 1 | Protein Coding | P09601 | 65 | GC22P035380 | 0.543539643287659 | https://www.genecards.org/cgi-bin/carddisp.pl?gene=HMOX1 |
| RPS6KA3 | Ribosomal Protein S6 Kinase A3 | Protein Coding | P51812 | 65 | GC0XM020149 | 0.543539643287659 | https://www.genecards.org/cgi-bin/carddisp.pl?gene=RPS6KA3 |
| SHH | Sonic Hedgehog Signaling Molecule | Protein Coding | Q15465 | 63 | GC07M155799 | 0.543539643287659 | https://www.genecards.org/cgi-bin/carddisp.pl?gene=SHH |
| CD40LG | CD40 Ligand | Protein Coding | P29965 | 62 | GC0XP136649 | 0.543539643287659 | https://www.genecards.org/cgi-bin/carddisp.pl?gene=CD40LG |
| FGG | Fibrinogen Gamma Chain | Protein Coding | P02679 | 61 | GC04M154604 | 0.543539643287659 | https://www.genecards.org/cgi-bin/carddisp.pl?gene=FGG |
| YAP1 | Yes1 Associated Transcriptional Regulator | Protein Coding | P46937 | 61 | GC11P102110 | 0.543539643287659 | https://www.genecards.org/cgi-bin/carddisp.pl?gene=YAP1 |
| BIRC3 | Baculoviral IAP Repeat Containing 3 | Protein Coding | Q13489 | 60 | GC11P102317 | 0.543539643287659 | https://www.genecards.org/cgi-bin/carddisp.pl?gene=BIRC3 |
| MBL2 | Mannose Binding Lectin 2 | Protein Coding | P11226 | 60 | GC10M052760 | 0.543539643287659 | https://www.genecards.org/cgi-bin/carddisp.pl?gene=MBL2 |
| ACAT2 | Acetyl-CoA Acetyltransferase 2 | Protein Coding | Q9BWD1 | 59 | GC06P183876 | 0.543539643287659 | https://www.genecards.org/cgi-bin/carddisp.pl?gene=ACAT2 |
| FOXP3 | Forkhead Box P3 | Protein Coding | Q9BZS1 | 59 | GC0XM049250 | 0.543539643287659 | https://www.genecards.org/cgi-bin/carddisp.pl?gene=FOXP3 |
| GNRHR | Gonadotropin Releasing Hormone Receptor | Protein Coding | P30968 | 59 | GC04M067737 | 0.543539643287659 | https://www.genecards.org/cgi-bin/carddisp.pl?gene=GNRHR |
| CD274 | CD274 Molecule | Protein Coding | Q9NZQ7 | 58 | GC09P005450 | 0.543539643287659 | https://www.genecards.org/cgi-bin/carddisp.pl?gene=CD274 |
| SERPINA6 | Serpin Family A Member 6 | Protein Coding | P08185 | 58 | GC14M124249 | 0.543539643287659 | https://www.genecards.org/cgi-bin/carddisp.pl?gene=SERPINA6 |
| DRD3 | Dopamine Receptor D3 | Protein Coding | P35462 | 57 | GC03M114128 | 0.543539643287659 | https://www.genecards.org/cgi-bin/carddisp.pl?gene=DRD3 |
| DUOX1 | Dual Oxidase 1 | Protein Coding | Q9NRD9 | 55 | GC15P045129 | 0.543539643287659 | https://www.genecards.org/cgi-bin/carddisp.pl?gene=DUOX1 |
| FADS1 | Fatty Acid Desaturase 1 | Protein Coding | O60427 | 55 | GC11M061799 | 0.543539643287659 | https://www.genecards.org/cgi-bin/carddisp.pl?gene=FADS1 |
| HNF1B | HNF1 Homeobox B | Protein Coding | P35680 | 55 | GC17M037686 | 0.543539643287659 | https://www.genecards.org/cgi-bin/carddisp.pl?gene=HNF1B |
| IL7 | Interleukin 7 | Protein Coding | P13232 | 55 | GC08M078689 | 0.543539643287659 | https://www.genecards.org/cgi-bin/carddisp.pl?gene=IL7 |
| PRPF8 | Pre-MRNA Processing Factor 8 | Protein Coding | Q6P2Q9 | 54 | GC17M001650 | 0.543539643287659 | https://www.genecards.org/cgi-bin/carddisp.pl?gene=PRPF8 |
| CXCL5 | C-X-C Motif Chemokine Ligand 5 | Protein Coding | P42830 | 52 | GC04M073995 | 0.543539643287659 | https://www.genecards.org/cgi-bin/carddisp.pl?gene=CXCL5 |
| IFNL3 | Interferon Lambda 3 | Protein Coding | Q8IZI9 | 47 | GC19M039243 | 0.543539643287659 | https://www.genecards.org/cgi-bin/carddisp.pl?gene=IFNL3 |
| NNAT | Neuronatin | Protein Coding | Q16517 | 42 | GC20P037521 | 0.543539643287659 | https://www.genecards.org/cgi-bin/carddisp.pl?gene=NNAT |
| MIR143 | MicroRNA 143 | RNA Gene |  | 32 | GC05P159244 | 0.543539643287659 | https://www.genecards.org/cgi-bin/carddisp.pl?gene=MIR143 |
| MIR222 | MicroRNA 222 | RNA Gene |  | 29 | GC0XM045747 | 0.543539643287659 | https://www.genecards.org/cgi-bin/carddisp.pl?gene=MIR222 |
| MIR335 | MicroRNA 335 | RNA Gene |  | 28 | GC07P130496 | 0.543539643287659 | https://www.genecards.org/cgi-bin/carddisp.pl?gene=MIR335 |
| MSBP1 | Minisatellite Binding Protein 1 | Protein Coding |  | 10 | GC00U990213 | 0.543539643287659 | https://www.genecards.org/cgi-bin/carddisp.pl?gene=MSBP1 |
| BMIQ8 | Body Mass Index Quantitative Trait Locus 8 | Genetic Locus |  | 2 | GC10U901185 | 0.543539643287659 | https://www.genecards.org/cgi-bin/carddisp.pl?gene=BMIQ8 |
| FIQTL1 | Fasting Glucose And Specific Insulin Levels | Genetic Locus |  | 2 | GC06U902608 | 0.543539643287659 | https://www.genecards.org/cgi-bin/carddisp.pl?gene=FIQTL1 |
| SLC2A2 | Solute Carrier Family 2 Member 2 | Protein Coding | P11168 | 63 | GC03M171024 | 0.498346239328384 | https://www.genecards.org/cgi-bin/carddisp.pl?gene=SLC2A2 |
| CYP1B1 | Cytochrome P450 Family 1 Subfamily B Member 1 | Protein Coding | Q16678 | 61 | GC02M038066 | 0.498346239328384 | https://www.genecards.org/cgi-bin/carddisp.pl?gene=CYP1B1 |
| EPOR | Erythropoietin Receptor | Protein Coding | P19235 | 61 | GC19M011377 | 0.498346239328384 | https://www.genecards.org/cgi-bin/carddisp.pl?gene=EPOR |
| NOD2 | Nucleotide Binding Oligomerization Domain Containing 2 | Protein Coding | Q9HC29 | 61 | GC16P050693 | 0.498346239328384 | https://www.genecards.org/cgi-bin/carddisp.pl?gene=NOD2 |
| PTK2 | Protein Tyrosine Kinase 2 | Protein Coding | Q05397 | 61 | GC08M140657 | 0.498346239328384 | https://www.genecards.org/cgi-bin/carddisp.pl?gene=PTK2 |
| HDAC5 | Histone Deacetylase 5 | Protein Coding | Q9UQL6 | 60 | GC17M044076 | 0.498346239328384 | https://www.genecards.org/cgi-bin/carddisp.pl?gene=HDAC5 |
| PDCD1 | Programmed Cell Death 1 | Protein Coding | Q15116 | 60 | GC02M241849 | 0.498346239328384 | https://www.genecards.org/cgi-bin/carddisp.pl?gene=PDCD1 |
| ATP5F1A | ATP Synthase F1 Subunit Alpha | Protein Coding | P25705 | 59 | GC18M046081 | 0.498346239328384 | https://www.genecards.org/cgi-bin/carddisp.pl?gene=ATP5F1A |
| GOT1 | Glutamic-Oxaloacetic Transaminase 1 | Protein Coding | P17174 | 58 | GC10M099396 | 0.498346239328384 | https://www.genecards.org/cgi-bin/carddisp.pl?gene=GOT1 |
| IKBKE | Inhibitor Of Nuclear Factor Kappa B Kinase Subunit Epsilon | Protein Coding | Q14164 | 58 | GC01P206470 | 0.498346239328384 | https://www.genecards.org/cgi-bin/carddisp.pl?gene=IKBKE |
| PEBP1 | Phosphatidylethanolamine Binding Protein 1 | Protein Coding | P30086 | 58 | GC12P118189 | 0.498346239328384 | https://www.genecards.org/cgi-bin/carddisp.pl?gene=PEBP1 |
| BDKRB2 | Bradykinin Receptor B2 | Protein Coding | P30411 | 57 | GC14P096205 | 0.498346239328384 | https://www.genecards.org/cgi-bin/carddisp.pl?gene=BDKRB2 |
| CUX1 | Cut Like Homeobox 1 | Protein Coding | Q13948 | 57 | GC07P101815 | 0.498346239328384 | https://www.genecards.org/cgi-bin/carddisp.pl?gene=CUX1 |
| IL3 | Interleukin 3 | Protein Coding | P08700 | 57 | GC05P132060 | 0.498346239328384 | https://www.genecards.org/cgi-bin/carddisp.pl?gene=IL3 |
| INHBA | Inhibin Subunit Beta A | Protein Coding | P08476 | 57 | GC07M041670 | 0.498346239328384 | https://www.genecards.org/cgi-bin/carddisp.pl?gene=INHBA |
| LCT | Lactase | Protein Coding | P09848 | 57 | GC02M135787 | 0.498346239328384 | https://www.genecards.org/cgi-bin/carddisp.pl?gene=LCT |
| NNMT | Nicotinamide N-Methyltransferase | Protein Coding | P40261 | 57 | GC11P114257 | 0.498346239328384 | https://www.genecards.org/cgi-bin/carddisp.pl?gene=NNMT |
| SERPINB2 | Serpin Family B Member 2 | Protein Coding | P05120 | 57 | GC18P063871 | 0.498346239328384 | https://www.genecards.org/cgi-bin/carddisp.pl?gene=SERPINB2 |
| LGALS1 | Galectin 1 | Protein Coding | P09382 | 56 | GC22P037675 | 0.498346239328384 | https://www.genecards.org/cgi-bin/carddisp.pl?gene=LGALS1 |
| NID1 | Nidogen 1 | Protein Coding | P14543 | 56 | GC01M235975 | 0.498346239328384 | https://www.genecards.org/cgi-bin/carddisp.pl?gene=NID1 |
| FABP1 | Fatty Acid Binding Protein 1 | Protein Coding | P07148 | 55 | GC02M088122 | 0.498346239328384 | https://www.genecards.org/cgi-bin/carddisp.pl?gene=FABP1 |
| GADD45A | Growth Arrest And DNA Damage Inducible Alpha | Protein Coding | P24522 | 55 | GC01P067685 | 0.498346239328384 | https://www.genecards.org/cgi-bin/carddisp.pl?gene=GADD45A |
| GSTM1 | Glutathione S-Transferase Mu 1 | Protein Coding | P09488 | 55 | GC01P109687 | 0.498346239328384 | https://www.genecards.org/cgi-bin/carddisp.pl?gene=GSTM1 |
| NOD1 | Nucleotide Binding Oligomerization Domain Containing 1 | Protein Coding | Q9Y239 | 55 | GC07M030424 | 0.498346239328384 | https://www.genecards.org/cgi-bin/carddisp.pl?gene=NOD1 |
| OSM | Oncostatin M | Protein Coding | P13725 | 55 | GC22M030262 | 0.498346239328384 | https://www.genecards.org/cgi-bin/carddisp.pl?gene=OSM |
| SULT1E1 | Sulfotransferase Family 1E Member 1 | Protein Coding | P49888 | 55 | GC04M069823 | 0.498346239328384 | https://www.genecards.org/cgi-bin/carddisp.pl?gene=SULT1E1 |
| BPI | Bactericidal Permeability Increasing Protein | Protein Coding | P17213 | 54 | GC20P053399 | 0.498346239328384 | https://www.genecards.org/cgi-bin/carddisp.pl?gene=BPI |
| POU1F1 | POU Class 1 Homeobox 1 | Protein Coding | P28069 | 54 | GC03M087259 | 0.498346239328384 | https://www.genecards.org/cgi-bin/carddisp.pl?gene=POU1F1 |
| TAC1 | Tachykinin Precursor 1 | Protein Coding | P20366 | 54 | GC07P097734 | 0.498346239328384 | https://www.genecards.org/cgi-bin/carddisp.pl?gene=TAC1 |
| TRIB3 | Tribbles Pseudokinase 3 | Protein Coding | Q96RU7 | 54 | GC20P000528 | 0.498346239328384 | https://www.genecards.org/cgi-bin/carddisp.pl?gene=TRIB3 |
| TET3 | Tet Methylcytosine Dioxygenase 3 | Protein Coding | O43151 | 53 | GC02P074106 | 0.498346239328384 | https://www.genecards.org/cgi-bin/carddisp.pl?gene=TET3 |
| VEGFD | Vascular Endothelial Growth Factor D | Protein Coding | O43915 | 53 | GC0XM015345 | 0.498346239328384 | https://www.genecards.org/cgi-bin/carddisp.pl?gene=VEGFD |
| CRTC2 | CREB Regulated Transcription Coactivator 2 | Protein Coding | Q53ET0 | 52 | GC01M153947 | 0.498346239328384 | https://www.genecards.org/cgi-bin/carddisp.pl?gene=CRTC2 |
| INSRR | Insulin Receptor Related Receptor | Protein Coding | P14616 | 52 | GC01M156840 | 0.498346239328384 | https://www.genecards.org/cgi-bin/carddisp.pl?gene=INSRR |
| CCL26 | C-C Motif Chemokine Ligand 26 | Protein Coding | Q9Y258 | 50 | GC07M075769 | 0.498346239328384 | https://www.genecards.org/cgi-bin/carddisp.pl?gene=CCL26 |
| GPR101 | G Protein-Coupled Receptor 101 | Protein Coding | Q96P66 | 50 | GC0XM137030 | 0.498346239328384 | https://www.genecards.org/cgi-bin/carddisp.pl?gene=GPR101 |
| RNLS | Renalase, FAD Dependent Amine Oxidase | Protein Coding | Q5VYX0 | 50 | GC10M088723 | 0.498346239328384 | https://www.genecards.org/cgi-bin/carddisp.pl?gene=RNLS |
| CCL13 | C-C Motif Chemokine Ligand 13 | Protein Coding | Q99616 | 49 | GC17P034356 | 0.498346239328384 | https://www.genecards.org/cgi-bin/carddisp.pl?gene=CCL13 |
| TAS2R38 | Taste 2 Receptor Member 38 | Protein Coding | P59533 | 48 | GC07M141972 | 0.498346239328384 | https://www.genecards.org/cgi-bin/carddisp.pl?gene=TAS2R38 |
| EGFL6 | EGF Like Domain Multiple 6 | Protein Coding | Q8IUX8 | 47 | GC0XP013569 | 0.498346239328384 | https://www.genecards.org/cgi-bin/carddisp.pl?gene=EGFL6 |
| QRFP | Pyroglutamylated RFamide Peptide | Protein Coding | P83859 | 37 | GC09M130892 | 0.498346239328384 | https://www.genecards.org/cgi-bin/carddisp.pl?gene=QRFP |
| GNRHR2 | Gonadotropin Releasing Hormone Receptor 2 (Pseudogene) | Pseudogene | Q96P88 | 36 | GC01P145919 | 0.498346239328384 | https://www.genecards.org/cgi-bin/carddisp.pl?gene=GNRHR2 |
| ATP6V0D1-DT | ATP6V0D1 Divergent Transcript | RNA Gene |  | 12 | GC16P121877 | 0.498346239328384 | https://www.genecards.org/cgi-bin/carddisp.pl?gene=ATP6V0D1-DT |
| TRL-AAG2-3 | TRNA-Leu (Anticodon AAG) 2-3 | RNA Gene |  | 11 | GC14P057241 | 0.498346239328384 | https://www.genecards.org/cgi-bin/carddisp.pl?gene=TRL-AAG2-3 |
| ADIPQTL3 | Circulating Adiponectin QTL On Chromosome 14 | Genetic Locus |  | 2 | GC14U901513 | 0.498346239328384 | https://www.genecards.org/cgi-bin/carddisp.pl?gene=ADIPQTL3 |
| GP6 | Glycoprotein VI Platelet | Protein Coding | Q9HCN6 | 58 | GC19M055013 | 0.47159206867218 | https://www.genecards.org/cgi-bin/carddisp.pl?gene=GP6 |
| CFTR | CF Transmembrane Conductance Regulator | Protein Coding | P13569 | 66 | GC07P117287 | 0.439449071884155 | https://www.genecards.org/cgi-bin/carddisp.pl?gene=CFTR |
| ADAM17 | ADAM Metallopeptidase Domain 17 | Protein Coding | P78536 | 64 | GC02M009488 | 0.439449071884155 | https://www.genecards.org/cgi-bin/carddisp.pl?gene=ADAM17 |
| ALK | ALK Receptor Tyrosine Kinase | Protein Coding | Q9UM73 | 64 | GC02M029307 | 0.439449071884155 | https://www.genecards.org/cgi-bin/carddisp.pl?gene=ALK |
| EIF2AK3 | Eukaryotic Translation Initiation Factor 2 Alpha Kinase 3 | Protein Coding | Q9NZJ5 | 63 | GC02M088556 | 0.439449071884155 | https://www.genecards.org/cgi-bin/carddisp.pl?gene=EIF2AK3 |
| NEK2 | NIMA Related Kinase 2 | Protein Coding | P51955 | 63 | GC01M211658 | 0.439449071884155 | https://www.genecards.org/cgi-bin/carddisp.pl?gene=NEK2 |
| ALOX5 | Arachidonate 5-Lipoxygenase | Protein Coding | P09917 | 62 | GC10P045374 | 0.439449071884155 | https://www.genecards.org/cgi-bin/carddisp.pl?gene=ALOX5 |
| GDNF | Glial Cell Derived Neurotrophic Factor | Protein Coding | P39905 | 62 | GC05M037812 | 0.439449071884155 | https://www.genecards.org/cgi-bin/carddisp.pl?gene=GDNF |
| GSTP1 | Glutathione S-Transferase Pi 1 | Protein Coding | P09211 | 62 | GC11P067583 | 0.439449071884155 | https://www.genecards.org/cgi-bin/carddisp.pl?gene=GSTP1 |
| LRP5 | LDL Receptor Related Protein 5 | Protein Coding | O75197 | 62 | GC11P068298 | 0.439449071884155 | https://www.genecards.org/cgi-bin/carddisp.pl?gene=LRP5 |
| MYD88 | MYD88 Innate Immune Signal Transduction Adaptor | Protein Coding | Q99836 | 62 | GC03P038501 | 0.439449071884155 | https://www.genecards.org/cgi-bin/carddisp.pl?gene=MYD88 |
| ACVR1B | Activin A Receptor Type 1B | Protein Coding | P36896 | 60 | GC12P051951 | 0.439449071884155 | https://www.genecards.org/cgi-bin/carddisp.pl?gene=ACVR1B |
| ADORA1 | Adenosine A1 Receptor | Protein Coding | P30542 | 60 | GC01P203090 | 0.439449071884155 | https://www.genecards.org/cgi-bin/carddisp.pl?gene=ADORA1 |
| DIABLO | Diablo IAP-Binding Mitochondrial Protein | Protein Coding | Q9NR28 | 60 | GC12M124699 | 0.439449071884155 | https://www.genecards.org/cgi-bin/carddisp.pl?gene=DIABLO |
| GP1BA | Glycoprotein Ib Platelet Subunit Alpha | Protein Coding | P07359 | 60 | GC17P004932 | 0.439449071884155 | https://www.genecards.org/cgi-bin/carddisp.pl?gene=GP1BA |
| KLK3 | Kallikrein Related Peptidase 3 | Protein Coding | P07288 | 60 | GC19P050854 | 0.439449071884155 | https://www.genecards.org/cgi-bin/carddisp.pl?gene=KLK3 |
| OGT | O-Linked N-Acetylglucosamine (GlcNAc) Transferase | Protein Coding | O15294 | 60 | GC0XP072033 | 0.439449071884155 | https://www.genecards.org/cgi-bin/carddisp.pl?gene=OGT |
| PAX5 | Paired Box 5 | Protein Coding | Q02548 | 60 | GC09M037145 | 0.439449071884155 | https://www.genecards.org/cgi-bin/carddisp.pl?gene=PAX5 |
| SLC6A2 | Solute Carrier Family 6 Member 2 | Protein Coding | P23975 | 60 | GC16P121574 | 0.439449071884155 | https://www.genecards.org/cgi-bin/carddisp.pl?gene=SLC6A2 |
| TGFB3 | Transforming Growth Factor Beta 3 | Protein Coding | P10600 | 60 | GC14M075958 | 0.439449071884155 | https://www.genecards.org/cgi-bin/carddisp.pl?gene=TGFB3 |
| VCL | Vinculin | Protein Coding | P18206 | 60 | GC10P073995 | 0.439449071884155 | https://www.genecards.org/cgi-bin/carddisp.pl?gene=VCL |
| CA9 | Carbonic Anhydrase 9 | Protein Coding | Q16790 | 59 | GC09P035673 | 0.439449071884155 | https://www.genecards.org/cgi-bin/carddisp.pl?gene=CA9 |
| IDO1 | Indoleamine 2,3-Dioxygenase 1 | Protein Coding | P14902 | 59 | GC08P044830 | 0.439449071884155 | https://www.genecards.org/cgi-bin/carddisp.pl?gene=IDO1 |
| SUMO1 | Small Ubiquitin Like Modifier 1 | Protein Coding | P63165 | 59 | GC02M202206 | 0.439449071884155 | https://www.genecards.org/cgi-bin/carddisp.pl?gene=SUMO1 |
| TNFRSF11A | TNF Receptor Superfamily Member 11a | Protein Coding | Q9Y6Q6 | 59 | GC18P062325 | 0.439449071884155 | https://www.genecards.org/cgi-bin/carddisp.pl?gene=TNFRSF11A |
| VDAC1 | Voltage Dependent Anion Channel 1 | Protein Coding | P21796 | 59 | GC05M133975 | 0.439449071884155 | https://www.genecards.org/cgi-bin/carddisp.pl?gene=VDAC1 |
| DBI | Diazepam Binding Inhibitor, Acyl-CoA Binding Protein | Protein Coding | P07108 | 58 | GC02P119366 | 0.439449071884155 | https://www.genecards.org/cgi-bin/carddisp.pl?gene=DBI |
| ENPP2 | Ectonucleotide Pyrophosphatase/Phosphodiesterase 2 | Protein Coding | Q13822 | 58 | GC08M119556 | 0.439449071884155 | https://www.genecards.org/cgi-bin/carddisp.pl?gene=ENPP2 |
| HDC | Histidine Decarboxylase | Protein Coding | P19113 | 58 | GC15M050241 | 0.439449071884155 | https://www.genecards.org/cgi-bin/carddisp.pl?gene=HDC |
| KDM3B | Lysine Demethylase 3B | Protein Coding | Q7LBC6 | 58 | GC05P138352 | 0.439449071884155 | https://www.genecards.org/cgi-bin/carddisp.pl?gene=KDM3B |
| PTS | 6-Pyruvoyltetrahydropterin Synthase | Protein Coding | Q03393 | 58 | GC11P112226 | 0.439449071884155 | https://www.genecards.org/cgi-bin/carddisp.pl?gene=PTS |
| SI | Sucrase-Isomaltase | Protein Coding | P14410 | 58 | GC03M164978 | 0.439449071884155 | https://www.genecards.org/cgi-bin/carddisp.pl?gene=SI |
| CRK | CRK Proto-Oncogene, Adaptor Protein | Protein Coding | P46108 | 57 | GC17M001420 | 0.439449071884155 | https://www.genecards.org/cgi-bin/carddisp.pl?gene=CRK |
| DPEP1 | Dipeptidase 1 | Protein Coding | P16444 | 57 | GC16P089613 | 0.439449071884155 | https://www.genecards.org/cgi-bin/carddisp.pl?gene=DPEP1 |
| FGF4 | Fibroblast Growth Factor 4 | Protein Coding | P08620 | 57 | GC11M139942 | 0.439449071884155 | https://www.genecards.org/cgi-bin/carddisp.pl?gene=FGF4 |
| FSHB | Follicle Stimulating Hormone Subunit Beta | Protein Coding | P01225 | 57 | GC11P030210 | 0.439449071884155 | https://www.genecards.org/cgi-bin/carddisp.pl?gene=FSHB |
| NTF3 | Neurotrophin 3 | Protein Coding | P20783 | 57 | GC12P074017 | 0.439449071884155 | https://www.genecards.org/cgi-bin/carddisp.pl?gene=NTF3 |
| PTPRB | Protein Tyrosine Phosphatase Receptor Type B | Protein Coding | P23467 | 57 | GC12M070516 | 0.439449071884155 | https://www.genecards.org/cgi-bin/carddisp.pl?gene=PTPRB |
| SLC39A14 | Solute Carrier Family 39 Member 14 | Protein Coding | Q15043 | 57 | GC08P022367 | 0.439449071884155 | https://www.genecards.org/cgi-bin/carddisp.pl?gene=SLC39A14 |
| ARNT2 | Aryl Hydrocarbon Receptor Nuclear Translocator 2 | Protein Coding | Q9HBZ2 | 56 | GC15P080404 | 0.439449071884155 | https://www.genecards.org/cgi-bin/carddisp.pl?gene=ARNT2 |
| CD2AP | CD2 Associated Protein | Protein Coding | Q9Y5K6 | 56 | GC06P182098 | 0.439449071884155 | https://www.genecards.org/cgi-bin/carddisp.pl?gene=CD2AP |
| SELPLG | Selectin P Ligand | Protein Coding | Q14242 | 56 | GC12M108621 | 0.439449071884155 | https://www.genecards.org/cgi-bin/carddisp.pl?gene=SELPLG |
| AP2S1 | Adaptor Related Protein Complex 2 Subunit Sigma 1 | Protein Coding | P53680 | 55 | GC19M046838 | 0.439449071884155 | https://www.genecards.org/cgi-bin/carddisp.pl?gene=AP2S1 |
| FLCN | Folliculin | Protein Coding | Q8NFG4 | 55 | GC17M017212 | 0.439449071884155 | https://www.genecards.org/cgi-bin/carddisp.pl?gene=FLCN |
| GIPC1 | GIPC PDZ Domain Containing Family Member 1 | Protein Coding | O14908 | 55 | GC19M108267 | 0.439449071884155 | https://www.genecards.org/cgi-bin/carddisp.pl?gene=GIPC1 |
| LGMN | Legumain | Protein Coding | Q99538 | 55 | GC14M092703 | 0.439449071884155 | https://www.genecards.org/cgi-bin/carddisp.pl?gene=LGMN |
| S100A8 | S100 Calcium Binding Protein A8 | Protein Coding | P05109 | 55 | GC01M167351 | 0.439449071884155 | https://www.genecards.org/cgi-bin/carddisp.pl?gene=S100A8 |
| SLC25A19 | Solute Carrier Family 25 Member 19 | Protein Coding | Q9HC21 | 55 | GC17M098053 | 0.439449071884155 | https://www.genecards.org/cgi-bin/carddisp.pl?gene=SLC25A19 |
| SLC26A2 | Solute Carrier Family 26 Member 2 | Protein Coding | P50443 | 55 | GC05P159260 | 0.439449071884155 | https://www.genecards.org/cgi-bin/carddisp.pl?gene=SLC26A2 |
| BIRC6 | Baculoviral IAP Repeat Containing 6 | Protein Coding | Q9NR09 | 54 | GC02P032357 | 0.439449071884155 | https://www.genecards.org/cgi-bin/carddisp.pl?gene=BIRC6 |
| FAR1 | Fatty Acyl-CoA Reductase 1 | Protein Coding | Q8WVX9 | 54 | GC11P013668 | 0.439449071884155 | https://www.genecards.org/cgi-bin/carddisp.pl?gene=FAR1 |
| HOMER1 | Homer Scaffold Protein 1 | Protein Coding | Q86YM7 | 54 | GC05M079372 | 0.439449071884155 | https://www.genecards.org/cgi-bin/carddisp.pl?gene=HOMER1 |
| HOMER2 | Homer Scaffold Protein 2 | Protein Coding | Q9NSB8 | 54 | GC15M162994 | 0.439449071884155 | https://www.genecards.org/cgi-bin/carddisp.pl?gene=HOMER2 |
| PSMD9 | Proteasome 26S Subunit, Non-ATPase 9 | Protein Coding | O00233 | 54 | GC12P138925 | 0.439449071884155 | https://www.genecards.org/cgi-bin/carddisp.pl?gene=PSMD9 |
| SDC3 | Syndecan 3 | Protein Coding | O75056 | 54 | GC01M030869 | 0.439449071884155 | https://www.genecards.org/cgi-bin/carddisp.pl?gene=SDC3 |
| SIGLEC8 | Sialic Acid Binding Ig Like Lectin 8 | Protein Coding | Q9NYZ4 | 54 | GC19M051450 | 0.439449071884155 | https://www.genecards.org/cgi-bin/carddisp.pl?gene=SIGLEC8 |
| TIMP2 | TIMP Metallopeptidase Inhibitor 2 | Protein Coding | P16035 | 54 | GC17M078852 | 0.439449071884155 | https://www.genecards.org/cgi-bin/carddisp.pl?gene=TIMP2 |
| UTRN | Utrophin | Protein Coding | P46939 | 53 | GC06P144285 | 0.439449071884155 | https://www.genecards.org/cgi-bin/carddisp.pl?gene=UTRN |
| CAPZA2 | Capping Actin Protein Of Muscle Z-Line Subunit Alpha 2 | Protein Coding | P47755 | 52 | GC07P116811 | 0.439449071884155 | https://www.genecards.org/cgi-bin/carddisp.pl?gene=CAPZA2 |
| DIO3 | Iodothyronine Deiodinase 3 | Protein Coding | P55073 | 52 | GC14P120458 | 0.439449071884155 | https://www.genecards.org/cgi-bin/carddisp.pl?gene=DIO3 |
| ITGA11 | Integrin Subunit Alpha 11 | Protein Coding | Q9UKX5 | 52 | GC15M068296 | 0.439449071884155 | https://www.genecards.org/cgi-bin/carddisp.pl?gene=ITGA11 |
| PSCA | Prostate Stem Cell Antigen | Protein Coding | O43653 | 52 | GC08P142670 | 0.439449071884155 | https://www.genecards.org/cgi-bin/carddisp.pl?gene=PSCA |
| CCP110 | Centriolar Coiled-Coil Protein 110 | Protein Coding | O43303 | 51 | GC16P120606 | 0.439449071884155 | https://www.genecards.org/cgi-bin/carddisp.pl?gene=CCP110 |
| PCMT1 | Protein-L-Isoaspartate (D-Aspartate) O-Methyltransferase | Protein Coding | P22061 | 51 | GC06P149749 | 0.439449071884155 | https://www.genecards.org/cgi-bin/carddisp.pl?gene=PCMT1 |
| PENK | Proenkephalin | Protein Coding | P01210 | 51 | GC08M056436 | 0.439449071884155 | https://www.genecards.org/cgi-bin/carddisp.pl?gene=PENK |
| SLC5A4 | Solute Carrier Family 5 Member 4 | Protein Coding | Q9NY91 | 51 | GC22M032218 | 0.439449071884155 | https://www.genecards.org/cgi-bin/carddisp.pl?gene=SLC5A4 |
| TACC1 | Transforming Acidic Coiled-Coil Containing Protein 1 | Protein Coding | O75410 | 51 | GC08P038728 | 0.439449071884155 | https://www.genecards.org/cgi-bin/carddisp.pl?gene=TACC1 |
| C1QTNF5 | C1q And TNF Related 5 | Protein Coding | Q9BXJ0 | 50 | GC11M119338 | 0.439449071884155 | https://www.genecards.org/cgi-bin/carddisp.pl?gene=C1QTNF5 |
| CKS2 | CDC28 Protein Kinase Regulatory Subunit 2 | Protein Coding | P33552 | 50 | GC09P089311 | 0.439449071884155 | https://www.genecards.org/cgi-bin/carddisp.pl?gene=CKS2 |
| ENOPH1 | Enolase-Phosphatase 1 | Protein Coding | Q9UHY7 | 50 | GC04P082430 | 0.439449071884155 | https://www.genecards.org/cgi-bin/carddisp.pl?gene=ENOPH1 |
| IL32 | Interleukin 32 | Protein Coding | P24001 | 50 | GC16P120203 | 0.439449071884155 | https://www.genecards.org/cgi-bin/carddisp.pl?gene=IL32 |
| RIF1 | Replication Timing Regulatory Factor 1 | Protein Coding | Q5UIP0 | 50 | GC02P151409 | 0.439449071884155 | https://www.genecards.org/cgi-bin/carddisp.pl?gene=RIF1 |
| STX6 | Syntaxin 6 | Protein Coding | O43752 | 50 | GC01M180972 | 0.439449071884155 | https://www.genecards.org/cgi-bin/carddisp.pl?gene=STX6 |
| WWTR1 | WW Domain Containing Transcription Regulator 1 | Protein Coding | Q9GZV5 | 50 | GC03M149517 | 0.439449071884155 | https://www.genecards.org/cgi-bin/carddisp.pl?gene=WWTR1 |
| ZBTB33 | Zinc Finger And BTB Domain Containing 33 | Protein Coding | Q86T24 | 50 | GC0XP120250 | 0.439449071884155 | https://www.genecards.org/cgi-bin/carddisp.pl?gene=ZBTB33 |
| ANKK1 | Ankyrin Repeat And Kinase Domain Containing 1 | Protein Coding | Q8NFD2 | 49 | GC11P113387 | 0.439449071884155 | https://www.genecards.org/cgi-bin/carddisp.pl?gene=ANKK1 |
| CXCL6 | C-X-C Motif Chemokine Ligand 6 | Protein Coding | P80162 | 49 | GC04P073837 | 0.439449071884155 | https://www.genecards.org/cgi-bin/carddisp.pl?gene=CXCL6 |
| SIPA1L3 | Signal Induced Proliferation Associated 1 Like 3 | Protein Coding | O60292 | 49 | GC19P152447 | 0.439449071884155 | https://www.genecards.org/cgi-bin/carddisp.pl?gene=SIPA1L3 |
| AHNAK | AHNAK Nucleoprotein | Protein Coding | Q09666 | 48 | GC11M139644 | 0.439449071884155 | https://www.genecards.org/cgi-bin/carddisp.pl?gene=AHNAK |
| CACYBP | Calcyclin Binding Protein | Protein Coding | Q9HB71 | 48 | GC01P177461 | 0.439449071884155 | https://www.genecards.org/cgi-bin/carddisp.pl?gene=CACYBP |
| CEP97 | Centrosomal Protein 97 | Protein Coding | Q8IW35 | 48 | GC03P102363 | 0.439449071884155 | https://www.genecards.org/cgi-bin/carddisp.pl?gene=CEP97 |
| NAV1 | Neuron Navigator 1 | Protein Coding | Q8NEY1 | 48 | GC01P201712 | 0.439449071884155 | https://www.genecards.org/cgi-bin/carddisp.pl?gene=NAV1 |
| PIGF | Phosphatidylinositol Glycan Anchor Biosynthesis Class F | Protein Coding | Q07326 | 48 | GC02M046580 | 0.439449071884155 | https://www.genecards.org/cgi-bin/carddisp.pl?gene=PIGF |
| DNAJB12 | DnaJ Heat Shock Protein Family (Hsp40) Member B12 | Protein Coding | Q9NXW2 | 47 | GC10M072332 | 0.439449071884155 | https://www.genecards.org/cgi-bin/carddisp.pl?gene=DNAJB12 |
| FAM83H | Family With Sequence Similarity 83 Member H | Protein Coding | Q6ZRV2 | 47 | GC08M143723 | 0.439449071884155 | https://www.genecards.org/cgi-bin/carddisp.pl?gene=FAM83H |
| VCPIP1 | Valosin Containing Protein Interacting Protein 1 | Protein Coding | Q96JH7 | 47 | GC08M066628 | 0.439449071884155 | https://www.genecards.org/cgi-bin/carddisp.pl?gene=VCPIP1 |
| XRN1 | 5'-3' Exoribonuclease 1 | Protein Coding | Q8IZH2 | 47 | GC03M142306 | 0.439449071884155 | https://www.genecards.org/cgi-bin/carddisp.pl?gene=XRN1 |
| MRTFB | Myocardin Related Transcription Factor B | Protein Coding | Q9ULH7 | 46 | GC16P013994 | 0.439449071884155 | https://www.genecards.org/cgi-bin/carddisp.pl?gene=MRTFB |
| LVRN | Laeverin | Protein Coding | Q6Q4G3 | 45 | GC05P115962 | 0.439449071884155 | https://www.genecards.org/cgi-bin/carddisp.pl?gene=LVRN |
| METRNL | Meteorin Like, Glial Cell Differentiation Regulator | Protein Coding | Q641Q3 | 45 | GC17P083079 | 0.439449071884155 | https://www.genecards.org/cgi-bin/carddisp.pl?gene=METRNL |
| MTCL1 | Microtubule Crosslinking Factor 1 | Protein Coding | Q9Y4B5 | 45 | GC18P008705 | 0.439449071884155 | https://www.genecards.org/cgi-bin/carddisp.pl?gene=MTCL1 |
| RLN3 | Relaxin 3 | Protein Coding | Q8WXF3 | 45 | GC19P151984 | 0.439449071884155 | https://www.genecards.org/cgi-bin/carddisp.pl?gene=RLN3 |
| SCARA3 | Scavenger Receptor Class A Member 3 | Protein Coding | Q6AZY7 | 45 | GC08P027633 | 0.439449071884155 | https://www.genecards.org/cgi-bin/carddisp.pl?gene=SCARA3 |
| SHTN1 | Shootin 1 | Protein Coding | A0MZ66 | 45 | GC10M116903 | 0.439449071884155 | https://www.genecards.org/cgi-bin/carddisp.pl?gene=SHTN1 |
| TACC2 | Transforming Acidic Coiled-Coil Containing Protein 2 | Protein Coding | O95359 | 45 | GC10P121989 | 0.439449071884155 | https://www.genecards.org/cgi-bin/carddisp.pl?gene=TACC2 |
| TTC1 | Tetratricopeptide Repeat Domain 1 | Protein Coding | Q99614 | 45 | GC05P160009 | 0.439449071884155 | https://www.genecards.org/cgi-bin/carddisp.pl?gene=TTC1 |
| FABP12 | Fatty Acid Binding Protein 12 | Protein Coding | A6NFH5 | 44 | GC08M081524 | 0.439449071884155 | https://www.genecards.org/cgi-bin/carddisp.pl?gene=FABP12 |
| ZNF281 | Zinc Finger Protein 281 | Protein Coding | Q9Y2X9 | 44 | GC01M200404 | 0.439449071884155 | https://www.genecards.org/cgi-bin/carddisp.pl?gene=ZNF281 |
| ASB6 | Ankyrin Repeat And SOCS Box Containing 6 | Protein Coding | Q9NWX5 | 43 | GC09M131905 | 0.439449071884155 | https://www.genecards.org/cgi-bin/carddisp.pl?gene=ASB6 |
| CORO1B | Coronin 1B | Protein Coding | Q9BR76 | 42 | GC11M067435 | 0.439449071884155 | https://www.genecards.org/cgi-bin/carddisp.pl?gene=CORO1B |
| IFIT5 | Interferon Induced Protein With Tetratricopeptide Repeats 5 | Protein Coding | Q13325 | 42 | GC10P089414 | 0.439449071884155 | https://www.genecards.org/cgi-bin/carddisp.pl?gene=IFIT5 |
| LZTS2 | Leucine Zipper Tumor Suppressor 2 | Protein Coding | Q9BRK4 | 41 | GC10P100996 | 0.439449071884155 | https://www.genecards.org/cgi-bin/carddisp.pl?gene=LZTS2 |
| ZNF32 | Zinc Finger Protein 32 | Protein Coding | P17041 | 41 | GC10M043643 | 0.439449071884155 | https://www.genecards.org/cgi-bin/carddisp.pl?gene=ZNF32 |
| MTCL2 | Microtubule Crosslinking Factor 2 | Protein Coding | O94964 | 40 | GC20M036777 | 0.439449071884155 | https://www.genecards.org/cgi-bin/carddisp.pl?gene=MTCL2 |
| NKX1-1 | NK1 Homeobox 1 | Protein Coding | Q15270 | 40 | GC04M006274 | 0.439449071884155 | https://www.genecards.org/cgi-bin/carddisp.pl?gene=NKX1-1 |
| JPT2 | Jupiter Microtubule Associated Homolog 2 | Protein Coding | Q9H910 | 39 | GC16P120151 | 0.439449071884155 | https://www.genecards.org/cgi-bin/carddisp.pl?gene=JPT2 |
| SMIM1 | Small Integral Membrane Protein 1 (Vel Blood Group) | Protein Coding | B2RUZ4 | 38 | GC01P003772 | 0.439449071884155 | https://www.genecards.org/cgi-bin/carddisp.pl?gene=SMIM1 |
| IFFO2 | Intermediate Filament Family Orphan 2 | Protein Coding | Q5TF58 | 35 | GC01M018904 | 0.439449071884155 | https://www.genecards.org/cgi-bin/carddisp.pl?gene=IFFO2 |
| TRG | T Cell Receptor Gamma Locus | Protein Coding |  | 20 | GC07M038240 | 0.439449071884155 | https://www.genecards.org/cgi-bin/carddisp.pl?gene=TRG |
| ARHGAP19-SLIT1 | ARHGAP19-SLIT1 Readthrough (NMD Candidate) | RNA Gene |  | 19 | GC10M097153 | 0.439449071884155 | https://www.genecards.org/cgi-bin/carddisp.pl?gene=ARHGAP19-SLIT1 |
| SIM1-AS1 | SIM1 Antisense RNA 1 | RNA Gene |  | 14 | GC06P100393 | 0.439449071884155 | https://www.genecards.org/cgi-bin/carddisp.pl?gene=SIM1-AS1 |
| COPS5P1 | COP9 Signalosome Subunit 5 Pseudogene 1 | Pseudogene |  | 10 | GC06M093091 | 0.439449071884155 | https://www.genecards.org/cgi-bin/carddisp.pl?gene=COPS5P1 |
| RPL12P33 | Ribosomal Protein L12 Pseudogene 33 | Pseudogene |  | 10 | GC12P120916 | 0.439449071884155 | https://www.genecards.org/cgi-bin/carddisp.pl?gene=RPL12P33 |
| AOMS1 | Abdominal Obesity-Metabolic Syndrome QTL1 | Genetic Locus |  | 4 | GC03U990183 | 0.439449071884155 | https://www.genecards.org/cgi-bin/carddisp.pl?gene=AOMS1 |
| BMIQ1 | Body Mass Index QTL 1 | Genetic Locus |  | 3 | GC07U990348 | 0.439449071884155 | https://www.genecards.org/cgi-bin/carddisp.pl?gene=BMIQ1 |
| AOMS2 | Abdominal Obesity-Metabolic Syndrome QTL2 | Genetic Locus |  | 2 | GC17U990216 | 0.439449071884155 | https://www.genecards.org/cgi-bin/carddisp.pl?gene=AOMS2 |
| BMIQ2 | Body Mass Index QTL 2 | Genetic Locus |  | 2 | GC13U990085 | 0.439449071884155 | https://www.genecards.org/cgi-bin/carddisp.pl?gene=BMIQ2 |
| BMIQ3 | Body Mass Index QTL 3 | Genetic Locus |  | 2 | GC06U900016 | 0.439449071884155 | https://www.genecards.org/cgi-bin/carddisp.pl?gene=BMIQ3 |
| MEFV | MEFV Innate Immunity Regulator, Pyrin | Protein Coding | O15553 | 57 | GC16M049968 | 0.426571071147919 | https://www.genecards.org/cgi-bin/carddisp.pl?gene=MEFV |
| CST3 | Cystatin C | Protein Coding | P01034 | 56 | GC20M024204 | 0.426571071147919 | https://www.genecards.org/cgi-bin/carddisp.pl?gene=CST3 |
| KCNA2 | Potassium Voltage-Gated Channel Subfamily A Member 2 | Protein Coding | P16389 | 59 | GC01M110519 | 0.420387297868729 | https://www.genecards.org/cgi-bin/carddisp.pl?gene=KCNA2 |
| BRS3 | Bombesin Receptor Subtype 3 | Protein Coding | P32247 | 54 | GC0XP136543 | 0.420387297868729 | https://www.genecards.org/cgi-bin/carddisp.pl?gene=BRS3 |
| LPIN2 | Lipin 2 | Protein Coding | Q92539 | 52 | GC18M004017 | 0.420387297868729 | https://www.genecards.org/cgi-bin/carddisp.pl?gene=LPIN2 |
| LPIN3 | Lipin 3 | Protein Coding | Q9BQK8 | 44 | GC20P041340 | 0.420387297868729 | https://www.genecards.org/cgi-bin/carddisp.pl?gene=LPIN3 |
| C1QTNF7 | C1q And TNF Related 7 | Protein Coding | Q9BXJ2 | 43 | GC04P015339 | 0.420387297868729 | https://www.genecards.org/cgi-bin/carddisp.pl?gene=C1QTNF7 |
| C1QL2 | Complement C1q Like 2 | Protein Coding | Q7Z5L3 | 41 | GC02M119156 | 0.420387297868729 | https://www.genecards.org/cgi-bin/carddisp.pl?gene=C1QL2 |
| SNORD24 | Small Nucleolar RNA, C/D Box 24 | RNA Gene |  | 24 | GC09P133349 | 0.420387297868729 | https://www.genecards.org/cgi-bin/carddisp.pl?gene=SNORD24 |
| LOC101929710 | Uncharacterized LOC101929710 | RNA Gene |  | 21 | GC05P095962 | 0.420387297868729 | https://www.genecards.org/cgi-bin/carddisp.pl?gene=LOC101929710 |
| AFP | Alpha Fetoprotein | Protein Coding | P02771 | 60 | GC04P073431 | 0.376200318336487 | https://www.genecards.org/cgi-bin/carddisp.pl?gene=AFP |
| SCT | Secretin | Protein Coding | P09683 | 47 | GC11M000626 | 0.376200318336487 | https://www.genecards.org/cgi-bin/carddisp.pl?gene=SCT |
| F2 | Coagulation Factor II, Thrombin | Protein Coding | P00734 | 63 | GC11P049059 | 0.348293840885162 | https://www.genecards.org/cgi-bin/carddisp.pl?gene=F2 |
| ENTPD1 | Ectonucleoside Triphosphate Diphosphohydrolase 1 | Protein Coding | P49961 | 62 | GC10P124203 | 0.348293840885162 | https://www.genecards.org/cgi-bin/carddisp.pl?gene=ENTPD1 |
| BAD | BCL2 Associated Agonist Of Cell Death | Protein Coding | Q92934 | 57 | GC11M139722 | 0.348293840885162 | https://www.genecards.org/cgi-bin/carddisp.pl?gene=BAD |
| ATXN2 | Ataxin 2 | Protein Coding | Q99700 | 56 | GC12M111443 | 0.348293840885162 | https://www.genecards.org/cgi-bin/carddisp.pl?gene=ATXN2 |
| ATG16L1 | Autophagy Related 16 Like 1 | Protein Coding | Q676U5 | 55 | GC02P235056 | 0.348293840885162 | https://www.genecards.org/cgi-bin/carddisp.pl?gene=ATG16L1 |
| IL20 | Interleukin 20 | Protein Coding | Q9NYY1 | 50 | GC01P206866 | 0.348293840885162 | https://www.genecards.org/cgi-bin/carddisp.pl?gene=IL20 |
| PRR4 | Proline Rich 4 | Protein Coding | Q16378 | 41 | GC12M035811 | 0.348293840885162 | https://www.genecards.org/cgi-bin/carddisp.pl?gene=PRR4 |
| PRKAG2 | Protein Kinase AMP-Activated Non-Catalytic Subunit Gamma 2 | Protein Coding | Q9UGJ0 | 62 | GC07M151556 | 0.317947298288345 | https://www.genecards.org/cgi-bin/carddisp.pl?gene=PRKAG2 |
| PCK1 | Phosphoenolpyruvate Carboxykinase 1 | Protein Coding | P35558 | 60 | GC20P057561 | 0.317947298288345 | https://www.genecards.org/cgi-bin/carddisp.pl?gene=PCK1 |
| ADCYAP1 | Adenylate Cyclase Activating Polypeptide 1 | Protein Coding | P18509 | 55 | GC18P000895 | 0.317947298288345 | https://www.genecards.org/cgi-bin/carddisp.pl?gene=ADCYAP1 |
| SNX3 | Sorting Nexin 3 | Protein Coding | O60493 | 52 | GC06M108211 | 0.317947298288345 | https://www.genecards.org/cgi-bin/carddisp.pl?gene=SNX3 |
| COL20A1 | Collagen Type XX Alpha 1 Chain | Protein Coding | Q9P218 | 47 | GC20P063293 | 0.317947298288345 | https://www.genecards.org/cgi-bin/carddisp.pl?gene=COL20A1 |
| FGFR3 | Fibroblast Growth Factor Receptor 3 | Protein Coding | P22607 | 68 | GC04P029505 | 0.297258704900742 | https://www.genecards.org/cgi-bin/carddisp.pl?gene=FGFR3 |
| BRAF | B-Raf Proto-Oncogene, Serine/Threonine Kinase | Protein Coding | P15056 | 67 | GC07M140793 | 0.297258704900742 | https://www.genecards.org/cgi-bin/carddisp.pl?gene=BRAF |
| CREBBP | CREB Binding Protein | Protein Coding | Q92793 | 67 | GC16M049995 | 0.297258704900742 | https://www.genecards.org/cgi-bin/carddisp.pl?gene=CREBBP |
| EPHB4 | EPH Receptor B4 | Protein Coding | P54760 | 66 | GC07M106759 | 0.297258704900742 | https://www.genecards.org/cgi-bin/carddisp.pl?gene=EPHB4 |
| GATA2 | GATA Binding Protein 2 | Protein Coding | P23769 | 62 | GC03M128479 | 0.297258704900742 | https://www.genecards.org/cgi-bin/carddisp.pl?gene=GATA2 |
| KCNQ1 | Potassium Voltage-Gated Channel Subfamily Q Member 1 | Protein Coding | P51787 | 62 | GC11P002444 | 0.297258704900742 | https://www.genecards.org/cgi-bin/carddisp.pl?gene=KCNQ1 |
| RORA | RAR Related Orphan Receptor A | Protein Coding | P35398 | 61 | GC15M060488 | 0.297258704900742 | https://www.genecards.org/cgi-bin/carddisp.pl?gene=RORA |
| TTN | Titin | Protein Coding | Q8WZ42 | 61 | GC02M178525 | 0.297258704900742 | https://www.genecards.org/cgi-bin/carddisp.pl?gene=TTN |
| CD22 | CD22 Molecule | Protein Coding | P20273 | 60 | GC19P035319 | 0.297258704900742 | https://www.genecards.org/cgi-bin/carddisp.pl?gene=CD22 |
| CYP1A1 | Cytochrome P450 Family 1 Subfamily A Member 1 | Protein Coding | P04798 | 60 | GC15M074719 | 0.297258704900742 | https://www.genecards.org/cgi-bin/carddisp.pl?gene=CYP1A1 |
| ERN1 | Endoplasmic Reticulum To Nucleus Signaling 1 | Protein Coding | O75460 | 60 | GC17M064039 | 0.297258704900742 | https://www.genecards.org/cgi-bin/carddisp.pl?gene=ERN1 |
| MYH9 | Myosin Heavy Chain 9 | Protein Coding | P35579 | 60 | GC22M036281 | 0.297258704900742 | https://www.genecards.org/cgi-bin/carddisp.pl?gene=MYH9 |
| PGM1 | Phosphoglucomutase 1 | Protein Coding | P36871 | 60 | GC01P063593 | 0.297258704900742 | https://www.genecards.org/cgi-bin/carddisp.pl?gene=PGM1 |
| CDKN2B | Cyclin Dependent Kinase Inhibitor 2B | Protein Coding | P42772 | 59 | GC09M022002 | 0.297258704900742 | https://www.genecards.org/cgi-bin/carddisp.pl?gene=CDKN2B |
| GNAI2 | G Protein Subunit Alpha I2 | Protein Coding | P04899 | 59 | GC03P050226 | 0.297258704900742 | https://www.genecards.org/cgi-bin/carddisp.pl?gene=GNAI2 |
| AVPR1A | Arginine Vasopressin Receptor 1A | Protein Coding | P37288 | 58 | GC12M063142 | 0.297258704900742 | https://www.genecards.org/cgi-bin/carddisp.pl?gene=AVPR1A |
| CTSS | Cathepsin S | Protein Coding | P25774 | 58 | GC01M150730 | 0.297258704900742 | https://www.genecards.org/cgi-bin/carddisp.pl?gene=CTSS |
| EIF2S1 | Eukaryotic Translation Initiation Factor 2 Subunit Alpha | Protein Coding | P05198 | 58 | GC14P067359 | 0.297258704900742 | https://www.genecards.org/cgi-bin/carddisp.pl?gene=EIF2S1 |
| KLF5 | KLF Transcription Factor 5 | Protein Coding | Q13887 | 58 | GC13P073054 | 0.297258704900742 | https://www.genecards.org/cgi-bin/carddisp.pl?gene=KLF5 |
| STEAP3 | STEAP3 Metalloreductase | Protein Coding | Q658P3 | 58 | GC02P154174 | 0.297258704900742 | https://www.genecards.org/cgi-bin/carddisp.pl?gene=STEAP3 |
| CRY1 | Cryptochrome Circadian Regulator 1 | Protein Coding | Q16526 | 57 | GC12M106991 | 0.297258704900742 | https://www.genecards.org/cgi-bin/carddisp.pl?gene=CRY1 |
| EEF1A1 | Eukaryotic Translation Elongation Factor 1 Alpha 1 | Protein Coding | P68104 | 57 | GC06M107761 | 0.297258704900742 | https://www.genecards.org/cgi-bin/carddisp.pl?gene=EEF1A1 |
| IGF2BP2 | Insulin Like Growth Factor 2 MRNA Binding Protein 2 | Protein Coding | Q9Y6M1 | 57 | GC03M185643 | 0.297258704900742 | https://www.genecards.org/cgi-bin/carddisp.pl?gene=IGF2BP2 |
| IL23R | Interleukin 23 Receptor | Protein Coding | Q5VWK5 | 57 | GC01P067138 | 0.297258704900742 | https://www.genecards.org/cgi-bin/carddisp.pl?gene=IL23R |
| UQCRB | Ubiquinol-Cytochrome C Reductase Binding Protein | Protein Coding | P14927 | 57 | GC08M096225 | 0.297258704900742 | https://www.genecards.org/cgi-bin/carddisp.pl?gene=UQCRB |
| ATN1 | Atrophin 1 | Protein Coding | P54259 | 56 | GC12P074081 | 0.297258704900742 | https://www.genecards.org/cgi-bin/carddisp.pl?gene=ATN1 |
| G6PC1 | Glucose-6-Phosphatase Catalytic Subunit 1 | Protein Coding | P35575 | 56 | GC17P156244 | 0.297258704900742 | https://www.genecards.org/cgi-bin/carddisp.pl?gene=G6PC1 |
| KRT10 | Keratin 10 | Protein Coding | P13645 | 56 | GC17M040818 | 0.297258704900742 | https://www.genecards.org/cgi-bin/carddisp.pl?gene=KRT10 |
| NCOR2 | Nuclear Receptor Corepressor 2 | Protein Coding | Q9Y618 | 56 | GC12M124324 | 0.297258704900742 | https://www.genecards.org/cgi-bin/carddisp.pl?gene=NCOR2 |
| PDCD1LG2 | Programmed Cell Death 1 Ligand 2 | Protein Coding | Q9BQ51 | 56 | GC09P005510 | 0.297258704900742 | https://www.genecards.org/cgi-bin/carddisp.pl?gene=PDCD1LG2 |
| ASPH | Aspartate Beta-Hydroxylase | Protein Coding | Q12797 | 55 | GC08M061500 | 0.297258704900742 | https://www.genecards.org/cgi-bin/carddisp.pl?gene=ASPH |
| CAV2 | Caveolin 2 | Protein Coding | P51636 | 55 | GC07P116287 | 0.297258704900742 | https://www.genecards.org/cgi-bin/carddisp.pl?gene=CAV2 |
| CPA1 | Carboxypeptidase A1 | Protein Coding | P15085 | 55 | GC07P130380 | 0.297258704900742 | https://www.genecards.org/cgi-bin/carddisp.pl?gene=CPA1 |
| TFEB | Transcription Factor EB | Protein Coding | P19484 | 55 | GC06M106693 | 0.297258704900742 | https://www.genecards.org/cgi-bin/carddisp.pl?gene=TFEB |
| ARHGEF4 | Rho Guanine Nucleotide Exchange Factor 4 | Protein Coding | Q9NR80 | 54 | GC02P130836 | 0.297258704900742 | https://www.genecards.org/cgi-bin/carddisp.pl?gene=ARHGEF4 |
| NMBR | Neuromedin B Receptor | Protein Coding | P28336 | 54 | GC06M142059 | 0.297258704900742 | https://www.genecards.org/cgi-bin/carddisp.pl?gene=NMBR |
| PLXNA2 | Plexin A2 | Protein Coding | O75051 | 54 | GC01M208023 | 0.297258704900742 | https://www.genecards.org/cgi-bin/carddisp.pl?gene=PLXNA2 |
| PTTG1 | PTTG1 Regulator Of Sister Chromatid Separation, Securin | Protein Coding | O95997 | 54 | GC05P160930 | 0.297258704900742 | https://www.genecards.org/cgi-bin/carddisp.pl?gene=PTTG1 |
| HNRNPF | Heterogeneous Nuclear Ribonucleoprotein F | Protein Coding | P52597 | 53 | GC10M043385 | 0.297258704900742 | https://www.genecards.org/cgi-bin/carddisp.pl?gene=HNRNPF |
| CDKAL1 | CDKAL1 Threonylcarbamoyladenosine TRNA Methylthiotransferase | Protein Coding | Q5VV42 | 52 | GC06P020534 | 0.297258704900742 | https://www.genecards.org/cgi-bin/carddisp.pl?gene=CDKAL1 |
| HHEX | Hematopoietically Expressed Homeobox | Protein Coding | Q03014 | 52 | GC10P092689 | 0.297258704900742 | https://www.genecards.org/cgi-bin/carddisp.pl?gene=HHEX |
| TOMM40 | Translocase Of Outer Mitochondrial Membrane 40 | Protein Coding | O96008 | 52 | GC19P044890 | 0.297258704900742 | https://www.genecards.org/cgi-bin/carddisp.pl?gene=TOMM40 |
| USP4 | Ubiquitin Specific Peptidase 4 | Protein Coding | Q13107 | 52 | GC03M049277 | 0.297258704900742 | https://www.genecards.org/cgi-bin/carddisp.pl?gene=USP4 |
| ARVCF | ARVCF Delta Catenin Family Member | Protein Coding | O00192 | 51 | GC22M085610 | 0.297258704900742 | https://www.genecards.org/cgi-bin/carddisp.pl?gene=ARVCF |
| EIF5B | Eukaryotic Translation Initiation Factor 5B | Protein Coding | O60841 | 51 | GC02P099337 | 0.297258704900742 | https://www.genecards.org/cgi-bin/carddisp.pl?gene=EIF5B |
| GPR39 | G Protein-Coupled Receptor 39 | Protein Coding | O43194 | 51 | GC02P154498 | 0.297258704900742 | https://www.genecards.org/cgi-bin/carddisp.pl?gene=GPR39 |
| IVL | Involucrin | Protein Coding | P07476 | 51 | GC01P176624 | 0.297258704900742 | https://www.genecards.org/cgi-bin/carddisp.pl?gene=IVL |
| PAEP | Progestagen Associated Endometrial Protein | Protein Coding | P09466 | 51 | GC09P135561 | 0.297258704900742 | https://www.genecards.org/cgi-bin/carddisp.pl?gene=PAEP |
| UBE2E2 | Ubiquitin Conjugating Enzyme E2 E2 | Protein Coding | Q96LR5 | 51 | GC03P027275 | 0.297258704900742 | https://www.genecards.org/cgi-bin/carddisp.pl?gene=UBE2E2 |
| VGF | VGF Nerve Growth Factor Inducible | Protein Coding | O15240 | 51 | GC07M101162 | 0.297258704900742 | https://www.genecards.org/cgi-bin/carddisp.pl?gene=VGF |
| DUSP9 | Dual Specificity Phosphatase 9 | Protein Coding | Q99956 | 50 | GC0XP153642 | 0.297258704900742 | https://www.genecards.org/cgi-bin/carddisp.pl?gene=DUSP9 |
| SLC16A4 | Solute Carrier Family 16 Member 4 | Protein Coding | O15374 | 50 | GC01M110362 | 0.297258704900742 | https://www.genecards.org/cgi-bin/carddisp.pl?gene=SLC16A4 |
| MAEA | Macrophage Erythroblast Attacher, E3 Ubiquitin Ligase | Protein Coding | Q7L5Y9 | 49 | GC04P001289 | 0.297258704900742 | https://www.genecards.org/cgi-bin/carddisp.pl?gene=MAEA |
| SH3PXD2A | SH3 And PX Domains 2A | Protein Coding | Q5TCZ1 | 48 | GC10M103594 | 0.297258704900742 | https://www.genecards.org/cgi-bin/carddisp.pl?gene=SH3PXD2A |
| AP3S2 | Adaptor Related Protein Complex 3 Subunit Sigma 2 | Protein Coding | P59780 | 46 | GC15M163241 | 0.297258704900742 | https://www.genecards.org/cgi-bin/carddisp.pl?gene=AP3S2 |
| GPSM1 | G Protein Signaling Modulator 1 | Protein Coding | Q86YR5 | 45 | GC09P136327 | 0.297258704900742 | https://www.genecards.org/cgi-bin/carddisp.pl?gene=GPSM1 |
| C2CD4A | C2 Calcium Dependent Domain Containing 4A | Protein Coding | Q8NCU7 | 41 | GC15P062066 | 0.297258704900742 | https://www.genecards.org/cgi-bin/carddisp.pl?gene=C2CD4A |
| C2CD4B | C2 Calcium Dependent Domain Containing 4B | Protein Coding | A6NLJ0 | 40 | GC15M162526 | 0.297258704900742 | https://www.genecards.org/cgi-bin/carddisp.pl?gene=C2CD4B |
| CPXCR1 | CPX Chromosome Region Candidate 1 | Protein Coding | Q8N123 | 40 | GC0XP088747 | 0.297258704900742 | https://www.genecards.org/cgi-bin/carddisp.pl?gene=CPXCR1 |
| SLC16A13 | Solute Carrier Family 16 Member 13 | Protein Coding | Q7RTY0 | 40 | GC17P007036 | 0.297258704900742 | https://www.genecards.org/cgi-bin/carddisp.pl?gene=SLC16A13 |
| MEG3 | Maternally Expressed 3 | RNA Gene |  | 37 | GC14P121348 | 0.297258704900742 | https://www.genecards.org/cgi-bin/carddisp.pl?gene=MEG3 |
| ARPIN-AP3S2 | ARPIN-AP3S2 Readthrough | Protein Coding |  | 30 | GC15M163242 | 0.297258704900742 | https://www.genecards.org/cgi-bin/carddisp.pl?gene=ARPIN-AP3S2 |
| GNAS-AS1 | GNAS Antisense RNA 1 | RNA Gene |  | 30 | GC20M058811 | 0.297258704900742 | https://www.genecards.org/cgi-bin/carddisp.pl?gene=GNAS-AS1 |
| MIR200B | MicroRNA 200b | RNA Gene |  | 30 | GC01P001167 | 0.297258704900742 | https://www.genecards.org/cgi-bin/carddisp.pl?gene=MIR200B |
| MIR128-1 | MicroRNA 128-1 | RNA Gene |  | 27 | GC02P135665 | 0.297258704900742 | https://www.genecards.org/cgi-bin/carddisp.pl?gene=MIR128-1 |
| TTN-AS1 | TTN Antisense RNA 1 | RNA Gene |  | 26 | GC02P178521 | 0.297258704900742 | https://www.genecards.org/cgi-bin/carddisp.pl?gene=TTN-AS1 |
| MIR487A | MicroRNA 487a | RNA Gene |  | 22 | GC14P121380 | 0.297258704900742 | https://www.genecards.org/cgi-bin/carddisp.pl?gene=MIR487A |
| LOC654780 | Splicing Factor Proline/Glutamine-Rich | RNA Gene |  | 20 | GC16M084193 | 0.297258704900742 | https://www.genecards.org/cgi-bin/carddisp.pl?gene=LOC654780 |
| MIR544A | MicroRNA 544a | RNA Gene |  | 17 | GC14P121389 | 0.297258704900742 | https://www.genecards.org/cgi-bin/carddisp.pl?gene=MIR544A |
| LOC110386948 | CYP19A1 Promoter I.4 | Functional Element |  | 5 | GC15P193122 | 0.297258704900742 | https://www.genecards.org/cgi-bin/carddisp.pl?gene=LOC110386948 |
| LDHA | Lactate Dehydrogenase A | Protein Coding | P00338 | 64 | GC11P018394 | 0.284380733966827 | https://www.genecards.org/cgi-bin/carddisp.pl?gene=LDHA |
| COL3A1 | Collagen Type III Alpha 1 Chain | Protein Coding | P02461 | 61 | GC02P188974 | 0.284380733966827 | https://www.genecards.org/cgi-bin/carddisp.pl?gene=COL3A1 |
| F5 | Coagulation Factor V | Protein Coding | P12259 | 60 | GC01M169511 | 0.284380733966827 | https://www.genecards.org/cgi-bin/carddisp.pl?gene=F5 |
| OXTR | Oxytocin Receptor | Protein Coding | P30559 | 60 | GC03M008767 | 0.284380733966827 | https://www.genecards.org/cgi-bin/carddisp.pl?gene=OXTR |
| ABCC8 | ATP Binding Cassette Subfamily C Member 8 | Protein Coding | Q09428 | 59 | GC11M017392 | 0.284380733966827 | https://www.genecards.org/cgi-bin/carddisp.pl?gene=ABCC8 |
| CD14 | CD14 Molecule | Protein Coding | P08571 | 58 | GC05M140631 | 0.284380733966827 | https://www.genecards.org/cgi-bin/carddisp.pl?gene=CD14 |
| FOXM1 | Forkhead Box M1 | Protein Coding | Q08050 | 56 | GC12M002857 | 0.284380733966827 | https://www.genecards.org/cgi-bin/carddisp.pl?gene=FOXM1 |
| GTF2H1 | General Transcription Factor IIH Subunit 1 | Protein Coding | P32780 | 54 | GC11P021408 | 0.284380733966827 | https://www.genecards.org/cgi-bin/carddisp.pl?gene=GTF2H1 |
| PIAS3 | Protein Inhibitor Of Activated STAT 3 | Protein Coding | Q9Y6X2 | 54 | GC01M145848 | 0.284380733966827 | https://www.genecards.org/cgi-bin/carddisp.pl?gene=PIAS3 |
| LDHC | Lactate Dehydrogenase C | Protein Coding | P07864 | 52 | GC11P021410 | 0.284380733966827 | https://www.genecards.org/cgi-bin/carddisp.pl?gene=LDHC |
| HPS5 | HPS5 Biogenesis Of Lysosomal Organelles Complex 2 Subunit 2 | Protein Coding | Q9UPZ3 | 51 | GC11M018278 | 0.284380733966827 | https://www.genecards.org/cgi-bin/carddisp.pl?gene=HPS5 |
| LOC126806446 | P300/CBP Strongly-Dependent Group 1 Enhancer GRCh37_chr2:189866210-189867409 | Functional Element |  | 7 | GC02P189002 | 0.284380733966827 | https://www.genecards.org/cgi-bin/carddisp.pl?gene=LOC126806446 |
| TLR2 | Toll Like Receptor 2 | Protein Coding | O60603 | 65 | GC04P153684 | 0.246280923485756 | https://www.genecards.org/cgi-bin/carddisp.pl?gene=TLR2 |
| HNF4A | Hepatocyte Nuclear Factor 4 Alpha | Protein Coding | P41235 | 62 | GC20P044355 | 0.246280923485756 | https://www.genecards.org/cgi-bin/carddisp.pl?gene=HNF4A |
| TNFAIP3 | TNF Alpha Induced Protein 3 | Protein Coding | P21580 | 62 | GC06P137866 | 0.246280923485756 | https://www.genecards.org/cgi-bin/carddisp.pl?gene=TNFAIP3 |
| AHR | Aryl Hydrocarbon Receptor | Protein Coding | P35869 | 61 | GC07P016916 | 0.246280923485756 | https://www.genecards.org/cgi-bin/carddisp.pl?gene=AHR |
| GK | Glycerol Kinase | Protein Coding | P32189 | 59 | GC0XP031646 | 0.246280923485756 | https://www.genecards.org/cgi-bin/carddisp.pl?gene=GK |
| ACSS2 | Acyl-CoA Synthetase Short Chain Family Member 2 | Protein Coding | Q9NR19 | 58 | GC20P053239 | 0.246280923485756 | https://www.genecards.org/cgi-bin/carddisp.pl?gene=ACSS2 |
| CYP2E1 | Cytochrome P450 Family 2 Subfamily E Member 1 | Protein Coding | P05181 | 58 | GC10P133520 | 0.246280923485756 | https://www.genecards.org/cgi-bin/carddisp.pl?gene=CYP2E1 |
| HMGA2 | High Mobility Group AT-Hook 2 | Protein Coding | P52926 | 58 | GC12P065824 | 0.246280923485756 | https://www.genecards.org/cgi-bin/carddisp.pl?gene=HMGA2 |
| ALPP | Alkaline Phosphatase, Placental | Protein Coding | P05187 | 57 | GC02P232378 | 0.246280923485756 | https://www.genecards.org/cgi-bin/carddisp.pl?gene=ALPP |
| GAS6 | Growth Arrest Specific 6 | Protein Coding | Q14393 | 56 | GC13M113820 | 0.246280923485756 | https://www.genecards.org/cgi-bin/carddisp.pl?gene=GAS6 |
| SFRP1 | Secreted Frizzled Related Protein 1 | Protein Coding | Q8N474 | 55 | GC08M041262 | 0.246280923485756 | https://www.genecards.org/cgi-bin/carddisp.pl?gene=SFRP1 |
| THPO | Thrombopoietin | Protein Coding | P40225 | 55 | GC03M184371 | 0.246280923485756 | https://www.genecards.org/cgi-bin/carddisp.pl?gene=THPO |
| LGALS3BP | Galectin 3 Binding Protein | Protein Coding | Q08380 | 54 | GC17M078971 | 0.246280923485756 | https://www.genecards.org/cgi-bin/carddisp.pl?gene=LGALS3BP |
| TRIM37 | Tripartite Motif Containing 37 | Protein Coding | O94972 | 54 | GC17M097706 | 0.246280923485756 | https://www.genecards.org/cgi-bin/carddisp.pl?gene=TRIM37 |
| KLF2 | KLF Transcription Factor 2 | Protein Coding | Q9Y5W3 | 53 | GC19P152062 | 0.246280923485756 | https://www.genecards.org/cgi-bin/carddisp.pl?gene=KLF2 |
| PRKAG3 | Protein Kinase AMP-Activated Non-Catalytic Subunit Gamma 3 | Protein Coding | Q9UGI9 | 53 | GC02M218823 | 0.246280923485756 | https://www.genecards.org/cgi-bin/carddisp.pl?gene=PRKAG3 |
| KLRK1 | Killer Cell Lectin Like Receptor K1 | Protein Coding | P26718 | 52 | GC12M035797 | 0.246280923485756 | https://www.genecards.org/cgi-bin/carddisp.pl?gene=KLRK1 |
| LHB | Luteinizing Hormone Subunit Beta | Protein Coding | P01229 | 52 | GC19M049015 | 0.246280923485756 | https://www.genecards.org/cgi-bin/carddisp.pl?gene=LHB |
| SPG11 | SPG11 Vesicle Trafficking Associated, Spatacsin | Protein Coding | Q96JI7 | 52 | GC15M048180 | 0.246280923485756 | https://www.genecards.org/cgi-bin/carddisp.pl?gene=SPG11 |
| STC1 | Stanniocalcin 1 | Protein Coding | P52823 | 52 | GC08M023841 | 0.246280923485756 | https://www.genecards.org/cgi-bin/carddisp.pl?gene=STC1 |
| PLAG1 | PLAG1 Zinc Finger | Protein Coding | Q6DJT9 | 50 | GC08M056798 | 0.246280923485756 | https://www.genecards.org/cgi-bin/carddisp.pl?gene=PLAG1 |
| PNOC | Prepronociceptin | Protein Coding | Q13519 | 50 | GC08P028316 | 0.246280923485756 | https://www.genecards.org/cgi-bin/carddisp.pl?gene=PNOC |
| PLA2G15 | Phospholipase A2 Group XV | Protein Coding | Q8NCC3 | 49 | GC16P068245 | 0.246280923485756 | https://www.genecards.org/cgi-bin/carddisp.pl?gene=PLA2G15 |
| GCM1 | Glial Cells Missing Transcription Factor 1 | Protein Coding | Q9NP62 | 47 | GC06M106890 | 0.246280923485756 | https://www.genecards.org/cgi-bin/carddisp.pl?gene=GCM1 |
| C1QTNF12 | C1q And TNF Related 12 | Protein Coding | Q5T7M4 | 40 | GC01M022159 | 0.246280923485756 | https://www.genecards.org/cgi-bin/carddisp.pl?gene=C1QTNF12 |
| MIRLET7C | MicroRNA Let-7c | RNA Gene |  | 31 | GC21P021313 | 0.246280923485756 | https://www.genecards.org/cgi-bin/carddisp.pl?gene=MIRLET7C |
| MIR155 | MicroRNA 155 | RNA Gene |  | 30 | GC21P025573 | 0.246280923485756 | https://www.genecards.org/cgi-bin/carddisp.pl?gene=MIR155 |
| MIR34C | MicroRNA 34c | RNA Gene |  | 30 | GC11P113442 | 0.246280923485756 | https://www.genecards.org/cgi-bin/carddisp.pl?gene=MIR34C |
| MIR483 | MicroRNA 483 | RNA Gene |  | 30 | GC11M015126 | 0.246280923485756 | https://www.genecards.org/cgi-bin/carddisp.pl?gene=MIR483 |
| MIR302A | MicroRNA 302a | RNA Gene |  | 28 | GC04M113185 | 0.246280923485756 | https://www.genecards.org/cgi-bin/carddisp.pl?gene=MIR302A |
| LOC109623489 | Insulin Repeat Instability Region | Functional Element |  | 5 | GC11P020312 | 0.246280923485756 | https://www.genecards.org/cgi-bin/carddisp.pl?gene=LOC109623489 |
| MET | MET Proto-Oncogene, Receptor Tyrosine Kinase | Protein Coding | P08581 | 67 | GC07P116672 | 0.201087534427643 | https://www.genecards.org/cgi-bin/carddisp.pl?gene=MET |
| AXL | AXL Receptor Tyrosine Kinase | Protein Coding | P30530 | 64 | GC19P041219 | 0.201087534427643 | https://www.genecards.org/cgi-bin/carddisp.pl?gene=AXL |
| GNAS | GNAS Complex Locus | Protein Coding | P84996 | 63 | GC20P058839 | 0.201087534427643 | https://www.genecards.org/cgi-bin/carddisp.pl?gene=GNAS |
| PLA2G7 | Phospholipase A2 Group VII | Protein Coding | Q13093 | 63 | GC06M046704 | 0.201087534427643 | https://www.genecards.org/cgi-bin/carddisp.pl?gene=PLA2G7 |
| ITGB1 | Integrin Subunit Beta 1 | Protein Coding | P05556 | 62 | GC10M037195 | 0.201087534427643 | https://www.genecards.org/cgi-bin/carddisp.pl?gene=ITGB1 |
| MAOA | Monoamine Oxidase A | Protein Coding | P21397 | 62 | GC0XP043654 | 0.201087534427643 | https://www.genecards.org/cgi-bin/carddisp.pl?gene=MAOA |
| ATP7B | ATPase Copper Transporting Beta | Protein Coding | P35670 | 61 | GC13M051930 | 0.201087534427643 | https://www.genecards.org/cgi-bin/carddisp.pl?gene=ATP7B |
| BMPR1B | Bone Morphogenetic Protein Receptor Type 1B | Protein Coding | O00238 | 61 | GC04P094757 | 0.201087534427643 | https://www.genecards.org/cgi-bin/carddisp.pl?gene=BMPR1B |
| FASLG | Fas Ligand | Protein Coding | P48023 | 61 | GC01P172659 | 0.201087534427643 | https://www.genecards.org/cgi-bin/carddisp.pl?gene=FASLG |
| KNG1 | Kininogen 1 | Protein Coding | P01042 | 61 | GC03P186717 | 0.201087534427643 | https://www.genecards.org/cgi-bin/carddisp.pl?gene=KNG1 |
| TRPV4 | Transient Receptor Potential Cation Channel Subfamily V Member 4 | Protein Coding | Q9HBA0 | 61 | GC12M109783 | 0.201087534427643 | https://www.genecards.org/cgi-bin/carddisp.pl?gene=TRPV4 |
| ARSA | Arylsulfatase A | Protein Coding | P15289 | 60 | GC22M050622 | 0.201087534427643 | https://www.genecards.org/cgi-bin/carddisp.pl?gene=ARSA |
| CAMKK2 | Calcium/Calmodulin Dependent Protein Kinase Kinase 2 | Protein Coding | Q96RR4 | 60 | GC12M124682 | 0.201087534427643 | https://www.genecards.org/cgi-bin/carddisp.pl?gene=CAMKK2 |
| CUL3 | Cullin 3 | Protein Coding | Q13618 | 60 | GC02M224470 | 0.201087534427643 | https://www.genecards.org/cgi-bin/carddisp.pl?gene=CUL3 |
| GNAO1 | G Protein Subunit Alpha O1 | Protein Coding | P09471 | 60 | GC16P121581 | 0.201087534427643 | https://www.genecards.org/cgi-bin/carddisp.pl?gene=GNAO1 |
| GOT2 | Glutamic-Oxaloacetic Transaminase 2 | Protein Coding | P00505 | 60 | GC16M058707 | 0.201087534427643 | https://www.genecards.org/cgi-bin/carddisp.pl?gene=GOT2 |
| HSPA1A | Heat Shock Protein Family A (Hsp70) Member 1A | Protein Coding | P0DMV8 | 60 | GC06P181910 | 0.201087534427643 | https://www.genecards.org/cgi-bin/carddisp.pl?gene=HSPA1A |
| SLC5A2 | Solute Carrier Family 5 Member 2 | Protein Coding | P31639 | 60 | GC16P121110 | 0.201087534427643 | https://www.genecards.org/cgi-bin/carddisp.pl?gene=SLC5A2 |
| SSTR2 | Somatostatin Receptor 2 | Protein Coding | P30874 | 60 | GC17P073165 | 0.201087534427643 | https://www.genecards.org/cgi-bin/carddisp.pl?gene=SSTR2 |
| TGM2 | Transglutaminase 2 | Protein Coding | P21980 | 60 | GC20M038127 | 0.201087534427643 | https://www.genecards.org/cgi-bin/carddisp.pl?gene=TGM2 |
| ACADVL | Acyl-CoA Dehydrogenase Very Long Chain | Protein Coding | P49748 | 59 | GC17P153721 | 0.201087534427643 | https://www.genecards.org/cgi-bin/carddisp.pl?gene=ACADVL |
| GCGR | Glucagon Receptor | Protein Coding | P47871 | 59 | GC17P081804 | 0.201087534427643 | https://www.genecards.org/cgi-bin/carddisp.pl?gene=GCGR |
| HADHA | Hydroxyacyl-CoA Dehydrogenase Trifunctional Multienzyme Complex Subunit Alpha | Protein Coding | P40939 | 59 | GC02M026190 | 0.201087534427643 | https://www.genecards.org/cgi-bin/carddisp.pl?gene=HADHA |
| HTR1A | 5-Hydroxytryptamine Receptor 1A | Protein Coding | P08908 | 59 | GC05M063960 | 0.201087534427643 | https://www.genecards.org/cgi-bin/carddisp.pl?gene=HTR1A |
| PTPN2 | Protein Tyrosine Phosphatase Non-Receptor Type 2 | Protein Coding | P17706 | 59 | GC18M038212 | 0.201087534427643 | https://www.genecards.org/cgi-bin/carddisp.pl?gene=PTPN2 |
| PTPN22 | Protein Tyrosine Phosphatase Non-Receptor Type 22 | Protein Coding | Q9Y2R2 | 59 | GC01M113813 | 0.201087534427643 | https://www.genecards.org/cgi-bin/carddisp.pl?gene=PTPN22 |
| SLCO1B1 | Solute Carrier Organic Anion Transporter Family Member 1B1 | Protein Coding | Q9Y6L6 | 59 | GC12P074424 | 0.201087534427643 | https://www.genecards.org/cgi-bin/carddisp.pl?gene=SLCO1B1 |
| AQP4 | Aquaporin 4 | Protein Coding | P55087 | 58 | GC18M026852 | 0.201087534427643 | https://www.genecards.org/cgi-bin/carddisp.pl?gene=AQP4 |
| CYSLTR2 | Cysteinyl Leukotriene Receptor 2 | Protein Coding | Q9NS75 | 58 | GC13P048653 | 0.201087534427643 | https://www.genecards.org/cgi-bin/carddisp.pl?gene=CYSLTR2 |
| GABRA6 | Gamma-Aminobutyric Acid Type A Receptor Subunit Alpha6 | Protein Coding | Q16445 | 58 | GC05P161547 | 0.201087534427643 | https://www.genecards.org/cgi-bin/carddisp.pl?gene=GABRA6 |
| GUCY2C | Guanylate Cyclase 2C | Protein Coding | P25092 | 58 | GC12M014612 | 0.201087534427643 | https://www.genecards.org/cgi-bin/carddisp.pl?gene=GUCY2C |
| PKD1 | Polycystin 1, Transient Receptor Potential Channel Interacting | Protein Coding | P98161 | 58 | GC16M049832 | 0.201087534427643 | https://www.genecards.org/cgi-bin/carddisp.pl?gene=PKD1 |
| SLC6A6 | Solute Carrier Family 6 Member 6 | Protein Coding | P31641 | 58 | GC03P014402 | 0.201087534427643 | https://www.genecards.org/cgi-bin/carddisp.pl?gene=SLC6A6 |
| GIPR | Gastric Inhibitory Polypeptide Receptor | Protein Coding | P48546 | 57 | GC19P045668 | 0.201087534427643 | https://www.genecards.org/cgi-bin/carddisp.pl?gene=GIPR |
| MSX2 | Msh Homeobox 2 | Protein Coding | P35548 | 57 | GC05P174724 | 0.201087534427643 | https://www.genecards.org/cgi-bin/carddisp.pl?gene=MSX2 |
| PCSK2 | Proprotein Convertase Subtilisin/Kexin Type 2 | Protein Coding | P16519 | 57 | GC20P017226 | 0.201087534427643 | https://www.genecards.org/cgi-bin/carddisp.pl?gene=PCSK2 |
| PON3 | Paraoxonase 3 | Protein Coding | Q15166 | 57 | GC07M095359 | 0.201087534427643 | https://www.genecards.org/cgi-bin/carddisp.pl?gene=PON3 |
| AOX1 | Aldehyde Oxidase 1 | Protein Coding | Q06278 | 56 | GC02P200585 | 0.201087534427643 | https://www.genecards.org/cgi-bin/carddisp.pl?gene=AOX1 |
| BCAR1 | BCAR1 Scaffold Protein, Cas Family Member | Protein Coding | P56945 | 56 | GC16M075228 | 0.201087534427643 | https://www.genecards.org/cgi-bin/carddisp.pl?gene=BCAR1 |
| SLC22A2 | Solute Carrier Family 22 Member 2 | Protein Coding | O15244 | 56 | GC06M160232 | 0.201087534427643 | https://www.genecards.org/cgi-bin/carddisp.pl?gene=SLC22A2 |
| SULT2B1 | Sulfotransferase Family 2B Member 1 | Protein Coding | O00204 | 56 | GC19P048552 | 0.201087534427643 | https://www.genecards.org/cgi-bin/carddisp.pl?gene=SULT2B1 |
| GRB14 | Growth Factor Receptor Bound Protein 14 | Protein Coding | Q14449 | 55 | GC02M164492 | 0.201087534427643 | https://www.genecards.org/cgi-bin/carddisp.pl?gene=GRB14 |
| HSD3B1 | Hydroxy-Delta-5-Steroid Dehydrogenase, 3 Beta- And Steroid Delta-Isomerase 1 | Protein Coding | P14060 | 55 | GC01P119507 | 0.201087534427643 | https://www.genecards.org/cgi-bin/carddisp.pl?gene=HSD3B1 |
| LOXL1 | Lysyl Oxidase Like 1 | Protein Coding | Q08397 | 55 | GC15P073925 | 0.201087534427643 | https://www.genecards.org/cgi-bin/carddisp.pl?gene=LOXL1 |
| PTPN3 | Protein Tyrosine Phosphatase Non-Receptor Type 3 | Protein Coding | P26045 | 55 | GC09M109375 | 0.201087534427643 | https://www.genecards.org/cgi-bin/carddisp.pl?gene=PTPN3 |
| SLC22A1 | Solute Carrier Family 22 Member 1 | Protein Coding | O15245 | 55 | GC06P160121 | 0.201087534427643 | https://www.genecards.org/cgi-bin/carddisp.pl?gene=SLC22A1 |
| TNFRSF12A | TNF Receptor Superfamily Member 12A | Protein Coding | Q9NP84 | 55 | GC16P003018 | 0.201087534427643 | https://www.genecards.org/cgi-bin/carddisp.pl?gene=TNFRSF12A |
| CYP7A1 | Cytochrome P450 Family 7 Subfamily A Member 1 | Protein Coding | P22680 | 54 | GC08M058490 | 0.201087534427643 | https://www.genecards.org/cgi-bin/carddisp.pl?gene=CYP7A1 |
| IL1RL1 | Interleukin 1 Receptor Like 1 | Protein Coding | Q01638 | 54 | GC02P102294 | 0.201087534427643 | https://www.genecards.org/cgi-bin/carddisp.pl?gene=IL1RL1 |
| PTAFR | Platelet Activating Factor Receptor | Protein Coding | P25105 | 54 | GC01M028147 | 0.201087534427643 | https://www.genecards.org/cgi-bin/carddisp.pl?gene=PTAFR |
| IL33 | Interleukin 33 | Protein Coding | O95760 | 53 | GC09P010645 | 0.201087534427643 | https://www.genecards.org/cgi-bin/carddisp.pl?gene=IL33 |
| NDUFB3 | NADH:Ubiquinone Oxidoreductase Subunit B3 | Protein Coding | O43676 | 53 | GC02P201071 | 0.201087534427643 | https://www.genecards.org/cgi-bin/carddisp.pl?gene=NDUFB3 |
| SLC47A1 | Solute Carrier Family 47 Member 1 | Protein Coding | Q96FL8 | 52 | GC17P019495 | 0.201087534427643 | https://www.genecards.org/cgi-bin/carddisp.pl?gene=SLC47A1 |
| TTC8 | Tetratricopeptide Repeat Domain 8 | Protein Coding | Q8TAM2 | 52 | GC14P095128 | 0.201087534427643 | https://www.genecards.org/cgi-bin/carddisp.pl?gene=TTC8 |
| KLRB1 | Killer Cell Lectin Like Receptor B1 | Protein Coding | Q12918 | 51 | GC12M035788 | 0.201087534427643 | https://www.genecards.org/cgi-bin/carddisp.pl?gene=KLRB1 |
| SLC2A12 | Solute Carrier Family 2 Member 12 | Protein Coding | Q8TD20 | 51 | GC06M133987 | 0.201087534427643 | https://www.genecards.org/cgi-bin/carddisp.pl?gene=SLC2A12 |
| FFAR4 | Free Fatty Acid Receptor 4 | Protein Coding | Q5NUL3 | 50 | GC10P093566 | 0.201087534427643 | https://www.genecards.org/cgi-bin/carddisp.pl?gene=FFAR4 |
| NR2F6 | Nuclear Receptor Subfamily 2 Group F Member 6 | Protein Coding | P10588 | 50 | GC19M017231 | 0.201087534427643 | https://www.genecards.org/cgi-bin/carddisp.pl?gene=NR2F6 |
| PSG1 | Pregnancy Specific Beta-1-Glycoprotein 1 | Protein Coding | P11464 | 50 | GC19M042866 | 0.201087534427643 | https://www.genecards.org/cgi-bin/carddisp.pl?gene=PSG1 |
| CCL25 | C-C Motif Chemokine Ligand 25 | Protein Coding | O15444 | 49 | GC19P008052 | 0.201087534427643 | https://www.genecards.org/cgi-bin/carddisp.pl?gene=CCL25 |
| PRMT2 | Protein Arginine Methyltransferase 2 | Protein Coding | P55345 | 48 | GC21P046635 | 0.201087534427643 | https://www.genecards.org/cgi-bin/carddisp.pl?gene=PRMT2 |
| FAM3B | FAM3 Metabolism Regulating Signaling Molecule B | Protein Coding | P58499 | 47 | GC21P041304 | 0.201087534427643 | https://www.genecards.org/cgi-bin/carddisp.pl?gene=FAM3B |
| MCHR2 | Melanin Concentrating Hormone Receptor 2 | Protein Coding | Q969V1 | 47 | GC06M108596 | 0.201087534427643 | https://www.genecards.org/cgi-bin/carddisp.pl?gene=MCHR2 |
| NMUR2 | Neuromedin U Receptor 2 | Protein Coding | Q9GZQ4 | 47 | GC05M152391 | 0.201087534427643 | https://www.genecards.org/cgi-bin/carddisp.pl?gene=NMUR2 |
| UCN3 | Urocortin 3 | Protein Coding | Q969E3 | 47 | GC10P005364 | 0.201087534427643 | https://www.genecards.org/cgi-bin/carddisp.pl?gene=UCN3 |
| XCL1 | X-C Motif Chemokine Ligand 1 | Protein Coding | P47992 | 47 | GC01P168576 | 0.201087534427643 | https://www.genecards.org/cgi-bin/carddisp.pl?gene=XCL1 |
| KBTBD2 | Kelch Repeat And BTB Domain Containing 2 | Protein Coding | Q8IY47 | 43 | GC07M032868 | 0.201087534427643 | https://www.genecards.org/cgi-bin/carddisp.pl?gene=KBTBD2 |
| PAQR4 | Progestin And AdipoQ Receptor Family Member 4 | Protein Coding | Q8N4S7 | 43 | GC16P002969 | 0.201087534427643 | https://www.genecards.org/cgi-bin/carddisp.pl?gene=PAQR4 |
| GPR171 | G Protein-Coupled Receptor 171 | Protein Coding | O14626 | 42 | GC03M151197 | 0.201087534427643 | https://www.genecards.org/cgi-bin/carddisp.pl?gene=GPR171 |
| ASB4 | Ankyrin Repeat And SOCS Box Containing 4 | Protein Coding | Q9Y574 | 40 | GC07P096556 | 0.201087534427643 | https://www.genecards.org/cgi-bin/carddisp.pl?gene=ASB4 |
| C1QTNF4 | C1q And TNF Related 4 | Protein Coding | Q9BXJ3 | 40 | GC11M139459 | 0.201087534427643 | https://www.genecards.org/cgi-bin/carddisp.pl?gene=C1QTNF4 |
| MGARP | Mitochondria Localized Glutamic Acid Rich Protein | Protein Coding | Q8TDB4 | 38 | GC04M139266 | 0.201087534427643 | https://www.genecards.org/cgi-bin/carddisp.pl?gene=MGARP |
| MIR17 | MicroRNA 17 | RNA Gene |  | 28 | GC13P091350 | 0.201087534427643 | https://www.genecards.org/cgi-bin/carddisp.pl?gene=MIR17 |
| MIR193A | MicroRNA 193a | RNA Gene |  | 28 | GC17P031559 | 0.201087534427643 | https://www.genecards.org/cgi-bin/carddisp.pl?gene=MIR193A |
| MIR708 | MicroRNA 708 | RNA Gene |  | 28 | GC11M079402 | 0.201087534427643 | https://www.genecards.org/cgi-bin/carddisp.pl?gene=MIR708 |
| MIR379 | MicroRNA 379 | RNA Gene |  | 27 | GC14P121370 | 0.201087534427643 | https://www.genecards.org/cgi-bin/carddisp.pl?gene=MIR379 |
| SNORD15A | Small Nucleolar RNA, C/D Box 15A | RNA Gene |  | 25 | GC11P075400 | 0.201087534427643 | https://www.genecards.org/cgi-bin/carddisp.pl?gene=SNORD15A |
| BICDL3P | BICD Family Like 3, Pseudogene | Pseudogene |  | 24 | GC07P095781 | 0.201087534427643 | https://www.genecards.org/cgi-bin/carddisp.pl?gene=BICDL3P |
| HIF1A-AS2 | HIF1A Antisense RNA 2 | RNA Gene |  | 23 | GC14M061747 | 0.201087534427643 | https://www.genecards.org/cgi-bin/carddisp.pl?gene=HIF1A-AS2 |
| PWAR1 | Prader Willi/Angelman Region RNA 1 | RNA Gene |  | 22 | GC15P025135 | 0.201087534427643 | https://www.genecards.org/cgi-bin/carddisp.pl?gene=PWAR1 |
| MIR758 | MicroRNA 758 | RNA Gene |  | 21 | GC14P121395 | 0.201087534427643 | https://www.genecards.org/cgi-bin/carddisp.pl?gene=MIR758 |
| ZFP91-CNTF | ZFP91-CNTF Readthrough (NMD Candidate) | RNA Gene |  | 18 | GC11P058579 | 0.201087534427643 | https://www.genecards.org/cgi-bin/carddisp.pl?gene=ZFP91-CNTF |
| PWAR4 | Prader Willi/Angelman Region RNA 4 | RNA Gene |  | 17 | GC15P025211 | 0.201087534427643 | https://www.genecards.org/cgi-bin/carddisp.pl?gene=PWAR4 |
| LOC129931597 | ATAC-STARR-Seq Lymphoblastoid Silent Region 1421 | Functional Element |  | 10 | GC01P178835 | 0.201087534427643 | https://www.genecards.org/cgi-bin/carddisp.pl?gene=LOC129931597 |
| TRR-TCG4-1 | TRNA-Arg (Anticodon TCG) 4-1 | RNA Gene |  | 10 | GC06P181708 | 0.201087534427643 | https://www.genecards.org/cgi-bin/carddisp.pl?gene=TRR-TCG4-1 |
| TRR-TCT2-1 | TRNA-Arg (Anticodon TCT) 2-1 | RNA Gene |  | 10 | GC17P153780 | 0.201087534427643 | https://www.genecards.org/cgi-bin/carddisp.pl?gene=TRR-TCT2-1 |
| TRR-ACG1-2 | TRNA-Arg (Anticodon ACG) 1-2 | RNA Gene |  | 9 | GC06P190490 | 0.201087534427643 | https://www.genecards.org/cgi-bin/carddisp.pl?gene=TRR-ACG1-2 |
| LOC110121472 | VISTA Enhancer Hs1980 | Functional Element |  | 8 | GC10P112997 | 0.201087534427643 | https://www.genecards.org/cgi-bin/carddisp.pl?gene=LOC110121472 |
| LOC129933280 | ATAC-STARR-Seq Lymphoblastoid Silent Region 11244 | Functional Element |  | 8 | GC02P025161 | 0.201087534427643 | https://www.genecards.org/cgi-bin/carddisp.pl?gene=LOC129933280 |
| LOC106707174 | ERVW-1 Upstream Regulatory Region | Functional Element |  | 2 | GC07P095724 | 0.201087534427643 | https://www.genecards.org/cgi-bin/carddisp.pl?gene=LOC106707174 |
| DDX41 | DEAD-Box Helicase 41 | Protein Coding | Q9UJV9 | 56 | GC05M177511 | 0.191701024770737 | https://www.genecards.org/cgi-bin/carddisp.pl?gene=DDX41 |
| IRGM | Immunity Related GTPase M | Protein Coding | A1A4Y4 | 45 | GC05P150846 | 0.15652322769165 | https://www.genecards.org/cgi-bin/carddisp.pl?gene=IRGM |
| IRGQ | Immunity Related GTPase Q | Protein Coding | Q8WZA9 | 44 | GC19M043584 | 0.15652322769165 | https://www.genecards.org/cgi-bin/carddisp.pl?gene=IRGQ |
| CDK4 | Cyclin Dependent Kinase 4 | Protein Coding | P11802 | 67 | GC12M060483 | 0.142190366983414 | https://www.genecards.org/cgi-bin/carddisp.pl?gene=CDK4 |
| HDAC4 | Histone Deacetylase 4 | Protein Coding | P56524 | 65 | GC02M239048 | 0.142190366983414 | https://www.genecards.org/cgi-bin/carddisp.pl?gene=HDAC4 |
| NT5E | 5'-Nucleotidase Ecto | Protein Coding | P21589 | 65 | GC06P085449 | 0.142190366983414 | https://www.genecards.org/cgi-bin/carddisp.pl?gene=NT5E |
| ABCB1 | ATP Binding Cassette Subfamily B Member 1 | Protein Coding | P08183 | 64 | GC07M087504 | 0.142190366983414 | https://www.genecards.org/cgi-bin/carddisp.pl?gene=ABCB1 |
| FOS | Fos Proto-Oncogene, AP-1 Transcription Factor Subunit | Protein Coding | P01100 | 64 | GC14P075278 | 0.142190366983414 | https://www.genecards.org/cgi-bin/carddisp.pl?gene=FOS |
| GJA1 | Gap Junction Protein Alpha 1 | Protein Coding | P17302 | 64 | GC06P182951 | 0.142190366983414 | https://www.genecards.org/cgi-bin/carddisp.pl?gene=GJA1 |
| PARP1 | Poly(ADP-Ribose) Polymerase 1 | Protein Coding | P09874 | 64 | GC01M226360 | 0.142190366983414 | https://www.genecards.org/cgi-bin/carddisp.pl?gene=PARP1 |
| PDGFB | Platelet Derived Growth Factor Subunit B | Protein Coding | P01127 | 64 | GC22M086976 | 0.142190366983414 | https://www.genecards.org/cgi-bin/carddisp.pl?gene=PDGFB |
| KCNH2 | Potassium Voltage-Gated Channel Subfamily H Member 2 | Protein Coding | Q12809 | 63 | GC07M150944 | 0.142190366983414 | https://www.genecards.org/cgi-bin/carddisp.pl?gene=KCNH2 |
| TBXA2R | Thromboxane A2 Receptor | Protein Coding | P21731 | 63 | GC19M003594 | 0.142190366983414 | https://www.genecards.org/cgi-bin/carddisp.pl?gene=TBXA2R |
| TUBB3 | Tubulin Beta 3 Class III | Protein Coding | Q13509 | 63 | GC16P122964 | 0.142190366983414 | https://www.genecards.org/cgi-bin/carddisp.pl?gene=TUBB3 |
| ACVR2B | Activin A Receptor Type 2B | Protein Coding | Q13705 | 62 | GC03P038453 | 0.142190366983414 | https://www.genecards.org/cgi-bin/carddisp.pl?gene=ACVR2B |
| ATP1A1 | ATPase Na+/K+ Transporting Subunit Alpha 1 | Protein Coding | P05023 | 62 | GC01P116372 | 0.142190366983414 | https://www.genecards.org/cgi-bin/carddisp.pl?gene=ATP1A1 |
| CAMK2G | Calcium/Calmodulin Dependent Protein Kinase II Gamma | Protein Coding | Q13555 | 62 | GC10M073812 | 0.142190366983414 | https://www.genecards.org/cgi-bin/carddisp.pl?gene=CAMK2G |
| CTSK | Cathepsin K | Protein Coding | P43235 | 62 | GC01M167274 | 0.142190366983414 | https://www.genecards.org/cgi-bin/carddisp.pl?gene=CTSK |
| GATA4 | GATA Binding Protein 4 | Protein Coding | P43694 | 62 | GC08P011676 | 0.142190366983414 | https://www.genecards.org/cgi-bin/carddisp.pl?gene=GATA4 |
| ITGA4 | Integrin Subunit Alpha 4 | Protein Coding | P13612 | 62 | GC02P181501 | 0.142190366983414 | https://www.genecards.org/cgi-bin/carddisp.pl?gene=ITGA4 |
| NLRP3 | NLR Family Pyrin Domain Containing 3 | Protein Coding | Q96P20 | 62 | GC01P247632 | 0.142190366983414 | https://www.genecards.org/cgi-bin/carddisp.pl?gene=NLRP3 |
| PIM1 | Pim-1 Proto-Oncogene, Serine/Threonine Kinase | Protein Coding | P11309 | 62 | GC06P182028 | 0.142190366983414 | https://www.genecards.org/cgi-bin/carddisp.pl?gene=PIM1 |
| SQSTM1 | Sequestosome 1 | Protein Coding | Q13501 | 62 | GC05P179806 | 0.142190366983414 | https://www.genecards.org/cgi-bin/carddisp.pl?gene=SQSTM1 |
| TLR8 | Toll Like Receptor 8 | Protein Coding | Q9NR97 | 62 | GC0XP012924 | 0.142190366983414 | https://www.genecards.org/cgi-bin/carddisp.pl?gene=TLR8 |
| TNC | Tenascin C | Protein Coding | P24821 | 62 | GC09M115019 | 0.142190366983414 | https://www.genecards.org/cgi-bin/carddisp.pl?gene=TNC |
| ADCY5 | Adenylate Cyclase 5 | Protein Coding | O95622 | 61 | GC03M123282 | 0.142190366983414 | https://www.genecards.org/cgi-bin/carddisp.pl?gene=ADCY5 |
| CAPN3 | Calpain 3 | Protein Coding | P20807 | 61 | GC15P042359 | 0.142190366983414 | https://www.genecards.org/cgi-bin/carddisp.pl?gene=CAPN3 |
| CD44 | CD44 Molecule (IN Blood Group) | Protein Coding | P16070 | 61 | GC11P035139 | 0.142190366983414 | https://www.genecards.org/cgi-bin/carddisp.pl?gene=CD44 |
| CDKN1B | Cyclin Dependent Kinase Inhibitor 1B | Protein Coding | P46527 | 61 | GC12P074277 | 0.142190366983414 | https://www.genecards.org/cgi-bin/carddisp.pl?gene=CDKN1B |
| CSNK2B | Casein Kinase 2 Beta | Protein Coding | P67870 | 61 | GC06P181896 | 0.142190366983414 | https://www.genecards.org/cgi-bin/carddisp.pl?gene=CSNK2B |
| DES | Desmin | Protein Coding | P17661 | 61 | GC02P219418 | 0.142190366983414 | https://www.genecards.org/cgi-bin/carddisp.pl?gene=DES |
| GNAQ | G Protein Subunit Alpha Q | Protein Coding | P50148 | 61 | GC09M077716 | 0.142190366983414 | https://www.genecards.org/cgi-bin/carddisp.pl?gene=GNAQ |
| MAP3K5 | Mitogen-Activated Protein Kinase Kinase Kinase 5 | Protein Coding | Q99683 | 61 | GC06M136557 | 0.142190366983414 | https://www.genecards.org/cgi-bin/carddisp.pl?gene=MAP3K5 |
| NRP1 | Neuropilin 1 | Protein Coding | O14786 | 61 | GC10M033177 | 0.142190366983414 | https://www.genecards.org/cgi-bin/carddisp.pl?gene=NRP1 |
| PRKCE | Protein Kinase C Epsilon | Protein Coding | Q02156 | 61 | GC02P045651 | 0.142190366983414 | https://www.genecards.org/cgi-bin/carddisp.pl?gene=PRKCE |
| REL | REL Proto-Oncogene, NF-KB Subunit | Protein Coding | Q04864 | 61 | GC02P060881 | 0.142190366983414 | https://www.genecards.org/cgi-bin/carddisp.pl?gene=REL |
| YY1 | YY1 Transcription Factor | Protein Coding | P25490 | 61 | GC14P100238 | 0.142190366983414 | https://www.genecards.org/cgi-bin/carddisp.pl?gene=YY1 |
| ABCB4 | ATP Binding Cassette Subfamily B Member 4 | Protein Coding | P21439 | 60 | GC07M087365 | 0.142190366983414 | https://www.genecards.org/cgi-bin/carddisp.pl?gene=ABCB4 |
| ACSL4 | Acyl-CoA Synthetase Long Chain Family Member 4 | Protein Coding | O60488 | 60 | GC0XM109624 | 0.142190366983414 | https://www.genecards.org/cgi-bin/carddisp.pl?gene=ACSL4 |
| ADORA2B | Adenosine A2b Receptor | Protein Coding | P29275 | 60 | GC17P153945 | 0.142190366983414 | https://www.genecards.org/cgi-bin/carddisp.pl?gene=ADORA2B |
| ANGPT1 | Angiopoietin 1 | Protein Coding | Q15389 | 60 | GC08M107246 | 0.142190366983414 | https://www.genecards.org/cgi-bin/carddisp.pl?gene=ANGPT1 |
| ANXA5 | Annexin A5 | Protein Coding | P08758 | 60 | GC04M121667 | 0.142190366983414 | https://www.genecards.org/cgi-bin/carddisp.pl?gene=ANXA5 |
| C5 | Complement C5 | Protein Coding | P01031 | 60 | GC09M122525 | 0.142190366983414 | https://www.genecards.org/cgi-bin/carddisp.pl?gene=C5 |
| CCR5 | C-C Motif Chemokine Receptor 5 | Protein Coding | P51681 | 60 | GC03P064850 | 0.142190366983414 | https://www.genecards.org/cgi-bin/carddisp.pl?gene=CCR5 |
| F13A1 | Coagulation Factor XIII A Chain | Protein Coding | P00488 | 60 | GC06M006144 | 0.142190366983414 | https://www.genecards.org/cgi-bin/carddisp.pl?gene=F13A1 |
| FCGR3A | Fc Gamma Receptor IIIa | Protein Coding | P08637 | 60 | GC01M161541 | 0.142190366983414 | https://www.genecards.org/cgi-bin/carddisp.pl?gene=FCGR3A |
| GAMT | Guanidinoacetate N-Methyltransferase | Protein Coding | Q14353 | 60 | GC19M001397 | 0.142190366983414 | https://www.genecards.org/cgi-bin/carddisp.pl?gene=GAMT |
| GPI | Glucose-6-Phosphate Isomerase | Protein Coding | P06744 | 60 | GC19P034359 | 0.142190366983414 | https://www.genecards.org/cgi-bin/carddisp.pl?gene=GPI |
| KLK1 | Kallikrein 1 | Protein Coding | P06870 | 60 | GC19M050819 | 0.142190366983414 | https://www.genecards.org/cgi-bin/carddisp.pl?gene=KLK1 |
| LOX | Lysyl Oxidase | Protein Coding | P28300 | 60 | GC05M122063 | 0.142190366983414 | https://www.genecards.org/cgi-bin/carddisp.pl?gene=LOX |
| P2RX7 | Purinergic Receptor P2X 7 | Protein Coding | Q99572 | 60 | GC12P138914 | 0.142190366983414 | https://www.genecards.org/cgi-bin/carddisp.pl?gene=P2RX7 |
| RASA1 | RAS P21 Protein Activator 1 | Protein Coding | P20936 | 60 | GC05P087267 | 0.142190366983414 | https://www.genecards.org/cgi-bin/carddisp.pl?gene=RASA1 |
| SLC12A3 | Solute Carrier Family 12 Member 3 | Protein Coding | P55017 | 60 | GC16P056865 | 0.142190366983414 | https://www.genecards.org/cgi-bin/carddisp.pl?gene=SLC12A3 |
| TLR5 | Toll Like Receptor 5 | Protein Coding | O60602 | 60 | GC01M223109 | 0.142190366983414 | https://www.genecards.org/cgi-bin/carddisp.pl?gene=TLR5 |
| ACP5 | Acid Phosphatase 5, Tartrate Resistant | Protein Coding | P13686 | 59 | GC19M011574 | 0.142190366983414 | https://www.genecards.org/cgi-bin/carddisp.pl?gene=ACP5 |
| ACSL1 | Acyl-CoA Synthetase Long Chain Family Member 1 | Protein Coding | P33121 | 59 | GC04M184755 | 0.142190366983414 | https://www.genecards.org/cgi-bin/carddisp.pl?gene=ACSL1 |
| BCL6 | BCL6 Transcription Repressor | Protein Coding | P41182 | 59 | GC03M187721 | 0.142190366983414 | https://www.genecards.org/cgi-bin/carddisp.pl?gene=BCL6 |
| CYP24A1 | Cytochrome P450 Family 24 Subfamily A Member 1 | Protein Coding | Q07973 | 59 | GC20M054153 | 0.142190366983414 | https://www.genecards.org/cgi-bin/carddisp.pl?gene=CYP24A1 |
| CYP27B1 | Cytochrome P450 Family 27 Subfamily B Member 1 | Protein Coding | O15528 | 59 | GC12M060484 | 0.142190366983414 | https://www.genecards.org/cgi-bin/carddisp.pl?gene=CYP27B1 |
| DUSP1 | Dual Specificity Phosphatase 1 | Protein Coding | P28562 | 59 | GC05M172768 | 0.142190366983414 | https://www.genecards.org/cgi-bin/carddisp.pl?gene=DUSP1 |
| IGFBP7 | Insulin Like Growth Factor Binding Protein 7 | Protein Coding | Q16270 | 59 | GC04M057030 | 0.142190366983414 | https://www.genecards.org/cgi-bin/carddisp.pl?gene=IGFBP7 |
| IL7R | Interleukin 7 Receptor | Protein Coding | P16871 | 59 | GC05P035852 | 0.142190366983414 | https://www.genecards.org/cgi-bin/carddisp.pl?gene=IL7R |
| MYH6 | Myosin Heavy Chain 6 | Protein Coding | P13533 | 59 | GC14M023381 | 0.142190366983414 | https://www.genecards.org/cgi-bin/carddisp.pl?gene=MYH6 |
| NTF4 | Neurotrophin 4 | Protein Coding | P34130 | 59 | GC19M109030 | 0.142190366983414 | https://www.genecards.org/cgi-bin/carddisp.pl?gene=NTF4 |
| PML | PML Nuclear Body Scaffold | Protein Coding | P29590 | 59 | GC15P073994 | 0.142190366983414 | https://www.genecards.org/cgi-bin/carddisp.pl?gene=PML |
| RELB | RELB Proto-Oncogene, NF-KB Subunit | Protein Coding | Q01201 | 59 | GC19P152702 | 0.142190366983414 | https://www.genecards.org/cgi-bin/carddisp.pl?gene=RELB |
| ANG | Angiogenin | Protein Coding | P03950 | 58 | GC14P057256 | 0.142190366983414 | https://www.genecards.org/cgi-bin/carddisp.pl?gene=ANG |
| AOC3 | Amine Oxidase Copper Containing 3 | Protein Coding | Q16853 | 58 | GC17P042851 | 0.142190366983414 | https://www.genecards.org/cgi-bin/carddisp.pl?gene=AOC3 |
| BCL2L11 | BCL2 Like 11 | Protein Coding | O43521 | 58 | GC02P111119 | 0.142190366983414 | https://www.genecards.org/cgi-bin/carddisp.pl?gene=BCL2L11 |
| CALCRL | Calcitonin Receptor Like Receptor | Protein Coding | Q16602 | 58 | GC02M187341 | 0.142190366983414 | https://www.genecards.org/cgi-bin/carddisp.pl?gene=CALCRL |
| CANX | Calnexin | Protein Coding | P27824 | 58 | GC05P179678 | 0.142190366983414 | https://www.genecards.org/cgi-bin/carddisp.pl?gene=CANX |
| CD163 | CD163 Molecule | Protein Coding | Q86VB7 | 58 | GC12M035717 | 0.142190366983414 | https://www.genecards.org/cgi-bin/carddisp.pl?gene=CD163 |
| CKM | Creatine Kinase, M-Type | Protein Coding | P06732 | 58 | GC19M045306 | 0.142190366983414 | https://www.genecards.org/cgi-bin/carddisp.pl?gene=CKM |
| CPOX | Coproporphyrinogen Oxidase | Protein Coding | P36551 | 58 | GC03M098576 | 0.142190366983414 | https://www.genecards.org/cgi-bin/carddisp.pl?gene=CPOX |
| CUBN | Cubilin | Protein Coding | O60494 | 58 | GC10M016824 | 0.142190366983414 | https://www.genecards.org/cgi-bin/carddisp.pl?gene=CUBN |
| CYP1A2 | Cytochrome P450 Family 1 Subfamily A Member 2 | Protein Coding | P05177 | 58 | GC15P074748 | 0.142190366983414 | https://www.genecards.org/cgi-bin/carddisp.pl?gene=CYP1A2 |
| DRD4 | Dopamine Receptor D4 | Protein Coding | P21917 | 58 | GC11P020260 | 0.142190366983414 | https://www.genecards.org/cgi-bin/carddisp.pl?gene=DRD4 |
| GNB1 | G Protein Subunit Beta 1 | Protein Coding | P62873 | 58 | GC01M001785 | 0.142190366983414 | https://www.genecards.org/cgi-bin/carddisp.pl?gene=GNB1 |
| HFE | Homeostatic Iron Regulator | Protein Coding | Q30201 | 58 | GC06P026087 | 0.142190366983414 | https://www.genecards.org/cgi-bin/carddisp.pl?gene=HFE |
| HNRNPK | Heterogeneous Nuclear Ribonucleoprotein K | Protein Coding | P61978 | 58 | GC09M121666 | 0.142190366983414 | https://www.genecards.org/cgi-bin/carddisp.pl?gene=HNRNPK |
| HPGD | 15-Hydroxyprostaglandin Dehydrogenase | Protein Coding | P15428 | 58 | GC04M174490 | 0.142190366983414 | https://www.genecards.org/cgi-bin/carddisp.pl?gene=HPGD |
| HTR3A | 5-Hydroxytryptamine Receptor 3A | Protein Coding | P46098 | 58 | GC11P113975 | 0.142190366983414 | https://www.genecards.org/cgi-bin/carddisp.pl?gene=HTR3A |
| IL1RAP | Interleukin 1 Receptor Accessory Protein | Protein Coding | Q9NPH3 | 58 | GC03P190514 | 0.142190366983414 | https://www.genecards.org/cgi-bin/carddisp.pl?gene=IL1RAP |
| LAMB3 | Laminin Subunit Beta 3 | Protein Coding | Q13751 | 58 | GC01M209614 | 0.142190366983414 | https://www.genecards.org/cgi-bin/carddisp.pl?gene=LAMB3 |
| LTF | Lactotransferrin | Protein Coding | P02788 | 58 | GC03M046435 | 0.142190366983414 | https://www.genecards.org/cgi-bin/carddisp.pl?gene=LTF |
| NUMA1 | Nuclear Mitotic Apparatus Protein 1 | Protein Coding | Q14980 | 58 | GC11M072002 | 0.142190366983414 | https://www.genecards.org/cgi-bin/carddisp.pl?gene=NUMA1 |
| PHB1 | Prohibitin 1 | Protein Coding | P35232 | 58 | GC17M097550 | 0.142190366983414 | https://www.genecards.org/cgi-bin/carddisp.pl?gene=PHB1 |
| SEMA3A | Semaphorin 3A | Protein Coding | Q14563 | 58 | GC07M083955 | 0.142190366983414 | https://www.genecards.org/cgi-bin/carddisp.pl?gene=SEMA3A |
| SLC25A20 | Solute Carrier Family 25 Member 20 | Protein Coding | O43772 | 58 | GC03M048978 | 0.142190366983414 | https://www.genecards.org/cgi-bin/carddisp.pl?gene=SLC25A20 |
| SOX9 | SRY-Box Transcription Factor 9 | Protein Coding | P48436 | 58 | GC17P072121 | 0.142190366983414 | https://www.genecards.org/cgi-bin/carddisp.pl?gene=SOX9 |
| TNFRSF13C | TNF Receptor Superfamily Member 13C | Protein Coding | Q96RJ3 | 58 | GC22M086155 | 0.142190366983414 | https://www.genecards.org/cgi-bin/carddisp.pl?gene=TNFRSF13C |
| TNFSF13B | TNF Superfamily Member 13b | Protein Coding | Q9Y275 | 58 | GC13P108251 | 0.142190366983414 | https://www.genecards.org/cgi-bin/carddisp.pl?gene=TNFSF13B |
| TP73 | Tumor Protein P73 | Protein Coding | O15350 | 58 | GC01P003652 | 0.142190366983414 | https://www.genecards.org/cgi-bin/carddisp.pl?gene=TP73 |
| BCAT2 | Branched Chain Amino Acid Transaminase 2 | Protein Coding | O15382 | 57 | GC19M048795 | 0.142190366983414 | https://www.genecards.org/cgi-bin/carddisp.pl?gene=BCAT2 |
| BCKDK | Branched Chain Keto Acid Dehydrogenase Kinase | Protein Coding | O14874 | 57 | GC16P121086 | 0.142190366983414 | https://www.genecards.org/cgi-bin/carddisp.pl?gene=BCKDK |
| CXCR1 | C-X-C Motif Chemokine Receptor 1 | Protein Coding | P25024 | 57 | GC02M218162 | 0.142190366983414 | https://www.genecards.org/cgi-bin/carddisp.pl?gene=CXCR1 |
| FKBP4 | FKBP Prolyl Isomerase 4 | Protein Coding | Q02790 | 57 | GC12P073950 | 0.142190366983414 | https://www.genecards.org/cgi-bin/carddisp.pl?gene=FKBP4 |
| GDF5 | Growth Differentiation Factor 5 | Protein Coding | P43026 | 57 | GC20M035433 | 0.142190366983414 | https://www.genecards.org/cgi-bin/carddisp.pl?gene=GDF5 |
| GLO1 | Glyoxalase I | Protein Coding | Q04760 | 57 | GC06M106643 | 0.142190366983414 | https://www.genecards.org/cgi-bin/carddisp.pl?gene=GLO1 |
| IL10RA | Interleukin 10 Receptor Subunit Alpha | Protein Coding | Q13651 | 57 | GC11P118000 | 0.142190366983414 | https://www.genecards.org/cgi-bin/carddisp.pl?gene=IL10RA |
| MKI67 | Marker Of Proliferation Ki-67 | Protein Coding | P46013 | 57 | GC10M128096 | 0.142190366983414 | https://www.genecards.org/cgi-bin/carddisp.pl?gene=MKI67 |
| NRP2 | Neuropilin 2 | Protein Coding | O60462 | 57 | GC02P205681 | 0.142190366983414 | https://www.genecards.org/cgi-bin/carddisp.pl?gene=NRP2 |
| PDK4 | Pyruvate Dehydrogenase Kinase 4 | Protein Coding | Q16654 | 57 | GC07M095583 | 0.142190366983414 | https://www.genecards.org/cgi-bin/carddisp.pl?gene=PDK4 |
| SLC16A2 | Solute Carrier Family 16 Member 2 | Protein Coding | P36021 | 57 | GC0XP075145 | 0.142190366983414 | https://www.genecards.org/cgi-bin/carddisp.pl?gene=SLC16A2 |
| TNFSF13 | TNF Superfamily Member 13 | Protein Coding | O75888 | 57 | GC17P007558 | 0.142190366983414 | https://www.genecards.org/cgi-bin/carddisp.pl?gene=TNFSF13 |
| TUB | TUB Bipartite Transcription Factor | Protein Coding | P50607 | 57 | GC11P008019 | 0.142190366983414 | https://www.genecards.org/cgi-bin/carddisp.pl?gene=TUB |
| TUBA1B | Tubulin Alpha 1b | Protein Coding | P68363 | 57 | GC12M049127 | 0.142190366983414 | https://www.genecards.org/cgi-bin/carddisp.pl?gene=TUBA1B |
| ADRA2B | Adrenoceptor Alpha 2B | Protein Coding | P18089 | 56 | GC02M096112 | 0.142190366983414 | https://www.genecards.org/cgi-bin/carddisp.pl?gene=ADRA2B |
| BCKDHA | Branched Chain Keto Acid Dehydrogenase E1 Subunit Alpha | Protein Coding | P12694 | 56 | GC19P152574 | 0.142190366983414 | https://www.genecards.org/cgi-bin/carddisp.pl?gene=BCKDHA |
| FCGRT | Fc Gamma Receptor And Transporter | Protein Coding | P55899 | 56 | GC19P049506 | 0.142190366983414 | https://www.genecards.org/cgi-bin/carddisp.pl?gene=FCGRT |
| FGF14 | Fibroblast Growth Factor 14 | Protein Coding | Q92915 | 56 | GC13M101710 | 0.142190366983414 | https://www.genecards.org/cgi-bin/carddisp.pl?gene=FGF14 |
| FOXC2 | Forkhead Box C2 | Protein Coding | Q99958 | 56 | GC16P122670 | 0.142190366983414 | https://www.genecards.org/cgi-bin/carddisp.pl?gene=FOXC2 |
| GRB10 | Growth Factor Receptor Bound Protein 10 | Protein Coding | Q13322 | 56 | GC07M050590 | 0.142190366983414 | https://www.genecards.org/cgi-bin/carddisp.pl?gene=GRB10 |
| IL12A | Interleukin 12A | Protein Coding | P29459 | 56 | GC03P159988 | 0.142190366983414 | https://www.genecards.org/cgi-bin/carddisp.pl?gene=IL12A |
| PITX1 | Paired Like Homeodomain 1 | Protein Coding | P78337 | 56 | GC05M135027 | 0.142190366983414 | https://www.genecards.org/cgi-bin/carddisp.pl?gene=PITX1 |
| RBX1 | Ring-Box 1 | Protein Coding | P62877 | 56 | GC22P040951 | 0.142190366983414 | https://www.genecards.org/cgi-bin/carddisp.pl?gene=RBX1 |
| VEGFB | Vascular Endothelial Growth Factor B | Protein Coding | P49765 | 56 | GC11P064234 | 0.142190366983414 | https://www.genecards.org/cgi-bin/carddisp.pl?gene=VEGFB |
| AGO2 | Argonaute RISC Catalytic Component 2 | Protein Coding | Q9UKV8 | 55 | GC08M141032 | 0.142190366983414 | https://www.genecards.org/cgi-bin/carddisp.pl?gene=AGO2 |
| ASCL1 | Achaete-Scute Family BHLH Transcription Factor 1 | Protein Coding | P50553 | 55 | GC12P102957 | 0.142190366983414 | https://www.genecards.org/cgi-bin/carddisp.pl?gene=ASCL1 |
| CERS2 | Ceramide Synthase 2 | Protein Coding | Q96G23 | 55 | GC01M150960 | 0.142190366983414 | https://www.genecards.org/cgi-bin/carddisp.pl?gene=CERS2 |
| GM2A | Ganglioside GM2 Activator | Protein Coding | P17900 | 55 | GC05P151212 | 0.142190366983414 | https://www.genecards.org/cgi-bin/carddisp.pl?gene=GM2A |
| MLXIPL | MLX Interacting Protein Like | Protein Coding | Q9NP71 | 55 | GC07M073593 | 0.142190366983414 | https://www.genecards.org/cgi-bin/carddisp.pl?gene=MLXIPL |
| NEUROD1 | Neuronal Differentiation 1 | Protein Coding | Q13562 | 55 | GC02M181673 | 0.142190366983414 | https://www.genecards.org/cgi-bin/carddisp.pl?gene=NEUROD1 |
| PLA2G10 | Phospholipase A2 Group X | Protein Coding | O15496 | 55 | GC16M014672 | 0.142190366983414 | https://www.genecards.org/cgi-bin/carddisp.pl?gene=PLA2G10 |
| POU3F2 | POU Class 3 Homeobox 2 | Protein Coding | P20265 | 55 | GC06P098834 | 0.142190366983414 | https://www.genecards.org/cgi-bin/carddisp.pl?gene=POU3F2 |
| PZP | PZP Alpha-2-Macroglobulin Like | Protein Coding | P20742 | 55 | GC12M035772 | 0.142190366983414 | https://www.genecards.org/cgi-bin/carddisp.pl?gene=PZP |
| SLC10A1 | Solute Carrier Family 10 Member 1 | Protein Coding | Q14973 | 55 | GC14M069775 | 0.142190366983414 | https://www.genecards.org/cgi-bin/carddisp.pl?gene=SLC10A1 |
| SPINT2 | Serine Peptidase Inhibitor, Kunitz Type 2 | Protein Coding | O43291 | 55 | GC19P038244 | 0.142190366983414 | https://www.genecards.org/cgi-bin/carddisp.pl?gene=SPINT2 |
| STRADA | STE20 Related Adaptor Alpha | Protein Coding | Q7RTN6 | 55 | GC17M097801 | 0.142190366983414 | https://www.genecards.org/cgi-bin/carddisp.pl?gene=STRADA |
| TNFSF15 | TNF Superfamily Member 15 | Protein Coding | O95150 | 55 | GC09M114784 | 0.142190366983414 | https://www.genecards.org/cgi-bin/carddisp.pl?gene=TNFSF15 |
| CAPN10 | Calpain 10 | Protein Coding | Q9HC96 | 54 | GC02P240586 | 0.142190366983414 | https://www.genecards.org/cgi-bin/carddisp.pl?gene=CAPN10 |
| CCL21 | C-C Motif Chemokine Ligand 21 | Protein Coding | O00585 | 54 | GC09M034709 | 0.142190366983414 | https://www.genecards.org/cgi-bin/carddisp.pl?gene=CCL21 |
| CLCF1 | Cardiotrophin Like Cytokine Factor 1 | Protein Coding | Q9UBD9 | 54 | GC11M067364 | 0.142190366983414 | https://www.genecards.org/cgi-bin/carddisp.pl?gene=CLCF1 |
| CNOT3 | CCR4-NOT Transcription Complex Subunit 3 | Protein Coding | O75175 | 54 | GC19P153091 | 0.142190366983414 | https://www.genecards.org/cgi-bin/carddisp.pl?gene=CNOT3 |
| FLT3LG | Fms Related Receptor Tyrosine Kinase 3 Ligand | Protein Coding | P49771 | 54 | GC19P152864 | 0.142190366983414 | https://www.genecards.org/cgi-bin/carddisp.pl?gene=FLT3LG |
| IFT88 | Intraflagellar Transport 88 | Protein Coding | Q13099 | 54 | GC13P021150 | 0.142190366983414 | https://www.genecards.org/cgi-bin/carddisp.pl?gene=IFT88 |
| IL22 | Interleukin 22 | Protein Coding | Q9GZX6 | 54 | GC12M068248 | 0.142190366983414 | https://www.genecards.org/cgi-bin/carddisp.pl?gene=IL22 |
| MYOC | Myocilin | Protein Coding | Q99972 | 54 | GC01M171604 | 0.142190366983414 | https://www.genecards.org/cgi-bin/carddisp.pl?gene=MYOC |
| ORM1 | Orosomucoid 1 | Protein Coding | P02763 | 54 | GC09P114323 | 0.142190366983414 | https://www.genecards.org/cgi-bin/carddisp.pl?gene=ORM1 |
| PECAM1 | Platelet And Endothelial Cell Adhesion Molecule 1 | Protein Coding | P16284 | 54 | GC17M064319 | 0.142190366983414 | https://www.genecards.org/cgi-bin/carddisp.pl?gene=PECAM1 |
| TFF3 | Trefoil Factor 3 | Protein Coding | Q07654 | 54 | GC21M042311 | 0.142190366983414 | https://www.genecards.org/cgi-bin/carddisp.pl?gene=TFF3 |
| AGK | Acylglycerol Kinase | Protein Coding | Q53H12 | 53 | GC07P141551 | 0.142190366983414 | https://www.genecards.org/cgi-bin/carddisp.pl?gene=AGK |
| CCL19 | C-C Motif Chemokine Ligand 19 | Protein Coding | Q99731 | 53 | GC09M035850 | 0.142190366983414 | https://www.genecards.org/cgi-bin/carddisp.pl?gene=CCL19 |
| GPER1 | G Protein-Coupled Estrogen Receptor 1 | Protein Coding | Q99527 | 53 | GC07P018825 | 0.142190366983414 | https://www.genecards.org/cgi-bin/carddisp.pl?gene=GPER1 |
| HTR5A | 5-Hydroxytryptamine Receptor 5A | Protein Coding | P47898 | 53 | GC07P155070 | 0.142190366983414 | https://www.genecards.org/cgi-bin/carddisp.pl?gene=HTR5A |
| NFYA | Nuclear Transcription Factor Y Subunit Alpha | Protein Coding | P23511 | 53 | GC06P182047 | 0.142190366983414 | https://www.genecards.org/cgi-bin/carddisp.pl?gene=NFYA |
| ARL6 | ARF Like GTPase 6 | Protein Coding | Q9H0F7 | 52 | GC03P097764 | 0.142190366983414 | https://www.genecards.org/cgi-bin/carddisp.pl?gene=ARL6 |
| CDK14 | Cyclin Dependent Kinase 14 | Protein Coding | O94921 | 52 | GC07P095672 | 0.142190366983414 | https://www.genecards.org/cgi-bin/carddisp.pl?gene=CDK14 |
| GPR55 | G Protein-Coupled Receptor 55 | Protein Coding | Q9Y2T6 | 52 | GC02M230907 | 0.142190366983414 | https://www.genecards.org/cgi-bin/carddisp.pl?gene=GPR55 |
| IL15RA | Interleukin 15 Receptor Subunit Alpha | Protein Coding | Q13261 | 52 | GC10M005943 | 0.142190366983414 | https://www.genecards.org/cgi-bin/carddisp.pl?gene=IL15RA |
| LGR4 | Leucine Rich Repeat Containing G Protein-Coupled Receptor 4 | Protein Coding | Q9BXB1 | 52 | GC11M027365 | 0.142190366983414 | https://www.genecards.org/cgi-bin/carddisp.pl?gene=LGR4 |
| MIP | Major Intrinsic Protein Of Lens Fiber | Protein Coding | P30301 | 52 | GC12M056449 | 0.142190366983414 | https://www.genecards.org/cgi-bin/carddisp.pl?gene=MIP |
| MTF1 | Metal Regulatory Transcription Factor 1 | Protein Coding | Q14872 | 52 | GC01M037810 | 0.142190366983414 | https://www.genecards.org/cgi-bin/carddisp.pl?gene=MTF1 |
| MYOG | Myogenin | Protein Coding | P15173 | 52 | GC01M203083 | 0.142190366983414 | https://www.genecards.org/cgi-bin/carddisp.pl?gene=MYOG |
| PF4 | Platelet Factor 4 | Protein Coding | P02776 | 52 | GC04M073980 | 0.142190366983414 | https://www.genecards.org/cgi-bin/carddisp.pl?gene=PF4 |
| SFRP2 | Secreted Frizzled Related Protein 2 | Protein Coding | Q96HF1 | 52 | GC04M153780 | 0.142190366983414 | https://www.genecards.org/cgi-bin/carddisp.pl?gene=SFRP2 |
| SLC15A2 | Solute Carrier Family 15 Member 2 | Protein Coding | Q16348 | 52 | GC03P121894 | 0.142190366983414 | https://www.genecards.org/cgi-bin/carddisp.pl?gene=SLC15A2 |
| TSLP | Thymic Stromal Lymphopoietin | Protein Coding | Q969D9 | 52 | GC05P111070 | 0.142190366983414 | https://www.genecards.org/cgi-bin/carddisp.pl?gene=TSLP |
| ANKRD26 | Ankyrin Repeat Domain Containing 26 | Protein Coding | Q9UPS8 | 51 | GC10M026947 | 0.142190366983414 | https://www.genecards.org/cgi-bin/carddisp.pl?gene=ANKRD26 |
| CTF1 | Cardiotrophin 1 | Protein Coding | Q16619 | 51 | GC16P121070 | 0.142190366983414 | https://www.genecards.org/cgi-bin/carddisp.pl?gene=CTF1 |
| HOXA9 | Homeobox A9 | Protein Coding | P31269 | 51 | GC07M027162 | 0.142190366983414 | https://www.genecards.org/cgi-bin/carddisp.pl?gene=HOXA9 |
| NFYC | Nuclear Transcription Factor Y Subunit Gamma | Protein Coding | Q13952 | 51 | GC01P040691 | 0.142190366983414 | https://www.genecards.org/cgi-bin/carddisp.pl?gene=NFYC |
| NSMF | NMDA Receptor Synaptonuclear Signaling And Neuronal Migration Factor | Protein Coding | Q6X4W1 | 51 | GC09M137447 | 0.142190366983414 | https://www.genecards.org/cgi-bin/carddisp.pl?gene=NSMF |
| RAI1 | Retinoic Acid Induced 1 | Protein Coding | Q7Z5J4 | 51 | GC17P154060 | 0.142190366983414 | https://www.genecards.org/cgi-bin/carddisp.pl?gene=RAI1 |
| STRADB | STE20 Related Adaptor Beta | Protein Coding | Q9C0K7 | 51 | GC02P201387 | 0.142190366983414 | https://www.genecards.org/cgi-bin/carddisp.pl?gene=STRADB |
| SULF2 | Sulfatase 2 | Protein Coding | Q8IWU5 | 51 | GC20M047656 | 0.142190366983414 | https://www.genecards.org/cgi-bin/carddisp.pl?gene=SULF2 |
| TNFSF12 | TNF Superfamily Member 12 | Protein Coding | O43508 | 51 | GC17P153754 | 0.142190366983414 | https://www.genecards.org/cgi-bin/carddisp.pl?gene=TNFSF12 |
| BAIAP2L1 | BAR/IMD Domain Containing Adaptor Protein 2 Like 1 | Protein Coding | Q9UHR4 | 50 | GC07M098322 | 0.142190366983414 | https://www.genecards.org/cgi-bin/carddisp.pl?gene=BAIAP2L1 |
| C5AR2 | Complement C5a Receptor 2 | Protein Coding | Q9P296 | 50 | GC19P152780 | 0.142190366983414 | https://www.genecards.org/cgi-bin/carddisp.pl?gene=C5AR2 |
| CCN4 | Cellular Communication Network Factor 4 | Protein Coding | O95388 | 50 | GC08P133192 | 0.142190366983414 | https://www.genecards.org/cgi-bin/carddisp.pl?gene=CCN4 |
| CERS4 | Ceramide Synthase 4 | Protein Coding | Q9HA82 | 50 | GC19P008206 | 0.142190366983414 | https://www.genecards.org/cgi-bin/carddisp.pl?gene=CERS4 |
| CLK1 | CDC Like Kinase 1 | Protein Coding | P49759 | 50 | GC02M200853 | 0.142190366983414 | https://www.genecards.org/cgi-bin/carddisp.pl?gene=CLK1 |
| DEFA1 | Defensin Alpha 1 | Protein Coding | P59665 | 50 | GC08M006977 | 0.142190366983414 | https://www.genecards.org/cgi-bin/carddisp.pl?gene=DEFA1 |
| NMUR1 | Neuromedin U Receptor 1 | Protein Coding | Q9HB89 | 50 | GC02M232530 | 0.142190366983414 | https://www.genecards.org/cgi-bin/carddisp.pl?gene=NMUR1 |
| PKHD1 | PKHD1 Ciliary IPT Domain Containing Fibrocystin/Polyductin | Protein Coding | P08F94 | 50 | GC06M106836 | 0.142190366983414 | https://www.genecards.org/cgi-bin/carddisp.pl?gene=PKHD1 |
| YTHDC2 | YTH N6-Methyladenosine RNA Binding Protein C2 | Protein Coding | Q9H6S0 | 50 | GC05P113513 | 0.142190366983414 | https://www.genecards.org/cgi-bin/carddisp.pl?gene=YTHDC2 |
| ATRN | Attractin | Protein Coding | O75882 | 49 | GC20P003471 | 0.142190366983414 | https://www.genecards.org/cgi-bin/carddisp.pl?gene=ATRN |
| CAB39 | Calcium Binding Protein 39 | Protein Coding | Q9Y376 | 49 | GC02P230712 | 0.142190366983414 | https://www.genecards.org/cgi-bin/carddisp.pl?gene=CAB39 |
| IL1F10 | Interleukin 1 Family Member 10 | Protein Coding | Q8WWZ1 | 49 | GC02P113067 | 0.142190366983414 | https://www.genecards.org/cgi-bin/carddisp.pl?gene=IL1F10 |
| RGN | Regucalcin | Protein Coding | Q15493 | 49 | GC0XP060786 | 0.142190366983414 | https://www.genecards.org/cgi-bin/carddisp.pl?gene=RGN |
| SOCS5 | Suppressor Of Cytokine Signaling 5 | Protein Coding | O75159 | 49 | GC02P046698 | 0.142190366983414 | https://www.genecards.org/cgi-bin/carddisp.pl?gene=SOCS5 |
| CERS5 | Ceramide Synthase 5 | Protein Coding | Q8N5B7 | 48 | GC12M050129 | 0.142190366983414 | https://www.genecards.org/cgi-bin/carddisp.pl?gene=CERS5 |
| CLEC11A | C-Type Lectin Domain Containing 11A | Protein Coding | Q9Y240 | 48 | GC19P050723 | 0.142190366983414 | https://www.genecards.org/cgi-bin/carddisp.pl?gene=CLEC11A |
| CRTC3 | CREB Regulated Transcription Coactivator 3 | Protein Coding | Q6UUV7 | 48 | GC15P090529 | 0.142190366983414 | https://www.genecards.org/cgi-bin/carddisp.pl?gene=CRTC3 |
| GABPB1 | GA Binding Protein Transcription Factor Subunit Beta 1 | Protein Coding | Q06547 | 48 | GC15M050275 | 0.142190366983414 | https://www.genecards.org/cgi-bin/carddisp.pl?gene=GABPB1 |
| GUCA2B | Guanylate Cyclase Activator 2B | Protein Coding | Q16661 | 48 | GC01P042153 | 0.142190366983414 | https://www.genecards.org/cgi-bin/carddisp.pl?gene=GUCA2B |
| LECT2 | Leukocyte Cell Derived Chemotaxin 2 | Protein Coding | O14960 | 48 | GC05M135922 | 0.142190366983414 | https://www.genecards.org/cgi-bin/carddisp.pl?gene=LECT2 |
| PRLHR | Prolactin Releasing Hormone Receptor | Protein Coding | P49683 | 48 | GC10M118590 | 0.142190366983414 | https://www.genecards.org/cgi-bin/carddisp.pl?gene=PRLHR |
| ADM2 | Adrenomedullin 2 | Protein Coding | Q7Z4H4 | 47 | GC22P050481 | 0.142190366983414 | https://www.genecards.org/cgi-bin/carddisp.pl?gene=ADM2 |
| COBL | Cordon-Bleu WH2 Repeat Protein | Protein Coding | O75128 | 47 | GC07M051016 | 0.142190366983414 | https://www.genecards.org/cgi-bin/carddisp.pl?gene=COBL |
| CORT | Cortistatin | Protein Coding | O00230 | 47 | GC01P075313 | 0.142190366983414 | https://www.genecards.org/cgi-bin/carddisp.pl?gene=CORT |
| FSTL3 | Follistatin Like 3 | Protein Coding | O95633 | 47 | GC19P000676 | 0.142190366983414 | https://www.genecards.org/cgi-bin/carddisp.pl?gene=FSTL3 |
| MKX | Mohawk Homeobox | Protein Coding | Q8IYA7 | 47 | GC10M028570 | 0.142190366983414 | https://www.genecards.org/cgi-bin/carddisp.pl?gene=MKX |
| OSBPL3 | Oxysterol Binding Protein Like 3 | Protein Coding | Q9H4L5 | 46 | GC07M024836 | 0.142190366983414 | https://www.genecards.org/cgi-bin/carddisp.pl?gene=OSBPL3 |
| ASCL2 | Achaete-Scute Family BHLH Transcription Factor 2 | Protein Coding | Q99929 | 45 | GC11M015132 | 0.142190366983414 | https://www.genecards.org/cgi-bin/carddisp.pl?gene=ASCL2 |
| C1QL3 | Complement C1q Like 3 | Protein Coding | Q5VWW1 | 45 | GC10M016513 | 0.142190366983414 | https://www.genecards.org/cgi-bin/carddisp.pl?gene=C1QL3 |
| PHLDA1 | Pleckstrin Homology Like Domain Family A Member 1 | Protein Coding | Q8WV24 | 45 | GC12M076025 | 0.142190366983414 | https://www.genecards.org/cgi-bin/carddisp.pl?gene=PHLDA1 |
| RLN2 | Relaxin 2 | Protein Coding | P04090 | 45 | GC09M005871 | 0.142190366983414 | https://www.genecards.org/cgi-bin/carddisp.pl?gene=RLN2 |
| ACTR6 | Actin Related Protein 6 | Protein Coding | Q9GZN1 | 44 | GC12P100276 | 0.142190366983414 | https://www.genecards.org/cgi-bin/carddisp.pl?gene=ACTR6 |
| CAB39L | Calcium Binding Protein 39 Like | Protein Coding | Q9H9S4 | 44 | GC13M049308 | 0.142190366983414 | https://www.genecards.org/cgi-bin/carddisp.pl?gene=CAB39L |
| SLC16A11 | Solute Carrier Family 16 Member 11 | Protein Coding | Q8NCK7 | 44 | GC17M007041 | 0.142190366983414 | https://www.genecards.org/cgi-bin/carddisp.pl?gene=SLC16A11 |
| PLAC1 | Placenta Enriched 1 | Protein Coding | Q9HBJ0 | 43 | GC0XM134565 | 0.142190366983414 | https://www.genecards.org/cgi-bin/carddisp.pl?gene=PLAC1 |
| SH2B2 | SH2B Adaptor Protein 2 | Protein Coding | O14492 | 43 | GC07P110826 | 0.142190366983414 | https://www.genecards.org/cgi-bin/carddisp.pl?gene=SH2B2 |
| C1QTNF6 | C1q And TNF Related 6 | Protein Coding | Q9BXI9 | 42 | GC22M037180 | 0.142190366983414 | https://www.genecards.org/cgi-bin/carddisp.pl?gene=C1QTNF6 |
| FNDC4 | Fibronectin Type III Domain Containing 4 | Protein Coding | Q9H6D8 | 42 | GC02M027491 | 0.142190366983414 | https://www.genecards.org/cgi-bin/carddisp.pl?gene=FNDC4 |
| IGKC | Immunoglobulin Kappa Constant | Protein Coding | P01834 | 42 | GC02M092913 | 0.142190366983414 | https://www.genecards.org/cgi-bin/carddisp.pl?gene=IGKC |
| MARCHF7 | Membrane Associated Ring-CH-Type Finger 7 | Protein Coding | Q9H992 | 42 | GC02P159763 | 0.142190366983414 | https://www.genecards.org/cgi-bin/carddisp.pl?gene=MARCHF7 |
| TRARG1 | Trafficking Regulator Of GLUT4 (SLC2A4) 1 (Gene/Pseudogene) | Protein Coding | Q8IXB3 | 42 | GC17P153465 | 0.142190366983414 | https://www.genecards.org/cgi-bin/carddisp.pl?gene=TRARG1 |
| QRFPR | Pyroglutamylated RFamide Peptide Receptor | Protein Coding | Q96P65 | 41 | GC04M121328 | 0.142190366983414 | https://www.genecards.org/cgi-bin/carddisp.pl?gene=QRFPR |
| CHCHD5 | Coiled-Coil-Helix-Coiled-Coil-Helix Domain Containing 5 | Protein Coding | Q9BSY4 | 40 | GC02P154103 | 0.142190366983414 | https://www.genecards.org/cgi-bin/carddisp.pl?gene=CHCHD5 |
| NPW | Neuropeptide W | Protein Coding | Q8N729 | 40 | GC16P120167 | 0.142190366983414 | https://www.genecards.org/cgi-bin/carddisp.pl?gene=NPW |
| LEAP2 | Liver Enriched Antimicrobial Peptide 2 | Protein Coding | Q969E1 | 39 | GC05P132872 | 0.142190366983414 | https://www.genecards.org/cgi-bin/carddisp.pl?gene=LEAP2 |
| TIPRL | TOR Signaling Pathway Regulator | Protein Coding | O75663 | 39 | GC01P177256 | 0.142190366983414 | https://www.genecards.org/cgi-bin/carddisp.pl?gene=TIPRL |
| SLC25A34 | Solute Carrier Family 25 Member 34 | Protein Coding | Q6PIV7 | 37 | GC01P075654 | 0.142190366983414 | https://www.genecards.org/cgi-bin/carddisp.pl?gene=SLC25A34 |
| MIR145 | MicroRNA 145 | RNA Gene |  | 32 | GC05P149430 | 0.142190366983414 | https://www.genecards.org/cgi-bin/carddisp.pl?gene=MIR145 |
| MIR133B | MicroRNA 133b | RNA Gene |  | 31 | GC06P052148 | 0.142190366983414 | https://www.genecards.org/cgi-bin/carddisp.pl?gene=MIR133B |
| MIR146A | MicroRNA 146a | RNA Gene |  | 31 | GC05P160485 | 0.142190366983414 | https://www.genecards.org/cgi-bin/carddisp.pl?gene=MIR146A |
| MIR26B | MicroRNA 26b | RNA Gene |  | 31 | GC02P218402 | 0.142190366983414 | https://www.genecards.org/cgi-bin/carddisp.pl?gene=MIR26B |
| MIR29A | MicroRNA 29a | RNA Gene |  | 31 | GC07M130876 | 0.142190366983414 | https://www.genecards.org/cgi-bin/carddisp.pl?gene=MIR29A |
| MIR30E | MicroRNA 30e | RNA Gene |  | 31 | GC01P040754 | 0.142190366983414 | https://www.genecards.org/cgi-bin/carddisp.pl?gene=MIR30E |
| MIR23A | MicroRNA 23a | RNA Gene |  | 30 | GC19M108248 | 0.142190366983414 | https://www.genecards.org/cgi-bin/carddisp.pl?gene=MIR23A |
| MIR93 | MicroRNA 93 | RNA Gene |  | 30 | GC07M106714 | 0.142190366983414 | https://www.genecards.org/cgi-bin/carddisp.pl?gene=MIR93 |
| MIR99A | MicroRNA 99a | RNA Gene |  | 30 | GC21P016539 | 0.142190366983414 | https://www.genecards.org/cgi-bin/carddisp.pl?gene=MIR99A |
| MIRLET7I | MicroRNA Let-7i | RNA Gene |  | 30 | GC12P075610 | 0.142190366983414 | https://www.genecards.org/cgi-bin/carddisp.pl?gene=MIRLET7I |
| MIR193B | MicroRNA 193b | RNA Gene |  | 29 | GC16P120415 | 0.142190366983414 | https://www.genecards.org/cgi-bin/carddisp.pl?gene=MIR193B |
| MIR25 | MicroRNA 25 | RNA Gene |  | 29 | GC07M100093 | 0.142190366983414 | https://www.genecards.org/cgi-bin/carddisp.pl?gene=MIR25 |
| MIR134 | MicroRNA 134 | RNA Gene |  | 28 | GC14P121354 | 0.142190366983414 | https://www.genecards.org/cgi-bin/carddisp.pl?gene=MIR134 |
| MIR181A2 | MicroRNA 181a-2 | RNA Gene |  | 28 | GC09P124692 | 0.142190366983414 | https://www.genecards.org/cgi-bin/carddisp.pl?gene=MIR181A2 |
| MIR197 | MicroRNA 197 | RNA Gene |  | 28 | GC01P109598 | 0.142190366983414 | https://www.genecards.org/cgi-bin/carddisp.pl?gene=MIR197 |
| MIR29B1 | MicroRNA 29b-1 | RNA Gene |  | 28 | GC07M130877 | 0.142190366983414 | https://www.genecards.org/cgi-bin/carddisp.pl?gene=MIR29B1 |
| MIR584 | MicroRNA 584 | RNA Gene |  | 28 | GC05M149062 | 0.142190366983414 | https://www.genecards.org/cgi-bin/carddisp.pl?gene=MIR584 |
| HCG27 | HLA Complex Group 27 | RNA Gene |  | 27 | GC06P031197 | 0.142190366983414 | https://www.genecards.org/cgi-bin/carddisp.pl?gene=HCG27 |
| MIR423 | MicroRNA 423 | RNA Gene |  | 27 | GC17P030117 | 0.142190366983414 | https://www.genecards.org/cgi-bin/carddisp.pl?gene=MIR423 |
| MIR505 | MicroRNA 505 | RNA Gene |  | 26 | GC0XM139924 | 0.142190366983414 | https://www.genecards.org/cgi-bin/carddisp.pl?gene=MIR505 |
| LINC-ROR | Long Intergenic Non-Protein Coding RNA, Regulator Of Reprogramming | RNA Gene |  | 25 | GC18M057054 | 0.142190366983414 | https://www.genecards.org/cgi-bin/carddisp.pl?gene=LINC-ROR |
| MIR431 | MicroRNA 431 | RNA Gene |  | 25 | GC14P120345 | 0.142190366983414 | https://www.genecards.org/cgi-bin/carddisp.pl?gene=MIR431 |
| OIP5-AS1 | OIP5 Antisense RNA 1 | RNA Gene |  | 25 | GC15P192868 | 0.142190366983414 | https://www.genecards.org/cgi-bin/carddisp.pl?gene=OIP5-AS1 |
| MIR153-2 | MicroRNA 153-2 | RNA Gene |  | 24 | GC07M157574 | 0.142190366983414 | https://www.genecards.org/cgi-bin/carddisp.pl?gene=MIR153-2 |
| MIR1908 | MicroRNA 1908 | RNA Gene |  | 24 | GC11M061815 | 0.142190366983414 | https://www.genecards.org/cgi-bin/carddisp.pl?gene=MIR1908 |
| FIRRE | Firre Intergenic Repeating RNA Element | RNA Gene |  | 22 | GC0XM131691 | 0.142190366983414 | https://www.genecards.org/cgi-bin/carddisp.pl?gene=FIRRE |
| MIR320E | MicroRNA 320e | RNA Gene |  | 22 | GC19M108943 | 0.142190366983414 | https://www.genecards.org/cgi-bin/carddisp.pl?gene=MIR320E |
| MIR660 | MicroRNA 660 | RNA Gene |  | 22 | GC0XP050013 | 0.142190366983414 | https://www.genecards.org/cgi-bin/carddisp.pl?gene=MIR660 |
| MIR937 | MicroRNA 937 | RNA Gene |  | 21 | GC08M143812 | 0.142190366983414 | https://www.genecards.org/cgi-bin/carddisp.pl?gene=MIR937 |
| MIR1285-1 | MicroRNA 1285-1 | RNA Gene |  | 20 | GC07M092204 | 0.142190366983414 | https://www.genecards.org/cgi-bin/carddisp.pl?gene=MIR1285-1 |
| MIR543 | MicroRNA 543 | RNA Gene |  | 20 | GC14P120390 | 0.142190366983414 | https://www.genecards.org/cgi-bin/carddisp.pl?gene=MIR543 |
| ST7-OT3 | ST7 Overlapping Transcript 3 | RNA Gene |  | 20 | GC07P117455 | 0.142190366983414 | https://www.genecards.org/cgi-bin/carddisp.pl?gene=ST7-OT3 |
| CCL15-CCL14 | CCL15-CCL14 Readthrough (NMD Candidate) | RNA Gene |  | 17 | GC17M035983 | 0.142190366983414 | https://www.genecards.org/cgi-bin/carddisp.pl?gene=CCL15-CCL14 |
| MIR3907 | MicroRNA 3907 | RNA Gene |  | 17 | GC07M151433 | 0.142190366983414 | https://www.genecards.org/cgi-bin/carddisp.pl?gene=MIR3907 |
| MIR665 | MicroRNA 665 | RNA Gene |  | 16 | GC14P121393 | 0.142190366983414 | https://www.genecards.org/cgi-bin/carddisp.pl?gene=MIR665 |
| MIR4491 | MicroRNA 4491 | RNA Gene |  | 14 | GC11P111347 | 0.142190366983414 | https://www.genecards.org/cgi-bin/carddisp.pl?gene=MIR4491 |
| MIR1285-2 | MicroRNA 1285-2 | RNA Gene |  | 13 | GC02M070252 | 0.142190366983414 | https://www.genecards.org/cgi-bin/carddisp.pl?gene=MIR1285-2 |
| ST2 | Suppression Of Tumorigenicity 2 | Genetic Locus |  | 8 | GC11U990127 | 0.142190366983414 | https://www.genecards.org/cgi-bin/carddisp.pl?gene=ST2 |
| LOC109611593 | RUNX2 P1 Promoter Region | Functional Element |  | 2 | GC06P185467 | 0.142190366983414 | https://www.genecards.org/cgi-bin/carddisp.pl?gene=LOC109611593 |
| LOC107882127 | EDNRB Upstream Promoter Region | Functional Element |  | 1 | GC13P077975 | 0.142190366983414 | https://www.genecards.org/cgi-bin/carddisp.pl?gene=LOC107882127 |
| EDAR | Ectodysplasin A Receptor | Protein Coding | Q9UNE0 | 53 | GC02M108894 | 0.110678635537624 | https://www.genecards.org/cgi-bin/carddisp.pl?gene=EDAR |
