# Supplementary material for: Machine learning-based identification of leptin-associated biomarkers and prognostic prediction models in sepsis
Source: Front Cell Infect Microbiol. 2025 Sep 29;15:1630446. doi: 10.3389/fcimb.2025.1630446 (PMC12515905; doi:10.3389/fcimb.2025.1630446)
Supplement: Supplementary file 5 [file Table4.doc]

Supplementary Table 4. The hub genes.

| RNASE2 |
| --- |
| SRPK1 |
| ARHGEF18 |
| LTB4R |
| PGLYRP1 |
| NFATC3 |
| SLC26A8 |
| DYSF |
| JAK1 |
| MTF1 |
| RETN |
| ROPN1L |
| PRPF8 |
| F5 |
| FAM53B |
| ALPL |
| FES |
| CBX7 |
| FLOT2 |
| AGTRAP |
| PADI4 |
| KREMEN1 |
| TCN1 |
| LCN2 |
| ST3GAL4 |
| MYCBP2 |
| FUT7 |
| BPI |
| NBEAL2 |
| P2RX1 |
| DHRS13 |
| MMP8 |
| GRAMD1A |
| NQO2 |
| SEMA4A |
| CTSD |
| CDKN2C |
| NTNG2 |
| RNASE3 |
| SLC11A1 |
| RAB13 |
| EXT1 |
| NLRP1 |
| B3GNT8 |
| LTF |
| GGH |
| SPTBN1 |
| UBE2C |
| CEACAM8 |
| OLFM4 |
| CHST7 |
| PECAM1 |
| AIG1 |
| SMARCD3 |
| VSTM1 |
| CD24 |
| DPEP2 |
| CCNB2 |
| CKS2 |
| MPO |
| PILRA |
| TFF3 |
| TFRC |
| CEACAM6 |
| RNASE6 |
| MS4A3 |
| BEX1 |
| DEFA4 |
| CTSG |
| KRT23 |
