# Supplementary material for: Machine learning-based identification of leptin-associated biomarkers and prognostic prediction models in sepsis
Source: Front Cell Infect Microbiol. 2025 Sep 29;15:1630446. doi: 10.3389/fcimb.2025.1630446 (PMC12515905; doi:10.3389/fcimb.2025.1630446)
Supplement: Supplementary file 6 [file Table5.doc]

Supplementary Table 5. Feature used for model construction.

| **Prediction Model for Sepsis Mortality** | **Prognostic Risk Model** |
| --- | --- |
| DEFA4 | TFRC |
| KRT23 | RAB13 |
| TFRC | DEFA4 |
| PILRA | NLRP1 |
| BEX1 | KRT23 |
| TFF3 | KREMEN1 |
| MYCBP2 | PILRA |
| NQO2 | CDKN2C |
| CHST7 | BEX1 |
| VSTM1 | PRPF8 |
| F5 | CTSG |
| ST3GAL4 | CEACAM6 |
| EXT1 |  |
| SRPK1 |  |
