# Supplementary material for: Machine learning-based identification of leptin-associated biomarkers and prognostic prediction models in sepsis
Source: Front Cell Infect Microbiol. 2025 Sep 29;15:1630446. doi: 10.3389/fcimb.2025.1630446 (PMC12515905; doi:10.3389/fcimb.2025.1630446)

**Supplementary Figure 1.** (A) Prognostic classification model for 28-day mortality in sepsis compared with selected clinical features. (B) Comparison of C-index values between the time-to-event prognostic model and clinical features. (C) Univariate Cox regression analysis showing the prognostic value of the time-to-event model risk score and clinical features. (D) Multivariate Cox regression analysis confirming that the time-to-event model risk score serves as an independent prognostic factor in sepsis.


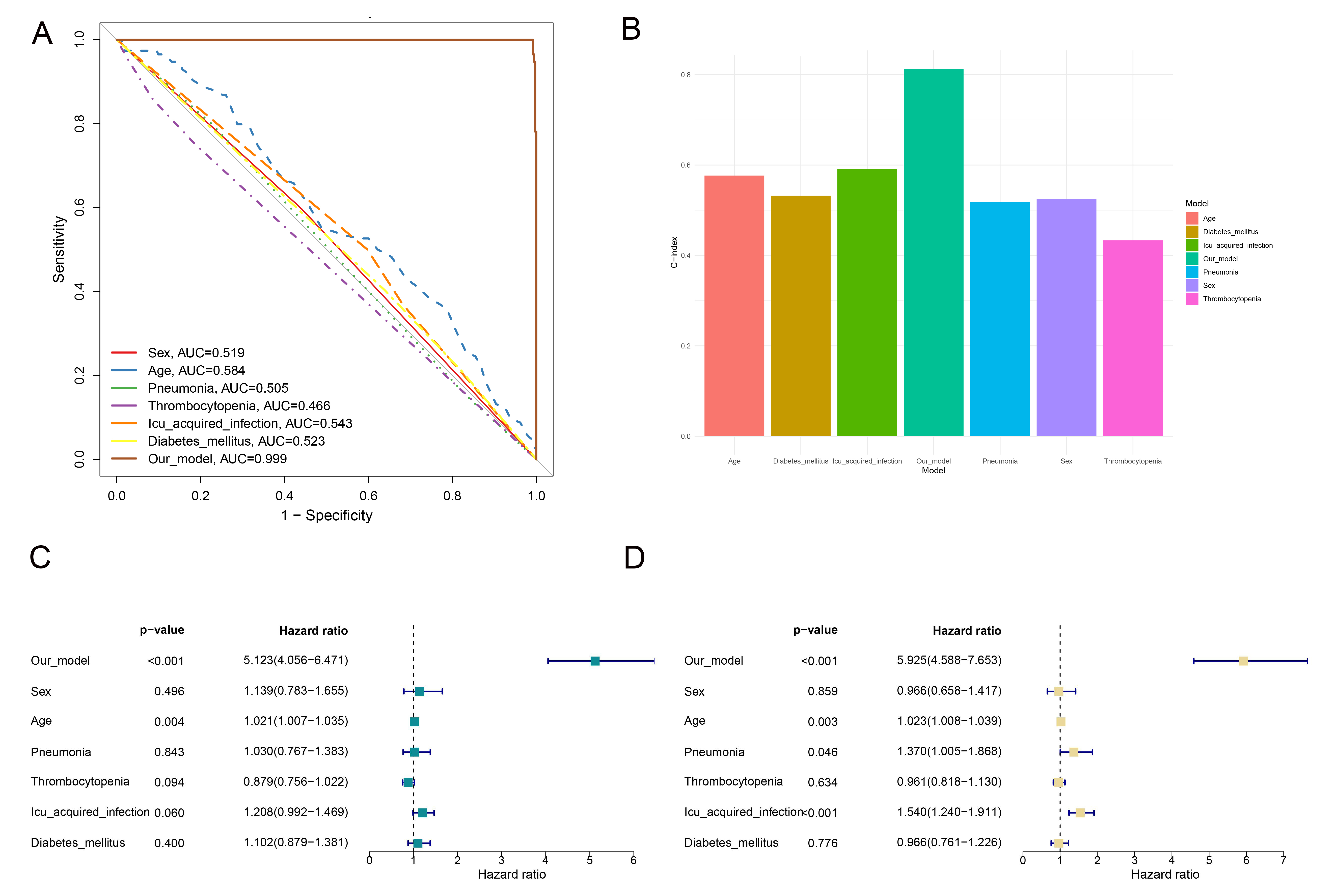

Supplement: Supplementary file 7 [file Table6.doc]
