# Supplementary material for: Machine learning-based identification of leptin-associated biomarkers and prognostic prediction models in sepsis
Source: Front Cell Infect Microbiol. 2025 Sep 29;15:1630446. doi: 10.3389/fcimb.2025.1630446 (PMC12515905; doi:10.3389/fcimb.2025.1630446)

Supplementary Figure 2. Preprocessing of Single-Cell RNA Sequencing Data: (A) Quality control metrics applied during normalization included the number of detected genes per cell (nFeature_RNA), the total transcript count per cell (nCount_RNA), the proportion of mitochondrial transcripts (percent.mt), and the proportion of ribosomal gene expression (percent.rb). (B) Hierarchical clustering dendrogram illustrating the grouping structure of single-cell profiles. (C) Principal component stability assessment using the Harmony algorithm to support reliable downstream clustering. (D) tSNE visualization of single-cell RNA-seq data showing six major immune cell populations, including Monocytes, NK cells, Neutrophils, Platelets, Erythroblasts, and B cells. (E) Dot plot illustrating the expression patterns of canonical marker genes used to resolve monocyte subsets into classical (CD14++), intermediate (CD14+CD16+), and non-classical (CD16+) lineages. Dot size represents the percentage of cells expressing a given gene, and color intensity corresponds to the average expression level.


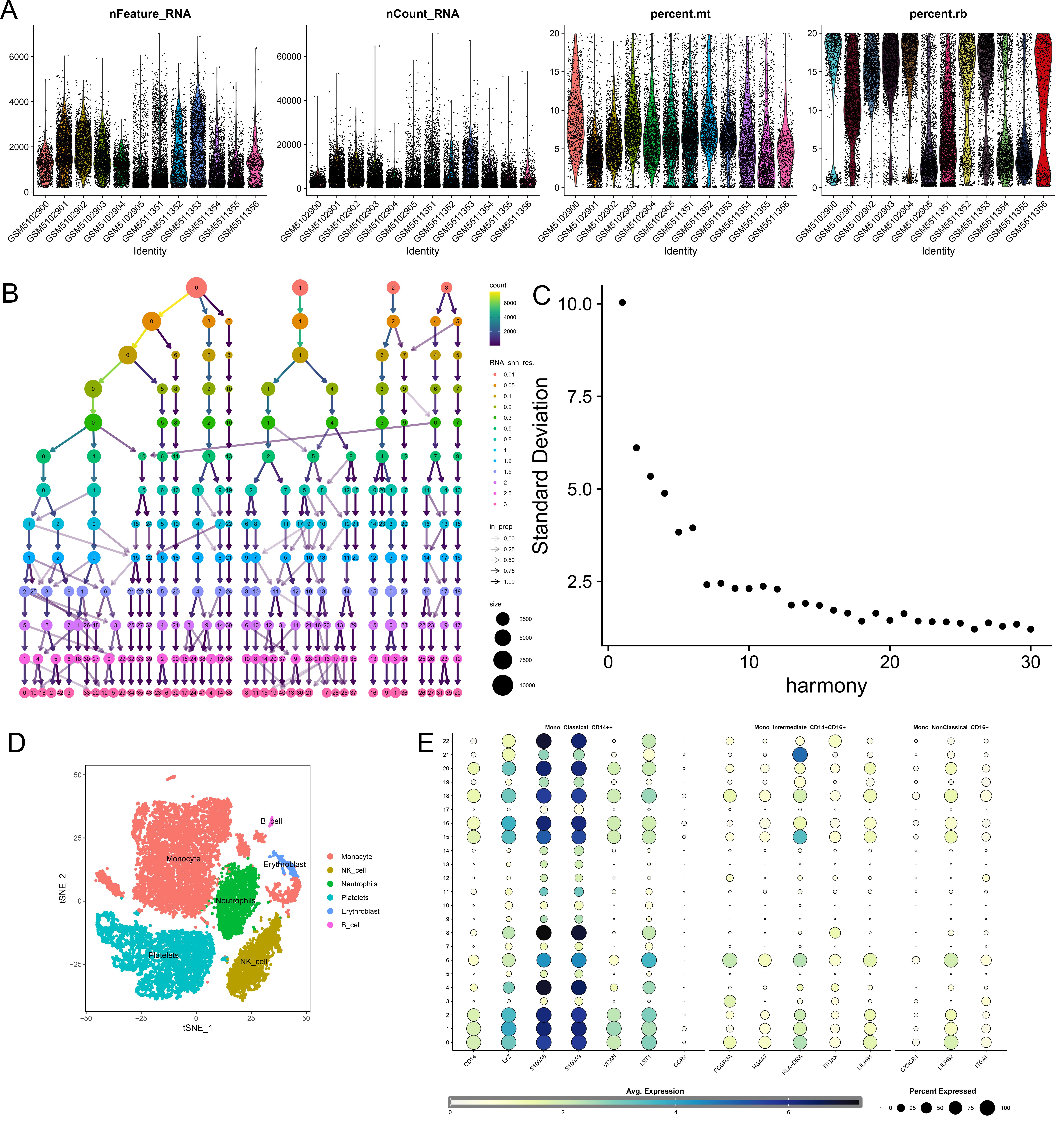

Supplement: Supplementary file 8 [file Table7.doc]
